# Effects of non-invasive respiratory support in post-operative patients: a systematic review and network meta-analysis

**DOI:** 10.1186/s13054-024-04924-0

**Published:** 2024-05-08

**Authors:** Tommaso Pettenuzzo, Annalisa Boscolo, Elisa Pistollato, Chiara Pretto, Tommaso Antonio Giacon, Sara Frasson, Francesco Maria Carbotti, Francesca Medici, Giovanni Pettenon, Giuliana Carofiglio, Marco Nardelli, Nicolas Cucci, Clara Letizia Tuccio, Veronica Gagliardi, Chiara Schiavolin, Caterina Simoni, Sabrina Congedi, Francesco Monteleone, Francesco Zarantonello, Nicolò Sella, Alessandro De Cassai, Paolo Navalesi

**Affiliations:** 1 Institute of Anesthesia and Intensive Care, University Hospital of Padua, 13 Via Gallucci, 35121 Padua, Italy; 2 Department of Medicine, University of Padua, 2 Via Giustiniani, 35128 Padua, Italy; 3 Department of Cardiac, Thoracic, Vascular Sciences, and Public Health, University of Padua, 2 Via Giustiniani, 35128 Padua, Italy

**Keywords:** Conventional oxygen therapy, Continuous positive airway pressure, High-flow nasal oxygen, Non-invasive ventilation, General anesthesia, Extubation, Post-operative respiratory failure

## Abstract

**Background:**

Re-intubation secondary to post-extubation respiratory failure in post-operative patients is associated with increased patient morbidity and mortality. Non-invasive respiratory support (NRS) alternative to conventional oxygen therapy (COT), i.e., high-flow nasal oxygen, continuous positive airway pressure, and non-invasive ventilation (NIV), has been proposed to prevent or treat post-extubation respiratory failure. Aim of the present study is assessing the effects of NRS application, compared to COT, on the re-intubation rate (primary outcome), and time to re-intubation, incidence of nosocomial pneumonia, patient discomfort, intensive care unit (ICU) and hospital length of stay, and mortality (secondary outcomes) in adult patients extubated after surgery.

**Methods:**

A systematic review and network meta-analysis of randomized and non-randomized controlled trials. A search from Medline, Embase, Scopus, Cochrane Central Register of Controlled Trials, and Web of Science from inception until February 2, 2024 was performed.

**Results:**

Thirty-three studies (11,292 patients) were included. Among all NRS modalities, only NIV reduced the re-intubation rate, compared to COT (odds ratio 0.49, 95% confidence interval 0.28; 0.87, *p* = 0.015, I^2^ = 60.5%, low certainty of evidence). In particular, this effect was observed in patients receiving NIV for treatment, while not for prevention, of post-extubation respiratory failure, and in patients at high, while not low, risk of post-extubation respiratory failure. NIV reduced the rate of nosocomial pneumonia, ICU length of stay, and ICU, hospital, and long-term mortality, while not worsening patient discomfort.

**Conclusions:**

In post-operative patients receiving NRS after extubation, NIV reduced the rate of re-intubation, compared to COT, when used for treatment of post-extubation respiratory failure and in patients at high risk of post-extubation respiratory failure.

**Supplementary Information:**

The online version contains supplementary material available at 10.1186/s13054-024-04924-0.

## Background

Re-intubation consequent to post-extubation respiratory failure in post-operative patients who underwent general anesthesia occurs in a relevant number of patients, i.e., 2.1% (range 0.2–33%) of 370,617 patients included in a recent meta-analysis [1]. The observed variability depends on a variety of factors including underlying disease, comorbidities, and type of surgery and anesthesia [1–3]. Post-operative re-intubation is associated with increased morbidity, mortality, and healthcare costs [3–5].

To prevent or treat post-extubation respiratory failure, forms of non-invasive respiratory support (NRS) alternative to conventional oxygen therapy (COT) have been proposed, i.e., high-flow nasal oxygen (HFNO), continuous positive airway pressure (CPAP), and bilevel non-invasive ventilation (NIV). These techniques aim at maintaining adequate gas exchanges, while reducing patient’s work of breathing [6, 7], and improving airway secretion clearance [8].

The 2017 European Respiratory Society (ERS)/American Thoracic Society (ATS) clinical practice guidelines recommend the use of NIV/CPAP, over COT, for both treating and preventing post-extubation respiratory failure after surgery (conditional recommendation, moderate certainty of evidence) [9]. Moreover, the 2022 ERS guidelines recommend the use of either COT or HFNO in post-operative patients at low risk of respiratory complications, and either HFNO or NIV in post-operative patients at high risk of respiratory complications (both conditional recommendations with low certainty of evidence) [10].

The aims of the present systematic review and network meta-analysis of randomized and non-randomized controlled trials are assessments of the effect of NRS application, as compared to COT, on the rate of re-intubation (primary outcome), and time to re-intubation, incidence of nosocomial pneumonia, patient discomfort, ICU and hospital length of stay, and ICU, hospital, and long-term mortality (secondary outcomes), in adult patients extubated after surgery. Additional subgroup analyses, for the rate of re-intubation only, aim to compare, as opposed to COT, the efficacy of NRS (1) for the prevention or treatment of post-operative respiratory failure after extubation; (2) for supra-diaphragmatic or infra-diaphragmatic surgery; (3) for patients at high or low risk of post-operative respiratory failure; and (4) for patients transferred to the intensive care unit (ICU) or out of the ICU, i.e., post-anesthesia care units or wards, after surgery.

## Methods

Reporting of this systematic review and meta-analysis conforms to the Preferred Reporting Items for Systematic reviews and Meta-Analysis (PRISMA) Statement extension for network meta-analysis (Additional file 1: Supplementary Digital Content [SDC] 1) [11]. The review protocol was registered in PROSPERO (CRD42022377859), an international prospective register of systematic reviews, on Dec 30, 2022.

### Literature search

An electronic search of Medline, Embase, Scopus, Cochrane Central Register of Controlled Trials, and Web of Science from inception until February 2, 2024 was performed with no language restrictions. In addition, a research-in-progress database (ClinicalTrials.gov), grey literature (OpenGrey), and all references of included articles and related reviews and guidelines were searched. Abstracts and conference proceedings were excluded. Controlled vocabulary terms (when available), text words, and keywords were variably combined with blocks of terms per concept: (“non-invasive respiratory support”) AND (“extubation” OR “weaning”) AND (“surgery” OR “general anesthesia”). MEDLINE and Scopus search strategies were adapted for searches in other databases and are reported in SDC 2.

### Study selection

All studies meeting the following Participants, Interventions, Comparisons, Outcomes, and Study design (PICOS) question were included: participants were adult patients admitted to ICU or non-ICU settings and extubated after surgery; the intervention was any NRS modality; the comparison was COT or another NRS modality; the primary outcome was all-cause re-intubation at any time-point, whereas the time to re-intubation, the incidence of nosocomial pneumonia and patient discomfort, ICU and hospital length of stay, and ICU, hospital, and long-term mortality were secondary outcomes; and eligible study designs were randomized controlled trials (RCTs) or non-randomized controlled studies. Studies not comparing at least two different NRS modalities or COT, studies investigating NRS or COT before surgery, and studies assessing NRS to facilitate early weaning from invasive mechanical ventilation were excluded. Moreover, studies exclusively on patients undergoing self-extubation or requiring palliative care and studies with cross-over design were omitted. Search results were merged and duplicate records of the same report were removed. The remaining studies were stored using Microsoft Excel software (Microsoft Corporation, Redmond, WA, USA).

### Data collection

Eight researchers (EP, CP, TAG, SF, FMC, FM, GP, GC) were split into four couples, each analyzing the same number of overall identified citations. Specifically, each member of the couple independently screened the titles and abstracts of assigned papers and retrieved the full texts of potentially relevant reports. Reasons for exclusions were detailed and excluded full texts were listed (SDC 3). Eight researchers (MN, NC, CLT, VG, CSc, CSi, SC, FM) were split into four couples, each analyzing the same number of eligible full texts. Specifically, each member of the couple independently assessed the full text of the assigned papers. Data from included studies were recorded using a Microsoft Excel specific report form. Four researchers (TP, AB, NS, FZ) independently verified all extracted data for accuracy. Any disagreements on both study selection and data extraction were resolved by referral to other authors (ADC, PN), if necessary. The following information was collected: first author, year of the study, type of surgery, inclusion and exclusion criteria, patient age and gender, risk of post-operative respiratory failure, clinical setting, NRS application for prevention or treatment, and primary and secondary outcomes.

### Quality and certainty of evidence assessment

Eight researchers (EP, CP, TAG, SF, FM, GP, CSc, CSi) were split into four couples and assessed the risk of bias of the same number of included studies. Specifically, each member of the couple independently evaluated the quality of included RCTs and non-RCTs by using the Risk of Bias (RoB) 2 and the Risk Of Bias In Non-randomized Studies of Interventions (ROBINS-I) assessment tools, respectively. The RoB2 examines five domains of bias: randomization process, deviations from intended interventions, missing outcome data, measurement of the outcome, and selection of the reported results. The study-level risk of bias is expressed on a three-grade scale, i.e., low risk of bias, high risk of bias or some concerns [12]. The ROBINS-I considers seven domains of bias: confounding, selection of participants, classification of interventions, deviations from intended interventions, missing data, measurement of outcomes, and selection of the reported result. The study-level risk of bias is described on a four-grade scale, i.e., low, moderate, serious, and critical risk of bias [13]. Disagreements were resolved by discussion with other authors (TP, PN), if necessary.

The Grades of Recommendation, Assessment, Development and Evaluation (GRADE) approach, addressing the domains of risk of bias, inconsistency, indirectness, publication bias, intransitivity, incoherence, and imprecision, was used to assess the certainty of evidence related to the each of the outcomes [14] Imprecision for each comparison was incorporated only at the network level, not at the level of the direct or indirect estimate. The most recent GRADE guidance on imprecision rating using a minimally contextualized approach was applied [15]. A partially contextualized approach was used to evaluate the magnitudes of the intervention effects [16].

### Sensitivity analyses

For the primary outcome only, a pre-planned sensitivity analysis to assess the effect on study findings of excluding RCTs at high risk of bias and non-RCTs at serious or critical risk of bias was conducted. In addition, several post-hoc sensitivity analyses were performed to assess the robustness of results after (1) excluding those studies comparing COT with a sequential combination of NRS modalities, i.e., two NRS strategies applied in sequence; (2) considering only one NRS setting at a time in those studies investigating two different HFNO flow rates or two different NIV interfaces; (3) excluding those studies requiring the application of a continuity correction, in case no events were observed in the intervention groups (see paragraph 2.7 “Statistical Analysis”); and (4) considering in the same group those studies comparing either CPAP or NIV to COT.

### Subgroup analyses

For the primary outcome only, pre-planned subgroup analyses were performed according to the following subgroups: (1) prophylactic NRS, i.e., applied immediately after extubation, versus therapeutic NRS, i.e., applied only after evidence of respiratory deterioration; (2) patients at high versus low risk of post-operative respiratory failure, as defined in the individual study; (3) studies including more than 90% of patients undergoing supra-diaphragmatic surgery versus studies including more than 90% of patients undergoing infra-diaphragmatic surgery; and (4) studies enrolling more than 90% of patients admitted to the ICU after surgery versus studies enrolling less than 10% of patients admitted to the ICU after surgery. Subgroup analyses were also performed after considering those studies comparing either CPAP or NIV to COT in the same group (see paragraph 2.5 “Sensitivity Analyses”).

### Statistical analysis

Conventional pairwise meta-analyses were performed using a random-effects model to account for between-study heterogeneity [17]. The treatment effect for dichotomous outcomes was analyzed with the Mantel–Haenszel method and expressed as odds ratio (OR) with 95% confidence interval (CI). The treatment effect for continuous outcomes was analyzed with the inverse variance method and expressed as standardized mean difference (SMD) with 95% CI. Whenever necessary, we converted reported median and interquartile range to estimated mean and standard deviation (SD) using Wan’s method [18]. When no events were observed in any of the groups in an individual study, a fixed value of 1 was added as a continuity correction to the cells corresponding to the number of events [17]. Statistical heterogeneity for the outcomes among studies was assessed using the chi-squared test and I^2^ statistic. Heterogeneity was defined as follows: low for I^2^ < 25%, moderate for I^2^ 25–50%, and high for I^2^ > 50% [17].

Availability of evidence, transitivity assumption, intra-network connectivity, and network coherence were considered to assess the feasibility of conducting a network meta-analysis [19]. The following variables were included as potential moderators in a Bayesian network meta-regression to evaluate whether the transitivity assumption was satisfied [20, 21]: prophylactic versus therapeutic NRS; patients at high versus low risk of post-operative respiratory failure; supra-diaphragmatic versus infra-diaphragmatic surgery; and ICU vs. non-ICU setting. Surface under the cumulative ranking curve (SUCRA) scatterplots indicating confidence rectangles were obtained to identify those treatment effects influenced by mediators [22]. Direct and indirect treatment estimates were compared to check for incoherence in the network meta-analyses [23].

We performed frequentist random-effects network meta-analyses [19]. The direct treatment estimates were based on the common between-study variance Tau^2^ from the network meta-analysis. The indirect estimates were obtained with the Separate Indirect from Direct Evidence and the Separate Indirect from Direct Design Evidence methods [19].

A ranking among treatments was performed based on the frequentist analogue of the SUCRA. The ranking was provided as p-scores, ranging from 0 (minimum) to 1 (maximum) and measuring the mean extent of certainty that a NRS modality is better than the competing modalities [19].

Publication bias was assessed by visually inspecting a funnel plot for potential asymmetry and Egger’s test was applied when more than 10 studies were available for a specific outcome [24].

All analyses were performed with Review Manager version 5.3 (Nordic Cochrane Centre, Cochrane Collaboration) and R version 4.3.1 (R Foundation for Statistical Computing, Vienna, Austria) with the “netmeta” package for frequentist network meta-analyses and the “rnmamod” package for Bayesian network meta-regression. For all analyses, two-sided *p* values < 0.05 were considered significant.

## Results

### Study selection, characteristics, and quality

**Fig. 1:**
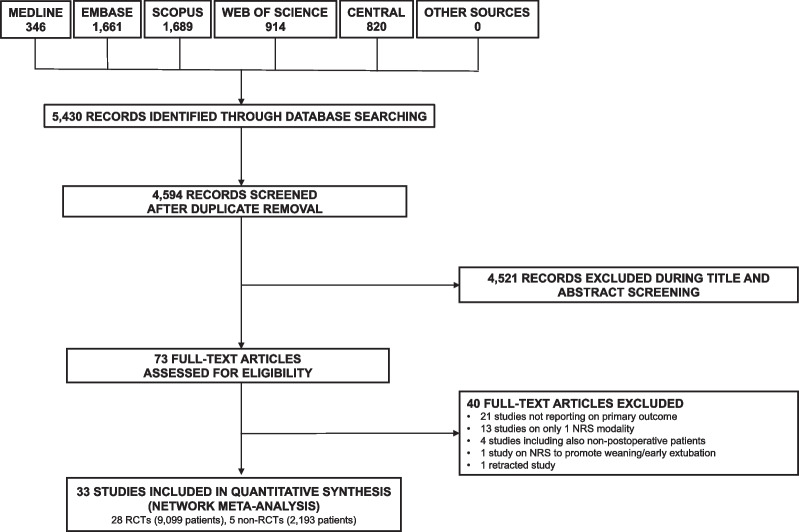
Study selection flow-chart. Abbreviations: *NRS* non-invasive respiratory support; *RCT* randomized controlled trial

The study selection flow-chart is shown in Fig. 1.

We identified 4594 unique citations and assessed the full text of 73 articles for eligibility. Of these, 28 RCTs (9099 patients) [25–52] and five non-RCTs (2193 patients) [53–57] were included in the systematic review.

The characteristics of the included studies are reported in SDC 4. Studies were published between 1997 and 2022. Study populations ranged from 20 to 4793 patients and 4285 (38%) subjects were females. Twenty-three studies (70%) were performed in the ICU, while three (9%) outside of the ICU, and 6 (18%) in a mixed setting. One study did not specify the clinical setting [32]. One study included 8% of patients admitted to the ward or high-dependency unit after surgery and was considered in the ICU group [25].

COT was administered in 5056 patients (45%), CPAP in 3130 (28%), NIV in 1814 (16%), and HFNO in 1292 (11%). NRS was used for prevention of post-operative respiratory failure after extubation in 20 studies (61%) and for treatment in 11 (33%). One study did not specify the indication for NRS [53] and another one included patients receiving either prophylactic or therapeutic NRS [45].

Only two studies (6%) investigated the use of a sequential combination of COT and CPAP [35, 51]. Therefore, these studies were not considered as a separate node of treatment. One study tested two different HFNO flow rates [46] and another tested two different NIV interfaces [49]. For two studies, the application of continuity correction was required [29, 48]. Twelve studies (36%), including 1613 patients (14%), compared COT to HFNO, 9 (27%), 5955 patients (53%), compared COT to CPAP, 9 (27%), 2574 patients (23%), compared COT to NIV, two (6%), 959 patients (8%), compared HFNO to NIV, and one (3%), 191 patients (2%), CPAP to NIV.

**Fig. 2:**
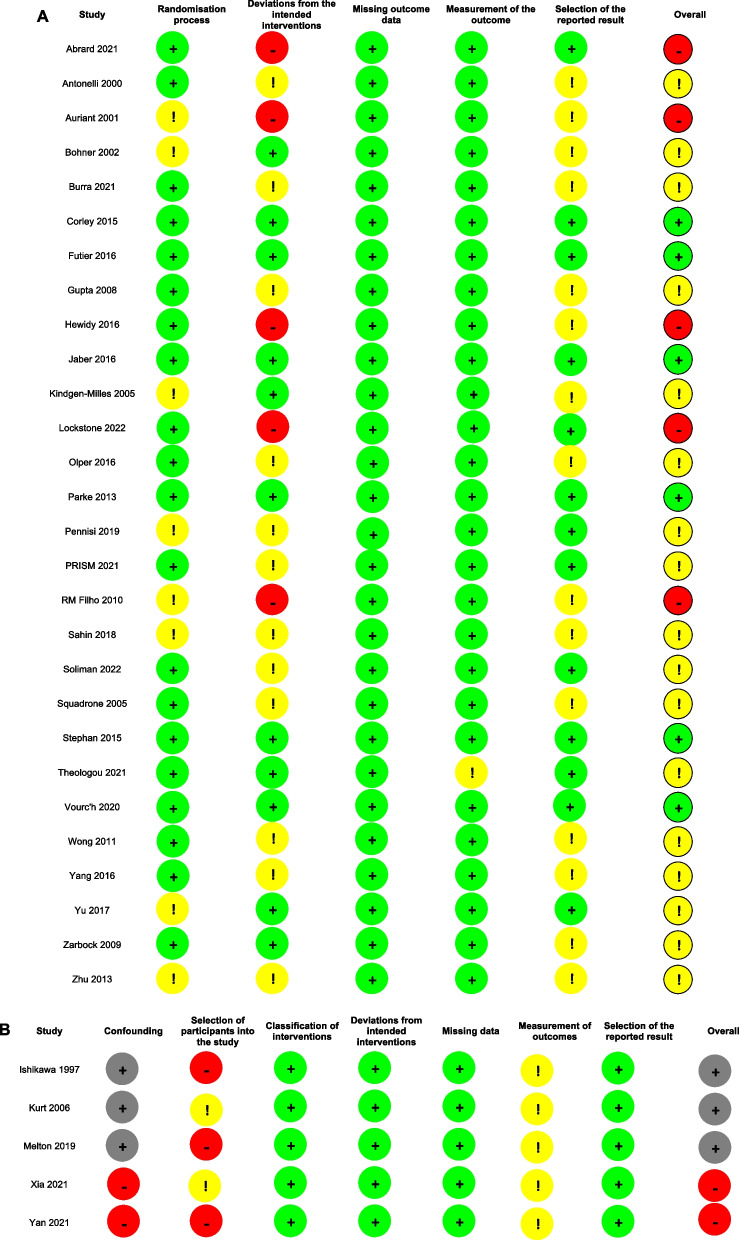
Risk of bias assessment figures for randomized (**A**) and non-randomized (**B**) controlled trials. For randomized controlled trials, green, yellow, and red circles indicate low risk of bias, some concerns, and high risk of bias, respectively. For non-randomized controlled trials, green, yellow, red, and grey circles indicate low, moderate, serious, and critical risk of bias, respectively

The risk of bias assessments are shown in Fig. 2 and SDC 5.

Among the RCTs, five studies (18%) were considered to be at high risk of bias, 16 (57%) arose some concerns, and 7 (25%) were considered to be at low risk of bias. Among the non-RCTs, three studies (60%) were considered at critical risk of bias, while two (40%) at serious risk of bias.

The presence of publication bias was strongly suspected from the visual inspection of the funnel plot for the following outcomes: nosocomial pneumonia, discomfort, ICU length of stay, and hospital length of stay (SDC 6). Egger’s test confirmed the occurrence of publication bias for nosocomial pneumonia (*p* = 0.021), ICU length of stay (*p* = 0.050), and hospital length of stay (*p* = 0.042).

As described in SDC7, SUCRA scatterplots indicated an overall consistency of the NMA with respect to the considered mediators. Only NIV treatment indicated a potential inconsistency, i.e., 50% relative overestimation when accounting for the mediation effect of prophylactic respiratory support.

As reported in SDC 8, from the comparison of direct and indirect evidence of the impact of the interventions on primary and secondary outcomes, no serious incoherence was detected.

GRADE assessment is reported in SDC 9. Overall, the certainty of evidence was rated down to low or very low for most outcomes and comparisons because of concerns related to risk of bias, publication bias, and imprecision.

### Primary and secondary outcomes

Eighteen studies reported on the incidence of nosocomial pneumonia, 9 on discomfort, 22 and 18 on ICU and hospital length of stay, respectively, 7 and 20 on ICU and hospital mortality, respectively, and 5 on long-term mortality. Since only one study provided data on the timing of re-intubation, this variable could not be included in the meta-analyses.

**Fig. 3:**
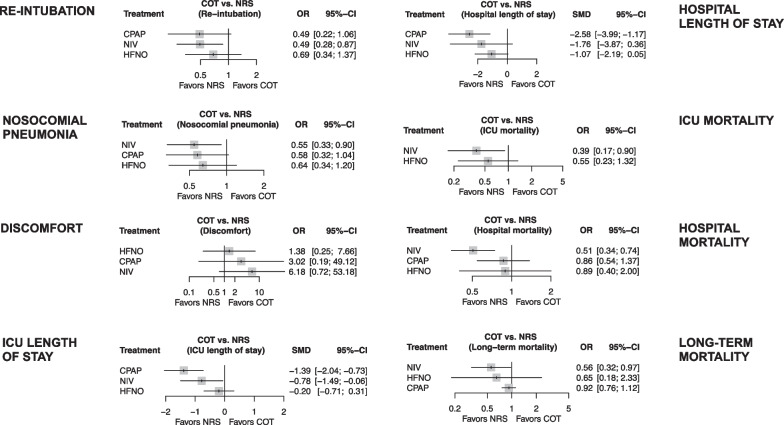
Forest plots of the effect of non-invasive respiratory support on primary and secondary outcomes. Abbreviations: *COT* conventional oxygen therapy; *NRS* non-invasive respiratory support; *OR* odds ratio; *CI* confidence interval; *CPAP* continuous positive airway pressure; *NIV* non-invasive ventilation; *HFNO* high-flow nasal oxygen; *ICU* intensive care unit; *SMD* standardized mean difference

Table 1 details network estimates evaluating the impact of the interventions on primary and secondary outcomes in the overall patient population. Figure 3 depicts the forest plots for primary and secondary outcomes in the overall patient population. Forest plots of pairwise comparisons of the effect of non-invasive respiratory support on the primary outcome are reported in SDC 10. SDC 11 depicts network diagrams for primary and secondary outcomes in the overall patient population. SDC 12 reports network estimates evaluating the impact of the interventions on the primary outcome in sensitivity analyses and patient subgroups. SDC 13 details the p-scores of the interventions for primary and secondary outcomes in the overall patient population, in sensitivity analyses, and in patient subgroups.

**Table 1:** Network estimates evaluating the impact of the interventions on primary and secondary outcomes in the overall population

Comparison	SMD or OR (95% CI)	*p* value	I^2^ (95% CI)	Tau^2^	K	GRADE	Classification of intervention^a^
*Re-intubation*							
HFNO versus COT	0.69 [0.34; 1.37]	0.284	60.5% (41.6%; 73.3%)	0.6088	12	Very low	Large beneficial effect
CPAP versus COT	0.49 [0.22; 1.06]	0.071			9	Very low	Large beneficial effect
NIV versus COT	0.49 [0.28; 0.87]	0.015			9	Low	Large beneficial effect
CPAP versus HFNO	0.71 [0.26; 1.98]	0.515			0	Very low	Large beneficial effect
HFNO versus NIV	1.39 [0.64; 3.05]	0.408			2	Very low	Large harmful effect
CPAP versus NIV	0.99 [0.40; 2.45]	0.984			1	Very low	Little to no effect
*Nosocomial pneumonia*							
HFNO versus COT	0.64 [0.34; 1.20]	0.167	31% [0.0%; 62.2%]	0.1472	4	Very low	Large beneficial effect
CPAP versus COT	0.58 [0.32; 1.04]	0.066			6	Very low	Large beneficial effect
NIV versus COT	0.55 [0.33; 0.90]	0.019			5	Low	Large beneficial effect
CPAP versus HFNO	0.90 [0.39; 2.08]	0.813			0	Very low	Small beneficial effect
HFNO versus NIV	1.17 [0.67; 2.07]	0.580			2	Very low	Moderate harmful effect
CPAP versus NIV	1.06 [0.51; 2.20]	0.872			1	Very low	Small beneficial effect
*Discomfort*							
HFNO versus COT	1.38 [0.25; 7.66]	0.714	79.1% [57.0%; 89.8%]	2.5940	4	Very low	Large harmful effect
CPAP versus COT	3.02 [0.19; 49.12]	0.438			2	Very low	Large harmful effect
NIV versus COT	6.18 [0.72; 53.18]	0.097			2	Very low	Large harmful effect
CPAP versus HFNO	2.19 [0.08; 57.84]	0.639			0	Very low	Large harmful effect
HFNO versus NIV	0.22 [0.02; 2.16]	0.196			1	Very low	Large beneficial effect
CPAP versus NIV	0.49 [0.01; 16.56]	0.690			0	Very low	Large beneficial effect
*ICU length of stay*							
HFNO versus COT	− 0.20 [− 0.71; 0.31]	0.447	84.1% [76.6%; 89.2%]	0.4219	10	Very low	–
CPAP versus COT	− 1.39 [− 2.04; − 0.73]	< 0.001			5	Very low	–
NIV versus COT	− 0.78 [− 1.49; − 0.06]	0.033			5	Very low	–
CPAP versus HFNO	− 1.19 [− 2.02; − 0.36]	0.005			0	Very low	–
HFNO versus NIV	0.58 [− 0.18; 1.34]	0.133			2	Very low	–
CPAP versus NIV	− 0.61 [− 1.58; 0.36]	0.220			0	Very low	–
*Hospital length of stay*							
HFNO versus COT	− 1.07 [− 2.19; 0.05]	0.061	92.7% [89.7%; 94.8%]	2.1523	7	Very low	–
CPAP versus COT	− 2.58 [− 3.99; − 1.17]	0.000			6	Very low	–
NIV versus COT	− 1.76 [− 3.87; 0.36]	0.104			3	Very low	–
CPAP versus HFNO	− 1.51 [− 3.31; 0.29]	0.101			0	Very low	–
HFNO versus NIV	0.69 [− 1.32; 2.70]	0.503			2	Very low	–
CPAP versus NIV	− 0.82 [− 3.36; 1.72]	0.528			0	Very low	–
*ICU mortality*							
HFNO versus COT	0.55 [0.23; 1.32]	0.182	0% [0.0%; 74.6%]	0	4	Very low	Large beneficial effect
CPAP versus COT	–	–			–	–	
NIV versus COT	0.39 [0.17; 0.90]	0.027			2	Moderate	Large beneficial effect
CPAP versus HFNO	–	–			–	–	
HFNO versus NIV	1.41 [0.82; 2.42]	0.209			1	Low	Large harmful effect
CPAP versus NIV	–	–			–	–	
*Hospital mortality*							
HFNO versus COT	0.89 [0.40; 2.00]	0.783	0% [0.0%; 48.9%]	0	7	Very low	Small beneficial effect
CPAP versus COT	0.86 [0.54; 1.37]	0.534			5	Very low	Small beneficial effect
NIV versus COT	0.51 [0.34; 0.74]	0.001			7	Low	Large beneficial effect
CPAP versus HFNO	0.97 [0.38; 2.45]	0.945			0	Very low	Little to no effect
HFNO versus NIV	1.77 [0.74; 4.23]	0.202			1	Very low	Large harmful effect
CPAP versus NIV	1.71 [0.95; 3.08]	0.074			1	Very low	Large harmful effect
*Long-term mortality*							
HFNO versus COT	0.65 [0.18; 2.33]	0.507	0% [0.0%; 89.6%]	0	0	Very low	Large beneficial effect
CPAP versus COT	0.92 [0.76; 1.12]	0.399			2	Very low	Small beneficial effect
NIV versus COT	0.56 [0.32; 0.97]	0.039			2	Moderate	Large beneficial effect
CPAP versus HFNO	1.42 [0.39; 5.16]	0.597			0	Very low	Large harmful effect
HFNO versus NIV	1.17 [0.37; 3.68]	0.793			1	Very low	Moderate harmful effect
CPAP versus NIV	1.65 [0.92; 2.98]	0.095			0	Very low	Large harmful effect

*SMD* standardized mean difference, *OR* odds ratio, *CI* confidence interval, *I*^*2*^ within-design heterogeneity, *Tau*^*2*^ between-design inconsistency, *K* number of studies providing direct evidence for each outcome, *GRADE* grades of recommendation, assessment, development and evaluation, *HFNO* high-flow nasal oxygen, *COT* conventional oxygen therapy, *CPAP* continuous positive airway pressure, *NIV* non-invasive ventilation, *ICU* intensive care unit

^a^An OR between 0.95 and 1.05 was considered as little to no effect, an OR between 1.06 and 1.15 or between 0.85 and 0.94 was considered as a small effect, an OR between 1.16 and 1.25 or between 0.75 and 0.84 was considered as a moderate effect, and an OR greater than 1.25 or smaller than 0.75 was considered as a large effect. The magnitude of effect was not expressed for SMD

As detailed in Table 1 and Fig. 3, only NIV reduced the rate of re-intubation, compared to COT (OR 0.49, 95% CI 0.28; 0.87, *p* = 0.015, I^2^ = 60.5%, low certainty of evidence). No differences were found between HFNO and CPAP, HFNO and NIV, and CPAP and NIV.

As depicted in Fig. 3, compared to COT, (1) only NIV was associated with a lower rate of nosocomial pneumonia (OR 0.55, 95% CI 0.33; 0.90, *p* = 0.019, I^2^ = 31%, low certainty of evidence); (2) both CPAP (SMD − 1.39, 95% CI − 2.04; − 0.73, *p* < 0.001, I^2^ = 84.1%, very low certainty of evidence) and NIV (SMD − 0.78, 95% CI − 1.49; − 0.06, *p* = 0.033, I^2^ = 84.1%, very low certainty of evidence) were associated with a shorter ICU length of stay, while only CPAP (SMD − 2.58, 95% CI − 3.99; − 1.17, *p* < 0.001, I^2^ = 92.7%, very low certainty) reduced hospital length of stay; and 3) only NIV reduced ICU (OR 0.39, 95% CI 0.17; 0.90, *p* = 0.027, I^2^ = 0%, moderate certainty of evidence), hospital (OR 0.51, 95% CI 0.34; 0.74, *p* = 0.001, I^2^ = 0%, low certainty of evidence), and long-term mortality (OR 0.56, 95% CI 0.32; 0.97, *p* = 0.039, I^2^ = 0%, moderate certainty of evidence). Finally, patient discomfort was similar for all interventions.

### Sensitivity analyses

As reported in Additional file 1: Supplementary Table 9 in SDC 12, compared to COT, the application of NIV significantly reduced the rate of re-intubation also when excluding (1) RCTs at high risk of bias and non-RCTs at serious or critical risk of bias; (2) studies investigating a sequential combination of NRS modalities; and (3) studies requiring the application of continuity correction. Moreover, considering only one setting in one study investigating two different HFNO flow rates and in another one using two different NIV interfaces did not change the results relative to the primary outcome.

None of the other NRS modalities were found to significantly reduce the risk of re-intubation, when compared to COT, after the performance of sensitivity analyses.

Finally, considering in the same group those studies comparing either CPAP or NIV to COT identified a significant association between the application of CPAP/NIV, compared to COT, and the rate of re-intubation (OR 0.50, 95% CI 0.32–0.78, *p* = 0.003, I^2^ = 61.3%).

### Subgroup analyses

As shown in Additional file 1: Supplementary Table 10 and Supplementary Fig. 3 in SDC 12, compared with COT, NIV was effective in reducing the risk of re-intubation only when applied for treatment (OR 0.23, 95% CI 0.09; 0.58, *p* = 0.002, I^2^ = 70.2%) and in high-risk patients (OR 0.36, 95% CI 0.14; 0.93, *p* = 0.034, I^2^ = 51.3%). This effect of NIV was observed only in patients admitted to the ICU (OR 0.48, 95% CI 0.25; 0.91, *p* = 0.024, I^2^ = 68.8%). Notably, however, no study has investigated the effect of NIV outside of the ICU. No difference in the risk of re-intubation was observed between any NRS modality and COT in supra-diaphragmatic versus infra-diaphragmatic surgery.

As reported in Additional file 1: Supplementary Table 11 and Supplementary Fig. 4 in SDC 12, subgroup analyses performed after the inclusion of those studies comparing either CPAP or NIV to COT in the same group identified a significant association of the application of CPAP/NIV, compared to COT, with a reduced rate of re-intubation only when used as treatment (OR 0.25, 95% CI 0.11; 0.60, *p* = 0.002, I^2^ = 70.1%), in the ICU (OR 0.45, 95% CI 0.24; 0.82, *p* = 0.009, I^2^ = 69.2%), and in both patients at high (OR 0.39, 95% CI 0.17; 0.90, *p* = 0.027, I^2^ = 45.2%) and low (OR 0.53, 95% CI 0.30; 0.96, *p* = 0.035, I^2^ = 68.2%) risk of post-operative respiratory failure.

## Discussion

This systematic review and network meta-analysis including 11,292 adult patients from 33 studies found that in post-operative patients receiving NRS after extubation for post-extubation respiratory failure, the risk of re-intubation was significantly reduced by NIV, as opposed to COT. In particular, this effect was observed in the following subgroups: (1) patients receiving NIV for treatment, while not for prevention, of post- extubation respiratory failure, (2) patients at high, while not low, risk of post-operative respiratory failure, (3) and ICU patients (no data available for out-of-ICU patients). In addition, in the overall patient population, NIV reduced the rate of nosocomial pneumonia, ICU length of stay, and ICU, hospital, and long-term mortality. Moreover, compared to COT, NIV did not worsen patient discomfort. Neither HFNO nor CPAP, compared to COT, significantly improved outcomes, except for the reduction in the ICU and hospital length of stay conferred by CPAP.

The 2022 ERS clinical practice guidelines on the application of HFNO in acute respiratory failure suggest the use of either COT or HFNO in post-operative patients at low risk of respiratory complications [10]. Our data confirm that COT and HFNO were equally effective in reducing the rate of re-intubation in low-risk patients and we also found CPAP and NIV, when considered separately, not to confer any improvement, compared to COT, in this patient subgroup. The same guidelines suggest, based on a single RCT not considering COT [45], that HFNO and NIV are equally effective in patients at high risk of respiratory complications [10]. Our data confirm this indication, since HFNO and NIV resulted in similar re-intubation rates in high-risk patients. Nonetheless, our network meta-analysis compared COT to each of the three NRS modalities and revealed that only NIV significantly decreased the rate of re-intubation in this subgroup of post-operative patients.

The 2017 ERS/ATS clinical practice guidelines on the application of NIV/CPAP for acute respiratory failure suggest the use of NIV/CPAP, rather than COT, for treating or preventing post-operative acute respiratory failure because of the improvement in the rate of re-intubation, nosocomial pneumonia, and mortality [9]. Furthermore, the 2020 European Society of Anesthesiology (ESA) and European Society of Intensive Care Medicine (ESICM) guidelines indicate either NIV or CPAP, over COT, to prevent re-intubation (weak recommendation, moderate certainty of evidence) and nosocomial pneumonia (weak recommendation, high‐quality evidence) and NIV rather than COT (weak recommendation, low certainty of evidence) to reduce mortality in the peri‐operative/peri-procedural hypoxemic patient [58]. Our results are in keeping with these indications in the general post-operative patient population. Noteworthy, however, in the present study only NIV was effective, compared to COT, in reducing the rate of re-intubation, nosocomial pneumonia, and mortality in the overall patient population, while CPAP only reduced ICU and hospital length of stay.

The 2017 ERS/ATS guidelines also suggest NIV should not be used for treatment of established post-extubation respiratory failure in unselected critically ill patients [9]. This recommendation does not appear to be in keeping with our results. Worth reminding, however, quite few post-operative patients, i.e., 50 patients (17% of the overall population) from two studies [59, 60], were included in the analysis leading to that guideline statement, while our network meta-analysis included data from 11,292 post-operative patients. It is important to note that atelectasis is the most frequent pulmonary complication after general anesthesia [61], occurring in approximately 90% of patients intraoperatively and potentially persisting for several days after abdominal surgery [62]. The mechanisms contributing to post-operative atelectasis [63] are readily reversible by the application of NIV [6], which explains the benefit provided by NIV observed in our study. This hypothesis is consistent with previous studies showing NIV to be effective, compared to standard treatment, when used immediately after early extubation in surgical patients with the purpose of shortening the duration of invasive mechanical ventilation [64].

Our results are in keeping with a network meta-analysis, overall including nine RCTs and 1865 patients at high-risk for or with established postoperative respiratory failure, concluding that, in comparison with COT, NIV/CPAP is associated with reduced re-intubation and mortality, while HFNO was associated with reduced re-intubation only [65]. Different from this previous study, however, we separated the effects of NIV and CPAP and included a much larger patient cohort.

A recent network meta-analysis including unselected critically ill patients found, in a subgroup of 2259 post-surgical patients, that, compared to COT, NIV/CPAP, combining both the prophylactic and therapeutic use, reduced the rate of re-intubation, while HFNO did not [66]. Our sensitivity analysis considering either CPAP or NIV, as opposed to COT, confirmed these findings in a much larger patient population (8529 patients). When considering CPAP and NIV separately, however, only NIV was beneficial.

The results of our sensitivity analysis are in accordance with those of a recent meta-analysis, including 5614 patients from four studies considering only upper abdominal surgery, which reported that post-operative prophylactic NIV/CPAP does not reduce the rate of re-intubation [67]. Nonetheless, our study also demonstrated that NIV/CPAP reduces the risk of re-intubation when used for the treatment of overt post-extubation respiratory failure.

In a recent network meta-analysis including only RCTs performed in the ICU, we found that prophylactic HFNO, while not NIV/CPAP, reduced the rate of re-intubation, as opposed to COT, in a subgroup of 544 post-operative patients from 5 RCTs [68]. Worth remarking, however, in the present work we also included non-RCTs, and out-of-ICU studies, overall resulting in 11,292 patients from 33 investigations, which definitely explains the different findings of the two network meta-analyses.

Finally, another recent meta-analysis, including 38 RCTs (9782 patients), did not report preventative post-operative non-invasive respiratory support, overall including CPAP, NIV, and HFNO, to reduce the occurrence of re-intubation, compared with usual care [69]. Notwithstanding, we considered NRS strategies separately and also included non-RCTs.

Our study has some strengths, including the use of a reproducible and comprehensive literature search strategy, comprising clinical trials and grey literature, the duplicate independent citation review, data extraction, and quality assessment, the inclusion of a large number of patients from different countries, the use of Bayesian network meta-regression to test several covariates as potential effect modifiers, and the use of multiple sensitivity analyses to assess the robustness of our results. Several limitations must also be mentioned. First, studies investigating the occurrence of all-cause re-intubation at any point during hospital stay were included, given the lack of a universally accepted definition for the timing of extubation failure. Therefore, we could not differentiate among different etiologies of respiratory failure following post-operative extubation. Second, intransitivity may have arisen from the inclusion of studies published over a 25 years period [19]; nonetheless, we did not detect significant intransitivity during our GRADE assessment. Third, low or very low certainty of evidence was attributed to most outcomes and comparisons, thus limiting the confidence in our findings. Indeed, some outcomes were characterized by high heterogeneity, possibly related to different outcome definitions among studies, variable criteria for re-intubation, and heterogeneous clinical settings and patient populations. For example, the results on patient comfort were rather surprising and need to be confirmed by further studies. Notwithstanding, our subgroup analyses partially explained this heterogeneity, indicating the scenarios where NRS may be of greater benefit over COT, while our network meta-regression did not identify further relevant sources of heterogeneity. Fourth, only nine of the included studies (27%) enrolled more than 100 patients per group, potentially introducing small-study effect biases [24]. Fifth, patients were classified at high risk of post-operative respiratory failure as defined in the original studies, in the absence of a universally applicable definition. Sixth, the inclusion of non-randomized trials may increase the risk of bias and violate the exchangeability assumption of network meta-analyses [70]. However, non-randomized studies complement some of the limitations of RCTs, increasing the generalizability of the findings, and improving network density [70]. Finally, multiple subgroup and sensitivity analyses increased the chance of type 1 error [71].

## Conclusions

In patients extubated after surgery and receiving NRS, compared to COT, only NIV reduced the rate of re-intubation and nosocomial pneumonia, and improved hospital and long-term mortality, without worsening patient discomfort. In contrast, CPAP and HFNO did not significantly improve outcomes. Therapeutic, while not prophylactic, NIV decreased the rate of re-intubation and NIV was beneficial in patients at high, rather than low, risk of post-operative respiratory failure.

### Supplementary Information

**Additional file 1.** Supplementary digital content.

## Data Availability

The datasets used and/or analyzed during the current study are available from the corresponding author on reasonable request.

## Abbreviations

NRS: Non-invasive respiratory support
COT: Conventional oxygen therapy
HFNO: High-flow nasal oxygen
CPAP: Continuous positive airway pressure
NIV: Non-invasive ventilation
ERS: European respiratory society
ATS: American thoracic society
ICU: Intensive care unit
PRISMA: Preferred reporting items for systematic reviews and meta-analysis
SDC: Supplementary digital content
PICOS: Participants, interventions, comparisons, outcomes, and study design
RCT: Randomized controlled trials
OR: Odds ratio
CI: Confidence interval
SMD: Standardized mean difference
MD: Mean difference
SUCRA: Surface under the cumulative ranking curve
RoB: Risk of bias
ROBINS-I: Risk of bias in non-randomized studies of interventions
