# Supplementary material for: Effects of non-invasive respiratory support in post-operative patients: a systematic review and network meta-analysis
Source: Crit Care. 2024 May 8;28:152. doi: 10.1186/s13054-024-04924-0 (PMC11077852; doi:10.1186/s13054-024-04924-0)
Supplement: Supplementary file 1 — Additional file 1. Supplementary digital content. [file 13054_2024_4924_MOESM1_ESM.pdf]

Effects of non-invasive respiratory support in post-operative patients: a systematic review and network meta-analysis

SUPPLEMENTARY DIGITAL CONTENT

- SDC 1. Preferred Reporting Items for Systematic reviews and Meta-Analysis (PRISMA) Statement extension for network meta-analysis checklist……….……….……….………..4
- SDC 2. Electronic search strategies……….……………….……………..………..……….10
- SDC 3. Excluded full-texts and reasons for exclusion……….……….………..……..……..13
- SDC 4. Characteristics of included studies……….……………….………..……..……..20
  - Supplementary Table 1. General study characteristics……….……………….……….20
  - Supplementary Table 2. Types of surgery……………………….……………..……...24
  - Supplementary Table 3. Patient populations……….………………….……….……...25
  - Supplementary Table 4. Outcomes……….……………………….………………..……38
- SDC 5. Risk of bias assessment……….………………………….………..……..……..43
- Risk of bias assessments for randomized controlled trials……………………………………43
  - Supplementary Figure 1. Risk of bias assessment summary for randomized controlled studies……….………………….………………….………………….………….304
  - Risk of bias assessments for non-randomized controlled trials…………………...305
- SDC 6. Funnel plots for primary and secondary outcomes………………..……..……...358
- SDC 7. Meta-regression surface under the cumulative ranking curve scatterplots……….………….………………….……………….………….……………..……359
- SDC 8. Direct and indirect evidence of the impact of the interventions……….………….………………….………………….………….………….…………362
  - Supplementary Table 5. Direct and indirect evidence of the impact of the interventions on primary and secondary outcomes in the overall population……362
  - Supplementary Figure 2. Direct and indirect evidence of the impact of the interventions on primary and secondary outcomes in the overall population……365
  - Supplementary Table 6. Direct and indirect evidence of the impact of the interventions on re-intubation in sensitivity analyses……….………….…….……….368
  - Supplementary Table 7. Direct and indirect evidence of the impact of the interventions on re-intubation in patient subgroups……….………….………….……...372

- Supplementary Table 8. Direct and indirect evidence of the impact of the interventions on re-intubation in patient subgroups when considering those studies comparing either CPAP or NIV to COT in the same group…………….………....375
- SDC 9. Grades of Recommendation, Assessment, Development and Evaluation (GRADE) assessment……………………………………………………………..………………378
- SDC 10. Forest plots of pairwise comparisons of the effect of non-invasive respiratory support on primary outcome……………………………………………..………..…….384
- SDC 11. Network diagrams for primary and secondary outcomes…………..……….…..386
- SDC 12. Network estimates evaluating the impact of the interventions………………………………………………………………….………….…..387
  - Supplementary Table 9. Network estimates evaluating the impact of the interventions on re-intubation in sensitivity analyses………………………………………..….387
  - Supplementary Table 10. Network estimates evaluating the impact of the interventions on re-intubation in patient subgroups………………………………...392
  - Supplementary Figure 3. Forest plots of the effect of NRS on re-intubation in patient subgroups……………………………………………………………….………395
  - Supplementary Table 11. Network estimates evaluating the impact of the interventions on re-intubation in patient subgroups when considering those studies comparing either CPAP or NIV to COT in the same group………….…………396
  - Supplementary Figure 4. Forest plots of the effect of NRS on re-intubation in patient subgroups when considering those studies comparing either CPAP or NIV to COT in the same group. …………………………………………………………...399
- SDC 13. P-values of the interventions……………………………..………..……...400
  - Supplementary Table 12. P-values of the interventions for primary and secondary outcomes in the overall patient population…………………………….………400
  - Supplementary Table 13. P-values of the interventions for re-intubation in sensitivity analyses……………………………………………………………………..401
  - Supplementary Table 14. P-values of the interventions for re-intubation in patient subgroups……………………………………………………………….………403
  - Supplementary Table 15. P-values of the interventions for re-intubation in patient subgroups when considering those studies comparing either CPAP or NIV to COT in the same group…………………………………………………………..…404

Supplementary Digital Content 1. Preferred Reporting Items for Systematic reviews and Meta-Analysis (PRISMA) Statement extension for network meta-analysis checklist

Section/Topic	Item #	Checklist Item	Reported on Page #
TITLE			
Title	1	Identify the report as a systematic review incorporating a network meta-analysis (or related form of meta-analysis).	1
ABSTRACT			
Structured summary	2	Provide a structured summary including, as applicable:<br>Background: main objectives<br>Methods: data sources; study eligibility criteria, participants, and interventions; study appraisal; and synthesis methods, such as network meta-analysis.<br>Results: number of studies and participants identified; summary estimates with corresponding confidence/credible intervals; treatment rankings may also be discussed. Authors may choose to summarize pairwise comparisons against a chosen treatment included in their analyses for brevity.<br>Discussion/Conclusions: limitations; conclusions and implications of findings.<br>Other: primary source of funding; systematic review registration number with registry name.	3, 4
INTRODUCTION			
Rationale	3	Describe the rationale for the review in the context of what is already known, including mention of why a network meta-analysis has been conducted.	4, 5

Objectives	4	Provide an explicit statement of questions being addressed, with reference to participants, interventions, comparisons, outcomes, and study design (PICOS).	5
METHODS			
Protocol and registration	5	Indicate whether a review protocol exists and if and where it can be accessed (e.g., Web address); and, if available, provide registration information, including registration number.	6
Eligibility criteria	6	Specify study characteristics (e.g., PICOS, length of follow-up) and report characteristics (e.g., years considered, language, publication status) used as criteria for eligibility, giving rationale. Clearly describe eligible treatments included in the treatment network, and note whether any have been clustered or merged into the same node (with justification).	6, 7
Information sources	7	Describe all information sources (e.g., databases with dates of coverage, contact with study authors to identify additional studies) in the search and date last searched.	6
Search	8	Present full electronic search strategy for at least one database, including any limits used, such that it could be repeated.	Supplementary Digital Content 2
Study selection	9	State the process for selecting studies (i.e., screening, eligibility, included in systematic review, and, if applicable, included in the meta-analysis).	6, 7
Data collection process	10	Describe method of data extraction from reports (e.g., piloted forms, independently, in duplicate) and any processes for obtaining and confirming data from investigators.	7, 8
Data items	11	List and define all variables for which data	7, 8

		were sought (e.g., PICOS, funding sources) and any assumptions and simplifications made.	
Geometry of the network	S1	Describe methods used to explore the geometry of the treatment network under study and potential biases related to it. This should include how the evidence base has been graphically summarized for presentation, and what characteristics were compiled and used to describe the evidence base to readers.	10, 11
Risk of bias within individual studies	12	Describe methods used for assessing risk of bias of individual studies (including specification of whether this was done at the study or outcome level), and how this information is to be used in any data synthesis.	8, 9
Summary measures	13	State the principal summary measures (e.g., risk ratio, difference in means). Also describe the use of additional summary measures assessed, such as treatment rankings and surface under the cumulative ranking curve (SUCRA) values, as well as modified approaches used to present summary findings from meta-analyses.	10, 11
Planned methods of analysis	14	Describe the methods of handling data and combining results of studies for each network meta-analysis. This should include, but not be limited to:<br>• Handling of multi-arm trials;<br>• Selection of variance structure;<br>• Selection of prior distributions in Bayesian analyses; and<br>• Assessment of model fit.	10, 11
Assessment of Inconsistency	S2	Describe the statistical methods used to evaluate the agreement of direct and indirect evidence in the treatment network(s) studied.	10, 11

		Describe efforts taken to address its presence when found.	
Risk of bias across studies	15	Specify any assessment of risk of bias that may affect the cumulative evidence (e.g., publication bias, selective reporting within studies).	8, 9, 11
Additional analyses	16	Describe methods of additional analyses if done, indicating which were pre-specified. This may include, but not be limited to, the following:<br>• Sensitivity or subgroup analyses;<br>• Meta-regression analyses;<br>• Alternative formulations of the treatment network; and<br>• Use of alternative prior distributions for Bayesian analyses (if applicable).	9, 10
RESULTS			
Study selection	17	Give numbers of studies screened, assessed for eligibility, and included in the review, with reasons for exclusions at each stage, ideally with a flow diagram.	12<br><br>Figure 1
Presentation of network structure	S3	Provide a network graph of the included studies to enable visualization of the geometry of the treatment network.	Supplementary Digital Content 11
Summary of network geometry	S4	Provide a brief overview of characteristics of the treatment network. This may include commentary on the abundance of trials and randomized patients for the different interventions and pairwise comparisons in the network, gaps of evidence in the treatment network, and potential biases reflected by the network structure.	12<br><br>Supplementary Digital Content 9, 10, 11

Study characteristics	18	For each study, present characteristics for which data were extracted (e.g., study size, PICOS, follow-up period) and provide the citations.	12<br><br>Supplementary Digital Content 4
Risk of bias within studies	19	Present data on risk of bias of each study and, if available, any outcome level assessment.	12, 13<br><br>Figure 2<br><br>Supplementary Digital Content 5, 9
Results of individual studies	20	For all outcomes considered (benefits or harms), present, for each study: 1) simple summary data for each intervention group, and 2) effect estimates and confidence intervals. Modified approaches may be needed to deal with information from larger networks.	Supplementary Digital Content 4, 10
Synthesis of results	21	Present results of each meta-analysis done, including confidence/credible intervals. In larger networks, authors may focus on comparisons versus a particular comparator (e.g., placebo or standard care), with full findings presented in an appendix. League tables and forest plots may be considered to summarize pairwise comparisons. If additional summary measures were explored (such as treatment rankings), these should also be presented.	13, 14<br><br>Figure 3<br><br>Supplementary Digital Content 10, 12, 13
Exploration for inconsistency	S5	Describe results from investigations of inconsistency. This may include such information as measures of model fit to compare consistency and inconsistency models, P values from statistical tests, or summary of inconsistency estimates from different parts of the treatment network.	13<br><br>Supplementary Digital Content 7, 8, 9

Risk of bias across studies	22	Present results of any assessment of risk of bias across studies for the evidence base being studied.	13<br><br>Supplementary Digital Content 6, 9
Results of additional analyses	23	Give results of additional analyses, if done (e.g., sensitivity or subgroup analyses, meta-regression analyses, alternative network geometries studied, alternative choice of prior distributions for Bayesian analyses, and so forth).	15, 16
DISCUSSION			
Summary of evidence	24	Summarize the main findings, including the strength of evidence for each main outcome; consider their relevance to key groups (e.g., healthcare providers, users, and policy-makers).	16
Limitations	25	Discuss limitations at study and outcome level (e.g., risk of bias), and at review level (e.g., incomplete retrieval of identified research, reporting bias). Comment on the validity of the assumptions, such as transitivity and consistency. Comment on any concerns regarding network geometry (e.g., avoidance of certain comparisons).	19
Conclusions	26	Provide a general interpretation of the results in the context of other evidence, and implications for future research.	20
FUNDING			
Funding	27	Describe sources of funding for the systematic review and other support (e.g., supply of data); role of funders for the systematic review. This should also include information regarding whether funding has been received from manufacturers of treatments in the network	22

		and/or whether some of the authors are content experts with professional conflicts of interest that could affect use of treatments in the network.	
Abbreviation: PICOS, population, intervention, comparators, outcomes, study design.			

Supplemental Digital Content 2. Electronic search strategies

Ovid MEDLINE(R) ALL <1946 to January 31, 2024>

1	positive pressure ventilation.mp.	8324
2	positive pressure ventilation.tw.	6675
3	bipap.mp.	878
4	bipap.tw.	830
5	face mask.mp.	4864
6	face mask.tw.	4590
7	helmet.tw.	5240
8	helmet.mp.	5316
9	nasal cannu*.mp.	3811
10	nasal cannu*.tw.	3656
11	non invasive ventilation.mp.	4192
12	non invasive ventilation.tw.	3792
13	1 or 2 or 3 or 4 or 5 or 6 or 7 or 8 or 9 or 10 or 11 or 12	25543
14	extubation.mp.	14352
15	extubation.tw.	13846
16	wean*.mp.	62688
17	wean*.tw.	57872
18	liberation.mp.	28651
19	liberation.tw.	12207
20	14 or 15 or 16 or 17 or 18 or 19	103168
21	surgery.mp.	3127714
22	surgery.tw.	1410763
23	post anesthesia.mp.	1708
24	post anesthesia.tw.	1685
25	general anesth*.mp.	50329
26	general anesth*.tw.	49760
27	21 or 22 or 23 or 24 or 25 or 26	3151477
28	13 and 20 and 27	346 citations

Embase Classic+Embase <1974 to 2024 January 31>

1	positive pressure ventilation.mp.	15322
2	positive pressure ventilation.tw.	9626
3	bipap.mp.	3036
4	bipap.tw.	2597
5	face mask.mp.	17815
6	face mask.tw.	5736
7	helmet.tw.	6537
8	helmet.mp.	9082
9	nasal cannu*.mp.	16136
10	nasal cannu*.tw.	7621
11	non invasive ventilation.mp.	9583
12	non invasive ventilation.tw.	8925
13	1 or 2 or 3 or 4 or 5 or 6 or 7 or 8 or 9 or 10 or 11 or 12	64887
14	extubation.mp.	34391
15	extubation.tw.	22492
16	wean*.mp.	86838
17	wean*.tw.	80716
18	liberation.mp.	14155
19	liberation.tw.	13396
20	14 or 15 or 16 or 17 or 18 or 19	130668
21	surgery.mp.	4408841
22	surgery.tw.	1994371
23	post anesthesia.mp.	2627
24	post anesthesia.tw.	2587
25	general anesth*.mp.	129440
26	general anesth*.tw.	69208
27	21 or 22 or 23 or 24 or 25 or 26	4461760
28	13 and 20 and 27	1661 citations

SCOPUS (until February 2, 2024) 1,689 citations

((positive AND pressure AND ventilation) OR bipap OR (face AND mask) OR helmet OR (nasal AND cannu*) OR (non AND invasive AND ventilation)) AND (extubation OR

wean* OR liberation) AND (surgery OR (general AND anesth*) OR (post AND anesthesi a))

Cochrane CENTRAL (until February 2, 2024) 820 citations

((positive AND pressure AND ventilation) OR bipap OR (face AND mask) OR helmet O R (nasal AND cannu*) OR (non AND invasive AND ventilation)) AND (extubation OR wean* OR liberation) AND (surgery OR (general AND anesth*) OR (post AND anesthesi a))

Web of Science (until February 2, 2024) 914 citations

ALL=(((positive AND pressure AND ventilation) OR bipap OR (face AND mask) OR helmet OR (nasal AND cannu*) OR (non AND invasive AND ventilation)) AND (extubation OR wean* OR liberation) AND (surgery OR (general AND anesth*) OR (post AND anesthesia)))

Supplemental Digital Content 3. Excluded full-texts and reasons for exclusion

Retracted articles

1. Ci R, Qin Y, Ci C, Zhang C, Dong S, Li M. Application Evaluation of High-Flow Humidified Oxygen in Patients with Respiratory Failure after General Anesthesia Extubation for Multiple Injuries. J Healthc Eng. 2021 Aug 12;2021:1387129. doi: 10.1155/2021/1387129. Retraction in: J Healthc Eng. 2022 Dec 14;2022:9845932. PMID: 34434537; PMCID: PMC8382541.

Studies with only one non-invasive respiratory support (NRS) modality

2. Lindner KH, Lotz P, Ahnefeld FW. Continuous positive airway pressure effect on functional residual capacity, vital capacity and its subdivisions. Chest. 1987 Jul;92(1):66-70. doi: 10.1378/chest.92.1.66. PMID: 3297521.

3. Osterkamp JTF, Strandby RB, Henningsen L, Marcussen KV, Thomsen T, Mortensen CR, Achiam MP, Jans Ø. Comparing the effects of continuous positive airway pressure via mask or helmet interface on oxygenation and pulmonary complications after major abdominal surgery: a randomized trial. J Clin Monit Comput. 2023 Feb;37(1):63-70. doi: 10.1007/s10877-022-00857-7. Epub 2022 Apr 16. PMID: 35429325; PMCID: PMC9013185.

4. Jaber S, Delay JM, Chanques G, Sebbane M, Jacquet E, Souche B, Perrigault PF, Eledjam JJ. Outcomes of patients with acute respiratory failure after abdominal surgery treated with noninvasive positive pressure ventilation. Chest. 2005 Oct;128(4):2688-95. doi: 10.1378/chest.128.4.2688. PMID: 16236943.

Studies comparing NRS with chest physiotherapy

5. Oikkonen M, Karjalainen K, Kähärä V, Kuosa R, Schavikin L. Comparison of incentive spirometry and intermittent positive pressure breathing after coronary artery bypass graft. Chest. 1991 Jan;99(1):60-5. doi: 10.1378/chest.99.1.60. PMID: 1984988.

6. Rocha MRSD, Souza S, Costa CMD, et al: Airway positive pres- sure vs. exercises with inspiratory loading focused on pulmo- nary and respiratory muscular functions in the postoperative period of bariatric surgery. Arq Bras Cir Dig 2018; 31:e1363

7. Al Jaaly E, Fiorentino F, Reeves BC, Ind PW, Angelini GD, Kemp S, Shiner RJ. Effect of adding postoperative noninvasive ventilation to usual care to prevent pulmonary complications in patients undergoing coronary artery bypass grafting: a randomized controlled trial. J Thorac Cardiovasc Surg. 2013 Oct;146(4):912-8. doi: 10.1016/j.jtcvs.2013.03.014. Epub 2013 Apr 11. PMID: 23582830.

8. Christensen EF, Schultz P, Jensen OV, Egebo K, Engberg M, Grøn I, Juhl B. Postoperative pulmonary complications and lung function in high-risk patients: a comparison of three physiotherapy regimens after upper abdominal surgery in general anesthesia. Acta Anaesthesiol Scand. 1991 Feb;35(2):97-104. doi: 10.1111/j.1399-6576.1991.tb03255.x. PMID: 2024569.

9. Ricksten SE, Bengtsson A, Soderberg C, Thorden M, Kvist H. Effects of periodic positive airway pressure by mask on postoperative pulmonary function. Chest. 1986 Jun;89(6):774-81. doi: 10.1378/chest.89.6.774. PMID: 3519107.

10. Stock MC, Downs JB, Gauer PK, Alster JM, Imrey PB. Prevention of postoperative pulmonary complications with CPAP, incentive spirometry, and conservative therapy. Chest. 1985 Feb;87(2):151-7. doi: 10.1378/chest.87.2.151. PMID: 3881226.

Studies comparing different settings of only one NRS modality

11. Franco AM, Torres FC, Simon IS, Morales D, Rodrigues AJ. Assessment of noninvasive ventilation with two levels of positive airway pressure in patients after cardiac surgery. Rev Bras Cir Cardiovasc. 2011 Oct-Dec;26(4):582-90. English, Portuguese. doi: 10.5935/1678-9741.20110048. PMID: 22358273.

12. Lotz P, Heise U, Schäffer J, Wollinsky KH. Die Wirkung einer intraoperativen PEEP-Beatmung und einer postoperative CPAP-Atmung auf die postoperative Lungenfunktion nach Oberbaucheingriffen [The effect of intraoperative PEEP ventilation and postoperative CPAP

breathing on postoperative lung function following upper abdominal surgery]. Anaesthesist. 1984 Apr;33(4):177-88. German. PMID: 6428260.

13. Cavalcanti MGO, Andrade LB, Santos PCPD, et al: Non-invasive preventive ventilation with two pressure levels in the postoperative period of Roux-en-Y gastric bypass: Randomized trial. Arq Bras Cir Dig 2018; 31:e1361

14. Denehy L, Carroll S, Ntoumenopoulos G, Jenkins S. A randomized controlled trial comparing periodic mask CPAP with physiotherapy after abdominal surgery. Physiother Res Int. 2001;6(4):236-50. doi: 10.1002/pri.231. PMID: 11833245.

Studies not reporting on primary outcome

15. Tatsuishi W, Sato T, Kataoka G, Sato A, Asano R, Nakano K. High-Flow Nasal Cannula Therapy With Early Extubation for Subjects Undergoing Off-Pump Coronary Artery Bypass Graft Surgery. Respir Care. 2020 Feb;65(2):183-190. doi: 10.4187/respcare.06382. Epub 2019 Oct 22. PMID: 31641074.

16. Twose P, Thomas C, Morgan M, Broad MA. Comparison of high-flow oxygen therapy with standard oxygen therapy for prevention of postoperative pulmonary complications after major head and neck surgery involving insertion of a tracheostomy: a feasibility study. Br J Oral Maxillofac Surg. 2019 Dec;57(10):1014-1018. doi: 10.1016/j.bjoms.2019.08.021. Epub 2019 Sep 9. PMID: 31515152.

17. Ferrando C, Puig J, Serralta F, Carrizo J, Pozo N, Arocas B, Gutierrez A, Villar J, Belda FJ, Soro M. High-flow nasal cannula oxygenation reduces postoperative hypoxemia in morbidly obese patients: a randomized controlled trial. Minerva Anestesiol. 2019 Oct;85(10):1062-1070. doi: 10.23736/S0375-9393.19.13364-0. Epub 2019 Apr 16. PMID: 30994312.

18. Brainard J, Scott BK, Sullivan BL, Fernandez-Bustamante A, Piccoli JR, Gebbink MG, Bartels K. Heated humidified high-flow nasal cannula oxygen after thoracic surgery - A randomized prospective clinical pilot trial. J Crit Care. 2017 Aug;40:225-228. doi: 10.1016/j.jcrc.2017.04.023. Epub 2017 Apr 19. PMID: 28454060; PMCID: PMC5563272.

19. Zochios V, Collier T, Blaudszun G, Butchart A, Earwaker M, Jones N, Klein AA. The effect of high-flow nasal oxygen on hospital length of stay in cardiac surgical patients at high risk for respiratory complications: a randomised controlled trial. Anaesthesia. 2018 Dec;73(12):1478-1488. doi: 10.1111/anae.14345. Epub 2018 Jul 18. PMID: 30019747; PMCID: PMC6282568.

20. Ansari BM, Hogan MP, Collier TJ, Baddeley RA, Scarci M, Coonar AS, Bottrill FE, Martinez GC, Klein AA. A Randomized Controlled Trial of High-Flow Nasal Oxygen (Optiflow) as Part of an Enhanced Recovery Program After Lung Resection Surgery. Ann Thorac Surg. 2016 Feb;101(2):459-64. doi: 10.1016/j.athoracsur.2015.07.025. Epub 2015 Sep 26. PMID: 26409713.

21. Matte P, Jacquet L, Van Dyck M, Goenen M. Effects of conventional physiotherapy, continuous positive airway pressure and non-invasive ventilatory support with bilevel positive airway pressure after coronary artery bypass grafting. Acta Anaesthesiol Scand. 2000 Jan;44(1):75-81. doi: 10.1034/j.1399-6576.2000.440114.x. PMID: 10669276.

22. Jousela I, Räsänen J, Verkkala K, Lamminen A, Mäkeläinen A, Nikki P. Continuous positive airway pressure by mask in patients after coronary surgery. Acta Anaesthesiol Scand. 1994 May;38(4):311-6. doi: 10.1111/j.1399-6576.1994.tb03899.x. PMID: 8067215.

23. Pessoa KC, Araújo GF, Pinheiro AN, Ramos MR, Maia SC. Noninvasive ventilation in the immediate postoperative of gastrojejunal derivation with Roux-en-Y gastric bypass. Rev Bras Fisioter. 2010 Jul-Aug;14(4):290-5. English, Portuguese. Epub 2010 Sep 3. PMID: 20949229.

24. Kılıç M, Şanal Baş S, Kultufan Turan S. Noninvasive Pressure Techniques on Postoperative Pulmonary Function in Patients undergoing Major Abdominal Surgery. Yoğun Bakım Derg 2017; 8: 71-6.

25. Alexandropoulou AN, Louis K, Papakonstantinou A, Tzirogiannis K, Stamataki E, Roussos C, Alchanatis M, Gratziou C, Vagiakis E, Roditis K. The influence of biphasic positive airway pressure vs. sham biphasic positive airway pressure on pulmonary function in morbidly obese patients after bariatric surgery. Anaesthesiol Intensive Ther. 2019;51(2):88-95. doi: 10.5114/ait.2019.85868. PMID: 31268268.

26. Allam, A.A.N.N.A., Elsersi, M.H.S.A., Elkady, G.A.M.A. et al. A comparative study between high-flow nasal oxygen therapy and venturi mask oxygen therapy for postoperative laparoscopic bariatric surgery patients with atelectasis: a randomized clinical trial. Ain-Shams J Anesthesiol 14, 38 (2022). https://doi.org/10.1186/s42077-022-00238-x

27. Carlsson C, Sondén B, Thylén U. Can postoperative continuous positive airway pressure (CPAP) prevent pulmonary complications after abdominal surgery? Intensive Care Med. 1981;7(5):225-9. doi: 10.1007/BF01702624. PMID: 7024383.

28. Fulton R, Millar JE, Merza M, Johnston H, Corley A, Faulke D, Rapchuk IL, Tarpey J, Fanning JP, Lockie P, Lockie S, Fraser JF. Prophylactic Postoperative High Flow Nasal Oxygen Versus Conventional Oxygen Therapy in Obese Patients Undergoing Bariatric Surgery (OXYBAR Study): a Pilot Randomised Controlled Trial. Obes Surg. 2021 Nov;31(11):4799-4807. doi: 10.1007/s11695-021-05644-y. Epub 2021 Aug 13. PMID: 34387826.

29. Gaszynski T, Tokarz A, Piotrowski D, Machala W. Boussignac CPAP in the postoperative period in morbidly obese patients. Obes Surg. 2007 Apr;17(4):452-6. doi: 10.1007/s11695-007-9079-1. PMID: 17608255.

30. Pinilla JC, Oleniuk FH, Tan L, Rebeyka I, Tanna N, Wilkinson A, Bharadwaj B. Use of a nasal continuous positive airway pressure mask in the treatment of postoperative atelectasis in aortocoronary bypass surgery. Crit Care Med. 1990 Aug;18(8):836-40. doi: 10.1097/00003246-199008000-00008. PMID: 2199148.

31. Thomas AN, Ryan JP, Doran BR, Pollard BJ. Nasal CPAP after coronary artery surgery. Anaesthesia. 1992 Apr;47(4):316-9. doi: 10.1111/j.1365-2044.1992.tb02172.x. PMID: 1519683.

32. Li XN, Zhou CC, Lin ZQ, Jia B, Li XY, Zhao GF, Ye F. High-flow nasal cannula oxygen therapy during anesthesia recovery for older orthopedic surgery patients: A prospective randomized controlled trial. World J Clin Cases. 2022 Aug 26;10(24):8615-8624. doi: 10.12998/wjcc.v10.i24.8615. PMID: 36157835; PMCID: PMC9453384.

33. Sun L, Wang J, Wei P, Ruan WQ, Guo J, Yin ZY, Li X, Song JG. Randomized Controlled Trial Investigating the Impact of High-Flow Nasal Cannula Oxygen Therapy on Patients Undergoing Robotic-Assisted Laparoscopic Rectal Cancer Surgery, with a Post-Extubation Atelectasis as a Complication. J Multidiscip Healthc. 2024 Jan 26;17:379-389. doi: 10.2147/JMDH.S449839. PMID: 38292922; PMCID: PMC10826707.

34. Ferrando C, Carramiñana A, Piñeiro P, Mirabella L, Spadaro S, Librero J, Ramasco F, Scaramuzzo G, Cervantes O, Garutti I, Parera A, Argilaga M, Herranz G, Unzueta C, Vives M, Regi K, Costa-Reverte M, Sonsoles Leal M, Nieves-Alonso J, García E, Rodríguez-Pérez A, Fariña R, Cabrera S, Guerra E, Gallego-Ligorit L, Herrero-Izquierdo A, Vallés-Torres J, Ramos S, López-Herrera D, De La Matta M, Gokhan S, Kucur E, Mugarra A, Soro M, García L, Sastre JA, Aguirre P, Salazar CJ, Ramos MC, Morocho DR, Trespalacios R, Ezequiel-Fernández F, Lamanna A, Pia Cantatore L, Laforgia D, Bellas S, López C, Navarro-Ripoll R, Martínez S, Vallverdú J, Jacas A, Yepes-Temiño MJ, Belda FJ, Tusman G, Suárez-Sipmann F, Villar J; iPROVE-OLV Research Network Group. Individualised, perioperative open-lung ventilation strategy during one-lung ventilation (iPROVE-OLV): a multicentre, randomised, controlled clinical trial. Lancet Respir Med. 2023 Dec 5:S2213-2600(23)00346-6. doi: 10.1016/S2213-2600(23)00346-6. Epub ahead of print. PMID: 38065200.

35. Imperatore F, Gritti F, Esposito R, Giudice CD, Cafora C, Pennacchio F, Maglione F, Catauro A, Pace MC, Docimo L, Gambardella C. Non-Invasive Ventilation Reduces Postoperative Respiratory Failure in Patients Undergoing Bariatric Surgery: A Retrospective Analysis. Medicina (Kaunas). 2023 Aug 12;59(8):1457. doi: 10.3390/medicina59081457. PMID: 37629747; PMCID: PMC10456476.

Studies on NRS applied to facilitate weaning

36. Lopes CR, Brandão CM, Nozawa E, Auler JO Jr. Benefits of non-invasive ventilation after extubation in the postoperative period of heart surgery. Rev Bras Cir Cardiovasc. 2008 Jul-Sep;23(3):344-50. English, Portuguese. doi: 10.1590/s0102-76382008000300010. PMID: 19082322.

Studies including also non-postoperative patients

37. De Jong A, Bignon A, Stephan F, Godet T, Constantin JM, Asehnoune K, Sylvestre A, Sautillet J, Blondonnet R, Ferrandière M, Seguin P, Lasocki S, Rollé A, Fayolle PM, Muller L, Pardo E, Terzi N, Ramin S, Jung B, Abback PS, Guerci P, Sarton B, Rozé H, Dupuis C, Cousson J, Faucher M, Lemiale V, Cholley B, Chanques G, Belafia F, Huguet H, Futier E, Azoulay E, Molinari N, Jaber S; EXTUB-OBESE trial group. Effect of non-invasive ventilation after extubation in critically ill patients with obesity in France: a multicentre, unblinded, pragmatic randomised clinical trial. Lancet Respir Med. 2023 Jun;11(6):530-539. doi: 10.1016/S2213-2600(22)00529-X. Epub 2023 Jan 21. PMID: 36693403.

38. Ge Y, Li Z, Xia A, Liu J, Zhou D. Effect of high-flow nasal cannula versus non-invasive ventilation after extubation on successful extubation in obese patients: a retrospective analysis of the MIMIC-IV database. BMJ Open Respir Res. 2023 Aug;10(1):e001737. doi: 10.1136/bmjresp-2023-001737. PMID: 37553185; PMCID: PMC10414122.

39. Tseng CW, Chao KY, Wu HL, Lin CC, Hsu HS. Effectiveness of high-flow nasal cannulae compared with noninvasive positive-pressure ventilation in preventing reintubation in patients receiving prolonged mechanical ventilation. Sci Rep. 2023 Mar 22;13(1):4689. doi: 10.1038/s41598-023-31444-8. PMID: 36949116; PMCID: PMC10033681.

40. Taran S, Diaz-Cruz C, Perrot B, Alvarez P, Godoy DA, Gurjar M, Haenggi M, Mijangos JC, Pelosi P, Robba C, Schultz MJ, Ueno Y, Asehnoune K, Cho SM, Yarnell CJ, Cinotti R, Stevens RD. Association of Noninvasive Respiratory Support with Extubation Outcomes in Brain-injured Patients Receiving Mechanical Ventilation: A Secondary Analysis of the ENIO Prospective Observational Study. Am J Respir Crit Care Med. 2023 Aug 1;208(3):270-279. doi: 10.1164/rccm.202212-2249OC. PMID: 37192445.

Supplementary Digital Content 4. Characteristics of included studies

Supplementary Table 1. General study characteristics								
**First author**	**Country**	**Study design**	**Surgery**	**Setting**	**NRS modality**	**Hypoxemia criterion**	**Risk of post-operative respiratory failure**	**Risk of bias**
Abrard, 2021	France	RCT	Cardiac or aortic surgery with CPB (80% of patients), thoracic surgery (12%), other	Ward (2% of patients), intermediate care unit (6%), or ICU (91%)	Prophylactic	No	High	High
Antonelli, 2000	Italy	RCT	Solid organ transplantion	ICU	Therapeutic	PaO_2/FIO_2 < 200 mmHg	Low-moderate	Some concerns
Auriant, 2001	France	RCT	Lung resection	ICU	Therapeutic	PaO_2/FIO_2 < 200 mmHg	Low-moderate	High
Bohner, 2002	Germany	RCT	Vascular	Intermediate care and ICU	Prophylactic	No	Not specified	Some concerns
Burra, 2021	India	RCT	Cardiac	ICU	Prophylactic	No	Not specified	Some concerns
Corley, 2015	Australia	RCT	Cardiac	ICU	Prophylactic	No	Low-moderate	Low
Futier, 2016	France	RCT	Abdominal surgery	ICU and ward	Prophylactic	No	Moderate-high	Low

Gupta, 2008	India	RCT	Laparoscopic cholecistectomy	Not specified	Therapeutic	PaO_2/FiO_2 250-300 mmHg	Not specified	Some concerns
Hewidy, 2016	Egypt	RCT	Sleeve gastrectomy	Ward	Prophylactic	No	Low-moderate	High
Ishikawa, 1997	Japan	Non-RCT	Cardiac	ICU	Not specified	No	Not specified	Critical
Jaber, 2016	France	RCT	Liver, gastric, esophageal, colorectal, pancreaticoduodenal, other	ICU	Therapeutic	PaO_2/FiO_2 < 286 mmHg	Not specified	Low
Kindgen-Milles, 2005	Germany	RCT	Thoracoabdominal aortic	ICU	Prophylactic	No	Not specified	Some concerns
Kurt, 2008	Germany	Non-RCT	Cardiac	ICU	Therapeutic	Not specified	Not specified	Critical
Lockstone, 2022	Australia	RCT	Upper abdominal surgery	ICU, PACU, and ward	Prophylactic	No	High	High
Melton, 2019	USA	Non-RCT	Cardiac	ICU	Prophylactic	No	Not specified	Critical
Olper, 2016	Italy	RCT	Cardiac	Ward	Therapeutic	PaO_2/FiO_2 100-250 mmHg	Not specified	Some concerns
Parke, 2013	New Zealand	RCT	Cardiac	ICU and ward	Prophylactic	No	Not specified	Low
Pennisi, 2019	Italy	RCT	Lung resection	ICU, PACU, and ward	Prophylactic	No	Not specified	Some concerns

PRISM, 2021	International	RCT	Intestine, hepatobiliopancreatic, stomach, bariatric, vascular, esophageal (< 1%)	ICU, PACU, and ward	Prophylactic	No	Not specified	Some concerns
RM Filho, 2010	Brazil	RCT	Cardiac	ICU	Prophylactic	No	Not specified	High
Sahin, 2018	Turkey	RCT	Cardiac	ICU	Prophylactic	No	Low-moderate	Some concerns
Soliman, 2022	Egypt	RCT	Mixed abdominal surgery	ICU	Prophylactic	No	Not specified	Some concerns
Squadrone, 2005	Italy	RCT	Colectomy, gastrectomy, pancreatico-duodenectomy, retroperitoneal mass, liver	ICU	Therapeutic	PaO_2/FiO_2 < 300 mmHg	Not specified	Low
Stephan, 2015	France	RCT	Cardiothoracic	ICU	Mixed	PaO_2/FiO_2 < 150-300 mmHg	Low-moderate	Low
Theologlu, 2021	Greece	RCT	Cardiac	ICU	Prophylactic	No	Not specified	Some concerns
Vourc'h, 2020	France	RCT	Cardiac	ICU	Therapeutic	SpO_2/FiO_2 < 192 mmHg	Not specified	Low
Wong, 2011	Canada	RCT	Bariatric	PACU and ward	Prophylactic	No	Low-moderate	Some concerns

Xia, 2021	China	Non-RCT	Esophageal	ICU	Therapeutic	100 mmHg ≤ PaO_2/FiO_2 < 300 mmHg	Not specified	Serious
Yan, 2021	China	Non-RCT	Cardiac	ICU and ward	Prophylactic	No	Not specified	Serious
Yang, 2016	China	RCT	Cardiac	ICU	Therapeutic	Not specified	Not specified	Some concerns
Yu, 2017	China	RCT	Lung lobectomy	ICU	Prophylactic	No	High	Some concerns
Zarbock, 2009	Germany	RCT	Cardiac	ICU	Prophylactic	No	Not specified	Some concerns
Zhu, 2013	China	RCT	Cardiac	ICU	Therapeutic	PaO_2 ≤ 60 or PaO_2/FiO_2 ≤ 200 mmHg	Not specified	Some concerns

Abbreviations: NRS, non-invasive respiratory support; RCT, randomized controlled trial; CPB, cardiopulmonary bypass; ICU, intensive care unit; PaO_2/FiO_2, arterial partial pressure of oxygen to fraction of inspired oxygen ratio; PACU, post-anesthesia care unit.

**Supplementary Table 2. Types of surgery**		
SUPRA-DIAPHRAGMATIC SURGERY: twenty-one studies (64%)		
Type of surgery	Number of studies	Authors
Cardiac surgery	15	Burra, Corley, Ishikawa, Kurt, Melton, Olper, Parke, RM Filho, Sahin, Theologlu, Vourc'h, Yan, Yang, Zarbock, Zhu
Lung surgery	3	Auriant, Pennisi, Yu
Cardio-thoracic surgery	2	Abrard, Stephan
Esophageal surgery	1	Xia
INFRA-DIAPHRAGMATIC SURGERY: ten studies (30%)		
Type of surgery	Number of studies	Authors
Variable abdominal surgery	6	Futier, Jaber, Lockstone, PRISM, Soliman, Squadrone
Bariatric surgery	2	Hewidy, Wong
Laparoscopic cholecystectomy	1	Gupta
Vascular surgery	1	Bohner
MIXED SURGERY: two studies (6%)		
Type of surgery	Number of studies	Authors
Solid organ transplantation	1	Antonelli
Thoraco-abdominal aortic surgery	1	Kindgen-Milles
One study included less than 10% of patients requiring digestive surgery and was considered in the supra-diaphragmatic surgery group (Abrard et al.), while two studies included less than 10% of patients requiring esophageal surgery and were considered in the infra-diaphragmatic surgery group (Jaber et al., PRISM).		

Supplementary Table 3. Patient populations					
**First author**	**Inclusion criteria**	**Exclusion criteria**	**Number of patients**	**Female (n, [%])**	**Age (years)**
Abrard, 2021	Adult patients; elective or semi-urgent procedures (>48 h between the pre-anaesthesia consultation and surgery); general or regional anaesthesia; ARISCAT score ≥ 45	Pregnancy; procedures related to previous post-operative complications; organ transplantation; preoperative intubation; outpatient procedures; inability to provide informed consent; patients under a legal protectorate	253 (COT 128, NIV 125)	50 (20)	COT: 66.8 (11.3) NIV: 68.10 (9.6)
Antonelli, 2000	Acute respiratory distress; respiratory rate > 35/min; $PaO_2/FIO_2 < 200$ mmHg while the patient was breathing oxygen through a Venturi mask; active contraction of the accessory muscles of respiration or paradoxical abdominal motion	Requirement for emergent intubation for cardiopulmonary resuscitation, respiratory arrest, severe hemodynamic instability, decreased level of consciousness; respiratory failure caused by neurological disease or status asthmaticus; more than 2 new organ failures; tracheostomy, facial deformities, or recent oral, esophageal, or gastric surgery	40 (COT 20, NIV 20)	15 (38)	COT: 44 (10) NIV: 45 (19)
Auriant, 2001	At least three of the following criteria: dyspnea at rest, defined as a respiratory rate ≥ 25 breaths/min; active contraction of the accessory respiratory muscles; PaO_2/FIO_2 ratio < 200 mmHg; chest radiographic abnormalities (alveolar condensation,	Upper airway obstruction; acute respiratory failure that required specific medical treatment (pulmonary embolism, status asthmaticus, pneumothorax); excessive secretions; respiratory arrest; need for emergency intubation; obvious excessive agitation; airways that could not be protected; unstable cardiac	48 (COT 24, NIV 24)	Not specified	COT: 63 (9) NIV: 58.9 (10)

Supplementary Table 3. Patient populations					
**First author**	**Inclusion criteria**	**Exclusion criteria**	**Number of patients**	**Female (n, [%])**	**Age (years)**
	atelectasis, or interstitial pulmonary edema)	conditions (ventricular dysrhythmia and myocardical ischemia or infarction); more than two new organ failures; pregnancy			
Bohner, 2002	Midline laparotomy for elective vascular surgery; extubation in the operation room	Emergency surgery; repair of thoracoabdominal aneurysm; retroperitoneal approach	204 (COT 105, CPAP 99)	38 (19)	COT: 64.5 (11.3) CPAP: 64.1 (12.3)
Burra, 2021	Age between 18 and 65 years; elective cardiac surgery	ASA greater than 3; BMI greater than 30 kg/m^2; pre-existing respiratory pathology; neuromuscular disorders; previous history of GERD; pregnant and lactating women; history of epistaxis; nasal pathology; emergency surgery	60 (COT 30, HFNO 30)	17 (28)	COT: 52.5 (13.9) HFNO: 50.6 (15.6)
Corley, 2015	Age ≥ 18 years; BMI ≥ 30 kg/m^2	Ventilation time > 36 h; extubation onto NIV; requirement for tracheostomy, extubation as part of end-of-life treatment	155 (COT 74, HFNO 81)	41 (26)	COT: 65 (11.1) HFNO: 63 (11.4)
Futier, 2016	Adult patients; planned or unplanned abdominal, or abdominal and thoracic, surgery with anticipated duration of ≥ 2 h; moderate to high risk of postoperative pulmonary	Lack of informed consent prior to randomisation; BMI > 35 kg/m^2; life-threatening condition requiring emergency surgery; obstructive sleep apnoea syndrome; pregnancy	220 (COT 112, HFNO 108)	95 (43)	COT: 61 (13) HFNO: 62 (12)

Supplementary Table 3. Patient populations					
**First author**	**Inclusion criteria**	**Exclusion criteria**	**Number of patients**	**Female (n, [%])**	**Age (years)**
	complications, defined by an ARISCAT risk score ≥ 26 points				
Gupta, 2008	Age 40-60 years; ASA class 1-2; laparoscopic cholecystectomy; duration of general anaesthesia 60-180 minutes; acute postoperative hypoxaemia with a PaO_2/FiO_2 ratio 250-300 mmHg	Pulmonary diseases; cardiac diseases; postoperative PaO_2/FiO_2 ratio <250 and >300 mmHg; severe hypotension; impaired consciousness	40 (COT 20, NIV 20)	14 (35)	COT: 48.35 (7.20) NIV: 47.64 (6.45)
Hewidy, 2016	Sleeve gastrectomy; BMI > 40 kg/m^2; age 18–65 years; presence of obesity for at least 3 years without hormonal problems; failure to lose weight for at least 1 year despite drug and dietary therapies; informed consent		46 (COT 22, CPAP 24)	27 (59)	COT: 26.86 (5.90) CPAP: 31.25 (10.36)
Ishikawa, 1997	Respiratory support of 72 h or more after extubation	Not specified	20 (COT 12, NIV 8)	2 (10)	Not specified
Jaber, 2016	Age > 18 years; elective or nonelective abdominal surgery under general anesthesia; acute respiratory failure occurring within 7 days of the surgical procedure, defined as the presence and	Withholding of life-sustaining treatment; contraindications to noninvasive ventilation; sleep apnea syndrome; immediate tracheal intubation; requirement for an emergent surgical procedure; previous recruitment in another trial	293 (COT 145, NIV 148)	69 (24)	COT: 64.4 (13.1) NIV: 62.5 (14.5)

Supplementary Table 3. Patient populations					
**First author**	**Inclusion criteria**	**Exclusion criteria**	**Number of patients**	**Female (n, [%])**	**Age (years)**
	persistence for more than 30 minutes of hypoxemia (defined by a partial oxygen pressure < 60 mmHg when breathing room air or < 80 mmHg when breathing 15 L/min of oxygen or an $SpO_2 \leq 90\%$ when breathing room air plus either a respiratory rate higher than 30/min or clinical signs suggestive of intense respiratory muscle work and/or labored breathing)				
Kindgen-Milles, 2005	Elective surgical repair of thoracoabdominal aortic aneurysms of types I to III (Crawford classification)	No consent was obtained; age < 18 years; pulmonary emphysema with bullae; glucocorticoid treatment; mechanical ventilation for > 48 h	50 (COT 25, CPAP 25)	21 (42)	COT: 67 (4)<br>CPAP: 66 (3)
Kurt, 2008	Extubation within 12 hours postoperatively with PaO_2/FIO_2 deterioration and without hypercapnia	Valve replacement procedures; need for immediate reintubation because of lack of consciousness and/or hypercapnia	191 (NIV 18, CPAP 173)	47 (25)	NIV: 65.1 (12.1)<br>CPAP: 64.5 (11.3)
Lockstone, 2022	English-speaking adults; laparoscopic upper abdominal surgery; high PPC risk defined by planned postsurgical	Obstructive sleep apnoea requiring overnight CPAP; extreme claustrophobia; admission for separate care episode; scheduled oesophageal or	129 (HFNO 65, NIV 64)	45 (35)	HFNO: 64 (12)<br>NIV: 66 (13)

Supplementary Table 3. Patient populations					
**First author**	**Inclusion criteria**	**Exclusion criteria**	**Number of patients**	**Female (n, [%])**	**Age (years)**
	admission to the ICU/high-dependency unit, or high-risk using the Melbourne Risk Prediction Tool	organ transplant surgery; contraindications to the first NIV application			
Melton, 2019	Not specified	NIV > 24 hours post-extubation	1718 (COT 859, NIV 859)	558 (32)	Not specified
Olper, 2016	Hypoxemic respiratory failure; admission to the cardiac surgery ward following a cardiac surgical intervention and ICU discharge	Pneumothorax; hemodynamic instability or unstable arrhythmias; seizures; severe expectoration deficit in presence of abundant secretions; hypercapnic respiratory failure with respiratory acidosis (pH < 7.30); inclusion in other randomized protocols; acute respiratory distress (respiratory rate > 30/min with activation of accessory respiratory muscles and/or $PaO_2/FiO_2 < 100$); age < 18 years; lack of informed consent.	64 (COT 31, CPAP 33)	26 (41)	COT: 66 (9.1)<br>CPAP: 65 (10.6)
Parke, 2013	Elective cardiac surgery utilizing cardiopulmonary bypass; age ≥ 18 years old; full median sternotomy	Contraindication to NHF, e.g., presence of a nasal septal defect, and previous recruitment	340 (COT 171, HFNO 169)	82 (24)	COT: 66 (21–87)<br>HFNO: 65 (19–88)

Supplementary Table 3. Patient populations					
**First author**	**Inclusion criteria**	**Exclusion criteria**	**Number of patients**	**Female (n, [%])**	**Age (years)**
Pennisi, 2019	Adult patients; elective thoracotomic pulmonary lobar resection for malignant disease	No informed consent; pregnancy; BMI ≥ 35 kg/m^2; history of obstructive sleep apnea syndrome; long-term oxygen therapy due to chronic pulmonary disease; tracheostomy; any nasal/facial defect that could impede HFNC or Venturi mask use	95 (COT 48, HFNO 47)	41 (43)	COT: 68 (9)<br>HFNO: 66 (10)
PRISM, 2021	Age ≥ 50 years; elective major intraperitoneal surgery using an open surgical technique (i.e., the incision was larger than that required to remove the surgical specimen)	No written informed consent; anticipated requirement for invasive or non-invasive mechanical ventilation for at least 4 h after surgery as part of routine care; pregnancy; previously enrolled in the PRISM trial; previously participated in another clinical trial of a treatment with a similar biological mechanism or associated primary outcome	4793 (COT 2397, CPAP 2396)	2230 (47)	COT: 67.9 (9.2)<br>CPAP: 67.8 (9-2)
RM Filho, 2010	Informed consent	Contraindication to NIV; indications for therapeutic NIV; need for NIV longer than 2 h; need for ventilatory support in the COT group	32 (COT 18, NIV 14)	13 (41)	COT: 61.0 (16.2)<br>NIV: 61.5 (9.4)
Sahin, 2018	BMI > 30 kg/m^2	Age < 18 years; hemodynamic instability; tracheostomy; obstructive sleep apnea; active	100 (COT 50, HFNO 50)	64 (64)	COT: 61.3 (8.5)<br>HFNO: 61.3 (8.5)

Supplementary Table 3. Patient populations					
**First author**	**Inclusion criteria**	**Exclusion criteria**	**Number of patients**	**Female (n, [%])**	**Age (years)**
		pulmonary disease; low cardiac output; emergent operations			
Soliman, 2022	Age 50-70 years; ASA physical status I–III	Pre-existing lung disease (pleural effusion, pneumothorax, or pulmonary atelectasis); obstructive sleep apnea; BMI ≥ 35 kg/m^2	80 (COT 40, HFNO 40)	41 (51)	COT: 59.20 (4.77) HFNO: 59.20 (5.0)
Squadrone, 2005	Elective abdominal surgery with laparotomy, time of viscera exposure longer than 90 min and general anesthesia; $PaO_2/FiO_2 \leq 300$ with 1 h from extubation during COT with Venturi mask	Age > 80 or < 18 years; New York Heart Association functional class of II, III, or IV; valvular heart disease, dilated cardiomyopathy, implanted cardiac pace-maker, unstable angina, or myocardial infarction and cardiac surgery within the previous 3 months; chronic obstructive pulmonary disease, asthma, or sleep disorders; preoperative infection, sepsis, or both; BMI > 40 kg/m^2; tracheostomy, facial, neck, or chest wall abnormalities; emergency procedure (operation that must be performed as soon as possible and no longer than 12 h after admission); abdominal aortic aneurysm surgery, chemotherapy, or immunosuppressive therapy	209 (COT 104, CPAP 105)	74 (35)	COT: 65 (10) CPAP: 66 (9)

Supplementary Table 3. Patient populations					
**First author**	**Inclusion criteria**	**Exclusion criteria**	**Number of patients**	**Female (n, [%])**	**Age (years)**
		within the previous 3 months; arterial pH < 7.30 with $PaCO_2$ > 50 mmHg; SaO_2 < 80% with the maximal FiO_2; clinical signs of acute myocardial infarction; systolic arterial pressure < 90 mmHg under optimal fluid therapy; ARDS; hemoglobin concentration <7 g/dL, serum albumin level < 3 g/dL; creatinine level > 3.5 mg/dL (309 µmol/L); GCS < 12			
Stephan, 2015	Cardiothoracic surgery; any of the following: failure of a spontaneous breathing trial, defined as SaO_2 < 90% with 12 L of oxygen during a T-tube trial or PaO_2 < 75 mmHg with a FIO_2 ≥ 50% during low level pressure support, or successful spontaneous breathing trial in patients with preexisting risk factors for postextubation acute respiratory failure (BMI > 30 kg/m^2, left ventricular ejection fraction < 40%, and failure of previous extubation), or	Obstructive sleep apnea; tracheostomy; do-not-intubate status; delirium; nausea and vomiting; bradypnea; impaired consciousness; hemodynamic instability	830 (HFNO 414, NIV 416)	279 (34)	HFNO: 63.8 (62.5-65.2) NIV: 63.9 (62.6-65.2)

**Supplementary Table 3. Patient populations**					
**First author**	**Inclusion criteria**	**Exclusion criteria**	**Number of patients**	**Female (n, [%])**	**Age (years)**
	successful spontaneous breathing trial followed by failed extubation, defined as at least one among PaO_2/FIO_2 ratio < 300 mmHg, respiratory rate > 25/min for ≥ 2 h, and use of accessory respiratory muscles or paradoxic respiration				
Theologlu, 2021	Age ≥ 18 years; alert or oriented patients; SAP 90–160 mmHg and norepinephrine infusion not exceeding 0.15 µg/kg/min; PaO_2/FiO_2 at the end of SBT < 200 mmHg	Obstructive sleep apnea syndrome requiring support with CPAP; preoperative diagnosis of COPD exacerbation; tracheostomy; do not resuscitate status; GCS score < 13; insufficient knowledge of Greek language; visual or hearing impairment	99 (COT 33, HFNO 66)	32 (32)	COT: 63.9 (62.6-65.2) HFNO: not specified
Vourc'h, 2020	Age ≥ 18 years; coronary artery bypass surgery; severe hypoxemia after extubation, defined as SpO_2 <96% with Venturi mask with FIO_2 of 50%	Pregnancy; chronic respiratory failure; combined cardiac surgery; alteration of consciousness requiring immediate intubation; surgical complications requiring reoperation; hemodynamic instability or ventricular arrhythmia; adults subject to legal protection, or	90 (COT 43, HFNO 47)	13 (14)	COT: 67.6 (9.4) HFNO: 65.8 (10.1)

Supplementary Table 3. Patient populations					
**First author**	**Inclusion criteria**	**Exclusion criteria**	**Number of patients**	**Female (n, [%])**	**Age (years)**
		already participating in an interventional study on oxygenation			
Wong, 2011	Morbidly obese patients (BMI > 35 kg/m^2); age 18-75 years; ASA 1-3; bariatric surgery	Congestive heart failure; asthma; chronic obstructive pulmonary disease or interstitial pulmonary disease; hemoglobin < 70 g/L; impaired gastric emptying; severe psychiatric disorder; language barrier	81 (COT 38, CPAP 43)	57 (70)	COT: 46.3 (10.4) CPAP: 42.9 (10.1)
Xia, 2021	Postoperative hypoxemia (100 mmHg ≤ PaO_2/FiO_2 <300 mmHg or SaO_2 <92%, RR >30 breath/min, without respiratory failure caused by other complications such as bleeding and heart failure) after weaning from mechanical ventilation	COPD, cardiogenic pulmonary edema, tracheostomy, delirium, nausea and vomiting, impaired consciousness or disorientation, hemodynamic instability, sudden cardiac arrest, moderate to severe respiratory acidosis hypercapnia (pH <7.30) combined with multiple organ dysfunctions	177 (COT 99, HFNO 78)	35	COT: 60.53 (9.39) HFNO: 62.01 (7.21)
Yan, 2021	Age > 18 years; Sun's procedure; PaO_2/FiO_2 < 300 mmHg before weaning; percentage of lung volume loss < 5% on preoperative three-dimensional computed tomography before surgery	Any surgery other than the Sun's procedure; mechanical ventilation for > 5 days; death within 24 h after surgery; low swallowing and cough reflex; severe postoperative complications, such as coma, cardiogenic shock, gastrointestinal ischemia, cardiac arrest history,	87 (COT 46, HFNO 41)	28 (20)	COT: 49.51 (11.14) HFNO: 52.11 (11.00)

Supplementary Table 3. Patient populations					
**First author**	**Inclusion criteria**	**Exclusion criteria**	**Number of patients**	**Female (n, [%])**	**Age (years)**
		and multiple organ dysfunctions; plan to use NIV after weaning			
Yang, 2016	Acute hypoxemic respiratory failure within 24 hours after extubation for Stanford type-A aortic dissection; GCS score ≥ 13; effective cough reflex; no need for sedation; absence of active bleeding of mediastinum and pericardium and intrathoracic drainage ≤ 100 mL/h; stable circulation, without necessity of administration of a large dose of vasoactive drugs (epinephrine ≤ 0.05 mcg/kg/min, dopamine ≤ 5 mcg/kg/min) and without severe arrhythmia; amount of urine ≥ 0.5 mL/kg/h and hemoglobin ≥ 90 g/L; no contraindication to NPPV	Severe cardiac insufficiency (ejection fraction < 25%); weak or stopped spontaneous breathing; conscious disturbance; high risk for aspiration, incapability of clearing oropharyngeal and upper respiratory tract secretions or incapability of effective expectoration; mechanical ventilation after extubation before NPPV; severe chronic obstructive pulmonary disease (patients who required oxygen therapy with forced expiratory volume in 1 s <50%); heart and lung transplantation; organ dysfunction (e.g., low blood pressure that was difficult to correct, severe arrhythmias, gastrointestinal perforation/hemorrhage, severe brain diseases); emphysema, pneumothorax, or undrained mediastinum; face and neck trauma, burn, malformation, or upper airway bleeding and obstruction; recent history of facial, upper	75 (COT 25, NIV 50)	29 (39)	COT: 54.3 (6.7) NIV: not specified

Supplementary Table 3. Patient populations					
**First author**	**Inclusion criteria**	**Exclusion criteria**	**Number of patients**	**Female (n, [%])**	**Age (years)**
		respiratory tract, or gastrointestinal surgery; septic shock			
Yu, 2017	Planned thoracoscopic lobectomy; intermediate to high risk for PPC (ARISCAT score ≥ 26)	Immunocompromization; pregnancy; open thoracotomy because of poor visualization or bleeding; age <18 or >80 years; no informed consent	110 (COT 54, HFNO 56)	52 (47)	COT: 55.82 (7.92) HFNO: 56.31 (7.03)
Zarbock, 2009	Coronary bypass surgery or heart valve replacement	No consent was obtained; age < 18 years; pulmonary emphysema with bullae; glucocorticoid treatment; left ventricular ejection fraction < 40%; perioperative myocardial ischemia; postoperative therapy with catecholamines; rethoracotomy; postoperative ventilation for > 18 h	468 (COT 236, CPAP 232)	114 (24)	Not specified
Zhu, 2013	ARF after initial extubation ($PaO_2 \leq 60$ mmHg after oxygen therapy through nasal prong or $PaO_2/FiO_2 \leq 200$ mmHg after oxygen therapy though a Venturi mask with or without respiratory acidosis, respiratory rate >25 breaths/min, or clinical signs of	Cardiac or respiratory arrest; lack of consciousness; high-risk of aspiration like abdominal distention or swallowing reflex abnormality; severe other organ dysfunction like refractory hypotension, severe arrhythmia, gastrointestinal perforation/bleeding, severe neurologic disease; undrained pneumothorax or	95 (COT 47, NIV 48)	36 (38)	COT: 61.0 (12.2) NIV: 62.0 (10.3)

Supplementary Table 3. Patient populations					
**First author**	**Inclusion criteria**	**Exclusion criteria**	**Number of patients**	**Female (n, [%])**	**Age (years)**
	increased respiratory workload or muscle fatigue); GCS score ≥ 13; ability to protect the airway; bleeding from mediastinal and pleural drainage < 100 ml/h; no obvious fever (temperature > 38°C) or hypothermia (temperature < 35°C); hemodynamic stability, without the need of high doses of vasopressors (epinephrine ≤ 0.05 mcg/kg/min, dopamine ≤ 5 mcg/kg/min), no severe arrhythmia, and no use of intra-aortic balloon pump; urine output ≥ 0.5 ml/kg/h.	pneumomediastinum; recent facial, esophageal, or upper airway surgery, or a facial deformity; refusal of NPPV or psychomotor agitation requiring sedation; need of immediate endotracheal intubation for excessive airway secretions			

Female gender is reported as number and percentage. Age is reported as mean (standard deviation) or median (first quartile-third quartile).

Abbreviations: ARISCAT, Assess Respiratory Risk in Surgical Patients in Catalonia; COT, conventional oxygen therapy; NIV, non-invasive ventilation; PaO_2, arterial partial pressure of oxygen; FIO_2, fraction of inspired oxygen; CPAP, continuous positive airway pressure; HFNO, high-flow nasal oxygen; ASA, American Society of Anesthesiology; BMI, body mass index; SpO2, peripheral oxygen saturation; PPC, postoperative pulmonary complications; ICU, intensive care unit; SaO2, arterial oxygen saturation; HFNC, high-flow nasal cannula; ARDS, acute respiratory distress syndrome; GCS, Glasgow coma scale; SAP, systolic arterial pressure; SBT, spontaneous breathing trial; COPD, chronic obstructive pulmonary disease; NPPV, non-invasive positive pressure ventilation; ARF, acute respiratory failure.

Supplementary Table 4. Outcomes							
**First author**	**Comparison**	**COT interface**	**CPAP/NIV interface**	**Re-intubation (n)**	**ICU mortality (n)**	**Hospital mortality (n)**	**Other secondary outcomes**
Abrard, 2021	COT vs. NIV	Not specified	Face mask	COT: 8<br>CPAP: 9		COT: 0<br>NIV: 0	ICU LOS, nosocomial pneumonia, discomfort
Antonelli, 2000	COT vs. NIV	Venturi mask	Face mask	COT: 14<br>NIV: 4	COT: 10<br>NIV: 4	COT: 11<br>NIV: 7	ICU LOS, VAP
Auriant, 2001	COT vs. NIV	Not specified	Nasal mask	COT: 12<br>NIV: 5		COT: 9<br>NIV: 3	ICU and hospital LOS, long-term mortality
Bohner, 2002	COT vs. CPAP	Face mask	Nasal mask	COT: 5<br>CPAP: 1			ICU and hospital LOS, nosocomial pneumonia
Burra, 2021	COT vs. HFNO	Nasal prongs		COT: 0<br>HFNO: 0	COT: 0<br>HFNO: 0		ICU LOS
Corley, 2015	COT vs. HFNO	Nasal prongs or face mask		COT: 1<br>HFNO: 0			ICU LOS
Futier, 2016	COT vs. HFNO	Nasal prongs or face mask		COT: 4<br>HFNO: 7		COT: 3<br>HFNO: 2	ICU and hospital LOS, nosocomial pneumonia, discomfort
Gupta, 2008	COT vs. CPAP	Venturi mask	Face mask	COT: 0<br>CPAP: 2			discomfort

Supplementary Table 4. Outcomes							
**First author**	**Comparison**	**COT interface**	**CPAP/NIV interface**	**Re-intubation (n)**	**ICU mortality (n)**	**Hospital mortality (n)**	**Other secondary outcomes**
Hewidy, 2016	COT vs. CPAP	Nasal prongs	Face mask	COT: 1<br>CPAP: 0		COT: 0<br>CPAP: 0	Nosocomial pneumonia
Ishikawa, 1997	COT vs. NIV	Face mask	Nasal mask	COT: 2<br>HFNO: 0			No
Jaber, 2016	COT vs. NIV	Not specified	Face mask	COT: 66<br>NIV: 49		COT: 22<br>NIV: 15	Time to re-intubation, ICU and hospital LOS, nosocomial pneumonia, long-term mortality
Kindgen-Milles, 2005	COT vs. CPAP	Not specified	Nasal mask	COT: 4<br>CPAP: 1		COT: 1<br>CPAP: 0	ICU and hospital LOS, nosocomial pneumonia, discomfort
Kurt, 2008	CPAP vs. NIV		Nasal mask	NIV: 4<br>CPAP: 44	NIV: 1<br>CPAP: 7		ICU and hospital LOS, nosocomial pneumonia
Lockstone, 2022	HFNO vs. NIV		Face mask	HFNO: 7<br>NIV: 3		HFNO: 4<br>NIV: 1	ICU and hospital LOS, nosocomial pneumonia, long-term mortality
Melton, 2019	COT vs. NIV	Not specified	Not specified	COT: 34<br>NIV: 35		COT: 15<br>NIV: 10	Hospital LOS, nosocomial pneumonia

Supplementary Table 4. Outcomes							
**First author**	**Comparison**	**COT interface**	**CPAP/NIV interface**	**Re-intubation (n)**	**ICU mortality (n)**	**Hospital mortality (n)**	**Other secondary outcomes**
Olper, 2016	COT vs. CPAP	Nasal prongs or Venturi mask	Oro-nasal mask	COT: 1<br>CPAP: 0			Hospital LOS, long-term mortality
Parke, 2013	COT vs. HFNO	Nasal prongs or face mask		COT: 0<br>HFNO: 2		COT: 1<br>HFNO: 1	ICU and hospital LOS, discomfort
Pennisi, 2019	COT vs. HFNO	Venturi mask		COT: 1<br>HFNO: 1		COT: 0<br>HFNO: 0	Hospital LOS
PRISM, 2021	COT vs. CPAP	Not specified	Face mask, hood, nasal mask	COT: 90<br>CPAP: 80		COT: 33<br>CPAP: 30	ICU and hospital LOS, nosocomial pneumonia, long-term mortality
RM Filho, 2010	COT vs. NIV	Not specified	Face mask	COT: 3<br>NIV: 0			No
Sahin, 2018	COT vs. HFNO	Face mask		COT: 4<br>HFNO: 0		COT: 2<br>HFNO: 0	ICU and hospital LOS, nosocomial pneumonia
Soliman, 2022	COT vs. HFNO	Face mask		COT: 2<br>HFNO: 0			ICU and hospital LOS, nosocomial pneumonia
Squadrone, 2005	COT vs. CPAP	Venturi mask	Helmet	COT: 10<br>CPAP: 1		COT: 3<br>CPAP: 0	ICU and hospital LOS, nosocomial pneumonia, discomfort

Supplementary Table 4. Outcomes							
**First author**	**Comparison**	**COT interface**	**CPAP/NIV interface**	**Re-intubation (n)**	**ICU mortality (n)**	**Hospital mortality (n)**	**Other secondary outcomes**
Stephan, 2015	HFNO vs. NIV		Face mask	HFNO: 58<br>NIV: 57	HFNO: 28<br>NIV: 23		ICU and hospital LOS, nosocomial pneumonia, discomfort
Theologlu, 2021	COT vs. HFNO	Venturi mask		COT: 2<br>HFNO: 7	COT: 1<br>HFNO: 3	COT: 1<br>HFNO: 9	ICU and hospital LOS, nosocomial pneumonia, discomfort
Vourc'h, 2020	COT vs. HFNO	Non-rebreather mask		COT: 1<br>HFNO: 3	COT: 0<br>HFNO: 0		ICU LOS, discomfort
Wong, 2011	COT vs. CPAP	Venturi mask	Boussignac face mask	COT: 0<br>CPAP: 0		COT: 0<br>CPAP: 0	No
Xia, 2021	COT vs. HFNO	Nasal prongs or face mask		COT: 8<br>HFNO: 3		COT: 3<br>HFNO: 0	Hospital LOS
Yan, 2021	COT vs. HFNO	Nasal prongs or face mask		COT: 8<br>HFNO: 1		COT: 4<br>HFNO: 2	ICU LOS
Yang, 2016	COT vs. NIV	Not specified	Face mask and helmet	COT: 9<br>CPAP: 8	COT: 5<br>CPAP: 3	COT: 5<br>CPAP: 4	ICU and hospital LOS, nosocomial pneumonia, discomfort

Supplementary Table 4. Outcomes							
**First author**	**Comparison**	**COT interface**	**CPAP/NIV interface**	**Re-intubation (n)**	**ICU mortality (n)**	**Hospital mortality (n)**	**Other secondary outcomes**
Yu, 2017	COT vs. HFNO	Nasal prongs or face mask		COT: 5<br>HFNO: 0	COT: 0<br>HFNO: 0		ICU and hospital LOS, nosocomial pneumonia, discomfort
Zarbock, 2009	COT vs. CPAP	Not specified	Nasal mask	COT: 6<br>CPAP: 3			ICU and hospital LOS, nosocomial pneumonia
Zhu, 2013	COT vs. NIV	Not specified	Face mask	COT: 38<br>NIV: 9		COT: 18<br>CPAP: 9	ICU and hospital LOS, nosocomial pneumonia
Abbreviations: COT, conventional oxygen therapy; CPAP, continuous positive airway pressure; NIV, non-invasive ventilation; ICU, intensive care unit; LOS, length of stay; HFNO, high-flow nasal oxygen.							

Supplementary Digital Content 5. Risk of bias assessment

Risk of bias assessments for randomized controlled trials

Revised Cochrane risk-of-bias tool for randomized trials (RoB 2)
TEMPLATE FOR COMPLETION

Edited by Julian PT Higgins, Jelena Savović, Matthew J Page, Jonathan AC Sterne
on behalf of the RoB2 Development Group

Version of 22 August 2019

The development of the RoB 2 tool was supported by the MRC Network of Hubs for Trials Methodology Research (MR/L004933/2- N61), with the support of the host MRC ConDuCT-II Hub (Collaboration and innovation for Difficult and Complex randomised controlled Trials In Invasive procedures - MR/K025643/1), by MRC research grant MR/M025209/1, and by a grant from The Cochrane Collaboration.

This work is licensed under a Creative Commons Attribution-NonCommercial-NoDerivatives 4.0 International License.

Study details

**Reference**	Abrard S, Rineau E, Seegers V, Lebrec N, Sargentini C, Jeanneteau A, Longeau E, Caron S, Callahan JC, Chudeau N, Beloncle F, Lasocki S, Dupoiron D. Postoperative prophylactic intermittent noninvasive ventilation versus usual postoperative care for patients at high risk of pulmonary complications: a multicentre randomised trial. Br J Anaesth. 2023 Jan;130(1):e160-e168. doi: 10.1016/j.bja.2021.11.033. Epub 2022 Jan 5. PMID: 34996593.

Study design

- X Individually-randomized parallel-group trial
- ☐ Cluster-randomized parallel-group trial
- ☐ Individually randomized cross-over (or other matched) trial

For the purposes of this assessment, the interventions being compared are defined as

Experimental:	NIV	Comparator:	Usual perioperative care with COT if required

**Specify which outcome is being assessed for risk of bias**	Re-intubation
**Specify the numerical result being assessed.** In case of multiple alternative analyses being presented, specify the numeric result (e.g. RR = 1.52 (95% CI 0.83 to 2.77) and/or a reference (e.g. to a table, figure or paragraph) that uniquely defines the result being assessed.	8/128 patients in usual care group vs. 9/125 patients in the NIV group (p = 0.76).

Is the review team's aim for this result…?

- X to assess the effect of *assignment to intervention* (the 'intention-to-treat' effect)
- ☐ to assess the effect of *adhering to intervention* (the 'per-protocol' effect)

If the aim is to assess the effect of *adhering to intervention*, select the deviations from intended intervention that should be addressed (at least one must be checked):

- ☐ occurrence of non-protocol interventions
- ☐ failures in implementing the intervention that could have affected the outcome
- ☐ non-adherence to their assigned intervention by trial participants

Which of the following sources were obtained to help inform the risk-of-bias assessment? (tick as many as apply)

- X Journal article(s) with results of the trial
- ☐ Trial protocol
- ☐ Statistical analysis plan (SAP)
- X Non-commercial trial registry record (e.g. ClinicalTrials.gov record)
- ☐ Company-owned trial registry record (e.g. GSK Clinical Study Register record)
- ☐ "Grey literature" (e.g. unpublished thesis)
- ☐ Conference abstract(s) about the trial
- ☐ Regulatory document (e.g. Clinical Study Report, Drug Approval Package)
- ☐ Research ethics application
- ☐ Grant database summary (e.g. NIH RePORTER or Research Councils UK Gateway to Research)
- ☐ Personal communication with trialist
- ☐ Personal communication with the sponsor

Risk of bias assessment

Responses underlined in green are potential markers for low risk of bias, and responses in red are potential markers for a risk of bias. Where questions relate only to sign posts to other questions, no formatting is used.

Domain 1: Risk of bias arising from the randomization process

Signalling questions	Comments	Response options
**1.1 Was the allocation sequence random?**	Citation: "Randomisation was performed before induction of anaesthesia. Treatment strategies were allocated in a 1:1 ratio. Randomisation was performed centrally, using a 100% unweighted minimisation algorithm implemented in the Ennov Clinical® (Paris, France)".	Y
**1.2 Was the allocation sequence concealed until participants were enrolled and assigned to interventions?**	Although patients and caregivers could not be blinded to the treatment allocation, it is likely that the allocation sequence was concealed until participants were enrolled and assigned to interventions.	PY
**1.3 Did baseline differences between intervention groups suggest a problem with the randomization process?**	Citation: "Patient and clinical characteristics were generally similar between groups, although obstructive sleep apnoea was more frequent in the NIV group". Being the only significant difference, it is likely this was related to chance.	PN
**Risk-of-bias judgement**	Low	Low
Optional: What is the predicted direction of bias arising from the randomization process?		NA / Favours experimental / Favours comparator / Towards null /Away from null / Unpredictable

Domain 2: Risk of bias due to deviations from the intended interventions (*effect of assignment to intervention*)

Signalling questions	Comments	Response options
**2.1. Were participants aware of their assigned intervention during the trial?**	Citation: "patients and caregivers could not be blinded to the treatment allocation"	Y
**2.2. Were carers and people delivering the interventions aware of participants' assigned intervention during the trial?**		Y
**2.3. If Y/PY/NI to 2.1 or 2.2: Were there deviations from the intended intervention that arose because of the trial context?**	Seventeen patients randomised to prophylactic non-invasive ventilation received usual care and two patients randomised to usual care received prophylactic non-invasive ventilation.<br><br>Citation: "The low daily duration of NIV (180 min/day [90-295]) compared with the target protocol requirement (360-480 min/day) is attributable to the high incidence of unplanned protocol termination in the prophylactic NIV group (58/125 patients [46.4%]), which was linked to NIV discomfort for 36 patients".	Y
**2.4 If Y/PY to 2.3: Were these deviations likely to have affected the outcome?**	The high number of patients not receiving the allocated intervention and the lower-than-expected duration of NIV sessions may have confounded the results.	PY
**2.5. If Y/PY/NI to 2.4: Were these deviations from intended intervention balanced between groups?**	No, seventeen patients randomised to prophylactic non-invasive ventilation received usual care and two patients randomised to usual care received prophylactic non-invasive ventilation.	N
**2.6 Was an appropriate analysis used to estimate the effect of assignment to intervention?**	Citation: "For primary and secondary outcomes, analyses were conducted using a modified intention-to-treat principle, considering all patients in the groups to which they had been randomly assigned regardless of the actual treatment received but excluding ineligible patients who did not undergo surgery or who were not extubated at the end of the procedure."	PY
**2.7 If N/PN/NI to 2.6: Was there potential for a substantial impact (on the result) of the failure to analyse participants in the group to which they were randomized?**		NA
**Risk-of-bias judgement**		High

Optional: What is the predicted direction of bias due to deviations from intended interventions?		NA / Favours experimental / Favours comparator / Towards null /Away from null / Unpredictable

Domain 3: Missing outcome data

Signalling questions	Comments	Response options
**3.1 Were data for this outcome available for all, or nearly all, participants randomized?**	Citation: "no patients were lost to follow-up." Furthermore, there is no mention of missing data for any patient.	PY
**3.2 If N/PN/NI to 3.1: Is there evidence that the result was not biased by missing outcome data?**		NA
**3.3 If N/PN to 3.2: Could missingness in the outcome depend on its true value?**		NA
**3.4 If Y/PY/NI to 3.3: Is it likely that missingness in the outcome depended on its true value?**		NA
**Risk-of-bias judgement**		Low
Optional: What is the predicted direction of bias due to missing outcome data?		NA / Favours experimental / Favours comparator / Towards null /Away from null / Unpredictable

Domain 4: Risk of bias in measurement of the outcome

Signalling questions	Comments	Response options
**4.1 Was the method of measuring the outcome inappropriate?**		N
**4.2 Could measurement or ascertainment of the outcome have differed between intervention groups?**	Citation: "concealment was maintained throughout outcomes evaluation (safety evaluation by the monitoring committee and primary judgement criteria by the adjudication committee). Data entry into the database and monitoring was performed by investigators who were not involved in patient care."	N
**4.3 If N/PN/NI to 4.1 and 4.2: Were outcome assessors aware of the intervention received by study participants?**	Citations: "concealment was maintained throughout outcomes evaluation (safety evaluation by the monitoring committee and primary judgement criteria by the adjudication committee). Data entry into the database and monitoring was performed by investigators who were not involved in patient care."; "The presence of acute respiratory failure was confirmed by an independent adjudication committee (at least two of the three physician assessors), which was blinded to the treatment group allocation."	PN
**4.4 If Y/PY/NI to 4.3: Could assessment of the outcome have been influenced by knowledge of intervention received?**		NA
**4.5 If Y/PY/NI to 4.4: Is it likely that assessment of the outcome was influenced by knowledge of intervention received?**		NA
**Risk-of-bias judgement**		Low
Optional: What is the predicted direction of bias in measurement of the outcome?		NA / Favours experimental / Favours comparator / Towards null /Away from null / Unpredictable

Domain 5: Risk of bias in selection of the reported result

Signalling questions	Comments	Response options
**5.1 Were the data that produced this result analysed in accordance with a pre-specified analysis plan that was finalized before unblinded outcome data were available for analysis?**	Adherence to the protocol registered in ClinicalTrial.gov.	Y
**Is the numerical result being assessed likely to have been selected, on the basis of the results, from...**		
**5.2. ... multiple eligible outcome measurements (e.g. scales, definitions, time points) within the outcome domain?**		N
**5.3 ... multiple eligible analyses of the data?**		N
**Risk-of-bias judgement**		Low
Optional: What is the predicted direction of bias due to selection of the reported result?		NA / Favours experimental / Favours comparator / Towards null /Away from null / Unpredictable

Overall risk of bias

**Risk-of-bias judgement**		High
Optional: What is the overall predicted direction of bias for this outcome?		NA / Favours experimental / Favours comparator / Towards null /Away from null / Unpredictable

This work is licensed under a Creative Commons Attribution-NonCommercial-NoDerivatives 4.0 International License.

Revised Cochrane risk-of-bias tool for randomized trials (RoB 2) TEMPLATE FOR COMPLETION

Edited by Julian PT Higgins, Jelena Savović, Matthew J Page, Jonathan AC Sterne
on behalf of the RoB2 Development Group

Version of 22 August 2019

The development of the RoB 2 tool was supported by the MRC Network of Hubs for Trials Methodology Research (MR/L004933/2- N61), with the support of the host MRC ConDuCT-II Hub (Collaboration and innovation for Difficult and Complex randomised controlled Trials In Invasive procedures - MR/K025643/1), by MRC research grant MR/M025209/1, and by a grant from The Cochrane Collaboration.

This work is licensed under a Creative Commons Attribution-NonCommercial-NoDerivatives 4.0 International License.

Study details

**Reference**	Antonelli M, Conti G, Bufi M, Costa MG, Lappa A, Rocco M, Gasparetto A, Meduri GU. Noninvasive ventilation for treatment of acute respiratory failure in patients undergoing solid organ transplantation: a randomized trial. JAMA. 2000 Jan 12;283(2):235-41. doi: 10.1001/jama.283.2.235. PMID: 10634340.

Study design

X Individually-randomized parallel-group trial
☐ Cluster-randomized parallel-group trial
☐ Individually randomized cross-over (or other matched) trial

For the purposes of this assessment, the interventions being compared are defined as

Experimental: NIV Comparator: COT

**Specify which outcome is being assessed for risk of bias**	Re-intubation
**Specify the numerical result being assessed.** In case of multiple alternative analyses being presented, specify the numeric result (e.g. RR = 1.52 (95% CI 0.83 to 2.77) and/or a reference (e.g. to a table, figure or paragraph) that uniquely defines the result being assessed.	14/20 patients in the COT group vs. 4/20 patients in the NIV group (p = 0.002)

Is the review team's aim for this result…?

X to assess the effect of *assignment to intervention* (the 'intention-to-treat' effect)
☐ to assess the effect of *adhering to intervention* (the 'per-protocol' effect)

If the aim is to assess the effect of *adhering to intervention*, select the deviations from intended intervention that should be addressed (at least one must be checked):

☐ occurrence of non-protocol interventions
☐ failures in implementing the intervention that could have affected the outcome
☐ non-adherence to their assigned intervention by trial participants

Which of the following sources were <u>obtained</u> to help inform the risk-of-bias assessment? (tick as many as apply)

X Journal article(s) with results of the trial

☐ Trial protocol

☐ Statistical analysis plan (SAP)

☐ Non-commercial trial registry record (e.g. ClinicalTrials.gov record)

☐ Company-owned trial registry record (e.g. GSK Clinical Study Register record)

☐ "Grey literature" (e.g. unpublished thesis)

☐ Conference abstract(s) about the trial

☐ Regulatory document (e.g. Clinical Study Report, Drug Approval Package)

☐ Research ethics application

☐ Grant database summary (e.g. NIH RePORTER or Research Councils UK Gateway to Research)

☐ Personal communication with trialist

☐ Personal communication with the sponsor

Risk of bias assessment

Responses underlined in green are potential markers for low risk of bias, and responses in red are potential markers for a risk of bias. Where questions relate only to sign posts to other questions, no formatting is used.

Domain 1: Risk of bias arising from the randomization process

Signalling questions	Comments	Response options
**1.1 Was the allocation sequence random?**	Citation: “Patients enrolled were randomly assigned to receive either standard treatment with oxygen supple- mentation delivered by Venturi mask or NIV through a face mask. Computer-generated random assignments were concealed in sealed envelopes”.	Y
**1.2 Was the allocation sequence concealed until participants were enrolled and assigned to interventions?**		PY
**1.3 Did baseline differences between intervention groups suggest a problem with the randomization process?**	Citation: “The baseline characteristics of the 2 groups were similar”.	N
**Risk-of-bias judgement**		Low
Optional: What is the predicted direction of bias arising from the randomization process?		NA / Favours experimental / Favours comparator / Towards null /Away from null / Unpredictable

Domain 2: Risk of bias due to deviations from the intended interventions (*effect of assignment to intervention*)

Signalling questions	Comments	Response options
**2.1. Were participants aware of their assigned intervention during the trial?**	Citation: “obvious difficulty of blinding in this study”.	Y
**2.2. Were carers and people delivering the interventions aware of participants' assigned intervention during the trial?**		Y
**2.3. If Y/PY/NI to 2.1 or 2.2: Were there deviations from the intended intervention that arose because of the trial context?**	No deviations from the intended interventions are reported.	PN
**2.4 If Y/PY to 2.3: Were these deviations likely to have affected the outcome?**		NA
**2.5. If Y/PY/NI to 2.4: Were these deviations from intended intervention balanced between groups?**		NA
**2.6 Was an appropriate analysis used to estimate the effect of assignment to intervention?**		NI
**2.7 If N/PN/NI to 2.6: Was there potential for a substantial impact (on the result) of the failure to analyse participants in the group to which they were randomized?**		PN
**Risk-of-bias judgement**		Some concerns
Optional: What is the predicted direction of bias due to deviations from intended interventions?		NA / Favours experimental / Favours comparator / Towards null /Away from null / Unpredictable

Domain 3: Missing outcome data

Signalling questions	Comments	Response options
**3.1 Were data for this outcome available for all, or nearly all, participants randomized?**	Citation: "Twenty patients were assigned to each group and all completed the study and follow-up".	Y
**3.2 If N/PN/NI to 3.1: Is there evidence that the result was not biased by missing outcome data?**		NA
**3.3 If N/PN to 3.2: Could missingness in the outcome depend on its true value?**		NA
**3.4 If Y/PY/NI to 3.3: Is it likely that missingness in the outcome depended on its true value?**		NA
**Risk-of-bias judgement**		Low
Optional: What is the predicted direction of bias due to missing outcome data?		NA / Favours experimental / Favours comparator / Towards null /Away from null / Unpredictable

Domain 4: Risk of bias in measurement of the outcome

Signalling questions	Comments	Response options
**4.1 Was the method of measuring the outcome inappropriate?**		N
**4.2 Could measurement or ascertainment of the outcome have differed between intervention groups?**	Although there is not explicit mention that concealment was maintained throughout outcomes evaluation and outcome assessment was performed by investigators who were not involved in patient care, it is unlikely that the measurement or ascertainment of the outcome differed between intervention groups.	N
**4.3 If N/PN/NI to 4.1 and 4.2: Were outcome assessors aware of the intervention received by study participants?**	There is not explicit mention that concealment was maintained throughout outcomes evaluation and outcome assessment was performed by investigators who were not involved in patient care.	Y
**4.4 If Y/PY/NI to 4.3: Could assessment of the outcome have been influenced by knowledge of intervention received?**	Although there is not explicit mention that concealment was maintained throughout outcomes evaluation and outcome assessment was performed by investigators who were not involved in patient care, it is unlikely that the measurement or ascertainment of the outcome differed between intervention groups, considering that re-intubation is an outcome that does not involve any judgement.	PN
**4.5 If Y/PY/NI to 4.4: Is it likely that assessment of the outcome was influenced by knowledge of intervention received?**		NA
**Risk-of-bias judgement**		Low
Optional: What is the predicted direction of bias in measurement of the outcome?		NA / Favours experimental / Favours comparator / Towards null /Away from null / Unpredictable

Domain 5: Risk of bias in selection of the reported result

Signalling questions	Comments	Response options
**5.1 Were the data that produced this result analysed in accordance with a pre-specified analysis plan that was finalized before unblinded outcome data were available for analysis?**	There is no mention that the prespecified analysis plan was published.	NI
**Is the numerical result being assessed likely to have been selected, on the basis of the results, from...**		
**5.2. ... multiple eligible outcome measurements (e.g. scales, definitions, time points) within the outcome domain?**		N
**5.3 ... multiple eligible analyses of the data?**		N
**Risk-of-bias judgement**		Some concerns
Optional: What is the predicted direction of bias due to selection of the reported result?		NA / Favours experimental / Favours comparator / Towards null /Away from null / Unpredictable

Overall risk of bias

**Risk-of-bias judgement**		Some concerns
Optional: What is the overall predicted direction of bias for this outcome?		NA / Favours experimental / Favours comparator / Towards null /Away from null / Unpredictable

This work is licensed under a Creative Commons Attribution-NonCommercial-NoDerivatives 4.0 International License.

Revised Cochrane risk-of-bias tool for randomized trials (RoB 2)
TEMPLATE FOR COMPLETION

Edited by Julian PT Higgins, Jelena Savović, Matthew J Page, Jonathan AC Sterne
on behalf of the RoB2 Development Group

Version of 22 August 2019

The development of the RoB 2 tool was supported by the MRC Network of Hubs for Trials Methodology Research (MR/L004933/2- N61), with the support of the host MRC ConDuCT-II Hub (Collaboration and innovation for Difficult and Complex randomised controlled Trials In Invasive procedures - MR/K025643/1), by MRC research grant MR/M025209/1, and by a grant from The Cochrane Collaboration.

This work is licensed under a Creative Commons Attribution-NonCommercial-NoDerivatives 4.0 International License.

Study details

Reference

Auriant I, Jallot A, Hervé P, Cerrina J, Le Roy Ladurie F, Fournier JL, Lescot B, Parquin F. Noninvasive ventilation reduces mortality in acute respiratory failure following lung resection. Am J Respir Crit Care Med. 2001 Oct 1;164(7):1231-5. doi: 10.1164/ajrccm.164.7.2101089. PMID: 11673215.

Study design

- X Individually-randomized parallel-group trial
- ☐ Cluster-randomized parallel-group trial
- ☐ Individually randomized cross-over (or other matched) trial

For the purposes of this assessment, the interventions being compared are defined as

Experimental: NIV Comparator: COT

Specify which outcome is being assessed for risk of bias

Re-intubation

Specify the numerical result being assessed. In case of multiple alternative analyses being presented, specify the numeric result (e.g. RR = 1.52 (95% CI 0.83 to 2.77) and/or a reference (e.g. to a table, figure or paragraph) that uniquely defines the result being assessed.

12/24 patients in the COT group vs. 5/24 patients in the NIV group (p = 0.035)

Is the review team's aim for this result…?

- X to assess the effect of *assignment to intervention* (the 'intention-to-treat' effect)
- ☐ to assess the effect of *adhering to intervention* (the 'per-protocol' effect)

If the aim is to assess the effect of *adhering to intervention*, select the deviations from intended intervention that should be addressed (at least one must be checked):

- ☐ occurrence of non-protocol interventions
- ☐ failures in implementing the intervention that could have affected the outcome
- ☐ non-adherence to their assigned intervention by trial participants

Which of the following sources were <u>obtained</u> to help inform the risk-of-bias assessment? (tick as many as apply)

X Journal article(s) with results of the trial
☐ Trial protocol
☐ Statistical analysis plan (SAP)
☐ Non-commercial trial registry record (e.g. ClinicalTrials.gov record)
☐ Company-owned trial registry record (e.g. GSK Clinical Study Register record)
☐ "Grey literature" (e.g. unpublished thesis)
☐ Conference abstract(s) about the trial
☐ Regulatory document (e.g. Clinical Study Report, Drug Approval Package)
☐ Research ethics application
☐ Grant database summary (e.g. NIH RePORTER or Research Councils UK Gateway to Research)
☐ Personal communication with trialist
☐ Personal communication with the sponsor

Risk of bias assessment

Responses underlined in green are potential markers for low risk of bias, and responses in red are potential markers for a risk of bias. Where questions relate only to sign posts to other questions, no formatting is used.

Domain 1: Risk of bias arising from the randomization process

Signalling questions	Comments	Response options
**1.1 Was the allocation sequence random?**	Citation: "Patients were randomly assigned to standard treatment with or without NPPV".	PY
**1.2 Was the allocation sequence concealed until participants were enrolled and assigned to interventions?**	There is no mention regarding the allocation sequence concealment.	NI
**1.3 Did baseline differences between intervention groups suggest a problem with the randomization process?**	Citation: "Inclusion characteristics were similar in the two groups except for a higher mean PaCO2 and lower mean arterial pH in the NPPV group".	PN
**Risk-of-bias judgement**		Some concerns
Optional: What is the predicted direction of bias arising from the randomization process?		NA / Favours experimental / Favours comparator / Towards null /Away from null / Unpredictable

Domain 2: Risk of bias due to deviations from the intended interventions (*effect of assignment to intervention*)

Signalling questions	Comments	Response options
**2.1. Were participants aware of their assigned intervention during the trial?**	Citation: "It is difficult to eliminate a bias when a study cannot be blinded"	Y
**2.2. Were carers and people delivering the interventions aware of participants' assigned intervention during the trial?**		Y
**2.3. If Y/PY/NI to 2.1 or 2.2: Were there deviations from the intended intervention that arose because of the trial context?**	The study was stopped after an unspecified interim analysis.	PY
**2.4 If Y/PY to 2.3: Were these deviations likely to have affected the outcome?**		PY
**2.5. If Y/PY/NI to 2.4: Were these deviations from intended intervention balanced between groups?**		NI
**2.6 Was an appropriate analysis used to estimate the effect of assignment to intervention?**		NI
**2.7 If N/PN/NI to 2.6: Was there potential for a substantial impact (on the result) of the failure to analyse participants in the group to which they were randomized?**		PY
**Risk-of-bias judgement**		High
Optional: What is the predicted direction of bias due to deviations from intended interventions?		NA / Favours experimental / Favours comparator / Towards null /Away from null / Unpredictable

Domain 3: Missing outcome data

Signalling questions	Comments	Response options
**3.1 Were data for this outcome available for all, or nearly all, participants randomized?**		Y
**3.2 If N/PN/NI to 3.1: Is there evidence that the result was not biased by missing outcome data?**		NA
**3.3 If N/PN to 3.2: Could missingness in the outcome depend on its true value?**		NA
**3.4 If Y/PY/NI to 3.3: Is it likely that missingness in the outcome depended on its true value?**		NA
**Risk-of-bias judgement**		Low
Optional: What is the predicted direction of bias due to missing outcome data?		NA / Favours experimental / Favours comparator / Towards null /Away from null / Unpredictable

Domain 4: Risk of bias in measurement of the outcome

Signalling questions	Comments	Response options
**4.1 Was the method of measuring the outcome inappropriate?**		N
**4.2 Could measurement or ascertainment of the outcome have differed between intervention groups?**	Although there is not explicit mention that concealment was maintained throughout outcomes evaluation and outcome assessment was performed by investigators who were not involved in patient care, it is unlikely that the measurement or ascertainment of the outcome differed between intervention groups.	N
**4.3 If N/PN/NI to 4.1 and 4.2: Were outcome assessors aware of the intervention received by study participants?**	There is not explicit mention that concealment was maintained throughout outcomes evaluation and outcome assessment was performed by investigators who were not involved in patient care.	Y
**4.4 If Y/PY/NI to 4.3: Could assessment of the outcome have been influenced by knowledge of intervention received?**	Although there is not explicit mention that concealment was maintained throughout outcomes evaluation and outcome assessment was performed by investigators who were not involved in patient care, it is unlikely that the measurement or ascertainment of the outcome differed between intervention groups, considering that re-intubation is an outcome that does not involve any judgement.	PN
**4.5 If Y/PY/NI to 4.4: Is it likely that assessment of the outcome was influenced by knowledge of intervention received?**		NA
**Risk-of-bias judgement**		Low
Optional: What is the predicted direction of bias in measurement of the outcome?		NA / Favours experimental / Favours comparator / Towards null /Away from null / Unpredictable

Domain 5: Risk of bias in selection of the reported result

Signalling questions	Comments	Response options
**5.1 Were the data that produced this result analysed in accordance with a pre-specified analysis plan that was finalized before unblinded outcome data were available for analysis?**	There is no mention that the prespecified analysis plan was published.	NI
**Is the numerical result being assessed likely to have been selected, on the basis of the results, from...**		
**5.2. ... multiple eligible outcome measurements (e.g. scales, definitions, time points) within the outcome domain?**		N
**5.3 ... multiple eligible analyses of the data?**		N
**Risk-of-bias judgement**		Some concerns
Optional: What is the predicted direction of bias due to selection of the reported result?		NA / Favours experimental / Favours comparator / Towards null /Away from null / Unpredictable

Overall risk of bias

**Risk-of-bias judgement**		High
Optional: What is the overall predicted direction of bias for this outcome?		NA / Favours experimental / Favours comparator / Towards null /Away from null / Unpredictable

This work is licensed under a Creative Commons Attribution-NonCommercial-NoDerivatives 4.0 International License.

Revised Cochrane risk-of-bias tool for randomized trials (RoB 2)
TEMPLATE FOR COMPLETION

Edited by Julian PT Higgins, Jelena Savović, Matthew J Page, Jonathan AC Sterne
on behalf of the RoB2 Development Group

Version of 22 August 2019

The development of the RoB 2 tool was supported by the MRC Network of Hubs for Trials Methodology Research (MR/L004933/2- N61), with the support of the host MRC ConDuCT-II Hub (Collaboration and innovation for Difficult and Complex randomised controlled Trials In Invasive procedures - MR/K025643/1), by MRC research grant MR/M025209/1, and by a grant from The Cochrane Collaboration.

This work is licensed under a Creative Commons Attribution-NonCommercial-NoDerivatives 4.0 International License.

Study details

**Reference**	Böhner H, Kindgen-Milles D, Grust A, Buhl R, Lillotte WC, Müller BT, Müller E, Fürst G, Sandmann W. Prophylactic nasal continuous positive airway pressure after major vascular surgery: results of a prospective randomized trial. Langenbecks Arch Surg. 2002 Apr;387(1):21-6. doi: 10.1007/s00423-002-0281-2. Epub 2002 Mar 1. PMID: 11981680.

Study design

X Individually-randomized parallel-group trial

☐ Cluster-randomized parallel-group trial

☐ Individually randomized cross-over (or other matched) trial

For the purposes of this assessment, the interventions being compared are defined as

Experimental:	CPAP	Comparator:	COT

**Specify which outcome is being assessed for risk of bias**	Re-intubation
**Specify the numerical result being assessed.** In case of multiple alternative analyses being presented, specify the numeric result (e.g. RR = 1.52 (95% CI 0.83 to 2.77) and/or a reference (e.g. to a table, figure or paragraph) that uniquely defines the result being assessed.	5/105 patients in the COT group vs. 1/99 patients in the CPAP group (p = 0.213)

Is the review team's aim for this result…?

x to assess the effect of *assignment to intervention* (the 'intention-to-treat' effect)

☐ to assess the effect of *adhering to intervention* (the 'per-protocol' effect)

If the aim is to assess the effect of *adhering to intervention*, select the deviations from intended intervention that should be addressed (at least one must be checked):

☐ occurrence of non-protocol interventions
☐ failures in implementing the intervention that could have affected the outcome
☐ non-adherence to their assigned intervention by trial participants

Which of the following sources were obtained to help inform the risk-of-bias assessment? (tick as many as apply)

x Journal article(s) with results of the trial
☐ Trial protocol
☐ Statistical analysis plan (SAP)
☐ Non-commercial trial registry record (e.g. ClinicalTrials.gov record)
☐ Company-owned trial registry record (e.g. GSK Clinical Study Register record)
☐ "Grey literature" (e.g. unpublished thesis)
☐ Conference abstract(s) about the trial
☐ Regulatory document (e.g. Clinical Study Report, Drug Approval Package)
☐ Research ethics application
☐ Grant database summary (e.g. NIH RePORTER or Research Councils UK Gateway to Research)
☐ Personal communication with trialist
☐ Personal communication with the sponsor

Risk of bias assessment

Responses underlined in green are potential markers for low risk of bias, and responses in red are potential markers for a risk of bias. Where questions relate only to sign posts to other questions, no formatting is used.

Domain 1: Risk of bias arising from the randomization process

Signalling questions	Comments	Response options
**1.1 Was the allocation sequence random?**	Citation "After informed consent, patients were randomized using a random list".	PY
**1.2 Was the allocation sequence concealed until participants were enrolled and assigned to interventions?**	No information regarding allocation sequence concealment.	NI
**1.3 Did baseline differences between intervention groups suggest a problem with the randomization process?**	No differences regarding pre- and intraoperative data were reported between groups.	N
**Risk-of-bias judgement**		Some concerns
Optional: What is the predicted direction of bias arising from the randomization process?		NA / Favours experimental / Favours comparator / Towards null /Away from null / Unpredictable

Domain 2: Risk of bias due to deviations from the intended interventions (*effect of assignment to intervention*)

**Signalling questions**	**Comments**	**Response options**
**2.1. Were participants aware of their assigned intervention during the trial?**	The interventions involve the use of a specifical device for oxygenation in awake patients, so both patients and healthcare providers are likely aware of the interventions.	PY
**2.2. Were carers and people delivering the interventions aware of participants' assigned intervention during the trial?**		PY
**2.3. If Y/PY/NI to 2.1 or 2.2: Were there deviations from the intended intervention that arose because of the trial context?**	There were no reported deviations from the intended intervention.	N
**2.4 If Y/PY to 2.3: Were these deviations likely to have affected the outcome?**		NA
**2.5. If Y/PY/NI to 2.4: Were these deviations from intended intervention balanced between groups?**		NA
**2.6 Was an appropriate analysis used to estimate the effect of assignment to intervention?**	Citation: "Patients ending prophylactic nCPAP before this time were included in the study group for further analysis (intention-to-treat)."	PY
**2.7 If N/PN/NI to 2.6: Was there potential for a substantial impact (on the result) of the failure to analyse participants in the group to which they were randomized?**		NA
**Risk-of-bias judgement**		Low
Optional: What is the predicted direction of bias due to deviations from intended interventions?		NA / Favours experimental / Favours comparator / Towards null /Away from null / Unpredictable

Domain 3: Missing outcome data

Signalling questions	Comments	Response options
**3.1 Were data for this outcome available for all, or nearly all, participants randomized?**		Y
**3.2 If N/PN/NI to 3.1: Is there evidence that the result was not biased by missing outcome data?**		NA
**3.3 If N/PN to 3.2: Could missingness in the outcome depend on its true value?**		NA
**3.4 If Y/PY/NI to 3.3: Is it likely that missingness in the outcome depended on its true value?**		NA
**Risk-of-bias judgement**		Low
Optional: What is the predicted direction of bias due to missing outcome data?		NA / Favours experimental / Favours comparator / Towards null /Away from null / Unpredictable

Domain 4: Risk of bias in measurement of the outcome

Signalling questions	Comments	Response options
**4.1 Was the method of measuring the outcome inappropriate?**		N
**4.2 Could measurement or ascertainment of the outcome have differed between intervention groups?**	Although there is not explicit mention that concealment was maintained throughout outcomes evaluation and outcome assessment was performed by investigators who were not involved in patient care, it is unlikely that the measurement or ascertainment of the outcome differed between intervention groups.	N
**4.3 If N/PN/NI to 4.1 and 4.2: Were outcome assessors aware of the intervention received by study participants?**	There is not explicit mention that concealment was maintained throughout outcomes evaluation and outcome assessment was performed by investigators who were not involved in patient care.	PY
**4.4 If Y/PY/NI to 4.3: Could assessment of the outcome have been influenced by knowledge of intervention received?**	Although there is not explicit mention that concealment was maintained throughout outcomes evaluation and outcome assessment was performed by investigators who were not involved in patient care, it is unlikely that the measurement or ascertainment of the outcome differed between intervention groups, considering that re-intubation is an outcome that does not involve any judgement.	PN
**4.5 If Y/PY/NI to 4.4: Is it likely that assessment of the outcome was influenced by knowledge of intervention received?**		NA
**Risk-of-bias judgement**		Low
Optional: What is the predicted direction of bias in measurement of the outcome?		NA / Favours experimental / Favours comparator / Towards null /Away from null / Unpredictable

Domain 5: Risk of bias in selection of the reported result

Signalling questions	Comments	Response options
**5.1 Were the data that produced this result analysed in accordance with a pre-specified analysis plan that was finalized before unblinded outcome data were available for analysis?**	No information regarding trial registration and prespecified analysis plan publication.	NI
**Is the numerical result being assessed likely to have been selected, on the basis of the results, from...**		
**5.2. ... multiple eligible outcome measurements (e.g. scales, definitions, time points) within the outcome domain?**		N
**5.3 ... multiple eligible analyses of the data?**		N
**Risk-of-bias judgement**		Some concerns
Optional: What is the predicted direction of bias due to selection of the reported result?		NA / Favours experimental / Favours comparator / Towards null /Away from null / Unpredictable

Overall risk of bias

**Risk-of-bias judgement**		Some concerns
Optional: What is the overall predicted direction of bias for this outcome?		NA / Favours experimental / Favours comparator / Towards null /Away from null / Unpredictable

This work is licensed under a Creative Commons Attribution-NonCommercial-NoDerivatives 4.0 International License.

Revised Cochrane risk-of-bias tool for randomized trials (RoB 2)
TEMPLATE FOR COMPLETION

Edited by Julian PT Higgins, Jelena Savović, Matthew J Page, Jonathan AC Sterne
on behalf of the RoB2 Development Group

Version of 22 August 2019

The development of the RoB 2 tool was supported by the MRC Network of Hubs for Trials Methodology Research (MR/L004933/2- N61), with the support of the host MRC ConDuCT-II Hub (Collaboration and innovation for Difficult and Complex randomised controlled Trials In Invasive procedures - MR/K025643/1), by MRC research grant MR/M025209/1, and by a grant from The Cochrane Collaboration.

This work is licensed under a Creative Commons Attribution-NonCommercial-NoDerivatives 4.0 International License.

Study details

**Reference**	Burra V, Putta G, Prasad SR, Manjunath V. A prospective study on use of thrive (transnasal humidified rapid insufflation ventilatory exchange) versus conventional nasal oxygenation following extubation of adult cardiac surgical patients. Ann Card Anaesth. 2021 Jul-Sep;24(3):353-357. doi: 10.4103/aca.ACA_16_20. PMID: 34269267; PMCID: PMC8404600.

Study design

X Individually-randomized parallel-group trial
☐ Cluster-randomized parallel-group trial
☐ Individually randomized cross-over (or other matched) trial

For the purposes of this assessment, the interventions being compared are defined as

Experimental: HFNO Comparator: COT

**Specify which outcome is being assessed for risk of bias**	Re-intubation
**Specify the numerical result being assessed.** In case of multiple alternative analyses being presented, specify the numeric result (e.g. RR = 1.52 (95% CI 0.83 to 2.77) and/or a reference (e.g. to a table, figure or paragraph) that uniquely defines the result being assessed.	0/30 patients in both groups

Is the review team's aim for this result…?

X to assess the effect of *assignment to intervention* (the 'intention-to-treat' effect)
☐ to assess the effect of *adhering to intervention* (the 'per-protocol' effect)

If the aim is to assess the effect of *adhering to intervention*, select the deviations from intended intervention that should be addressed (at least one must be checked):

☐ occurrence of non-protocol interventions
☐ failures in implementing the intervention that could have affected the outcome
☐ non-adherence to their assigned intervention by trial participants

Which of the following sources were <u>obtained</u> to help inform the risk-of-bias assessment? (tick as many as apply)

- X Journal article(s) with results of the trial
- ☐ Trial protocol
- ☐ Statistical analysis plan (SAP)
- ☐ Non-commercial trial registry record (e.g. ClinicalTrials.gov record)
- ☐ Company-owned trial registry record (e.g. GSK Clinical Study Register record)
- ☐ "Grey literature" (e.g. unpublished thesis)
- ☐ Conference abstract(s) about the trial
- ☐ Regulatory document (e.g. Clinical Study Report, Drug Approval Package)
- ☐ Research ethics application
- ☐ Grant database summary (e.g. NIH RePORTER or Research Councils UK Gateway to Research)
- ☐ Personal communication with trialist
- ☐ Personal communication with the sponsor

Risk of bias assessment

Responses underlined in green are potential markers for low risk of bias, and responses in red are potential markers for a risk of bias. Where questions relate only to sign posts to other questions, no formatting is used.

Domain 1: Risk of bias arising from the randomization process

Signalling questions	Comments	Response options
**1.1 Was the allocation sequence random?**	Citation: "Patients were randomized into group A and group B based on a computer-generated random sequence of numbers and allocation concealment was done using sequentially numbered opaque sealed envelopes."	Y
**1.2 Was the allocation sequence concealed until participants were enrolled and assigned to interventions?**	There is no evidence that the allocation sequence was not concealed until participants were enrolled and assigned to interventions.	PY
**1.3 Did baseline differences between intervention groups suggest a problem with the randomization process?**	Citation: "Demographic data and type of surgery were comparable between two groups".	N
**Risk-of-bias judgement**		Low
Optional: What is the predicted direction of bias arising from the randomization process?		NA / Favours experimental / Favours comparator / Towards null /Away from null / Unpredictable

Domain 2: Risk of bias due to deviations from the intended interventions (*effect of assignment to intervention*)

Signalling questions	Comments	Response options
**2.1. Were participants aware of their assigned intervention during the trial?**	The intervention involves the use of a specifical device for oxygenation in awake patients. Therefore, they were likely to be aware of their assigned intervention.	Y
**2.2. Were carers and people delivering the interventions aware of participants' assigned intervention during the trial?**		Y
**2.3. If Y/PY/NI to 2.1 or 2.2: Were there deviations from the intended intervention that arose because of the trial context?**	There were no reported deviations from the intended intervention.	N
**2.4 If Y/PY to 2.3: Were these deviations likely to have affected the outcome?**		NA
**2.5. If Y/PY/NI to 2.4: Were these deviations from intended intervention balanced between groups?**		NA
**2.6 Was an appropriate analysis used to estimate the effect of assignment to intervention?**	No information is provided regarding the analysis.	NI
**2.7 If N/PN/NI to 2.6: Was there potential for a substantial impact (on the result) of the failure to analyse participants in the group to which they were randomized?**	The failure to analyse participants in the group to which they were randomized is not likely to impact on the study results, considering that no patients were excluded after randomization.	PN
**Risk-of-bias judgement**		Some concerns
Optional: What is the predicted direction of bias due to deviations from intended interventions?		NA / Favours experimental / Favours comparator / Towards null /Away from null / Unpredictable

Domain 3: Missing outcome data

Signalling questions	Comments	Response options
**3.1 Were data for this outcome available for all, or nearly all, participants randomized?**	Data for the outcome were available for all patients enrolled.	Y
**3.2 If N/PN/NI to 3.1: Is there evidence that the result was not biased by missing outcome data?**		NA
**3.3 If N/PN to 3.2: Could missingness in the outcome depend on its true value?**		NA
**3.4 If Y/PY/NI to 3.3: Is it likely that missingness in the outcome depended on its true value?**		NA
**Risk-of-bias judgement**		Low
Optional: What is the predicted direction of bias due to missing outcome data?		NA / Favours experimental / Favours comparator / Towards null /Away from null / Unpredictable

Domain 4: Risk of bias in measurement of the outcome

Signalling questions	Comments	Response options
**4.1 Was the method of measuring the outcome inappropriate?**		N
**4.2 Could measurement or ascertainment of the outcome have differed between intervention groups?**	Although there is no mention that outcome assessors were blinded to the intervention received by study participants, it is not likely that the ascertainment of the outcome differed between intervention groups because re-intubation is an objective outcome.	PN
**4.3 If N/PN/NI to 4.1 and 4.2: Were outcome assessors aware of the intervention received by study participants?**	There is no mention that outcome assessors were blinded to the intervention received by study participants	PY
**4.4 If Y/PY/NI to 4.3: Could assessment of the outcome have been influenced by knowledge of intervention received?**	Reintubation is an outcome that does not involve any judgement. Therefore, it is not likely that the ascertainment of the outcome differed between intervention groups.	PN
**4.5 If Y/PY/NI to 4.4: Is it likely that assessment of the outcome was influenced by knowledge of intervention received?**		NA
**Risk-of-bias judgement**		Low
Optional: What is the predicted direction of bias in measurement of the outcome?		NA / Favours experimental / Favours comparator / Towards null /Away from null / Unpredictable

Domain 5: Risk of bias in selection of the reported result

Signalling questions	Comments	Response options
**5.1 Were the data that produced this result analysed in accordance with a pre-specified analysis plan that was finalized before unblinded outcome data were available for analysis?**	There is no mention of any trial registration or prespecified statistical analysis plan.	NI
**Is the numerical result being assessed likely to have been selected, on the basis of the results, from...**		
**5.2. ... multiple eligible outcome measurements (e.g. scales, definitions, time points) within the outcome domain?**		N
**5.3 ... multiple eligible analyses of the data?**		N
**Risk-of-bias judgement**		Some concerns
Optional: What is the predicted direction of bias due to selection of the reported result?		NA / Favours experimental / Favours comparator / Towards null /Away from null / Unpredictable

Overall risk of bias

**Risk-of-bias judgement**		Some concerns
Optional: What is the overall predicted direction of bias for this outcome?		NA / Favours experimental / Favours comparator / Towards null /Away from null / Unpredictable

This work is licensed under a Creative Commons Attribution-NonCommercial-NoDerivatives 4.0 International License.

Revised Cochrane risk-of-bias tool for randomized trials (RoB 2)
TEMPLATE FOR COMPLETION

Edited by Julian PT Higgins, Jelena Savović, Matthew J Page, Jonathan AC Sterne
on behalf of the RoB2 Development Group

Version of 22 August 2019

The development of the RoB 2 tool was supported by the MRC Network of Hubs for Trials Methodology Research (MR/L004933/2- N61), with the support of the host MRC ConDuCT-II Hub (Collaboration and innovation for Difficult and Complex randomised controlled Trials In Invasive procedures - MR/K025643/1), by MRC research grant MR/M025209/1, and by a grant from The Cochrane Collaboration.

This work is licensed under a Creative Commons Attribution-NonCommercial-NoDerivatives 4.0 International License.

Study details

**Reference**	Corley A, Bull T, Spooner AJ, Barnett AG, Fraser JF. Direct extubation onto high-flow nasal cannulae post-cardiac surgery versus standard treatment in patients with a BMI ≥30: a randomised controlled trial. Intensive Care Med. 2015 May;41(5):887-94. doi: 10.1007/s00134-015-3765-6. Epub 2015 Apr 8. PMID: 25851385.

Study design

X Individually-randomized parallel-group trial

☐ Cluster-randomized parallel-group trial

☐ Individually randomized cross-over (or other matched) trial

For the purposes of this assessment, the interventions being compared are defined as

Experimental: HFNO   Comparator: COT

**Specify which outcome is being assessed for risk of bias**	Re-intubation
**Specify the numerical result being assessed.** In case of multiple alternative analyses being presented, specify the numeric result (e.g. RR = 1.52 (95% CI 0.83 to 2.77) and/or a reference (e.g. to a table, figure or paragraph) that uniquely defines the result being assessed.	1/74 patients in the COT group vs. 1/81 patient in the HFNO group

Is the review team's aim for this result…?

X to assess the effect of *assignment to intervention* (the 'intention-to-treat' effect)

☐ to assess the effect of *adhering to intervention* (the 'per-protocol' effect)

If the aim is to assess the effect of *adhering to intervention*, select the deviations from intended intervention that should be addressed (at least one must be checked):

- ☐ occurrence of non-protocol interventions
- ☐ failures in implementing the intervention that could have affected the outcome
- ☐ non-adherence to their assigned intervention by trial participants

Which of the following sources were <u>obtained</u> to help inform the risk-of-bias assessment? (tick as many as apply)

- X Journal article(s) with results of the trial
- ☐ Trial protocol
- ☐ Statistical analysis plan (SAP)
- ☐ Non-commercial trial registry record (e.g. ClinicalTrials.gov record)
- ☐ Company-owned trial registry record (e.g. GSK Clinical Study Register record)
- ☐ "Grey literature" (e.g. unpublished thesis)
- ☐ Conference abstract(s) about the trial
- ☐ Regulatory document (e.g. Clinical Study Report, Drug Approval Package)
- ☐ Research ethics application
- ☐ Grant database summary (e.g. NIH RePORTER or Research Councils UK Gateway to Research)
- ☐ Personal communication with trialist
- ☐ Personal communication with the sponsor

Risk of bias assessment

Responses underlined in green are potential markers for low risk of bias, and responses in red are potential markers for a risk of bias. Where questions relate only to sign posts to other questions, no formatting is used.

Domain 1: Risk of bias arising from the randomization process

Signalling questions	Comments	Response options
**1.1 Was the allocation sequence random?**	Citation: "Patients were randomised to receive standard oxygen therapy or HFNC post-extubation. Randomisation was via a computerised random numbers table in blocks of eight. Allocation concealment was maintained through numbered opaque envelopes".	Y
**1.2 Was the allocation sequence concealed until participants were enrolled and assigned to interventions?**		Y
**1.3 Did baseline differences between intervention groups suggest a problem with the randomization process?**	No relevant baseline differences between intervention groups were reported.	N
**Risk-of-bias judgement**		Low
Optional: What is the predicted direction of bias arising from the randomization process?		NA / Favours experimental / Favours comparator / Towards null /Away from null / Unpredictable

Domain 2: Risk of bias due to deviations from the intended interventions (*effect of assignment to intervention*)

**Signalling questions**	**Comments**	**Response options**
**2.1. Were participants aware of their assigned intervention during the trial?**	Citation: "It was not possible to blind the patients, bedside staff or research nurse to treatment allocation".	Y
**2.2. Were carers and people delivering the interventions aware of participants' assigned intervention during the trial?**		Y
**2.3. If Y/PY/NI to 2.1 or 2.2: Were there deviations from the intended intervention that arose because of the trial context?**	No deviations from the intended intervention arose because of the trial context	N
**2.4 If Y/PY to 2.3: Were these deviations likely to have affected the outcome?**		NA
**2.5. If Y/PY/NI to 2.4: Were these deviations from intended intervention balanced between groups?**		NA
**2.6 Was an appropriate analysis used to estimate the effect of assignment to intervention?**	Citation: "The analysis was performed using intention-to-treat".	Y
**2.7 If N/PN/NI to 2.6: Was there potential for a substantial impact (on the result) of the failure to analyse participants in the group to which they were randomized?**		NA
**Risk-of-bias judgement**		Low
Optional: What is the predicted direction of bias due to deviations from intended interventions?		NA / Favours experimental / Favours comparator / Towards null /Away from null / Unpredictable

Domain 3: Missing outcome data

Signalling questions	Comments	Response options
**3.1 Were data for this outcome available for all, or nearly all, participants randomized?**	Although there was incomplete data for the second and third time periods due to patients' respiratory status stabilising and/ or being transferred to the ward, these missing data did not concern the re-intubation outcome.	Y
**3.2 If N/PN/NI to 3.1: Is there evidence that the result was not biased by missing outcome data?**		NA
**3.3 If N/PN to 3.2: Could missingness in the outcome depend on its true value?**		NA
**3.4 If Y/PY/NI to 3.3: Is it likely that missingness in the outcome depended on its true value?**		NA
**Risk-of-bias judgement**		Low
Optional: What is the predicted direction of bias due to missing outcome data?		NA / Favours experimental / Favours comparator / Towards null /Away from null / Unpredictable

Domain 4: Risk of bias in measurement of the outcome

**Signalling questions**	**Comments**	**Response options**
**4.1 Was the method of measuring the outcome inappropriate?**		N
**4.2 Could measurement or ascertainment of the outcome have differed between intervention groups?**	Citations: "…it was not possible to blind staff and participants to treatment allocation; however, for the primary outcome of atelectasis, assessors were blinded to treatment allocation".	PN
**4.3 If N/PN/NI to 4.1 and 4.2: Were outcome assessors aware of the intervention received by study participants?**	Citations: "…it was not possible to blind staff and participants to treatment allocation; however, for the primary outcome of atelectasis, assessors were blinded to treatment allocation".	N
**4.4 If Y/PY/NI to 4.3: Could assessment of the outcome have been influenced by knowledge of intervention received?**		NA
**4.5 If Y/PY/NI to 4.4: Is it likely that assessment of the outcome was influenced by knowledge of intervention received?**		NA
**Risk-of-bias judgement**		Low
Optional: What is the predicted direction of bias in measurement of the outcome?		NA / Favours experimental / Favours comparator / Towards null /Away from null / Unpredictable

Domain 5: Risk of bias in selection of the reported result

Signalling questions	Comments	Response options
**5.1 Were the data that produced this result analysed in accordance with a pre-specified analysis plan that was finalized before unblinded outcome data were available for analysis?**	Adherence to the registered protocol (ACTRN12610000942055)	Y
**Is the numerical result being assessed likely to have been selected, on the basis of the results, from...**		
**5.2. ... multiple eligible outcome measurements (e.g. scales, definitions, time points) within the outcome domain?**		N
**5.3 ... multiple eligible analyses of the data?**		N
**Risk-of-bias judgement**		Low
Optional: What is the predicted direction of bias due to selection of the reported result?		NA / Favours experimental / Favours comparator / Towards null /Away from null / Unpredictable

Overall risk of bias

**Risk-of-bias judgement**		Low
Optional: What is the overall predicted direction of bias for this outcome?		NA / Favours experimental / Favours comparator / Towards null /Away from null / Unpredictable

This work is licensed under a Creative Commons Attribution-NonCommercial-NoDerivatives 4.0 International License.

Revised Cochrane risk-of-bias tool for randomized trials (RoB 2)
TEMPLATE FOR COMPLETION

Edited by Julian PT Higgins, Jelena Savović, Matthew J Page, Jonathan AC Sterne
on behalf of the RoB2 Development Group

Version of 22 August 2019

The development of the RoB 2 tool was supported by the MRC Network of Hubs for Trials Methodology Research (MR/L004933/2- N61), with the support of the host MRC ConDuCT-II Hub (Collaboration and innovation for Difficult and Complex randomised controlled Trials In Invasive procedures - MR/K025643/1), by MRC research grant MR/M025209/1, and by a grant from The Cochrane Collaboration.

This work is licensed under a Creative Commons Attribution-NonCommercial-NoDerivatives 4.0 International License.

Study details

Reference

Futier E, Paugam-Burtz C, Godet T, Khoy-Ear L, Rozencwajg S, Delay JM, Verzilli D, Dupuis J, Chanques G, Bazin JE, Constantin JM, Pereira B, Jaber S; OPERA study investigators. Effect of early postextubation high-flow nasal cannula vs conventional oxygen therapy on hypoxaemia in patients after major abdominal surgery: a French multicentre randomised controlled trial (OPERA). Intensive Care Med. 2016 Dec;42(12):1888-1898. doi: 10.1007/s00134-016-4594-y. Epub 2016 Oct 22. PMID: 27771739.

Study design

- X Individually-randomized parallel-group trial
- ☐ Cluster-randomized parallel-group trial
- ☐ Individually randomized cross-over (or other matched) trial

For the purposes of this assessment, the interventions being compared are defined as

Experimental: HFNO Comparator: COT

Specify which outcome is being assessed for risk of bias: Re-intubation

Specify the numerical result being assessed. In case of multiple alternative analyses being presented, specify the numeric result (e.g. RR = 1.52 (95% CI 0.83 to 2.77) and/or a reference (e.g. to a table, figure or paragraph) that uniquely defines the result being assessed.

4/112 patients in the COT group vs. 7/108 patients in the HFNO group

Is the review team's aim for this result…?

- X to assess the effect of *assignment to intervention* (the 'intention-to-treat' effect)
- ☐ to assess the effect of *adhering to intervention* (the 'per-protocol' effect)

If the aim is to assess the effect of *adhering to intervention*, select the deviations from intended intervention that should be addressed (at least one must be checked):

- ☐ occurrence of non-protocol interventions
- ☐ failures in implementing the intervention that could have affected the outcome
- ☐ non-adherence to their assigned intervention by trial participants

Which of the following sources were <u>obtained</u> to help inform the risk-of-bias assessment? (tick as many as apply)

X Journal article(s) with results of the trial
X Trial protocol
☐ Statistical analysis plan (SAP)
X Non-commercial trial registry record (e.g. ClinicalTrials.gov record)
☐ Company-owned trial registry record (e.g. GSK Clinical Study Register record)
☐ "Grey literature" (e.g. unpublished thesis)
☐ Conference abstract(s) about the trial
☐ Regulatory document (e.g. Clinical Study Report, Drug Approval Package)
☐ Research ethics application
☐ Grant database summary (e.g. NIH RePORTER or Research Councils UK Gateway to Research)
☐ Personal communication with trialist
☐ Personal communication with the sponsor

Risk of bias assessment

Responses underlined in green are potential markers for low risk of bias, and responses in red are potential markers for a risk of bias. Where questions relate only to sign posts to other questions, no formatting is used.

Domain 1: Risk of bias arising from the randomization process

Signalling questions	Comments	Response options
**1.1 Was the allocation sequence random?**	Citation: "Randomisation was performed (in a 1:1 ratio) with the use of a computer-generated assignment sequence and a centralised telephone system accessible round the clock to receive either HFNC or standard oxygen therapy immediately after tracheal extubation. Randomisation was stratified according to study centre and the planned use or non-use of postoperative epidural analgesia, which is a factor that may influence outcomes".	Y
**1.2 Was the allocation sequence concealed until participants were enrolled and assigned to interventions?**	Citation: "Although the individual assignments of patients could not be masked to staff members who collected data during surgery and in the post-anaesthesia care unit, treatment allocation was concealed to outcome assessors throughout the study".	Y
**1.3 Did baseline differences between intervention groups suggest a problem with the randomization process?**	Citation: "The two groups of patients had similar baseline characteristics".	N
**Risk-of-bias judgement**		Low
Optional: What is the predicted direction of bias arising from the randomization process?		NA / Favours experimental / Favours comparator / Towards null /Away from null / Unpredictable

Domain 2: Risk of bias due to deviations from the intended interventions (*effect of assignment to intervention*)

Signalling questions	Comments	Response options
**2.1. Were participants aware of their assigned intervention during the trial?**	Citation: "Although the individual assignments of patients could not be masked to staff members who collected data during surgery and in the post-anaesthesia care unit, treatment allocation was concealed to outcome assessors throughout the study"	Y
**2.2. Were carers and people delivering the interventions aware of participants' assigned intervention during the trial?**		Y
**2.3. If Y/PY/NI to 2.1 or 2.2: Were there deviations from the intended intervention that arose because of the trial context?**	No deviations from the intended interventions are reported.	PN
**2.4 If Y/PY to 2.3: Were these deviations likely to have affected the outcome?**		NA
**2.5. If Y/PY/NI to 2.4: Were these deviations from intended intervention balanced between groups?**		NA
**2.6 Was an appropriate analysis used to estimate the effect of assignment to intervention?**	Citation: "All patients were included in the final intention-to-treat analysis".	Y
**2.7 If N/PN/NI to 2.6: Was there potential for a substantial impact (on the result) of the failure to analyse participants in the group to which they were randomized?**		NA
**Risk-of-bias judgement**		Low
Optional: What is the predicted direction of bias due to deviations from intended interventions?		NA / Favours experimental / Favours comparator / Towards null /Away from null / Unpredictable

Domain 3: Missing outcome data

Signalling questions	Comments	Response options
**3.1 Were data for this outcome available for all, or nearly all, participants randomized?**		Y
**3.2 If N/PN/NI to 3.1: Is there evidence that the result was not biased by missing outcome data?**		NA
**3.3 If N/PN to 3.2: Could missingness in the outcome depend on its true value?**		NA
**3.4 If Y/PY/NI to 3.3: Is it likely that missingness in the outcome depended on its true value?**		NA
**Risk-of-bias judgement**		Low
Optional: What is the predicted direction of bias due to missing outcome data?		NA / Favours experimental / Favours comparator / Towards null /Away from null / Unpredictable

Domain 4: Risk of bias in measurement of the outcome

Signalling questions	Comments	Response options
**4.1 Was the method of measuring the outcome inappropriate?**		N
**4.2 Could measurement or ascertainment of the outcome have differed between intervention groups?**	Citation: "A trained research coordinator, blinded to the randomised intervention, was responsible for centralisation of data from all sites and recording them onto the electronic database. Although the individual assignments of patients could not be masked to staff members who collected data during surgery and in the post-anaesthesia care unit, treatment allocation was concealed to outcome assessors throughout the study".	PN
**4.3 If N/PN/NI to 4.1 and 4.2: Were outcome assessors aware of the intervention received by study participants?**	Citation: "A trained research coordinator, blinded to the randomised intervention, was responsible for centralisation of data from all sites and recording them onto the electronic database. Although the individual assignments of patients could not be masked to staff members who collected data during surgery and in the post-anaesthesia care unit, treatment allocation was concealed to outcome assessors throughout the study".	PN
**4.4 If Y/PY/NI to 4.3: Could assessment of the outcome have been influenced by knowledge of intervention received?**		NA
**4.5 If Y/PY/NI to 4.4: Is it likely that assessment of the outcome was influenced by knowledge of intervention received?**		NA
**Risk-of-bias judgement**		Low
Optional: What is the predicted direction of bias in measurement of the outcome?		NA / Favours experimental / Favours comparator / Towards null /Away from null / Unpredictable

Domain 5: Risk of bias in selection of the reported result

Signalling questions	Comments	Response options
**5.1 Were the data that produced this result analysed in accordance with a pre-specified analysis plan that was finalized before unblinded outcome data were available for analysis?**	Adherence to clinical trial registration and published protocol.	Y
**Is the numerical result being assessed likely to have been selected, on the basis of the results, from...**		
**5.2. ... multiple eligible outcome measurements (e.g. scales, definitions, time points) within the outcome domain?**		N
**5.3 ... multiple eligible analyses of the data?**		N
**Risk-of-bias judgement**		Low
Optional: What is the predicted direction of bias due to selection of the reported result?		NA / Favours experimental / Favours comparator / Towards null /Away from null / Unpredictable

Overall risk of bias

**Risk-of-bias judgement**		Low
Optional: What is the overall predicted direction of bias for this outcome?		NA / Favours experimental / Favours comparator / Towards null /Away from null / Unpredictable

This work is licensed under a Creative Commons Attribution-NonCommercial-NoDerivatives 4.0 International License.

Revised Cochrane risk-of-bias tool for randomized trials (RoB 2)
TEMPLATE FOR COMPLETION

Edited by Julian PT Higgins, Jelena Savović, Matthew J Page, Jonathan AC Sterne
on behalf of the RoB2 Development Group

Version of 22 August 2019

The development of the RoB 2 tool was supported by the MRC Network of Hubs for Trials Methodology Research (MR/L004933/2- N61), with the support of the host MRC ConDuCT-II Hub (Collaboration and innovation for Difficult and Complex randomised controlled Trials In Invasive procedures - MR/K025643/1), by MRC research grant MR/M025209/1, and by a grant from The Cochrane Collaboration.

This work is licensed under a Creative Commons Attribution-NonCommercial-NoDerivatives 4.0 International License.

Study details

**Reference**	Gupta, Sampa Datta; Pareek, Anjana; Ghose, Tapas; Sarkar, Ujjal Kumar; Mukherjee, Sudakshina; Goswami, Anupam; Sarbapalli, Debabrata; Pal, Samarendra. Management of Postoperative Hypoxaemia in Patients Following Upper Abdominal Laparoscopic Surgery. A Comparative Study. Indian Journal of Anaesthesia 52(2):185-190, Mar–Apr 2008.

Study design

X Individually-randomized parallel-group trial

☐ Cluster-randomized parallel-group trial

☐ Individually randomized cross-over (or other matched) trial

For the purposes of this assessment, the interventions being compared are defined as

Experimental:	CPAP	Comparator:	COT

**Specify which outcome is being assessed for risk of bias**	Re-intubation
**Specify the numerical result being assessed.** In case of multiple alternative analyses being presented, specify the numeric result (e.g. RR = 1.52 (95% CI 0.83 to 2.77) and/or a reference (e.g. to a table, figure or paragraph) that uniquely defines the result being assessed.	2/20 patients in the CPAP group vs. 0/20 patients in the COT group ($p < 0.15$)

Is the review team's aim for this result…?

× to assess the effect of *assignment to intervention* (the 'intention-to-treat' effect)

☐ to assess the effect of *adhering to intervention* (the 'per-protocol' effect)

If the aim is to assess the effect of *adhering to intervention*, select the deviations from intended intervention that should be addressed (at least one must be checked):

☐ occurrence of non-protocol interventions
☐ failures in implementing the intervention that could have affected the outcome
☐ non-adherence to their assigned intervention by trial participants

Which of the following sources were <u>obtained</u> to help inform the risk-of-bias assessment? (tick as many as apply)

× Journal article(s) with results of the trial
☐ Trial protocol
☐ Statistical analysis plan (SAP)
☐ Non-commercial trial registry record (e.g. ClinicalTrials.gov record)
☐ Company-owned trial registry record (e.g. GSK Clinical Study Register record)
☐ “Grey literature” (e.g. unpublished thesis)
☐ Conference abstract(s) about the trial
☐ Regulatory document (e.g. Clinical Study Report, Drug Approval Package)
☐ Research ethics application
☐ Grant database summary (e.g. NIH RePORTER or Research Councils UK Gateway to Research)
☐ Personal communication with trialist
☐ Personal communication with the sponsor

Risk of bias assessment

Responses underlined in green are potential markers for low risk of bias, and responses in red are potential markers for a risk of bias. Where questions relate only to sign posts to other questions, no formatting is used.

Domain 1: Risk of bias arising from the randomization process

Signalling questions	Comments	Response options
**1.1 Was the allocation sequence random?**	Citation: "Forty patients with PaO2 / FiO2 ratio between 250 and 300 were randomly allocated into two groups through computer-generated random number concealed in sealed envelopes to receive oxygen supplementation delivered either by a venturi mask or a CPAP system".	Y
**1.2 Was the allocation sequence concealed until participants were enrolled and assigned to interventions?**	There is no evidence that the allocation sequence was not concealed until participants were enrolled and assigned to interventions.	PY
**1.3 Did baseline differences between intervention groups suggest a problem with the randomization process?**	Citations: "the two groups are comparable in terms of demographic profiles and duration of general anaesthesia"; "there is no significant difference between the two groups of patients at the time of initiation of therapy	N
**Risk-of-bias judgement**		Low
Optional: What is the predicted direction of bias arising from the randomization process?		NA / Favours experimental / Favours comparator / Towards null /Away from null / Unpredictable

Domain 2: Risk of bias due to deviations from the intended interventions (*effect of assignment to intervention*)

Signalling questions	Comments	Response options
**2.1. Were participants aware of their assigned intervention during the trial?**	The intervention involves the use of a specifical device for oxygenation in awake patients. Therefore, they were likely to be aware of their assigned intervention.	Y
**2.2. Were carers and people delivering the interventions aware of participants' assigned intervention during the trial?**		Y
**2.3. If Y/PY/NI to 2.1 or 2.2: Were there deviations from the intended intervention that arose because of the trial context?**	There were no reported deviations from the intended intervention.	PN
**2.4 If Y/PY to 2.3: Were these deviations likely to have affected the outcome?**		NA
**2.5. If Y/PY/NI to 2.4: Were these deviations from intended intervention balanced between groups?**		NA
**2.6 Was an appropriate analysis used to estimate the effect of assignment to intervention?**	No information is provided regarding the analysis.	NI
**2.7 If N/PN/NI to 2.6: Was there potential for a substantial impact (on the result) of the failure to analyse participants in the group to which they were randomized?**	The failure to analyse participants in the group to which they were randomized is not likely to impact on the study results, considering that no patients were excluded after randomization.	PN
**Risk-of-bias judgement**		Some concerns
Optional: What is the predicted direction of bias due to deviations from intended interventions?		NA / Favours experimental / Favours comparator / Towards null /Away from null / Unpredictable

Domain 3: Missing outcome data

Signalling questions	Comments	Response options
**3.1 Were data for this outcome available for all, or nearly all, participants randomized?**	Data for the outcome were available for all patients enrolled.	Y
**3.2 If N/PN/NI to 3.1: Is there evidence that the result was not biased by missing outcome data?**		NA
**3.3 If N/PN to 3.2: Could missingness in the outcome depend on its true value?**		NA
**3.4 If Y/PY/NI to 3.3: Is it likely that missingness in the outcome depended on its true value?**		NA
**Risk-of-bias judgement**		Low
Optional: What is the predicted direction of bias due to missing outcome data?		NA / Favours experimental / Favours comparator / Towards null /Away from null / Unpredictable

Domain 4: Risk of bias in measurement of the outcome

Signalling questions	Comments	Response options
**4.1 Was the method of measuring the outcome inappropriate?**		N
**4.2 Could measurement or ascertainment of the outcome have differed between intervention groups?**	Although there is no mention that outcome assessors were blinded to the intervention received by study participants, it is not likely that the ascertainment of the outcome differed between intervention groups because re-intubation is an objective outcome.	PN
**4.3 If N/PN/NI to 4.1 and 4.2: Were outcome assessors aware of the intervention received by study participants?**	There is no mention that outcome assessors were blinded to the intervention received by study participants	PY
**4.4 If Y/PY/NI to 4.3: Could assessment of the outcome have been influenced by knowledge of intervention received?**	Re-intubation is an outcome that does not involve any judgement. Therefore, it is not likely that the ascertainment of the outcome differed between intervention groups.	PN
**4.5 If Y/PY/NI to 4.4: Is it likely that assessment of the outcome was influenced by knowledge of intervention received?**		NA
**Risk-of-bias judgement**		Low
Optional: What is the predicted direction of bias in measurement of the outcome?		NA / Favours experimental / Favours comparator / Towards null /Away from null / Unpredictable

Domain 5: Risk of bias in selection of the reported result

Signalling questions	Comments	Response options
**5.1 Were the data that produced this result analysed in accordance with a pre-specified analysis plan that was finalized before unblinded outcome data were available for analysis?**	There is no mention of any trial registration or prespecified statistical analysis plan.	NI
**Is the numerical result being assessed likely to have been selected, on the basis of the results, from...**		
**5.2. ... multiple eligible outcome measurements (e.g. scales, definitions, time points) within the outcome domain?**		N
**5.3 ... multiple eligible analyses of the data?**		N
**Risk-of-bias judgement**		Some concerns
Optional: What is the predicted direction of bias due to selection of the reported result?		NA / Favours experimental / Favours comparator / Towards null /Away from null / Unpredictable

Overall risk of bias

**Risk-of-bias judgement**		Some concerns
Optional: What is the overall predicted direction of bias for this outcome?		NA / Favours experimental / Favours comparator / Towards null /Away from null / Unpredictable

This work is licensed under a Creative Commons Attribution-NonCommercial-NoDerivatives 4.0 International License.

Revised Cochrane risk-of-bias tool for randomized trials (RoB 2)
TEMPLATE FOR COMPLETION

Edited by Julian PT Higgins, Jelena Savović, Matthew J Page, Jonathan AC Sterne
on behalf of the RoB2 Development Group

Version of 22 August 2019

The development of the RoB 2 tool was supported by the MRC Network of Hubs for Trials Methodology Research (MR/L004933/2- N61), with the support of the host MRC ConDuCT-II Hub (Collaboration and innovation for Difficult and Complex randomised controlled Trials In Invasive procedures - MR/K025643/1), by MRC research grant MR/M025209/1, and by a grant from The Cochrane Collaboration.

This work is licensed under a Creative Commons Attribution-NonCommercial-NoDerivatives 4.0 International License.

Study details

**Reference**	Hewidy AA, Suliman LA, El Hefnawy E, Hassan AA. Immediate continuous positive airway pressure (CPAP) therapy after sleeve gastrectomy. Egyptian Journal of Chest Diseases and Tuberculosis. 2016;65(3):701-706.

Study design

X Individually-randomized parallel-group trial

☐ Cluster-randomized parallel-group trial

☐ Individually randomized cross-over (or other matched) trial

For the purposes of this assessment, the interventions being compared are defined as

Experimental:	CPAP	Comparator:	COT

**Specify which outcome is being assessed for risk of bias**	Re-intubation
**Specify the numerical result being assessed.** In case of multiple alternative analyses being presented, specify the numeric result (e.g. RR = 1.52 (95% CI 0.83 to 2.77) and/or a reference (e.g. to a table, figure or paragraph) that uniquely defines the result being assessed.	1/22 patients in the COT group vs. 0/24 patients in the CPAP group (p-value not significant)

Is the review team's aim for this result…?

x to assess the effect of *assignment to intervention* (the 'intention-to-treat' effect)

☐ to assess the effect of *adhering to intervention* (the 'per-protocol' effect)

If the aim is to assess the effect of *adhering to intervention*, select the deviations from intended intervention that should be addressed (at least one must be checked):

☐ occurrence of non-protocol interventions
☐ failures in implementing the intervention that could have affected the outcome
☐ non-adherence to their assigned intervention by trial participants

Which of the following sources were obtained to help inform the risk-of-bias assessment? (tick as many as apply)

x Journal article(s) with results of the trial
☐ Trial protocol
☐ Statistical analysis plan (SAP)
☐ Non-commercial trial registry record (e.g. ClinicalTrials.gov record)
☐ Company-owned trial registry record (e.g. GSK Clinical Study Register record)
☐ “Grey literature” (e.g. unpublished thesis)
☐ Conference abstract(s) about the trial
☐ Regulatory document (e.g. Clinical Study Report, Drug Approval Package)
☐ Research ethics application
☐ Grant database summary (e.g. NIH RePORTER or Research Councils UK Gateway to Research)
☐ Personal communication with trialist
☐ Personal communication with the sponsor

Risk of bias assessment

Responses underlined in green are potential markers for low risk of bias, and responses in red are potential markers for a risk of bias. Where questions relate only to sign posts to other questions, no formatting is used.

Domain 1: Risk of bias arising from the randomization process

Signalling questions	Comments	Response options
**1.1 Was the allocation sequence random?**	Citation "Patients were randomly divided into two groups".	PY
**1.2 Was the allocation sequence concealed until participants were enrolled and assigned to interventions?**	Citation: "The patients were randomly selected using the closed envelop method".	PY
**1.3 Did baseline differences between intervention groups suggest a problem with the randomization process?**	No differences regarding demographic data, anthropometric measurements, and preoperative pulmonary function tests were reported between groups.	N
**Risk-of-bias judgement**		Low
Optional: What is the predicted direction of bias arising from the randomization process?		NA / Favours experimental / Favours comparator / Towards null /Away from null / Unpredictable

Domain 2: Risk of bias due to deviations from the intended interventions (*effect of assignment to intervention*)

Signalling questions	Comments	Response options
**2.1. Were participants aware of their assigned intervention during the trial?**	The interventions involve the use of a specifical device for oxygenation in awake patients, so both patients and healthcare providers are likely aware of the interventions.	PY
**2.2. Were carers and people delivering the interventions aware of participants' assigned intervention during the trial?**		PY
**2.3. If Y/PY/NI to 2.1 or 2.2: Were there deviations from the intended intervention that arose because of the trial context?**	There were no reported deviations from the intended intervention.	PN
**2.4 If Y/PY to 2.3: Were these deviations likely to have affected the outcome?**		NA
**2.5. If Y/PY/NI to 2.4: Were these deviations from intended intervention balanced between groups?**		NA
**2.6 Was an appropriate analysis used to estimate the effect of assignment to intervention?**		NI
**2.7 If N/PN/NI to 2.6: Was there potential for a substantial impact (on the result) of the failure to analyse participants in the group to which they were randomized?**		PY
**Risk-of-bias judgement**		High
Optional: What is the predicted direction of bias due to deviations from intended interventions?		NA / Favours experimental / Favours comparator / Towards null /Away from null / Unpredictable

Domain 3: Missing outcome data

Signalling questions	Comments	Response options
**3.1 Were data for this outcome available for all, or nearly all, participants randomized?**		Y
**3.2 If N/PN/NI to 3.1: Is there evidence that the result was not biased by missing outcome data?**		NA
**3.3 If N/PN to 3.2: Could missingness in the outcome depend on its true value?**		NA
**3.4 If Y/PY/NI to 3.3: Is it likely that missingness in the outcome depended on its true value?**		NA
**Risk-of-bias judgement**		Low
Optional: What is the predicted direction of bias due to missing outcome data?		NA / Favours experimental / Favours comparator / Towards null /Away from null / Unpredictable

Domain 4: Risk of bias in measurement of the outcome

Signalling questions	Comments	Response options
**4.1 Was the method of measuring the outcome inappropriate?**		N
**4.2 Could measurement or ascertainment of the outcome have differed between intervention groups?**	Although there is not explicit mention that concealment was maintained throughout outcomes evaluation and outcome assessment was performed by investigators who were not involved in patient care, it is unlikely that the measurement or ascertainment of the outcome differed between intervention groups.	N
**4.3 If N/PN/NI to 4.1 and 4.2: Were outcome assessors aware of the intervention received by study participants?**	There is not explicit mention that concealment was maintained throughout outcomes evaluation and outcome assessment was performed by investigators who were not involved in patient care.	PY
**4.4 If Y/PY/NI to 4.3: Could assessment of the outcome have been influenced by knowledge of intervention received?**	Although there is not explicit mention that concealment was maintained throughout outcomes evaluation and outcome assessment was performed by investigators who were not involved in patient care, it is unlikely that the measurement or ascertainment of the outcome differed between intervention groups, considering that re-intubation is an outcome that does not involve any judgement.	PN
**4.5 If Y/PY/NI to 4.4: Is it likely that assessment of the outcome was influenced by knowledge of intervention received?**		NA
**Risk-of-bias judgement**		Low
Optional: What is the predicted direction of bias in measurement of the outcome?		NA / Favours experimental / Favours comparator / Towards null /Away from null / Unpredictable

Domain 5: Risk of bias in selection of the reported result

Signalling questions	Comments	Response options
**5.1 Were the data that produced this result analysed in accordance with a pre-specified analysis plan that was finalized before unblinded outcome data were available for analysis?**	No information regarding trial registration and prespecified analysis plan publication.	NI
**Is the numerical result being assessed likely to have been selected, on the basis of the results, from...**		
**5.2. ... multiple eligible outcome measurements (e.g. scales, definitions, time points) within the outcome domain?**		N
**5.3 ... multiple eligible analyses of the data?**		N
**Risk-of-bias judgement**		Some concerns
Optional: What is the predicted direction of bias due to selection of the reported result?		NA / Favours experimental / Favours comparator / Towards null /Away from null / Unpredictable

Overall risk of bias

**Risk-of-bias judgement**		High
Optional: What is the overall predicted direction of bias for this outcome?		NA / Favours experimental / Favours comparator / Towards null /Away from null / Unpredictable

This work is licensed under a Creative Commons Attribution-NonCommercial-NoDerivatives 4.0 International License.

Revised Cochrane risk-of-bias tool for randomized trials (RoB 2)
TEMPLATE FOR COMPLETION

Edited by Julian PT Higgins, Jelena Savović, Matthew J Page, Jonathan AC Sterne
on behalf of the RoB2 Development Group

Version of 22 August 2019

The development of the RoB 2 tool was supported by the MRC Network of Hubs for Trials Methodology Research (MR/L004933/2- N61), with the support of the host MRC ConDuCT-II Hub (Collaboration and innovation for Difficult and Complex randomised controlled Trials In Invasive procedures - MR/K025643/1), by MRC research grant MR/M025209/1, and by a grant from The Cochrane Collaboration.

This work is licensed under a Creative Commons Attribution-NonCommercial-NoDerivatives 4.0 International License.

Study details

**Reference**	Jaber S, Lescot T, Futier E, Paugam-Burtz C, Seguin P, Ferrandiere M, Lasocki S, Mimoz O, Hengy B, Sannini A, Pottecher J, Abback PS, Riu B, Belafia F, Constantin JM, Masseret E, Beaussier M, Verzilli D, De Jong A, Chanques G, Brochard L, Molinari N; NIVAS Study Group. Effect of Noninvasive Ventilation on Tracheal Reintubation Among Patients With Hypoxemic Respiratory Failure Following Abdominal Surgery: A Randomized Clinical Trial. JAMA. 2016 Apr 5;315(13):1345-53. doi: 10.1001/jama.2016.2706. PMID: 26975890.

Study design

X Individually-randomized parallel-group trial

☐ Cluster-randomized parallel-group trial

☐ Individually randomized cross-over (or other matched) trial

For the purposes of this assessment, the interventions being compared are defined as

Experimental:	NIV	Comparator:	COT

**Specify which outcome is being assessed for risk of bias**	Re-intubation
**Specify the numerical result being assessed.** In case of multiple alternative analyses being presented, specify the numeric result (e.g. RR = 1.52 (95% CI 0.83 to 2.77) and/or a reference (e.g. to a table, figure or paragraph) that uniquely defines the result being assessed.	66/145 patients in the COT group vs. 9/148 patients in the NIV group<br>Absolute difference: −12.41 (−23.51 to −1.31)<br>P = 0.03

Is the review team's aim for this result…?

X to assess the effect of *assignment to intervention* (the 'intention-to-treat' effect)

☐ to assess the effect of *adhering to intervention* (the 'per-protocol' effect)

If the aim is to assess the effect of *adhering to intervention*, select the deviations from intended intervention that should be addressed (at least one must be checked):

☐ occurrence of non-protocol interventions
☐ failures in implementing the intervention that could have affected the outcome
☐ non-adherence to their assigned intervention by trial participants

Which of the following sources were <u>obtained</u> to help inform the risk-of-bias assessment? (tick as many as apply)

X Journal article(s) with results of the trial
X Trial protocol
☐ Statistical analysis plan (SAP)
☐ Non-commercial trial registry record (e.g. ClinicalTrials.gov record)
☐ Company-owned trial registry record (e.g. GSK Clinical Study Register record)
☐ "Grey literature" (e.g. unpublished thesis)
☐ Conference abstract(s) about the trial
☐ Regulatory document (e.g. Clinical Study Report, Drug Approval Package)
☐ Research ethics application
☐ Grant database summary (e.g. NIH RePORTER or Research Councils UK Gateway to Research)
☐ Personal communication with trialist
☐ Personal communication with the sponsor

Risk of bias assessment

Responses underlined in green are potential markers for low risk of bias, and responses in red are potential markers for a risk of bias. Where questions relate only to sign posts to other questions, no formatting is used.

Domain 1: Risk of bias arising from the randomization process

Signalling questions	Comments	Response options
**1.1 Was the allocation sequence random?**	Citation: " Randomization was performed centrally by the minimization method with the use of a computer-generated and blinded assignment sequence".	Y
**1.2 Was the allocation sequence concealed until participants were enrolled and assigned to interventions?**		PY
**1.3 Did baseline differences between intervention groups suggest a problem with the randomization process?**	Citation: " Groups were similar with respect to inclusion, site, duration of surgery, causes of acute respiratory failure, time from surgery, time from extubation to acute respiratory failure (Table 1 and Table 2), and gas exchange (Table 2 and eTable 1 in Supplement 1)."	N
**Risk-of-bias judgement**		Low
Optional: What is the predicted direction of bias arising from the randomization process?		NA / Favours experimental / Favours comparator / Towards null /Away from null / Unpredictable

Domain 2: Risk of bias due to deviations from the intended interventions (*effect of assignment to intervention*)

Signalling questions	Comments	Response options
**2.1. Were participants aware of their assigned intervention during the trial?**	Citation: "Third, although we applied predefined criteria for reintubation, bias cannot be completely ruled out because blinding with NIV was not feasible."	Y
**2.2. Were carers and people delivering the interventions aware of participants' assigned intervention during the trial?**		Y
**2.3. If Y/PY/NI to 2.1 or 2.2: Were there deviations from the intended intervention that arose because of the trial context?**	Although five patients did not receive standard oxygen therapy and two did not receive NIV after randomization, there is no evidence that serious deviations from the intended intervention arose because of the trial context.	PN
**2.4 If Y/PY to 2.3: Were these deviations likely to have affected the outcome?**		NA
**2.5. If Y/PY/NI to 2.4: Were these deviations from intended intervention balanced between groups?**		NA
**2.6 Was an appropriate analysis used to estimate the effect of assignment to intervention?**	Citation: "Among the 293 patients (mean age, 63.4 [SD, 13.8] years; n = 224 men) included in the intention-to-treat analysis, reintubation occurred in 49 of 148 (33.1%) in the NIV group and in 66 of 145 (45.5%) in the standard oxygen therapy group within 7 days after randomization (absolute difference, −12.4%; 95% CI, −23.5% to −1.3%; P = .03)."	Y
**2.7 If N/PN/NI to 2.6: Was there potential for a substantial impact (on the result) of the failure to analyse participants in the group to which they were randomized?**		NA
**Risk-of-bias judgement**		Low
Optional: What is the predicted direction of bias due to deviations from intended interventions?		NA / Favours experimental / Favours comparator / Towards null /Away from null / Unpredictable

Domain 3: Missing outcome data

Signalling questions	Comments	Response options
**3.1 Were data for this outcome available for all, or nearly all, participants randomized?**	Citation: "Data on the primary outcome were available for all 293 remaining patients".	Y
**3.2 If N/PN/NI to 3.1: Is there evidence that the result was not biased by missing outcome data?**		NA
**3.3 If N/PN to 3.2: Could missingness in the outcome depend on its true value?**		NA
**3.4 If Y/PY/NI to 3.3: Is it likely that missingness in the outcome depended on its true value?**		NA
**Risk-of-bias judgement**		Low
Optional: What is the predicted direction of bias due to missing outcome data?		NA / Favours experimental / Favours comparator / Towards null /Away from null / Unpredictable

Domain 4: Risk of bias in measurement of the outcome

Signalling questions	Comments	Response options
**4.1 Was the method of measuring the outcome inappropriate?**		N
**4.2 Could measurement or ascertainment of the outcome have differed between intervention groups?**	Citations: “An independent data and safety monitoring committee oversaw the study conduct and reviewed blinded safety data”.	N
**4.3 If N/PN/NI to 4.1 and 4.2: Were outcome assessors aware of the intervention received by study participants?**	Citations: “An independent data and safety monitoring committee oversaw the study conduct and reviewed blinded safety data”. Although there is not explicit mention that concealment was maintained throughout outcomes evaluation and outcome assessment was performed by investigators who were not involved in patient care, this is likely to be the case, considering the overall methodological quality of the trial.	PN
**4.4 If Y/PY/NI to 4.3: Could assessment of the outcome have been influenced by knowledge of intervention received?**		NA
**4.5 If Y/PY/NI to 4.4: Is it likely that assessment of the outcome was influenced by knowledge of intervention received?**		NA
**Risk-of-bias judgement**		Low
Optional: What is the predicted direction of bias in measurement of the outcome?		NA / Favours experimental / Favours comparator / Towards null /Away from null / Unpredictable

Domain 5: Risk of bias in selection of the reported result

Signalling questions	Comments	Response options
**5.1 Were the data that produced this result analysed in accordance with a pre-specified analysis plan that was finalized before unblinded outcome data were available for analysis?**	Adherence to the protocol registered in ClinicalTrial.gov.	Y
**Is the numerical result being assessed likely to have been selected, on the basis of the results, from...**		
**5.2. ... multiple eligible outcome measurements (e.g. scales, definitions, time points) within the outcome domain?**		N
**5.3 ... multiple eligible analyses of the data?**		N
**Risk-of-bias judgement**		Low
Optional: What is the predicted direction of bias due to selection of the reported result?		NA / Favours experimental / Favours comparator / Towards null /Away from null / Unpredictable

Overall risk of bias

**Risk-of-bias judgement**		Low
Optional: What is the overall predicted direction of bias for this outcome?		NA / Favours experimental / Favours comparator / Towards null /Away from null / Unpredictable

This work is licensed under a Creative Commons Attribution-NonCommercial-NoDerivatives 4.0 International License.

Revised Cochrane risk-of-bias tool for randomized trials (RoB 2)
TEMPLATE FOR COMPLETION

Edited by Julian PT Higgins, Jelena Savović, Matthew J Page, Jonathan AC Sterne
on behalf of the RoB2 Development Group

Version of 22 August 2019

The development of the RoB 2 tool was supported by the MRC Network of Hubs for Trials Methodology Research (MR/L004933/2- N61), with the support of the host MRC ConDuCT-II Hub (Collaboration and innovation for Difficult and Complex randomised controlled Trials In Invasive procedures - MR/K025643/1), by MRC research grant MR/M025209/1, and by a grant from The Cochrane Collaboration.

This work is licensed under a Creative Commons Attribution-NonCommercial-NoDerivatives 4.0 International License.

Study details

Reference

Kindgen-Milles D, Müller E, Buhl R, Böhner H, Ritter D, Sandmann W, Tarnow J. Nasal-continuous positive airway pressure reduces pulmonary morbidity and length of hospital stay following thoracoabdominal aortic surgery. Chest. 2005 Aug;128(2):821-8. doi: 10.1378/chest.128.2.821. PMID: 16100174.

Study design

- X Individually-randomized parallel-group trial
- ☐ Cluster-randomized parallel-group trial
- ☐ Individually randomized cross-over (or other matched) trial

For the purposes of this assessment, the interventions being compared are defined as

Experimental: CPAP Comparator: COT

Specify which outcome is being assessed for risk of bias Re-intubation

Specify the numerical result being assessed. In case of multiple alternative analyses being presented, specify the numeric result (e.g. RR = 1.52 (95% CI 0.83 to 2.77) and/or a reference (e.g. to a table, figure or paragraph) that uniquely defines the result being assessed.

1/25 patient in the CPAP group vs. 4/25 patients in the COT group

Is the review team's aim for this result…?

- X to assess the effect of *assignment to intervention* (the 'intention-to-treat' effect)
- ☐ to assess the effect of *adhering to intervention* (the 'per-protocol' effect)

If the aim is to assess the effect of *adhering to intervention*, select the deviations from intended intervention that should be addressed (at least one must be checked):

- ☐ occurrence of non-protocol interventions
- ☐ failures in implementing the intervention that could have affected the outcome
- ☐ non-adherence to their assigned intervention by trial participants

Which of the following sources were obtained to help inform the risk-of-bias assessment? (tick as many as apply)

X Journal article(s) with results of the trial
☐ Trial protocol
☐ Statistical analysis plan (SAP)
☐ Non-commercial trial registry record (e.g. ClinicalTrials.gov record)
☐ Company-owned trial registry record (e.g. GSK Clinical Study Register record)
☐ “Grey literature” (e.g. unpublished thesis)
☐ Conference abstract(s) about the trial
☐ Regulatory document (e.g. Clinical Study Report, Drug Approval Package)
☐ Research ethics application
☐ Grant database summary (e.g. NIH RePORTER or Research Councils UK Gateway to Research)
☐ Personal communication with trialist
☐ Personal communication with the sponsor

Risk of bias assessment

Responses underlined in green are potential markers for low risk of bias, and responses in red are potential markers for a risk of bias. Where questions relate only to sign posts to other questions, no formatting is used.

Domain 1: Risk of bias arising from the randomization process

Signalling questions	Comments	Response options
**1.1 Was the allocation sequence random?**	Citation: "After arrival in the ICU, patients were randomized by means of a computer-generated randomization list".	Y
**1.2 Was the allocation sequence concealed until participants were enrolled and assigned to interventions?**	There is no mention regarding the concealment of the randomization list.	NI
**1.3 Did baseline differences between intervention groups suggest a problem with the randomization process?**	Citation: "One patient in the study group had undergone a tracheotomy for prolonged mechanical ventilation after pneumonia several years before. There were no other statistically significant differences between the control group and the study group".	PN
**Risk-of-bias judgement**		Low
Optional: What is the predicted direction of bias arising from the randomization process?		NA / Favours experimental / Favours comparator / Towards null /Away from null / Unpredictable

Domain 2: Risk of bias due to deviations from the intended interventions (*effect of assignment to intervention*)

Signalling questions	Comments	Response options
**2.1. Were participants aware of their assigned intervention during the trial?**	It is likely that patients and caregivers could not be blinded to the treatment allocation.	Y
**2.2. Were carers and people delivering the interventions aware of participants' assigned intervention during the trial?**		Y
**2.3. If Y/PY/NI to 2.1 or 2.2: Were there deviations from the intended intervention that arose because of the trial context?**	Although one patient refused nCPAP therapy after 4 h because of discomfort in the study group, no major deviations from the intended intervention arose because of the trial context	PN
**2.4 If Y/PY to 2.3: Were these deviations likely to have affected the outcome?**		NA
**2.5. If Y/PY/NI to 2.4: Were these deviations from intended intervention balanced between groups?**		NA
**2.6 Was an appropriate analysis used to estimate the effect of assignment to intervention?**	Citation: "The statistical analysis was performed on an intention-to-treat basis."	Y
**2.7 If N/PN/NI to 2.6: Was there potential for a substantial impact (on the result) of the failure to analyse participants in the group to which they were randomized?**		NA
**Risk-of-bias judgement**		Low
Optional: What is the predicted direction of bias due to deviations from intended interventions?		NA / Favours experimental / Favours comparator / Towards null /Away from null / Unpredictable

Domain 3: Missing outcome data

Signalling questions	Comments	Response options
**3.1 Were data for this outcome available for all, or nearly all, participants randomized?**		Y
**3.2 If N/PN/NI to 3.1: Is there evidence that the result was not biased by missing outcome data?**		NA
**3.3 If N/PN to 3.2: Could missingness in the outcome depend on its true value?**		NA
**3.4 If Y/PY/NI to 3.3: Is it likely that missingness in the outcome depended on its true value?**		NA
**Risk-of-bias judgement**		Low
Optional: What is the predicted direction of bias due to missing outcome data?		NA / Favours experimental / Favours comparator / Towards null /Away from null / Unpredictable

Domain 4: Risk of bias in measurement of the outcome

Signalling questions	Comments	Response options
**4.1 Was the method of measuring the outcome inappropriate?**		N
**4.2 Could measurement or ascertainment of the outcome have differed between intervention groups?**	Although there is not explicit mention that concealment was maintained throughout outcomes evaluation and outcome assessment was performed by investigators who were not involved in patient care, it is unlikely that the measurement or ascertainment of the outcome differed between intervention groups.	PN
**4.3 If N/PN/NI to 4.1 and 4.2: Were outcome assessors aware of the intervention received by study participants?**	Although the decision to transfer patients to a general ward was made by physicians who were not informed about the group allocation of the patient, there is not explicit mention that outcome assessors were blinded to the intervention received by study participants.	PY
**4.4 If Y/PY/NI to 4.3: Could assessment of the outcome have been influenced by knowledge of intervention received?**	Although there is not explicit mention that concealment was maintained throughout outcomes evaluation and outcome assessment was performed by investigators who were not involved in patient care, it is unlikely that the measurement or ascertainment of the outcome differed between intervention groups, considering that re-intubation is an outcome that does not involve any judgement.	PN
**4.5 If Y/PY/NI to 4.4: Is it likely that assessment of the outcome was influenced by knowledge of intervention received?**		NA
**Risk-of-bias judgement**		Low
Optional: What is the predicted direction of bias in measurement of the outcome?		NA / Favours experimental / Favours comparator / Towards null /Away from null / Unpredictable

Domain 5: Risk of bias in selection of the reported result

Signalling questions	Comments	Response options
**5.1 Were the data that produced this result analysed in accordance with a pre-specified analysis plan that was finalized before unblinded outcome data were available for analysis?**	There is no mention of any trial registration or prespecified statistical analysis plan.	NI
**Is the numerical result being assessed likely to have been selected, on the basis of the results, from...**		
**5.2. ... multiple eligible outcome measurements (e.g. scales, definitions, time points) within the outcome domain?**		N
**5.3 ... multiple eligible analyses of the data?**		N
**Risk-of-bias judgement**		Some concerns
Optional: What is the predicted direction of bias due to selection of the reported result?		NA / Favours experimental / Favours comparator / Towards null /Away from null / Unpredictable

Overall risk of bias

**Risk-of-bias judgement**		Some concerns
Optional: What is the overall predicted direction of bias for this outcome?		NA / Favours experimental / Favours comparator / Towards null /Away from null / Unpredictable

This work is licensed under a Creative Commons Attribution-NonCommercial-NoDerivatives 4.0 International License.

Revised Cochrane risk-of-bias tool for randomized trials (RoB 2)
TEMPLATE FOR COMPLETION

Edited by Julian PT Higgins, Jelena Savović, Matthew J Page, Jonathan AC Sterne
on behalf of the RoB2 Development Group

Version of 22 August 2019

The development of the RoB 2 tool was supported by the MRC Network of Hubs for Trials Methodology Research (MR/L004933/2- N61), with the support of the host MRC ConDuCT-II Hub (Collaboration and innovation for Difficult and Complex randomised controlled Trials In Invasive procedures - MR/K025643/1), by MRC research grant MR/M025209/1, and by a grant from The Cochrane Collaboration.

This work is licensed under a Creative Commons Attribution-NonCommercial-NoDerivatives 4.0 International License.

Study details

**Reference**	Lockstone J, Parry SM, Denehy L, Robertson IK, Story D, Boden I. Non-Invasive Positive airway Pressure thErapy to Reduce Postoperative Lung complications following Upper abdominal Surgery (NIPPER PLUS): a pilot randomised control trial. Physiotherapy. 2022 Dec;117:25-34. doi: 10.1016/j.physio.2022.06.001. Epub 2022 Jun 13. PMID: 36242928.

Study design

X Individually-randomized parallel-group trial

☐ Cluster-randomized parallel-group trial

☐ Individually randomized cross-over (or other matched) trial

For the purposes of this assessment, the interventions being compared are defined as

Experimental:	NIV	Comparator:	HFNO

**Specify which outcome is being assessed for risk of bias**	Re-intubation
**Specify the numerical result being assessed.** In case of multiple alternative analyses being presented, specify the numeric result (e.g. RR = 1.52 (95% CI 0.83 to 2.77) and/or a reference (e.g. to a table, figure or paragraph) that uniquely defines the result being assessed.	3/64 patients in the NIV group vs. 7/65 patients in the HFNO group (adjusted HR 0.39; 95% CI 0.18–0.79)

Is the review team's aim for this result…?

X to assess the effect of *assignment to intervention* (the 'intention-to-treat' effect)

☐ to assess the effect of *adhering to intervention* (the 'per-protocol' effect)

If the aim is to assess the effect of *adhering to intervention*, select the deviations from intended intervention that should be addressed (at least one must be checked):

☐ occurrence of non-protocol interventions
☐ failures in implementing the intervention that could have affected the outcome
☐ non-adherence to their assigned intervention by trial participants

Which of the following sources were obtained to help inform the risk-of-bias assessment? (tick as many as apply)

X Journal article(s) with results of the trial
X Trial protocol
☐ Statistical analysis plan (SAP)
☐ Non-commercial trial registry record (e.g. ClinicalTrials.gov record)
☐ Company-owned trial registry record (e.g. GSK Clinical Study Register record)
☐ “Grey literature” (e.g. unpublished thesis)
☐ Conference abstract(s) about the trial
☐ Regulatory document (e.g. Clinical Study Report, Drug Approval Package)
☐ Research ethics application
☐ Grant database summary (e.g. NIH RePORTER or Research Councils UK Gateway to Research)
☐ Personal communication with trialist
☐ Personal communication with the sponsor

Risk of bias assessment

Responses underlined in green are potential markers for low risk of bias, and responses in red are potential markers for a risk of bias. Where questions relate only to sign posts to other questions, no formatting is used.

Domain 1: Risk of bias arising from the randomization process

Signalling questions	Comments	Response options
**1.1 Was the allocation sequence random?**	"Consecutive eligible, consenting patients were randomly assigned by the lead or a site investigator in a 1:1 ratio postsurgery, using concealed opaque envelopes pre-prepared by a research assistant independent to the trial. Randomisation was stratified to post-surgical destination (ICU or WARD) and allocation sequence was generated by a web-based computer program"; "Following construction of the randomisation envelopes, the allocation sequence is locked securely in the hospital's research institute and unavailable to site investigators, those who enrol participants and/or assign interventions".	Y
**1.2 Was the allocation sequence concealed until participants were enrolled and assigned to interventions?**		Y
**1.3 Did baseline differences between intervention groups suggest a problem with the randomization process?**	Citation: "There were significant baseline imbalances between groups with respect to respiratory co-morbidity and functional comorbidity index, which were adjusted for within the analysis. Whilst baseline differences between groups also exist for surgical categories, this did not reach significance."	PN
**Risk-of-bias judgement**		Low
Optional: What is the predicted direction of bias arising from the randomization process?		NA / Favours experimental / Favours comparator / Towards null /Away from null / Unpredictable

Domain 2: Risk of bias due to deviations from the intended interventions (*effect of assignment to intervention*)

Signalling questions	Comments	Response options
**2.1. Were participants aware of their assigned intervention during the trial?**	It is likely that patients could not be blinded to the treatment allocation.	Y
**2.2. Were carers and people delivering the interventions aware of participants' assigned intervention during the trial?**	Citation: "Due to the nature of intervention, postoperative ward staff including nurses, doctors and treating physiotherapists are unable to be blinded."	Y
**2.3. If Y/PY/NI to 2.1 or 2.2: Were there deviations from the intended intervention that arose because of the trial context?**	Citations: "The planned physiotherapy-led NIV intervention was not successfully delivered, with only 33 (52%) participants receiving NIV as per-protocol. The main barriers to NIV delivery were physiotherapy service-related limitations with 12 (19%) participants not able to have at least one session of NIV due to unavailability of physiotherapy staff. Other reasons for non-delivery were medical advice to withhold therapy (n = 10, 3%) and participant refusal (n = 8, 3%)"; "The HFNC protocol was successful, with 81% (n = 105) of participants receiving HFNC for 48-continuous hours."	Y
**2.4 If Y/PY to 2.3: Were these deviations likely to have affected the outcome?**		PY
**2.5. If Y/PY/NI to 2.4: Were these deviations from intended intervention balanced between groups?**	Citations: "The planned physiotherapy-led NIV intervention was not successfully delivered, with only 33 (52%) participants receiving NIV as per-protocol"; "The HFNC protocol was successful, with 81% (n = 105) of participants receiving HFNC for 48-continuous hours."	N
**2.6 Was an appropriate analysis used to estimate the effect of assignment to intervention?**	Citation: "Analyses were conducted as intention to-treat".	Y
**2.7 If N/PN/NI to 2.6: Was there potential for a substantial impact (on the result) of the failure to analyse participants in the group to which they were randomized?**		NA
**Risk-of-bias judgement**		High

Optional: What is the predicted direction of bias due to deviations from intended interventions?		NA / Favours experimental / Favours comparator / Towards null /Away from null / Unpredictable

Domain 3: Missing outcome data

Signalling questions	Comments	Response options
**3.1 Were data for this outcome available for all, or nearly all, participants randomized?**		Y
**3.2 If N/PN/NI to 3.1: Is there evidence that the result was not biased by missing outcome data?**		NA
**3.3 If N/PN to 3.2: Could missingness in the outcome depend on its true value?**		NA
**3.4 If Y/PY/NI to 3.3: Is it likely that missingness in the outcome depended on its true value?**		NA
**Risk-of-bias judgement**		Low
Optional: What is the predicted direction of bias due to missing outcome data?		NA / Favours experimental / Favours comparator / Towards null /Away from null / Unpredictable

Domain 4: Risk of bias in measurement of the outcome

Signalling questions	Comments	Response options
**4.1 Was the method of measuring the outcome inappropriate?**		N
**4.2 Could measurement or ascertainment of the outcome have differed between intervention groups?**	Citation: "Outcome assessors and statisticians not involved in postoperative clinical management were blinded."	N
**4.3 If N/PN/NI to 4.1 and 4.2: Were outcome assessors aware of the intervention received by study participants?**	Citation: "Outcome assessors and statisticians not involved in postoperative clinical management were blinded."	N
**4.4 If Y/PY/NI to 4.3: Could assessment of the outcome have been influenced by knowledge of intervention received?**		NA
**4.5 If Y/PY/NI to 4.4: Is it likely that assessment of the outcome was influenced by knowledge of intervention received?**		NA
**Risk-of-bias judgement**		Low
Optional: What is the predicted direction of bias in measurement of the outcome?		NA / Favours experimental / Favours comparator / Towards null /Away from null / Unpredictable

Domain 5: Risk of bias in selection of the reported result

Signalling questions	Comments	Response options
**5.1 Were the data that produced this result analysed in accordance with a pre-specified analysis plan that was finalized before unblinded outcome data were available for analysis?**	Adherence to the protocol registered in the Australian New Zealand Clinical Trials Registry.	Y
**Is the numerical result being assessed likely to have been selected, on the basis of the results, from...**		
**5.2. ... multiple eligible outcome measurements (e.g. scales, definitions, time points) within the outcome domain?**		N
**5.3 ... multiple eligible analyses of the data?**		N
**Risk-of-bias judgement**		Low
Optional: What is the predicted direction of bias due to selection of the reported result?		NA / Favours experimental / Favours comparator / Towards null /Away from null / Unpredictable

Overall risk of bias

**Risk-of-bias judgement**		High
Optional: What is the overall predicted direction of bias for this outcome?		NA / Favours experimental / Favours comparator / Towards null /Away from null / Unpredictable

This work is licensed under a Creative Commons Attribution-NonCommercial-NoDerivatives 4.0 International License.

Revised Cochrane risk-of-bias tool for randomized trials (RoB 2)
TEMPLATE FOR COMPLETION

Edited by Julian PT Higgins, Jelena Savović, Matthew J Page, Jonathan AC Sterne
on behalf of the RoB2 Development Group

Version of 22 August 2019

The development of the RoB 2 tool was supported by the MRC Network of Hubs for Trials Methodology Research (MR/L004933/2- N61), with the support of the host MRC ConDuCT-II Hub (Collaboration and innovation for Difficult and Complex randomised controlled Trials In Invasive procedures - MR/K025643/1), by MRC research grant MR/M025209/1, and by a grant from The Cochrane Collaboration.

This work is licensed under a Creative Commons Attribution-NonCommercial-NoDerivatives 4.0 International License.

Study details

**Reference**	Olper L, Bignami E, Di Prima AL, Albini S, Nascimbene S, Cabrini L, Landoni G, Alfieri O. Continuous Positive Airway Pressure Versus Oxygen Therapy in the Cardiac Surgical Ward: A Randomized Trial. J Cardiothorac Vasc Anesth. 2017 Feb;31(1):115-121. doi: 10.1053/j.jvca.2016.08.007. Epub 2016 Aug 10. PMID: 27771274.

Study design

X Individually-randomized parallel-group trial
☐ Cluster-randomized parallel-group trial
☐ Individually randomized cross-over (or other matched) trial

For the purposes of this assessment, the interventions being compared are defined as

Experimental: CPAP Comparator: COT

**Specify which outcome is being assessed for risk of bias**	Re-intubation
**Specify the numerical result being assessed.** In case of multiple alternative analyses being presented, specify the numeric result (e.g. RR = 1.52 (95% CI 0.83 to 2.77) and/or a reference (e.g. to a table, figure or paragraph) that uniquely defines the result being assessed.	1/31 patients in the COT group vs. 0/33 patients in the CPAP group (p = 0.48)

Is the review team's aim for this result…?

X to assess the effect of *assignment to intervention* (the 'intention-to-treat' effect)
☐ to assess the effect of *adhering to intervention* (the 'per-protocol' effect)

If the aim is to assess the effect of *adhering to intervention*, select the deviations from intended intervention that should be addressed (at least one must be checked):

☐ occurrence of non-protocol interventions
☐ failures in implementing the intervention that could have affected the outcome
☐ non-adherence to their assigned intervention by trial participants

Which of the following sources were obtained to help inform the risk-of-bias assessment? (tick as many as apply)

X Journal article(s) with results of the trial

☐ Trial protocol

☐ Statistical analysis plan (SAP)

X Non-commercial trial registry record (e.g. ClinicalTrials.gov record)

☐ Company-owned trial registry record (e.g. GSK Clinical Study Register record)

☐ "Grey literature" (e.g. unpublished thesis)

☐ Conference abstract(s) about the trial

☐ Regulatory document (e.g. Clinical Study Report, Drug Approval Package)

☐ Research ethics application

☐ Grant database summary (e.g. NIH RePORTER or Research Councils UK Gateway to Research)

☐ Personal communication with trialist

☐ Personal communication with the sponsor

Risk of bias assessment

Responses underlined in green are potential markers for low risk of bias, and responses in red are potential markers for a risk of bias. Where questions relate only to sign posts to other questions, no formatting is used.

Domain 1: Risk of bias arising from the randomization process

Signalling questions	Comments	Response options
**1.1 Was the allocation sequence random?**	Citation: "Randomization was performed by a person not involved in the study in a 1:1 ratio in blocks of 20 patients using a computer-generated list. Assigned treatment (CPAP vs standard treatment) was concealed in sealed, numbered opaque envelopes by a person not involved in the study.	Y
**1.2 Was the allocation sequence concealed until participants were enrolled and assigned to interventions?**		PY
**1.3 Did baseline differences between intervention groups suggest a problem with the randomization process?**	Citation: “The two groups of patients had similar baseline characteristics”.	N
**Risk-of-bias judgement**		Low
Optional: What is the predicted direction of bias arising from the randomization process?		NA / Favours experimental / Favours comparator / Towards null /Away from null / Unpredictable

Domain 2: Risk of bias due to deviations from the intended interventions (*effect of assignment to intervention*)

Signalling questions	Comments	Response options
**2.1. Were participants aware of their assigned intervention during the trial?**	The interventions involved the use of a specifical device for oxygenation in awake patients, so both patients and healthcare providers were aware of the interventions. Citation: "The sample size was not blinded".	Y
**2.2. Were carers and people delivering the interventions aware of participants' assigned intervention during the trial?**		Y
**2.3. If Y/PY/NI to 2.1 or 2.2: Were there deviations from the intended intervention that arose because of the trial context?**	Citations: "in some patients especially, during the second day of treatment, we could not apply the amount of CPAP prescribed (3-9 hours per day)."<br>Moreover, two patients in the control group crossed over to NIV treatment because of worsening respiratory failure. Citation: "few patients [...] were later discovered not to have the inclusion criteria (inclusion in other studies in 2 patients)."	Y
**2.4 If Y/PY to 2.3: Were these deviations likely to have affected the outcome?**	Citation: "We continued to collect all data about these patients even in the presence of a protocol deviation." Minor deviations from the intended intervention that arose because of the trial context, which were likely not to affect the outcome.	PN
**2.5. If Y/PY/NI to 2.4: Were these deviations from intended intervention balanced between groups?**		NA
**2.6 Was an appropriate analysis used to estimate the effect of assignment to intervention?**	Citation: "All data analysis was carried out according to a pre-established intention-to-treat analysis plan, including those few patients who were later discovered not to have the inclusion criteria (inclusion in other studies in 2 patients)".	Y
**2.7 If N/PN/NI to 2.6: Was there potential for a substantial impact (on the result) of the failure to analyse participants in the group to which they were randomized?**		NA
**Risk-of-bias judgement**		Some concerns
Optional: What is the predicted direction of bias due to deviations from intended interventions?		NA / Favours experimental / Favours comparator / Towards null /Away from null / Unpredictable

Domain 3: Missing outcome data

Signalling questions	Comments	Response options
**3.1 Were data for this outcome available for all, or nearly all, participants randomized?**	Citation: "All 64 randomized patients completed their hospital follow up for major outcomes (PaO2/FiO2 after 48 hours and length of hospital stay). Missing data for baseline characteristics and secondary outcomes were < 10% if not otherwise stated in tables."	Y
**3.2 If N/PN/NI to 3.1: Is there evidence that the result was not biased by missing outcome data?**		NA
**3.3 If N/PN to 3.2: Could missingness in the outcome depend on its true value?**		NA
**3.4 If Y/PY/NI to 3.3: Is it likely that missingness in the outcome depended on its true value?**		NA
**Risk-of-bias judgement**		Low
Optional: What is the predicted direction of bias due to missing outcome data?		NA / Favours experimental / Favours comparator / Towards null /Away from null / Unpredictable

Domain 4: Risk of bias in measurement of the outcome

Signalling questions	Comments	Response options
**4.1 Was the method of measuring the outcome inappropriate?**	Re-intubation is an observer-reported outcome.	N
**4.2 Could measurement or ascertainment of the outcome have differed between intervention groups?**	Although there is not explicit mention that concealment was maintained throughout outcomes evaluation and outcome assessment was performed by investigators who were not involved in patient care, re-intubation is an objectifiable outcome. Therefore, it is not likely that the ascertainment of the outcome differed between intervention group	PN
**4.3 If N/PN/NI to 4.1 and 4.2: Were outcome assessors aware of the intervention received by study participants?**	There is not explicit mention that concealment was maintained throughout outcomes evaluation and outcome assessment was performed by investigators who were not involved in patient care.	PY
**4.4 If Y/PY/NI to 4.3: Could assessment of the outcome have been influenced by knowledge of intervention received?**	Re-intubation is an outcome that does not involve any judgement and it is unlikely that outcome assessors were influenced by knowledge of intervention received by study participants.	PN
**4.5 If Y/PY/NI to 4.4: Is it likely that assessment of the outcome was influenced by knowledge of intervention received?**		NA
**Risk-of-bias judgement**		Low
Optional: What is the predicted direction of bias in measurement of the outcome?		NA / Favours experimental / Favours comparator / Towards null /Away from null / Unpredictable

Domain 5: Risk of bias in selection of the reported result

Signalling questions	Comments	Response options
**5.1 Were the data that produced this result analysed in accordance with a pre-specified analysis plan that was finalized before unblinded outcome data were available for analysis?**	Despite the protocol was registered in ClinicalTrial.gov, there are not details regarding the analysis plan reported in the trial registration.	NI
**Is the numerical result being assessed likely to have been selected, on the basis of the results, from...**		
**5.2. ... multiple eligible outcome measurements (e.g. scales, definitions, time points) within the outcome domain?**		N
**5.3 ... multiple eligible analyses of the data?**		N
**Risk-of-bias judgement**		Some concerns
Optional: What is the predicted direction of bias due to selection of the reported result?		NA / Favours experimental / Favours comparator / Towards null /Away from null / Unpredictable

Overall risk of bias

**Risk-of-bias judgement**		Some concerns
Optional: What is the overall predicted direction of bias for this outcome?		NA / Favours experimental / Favours comparator / Towards null /Away from null / Unpredictable

This work is licensed under a Creative Commons Attribution-NonCommercial-NoDerivatives 4.0 International License.

Revised Cochrane risk-of-bias tool for randomized trials (RoB 2)
TEMPLATE FOR COMPLETION

Edited by Julian PT Higgins, Jelena Savović, Matthew J Page, Jonathan AC Sterne
on behalf of the RoB2 Development Group

Version of 22 August 2019

The development of the RoB 2 tool was supported by the MRC Network of Hubs for Trials Methodology Research (MR/L004933/2- N61), with the support of the host MRC ConDuCT-II Hub (Collaboration and innovation for Difficult and Complex randomised controlled Trials In Invasive procedures - MR/K025643/1), by MRC research grant MR/M025209/1, and by a grant from The Cochrane Collaboration.

This work is licensed under a Creative Commons Attribution-NonCommercial-NoDerivatives 4.0 International License.

Study details

Reference

Parke R, McGuinness S, Dixon R, Jull A. Open-label, phase II study of routine high-flow nasal oxygen therapy in cardiac surgical patients. Br J Anaesth. 2013 Dec;111(6):925-31. doi: 10.1093/bja/aet262. Epub 2013 Aug 6. PMID: 23921199.

Study design

- X Individually-randomized parallel-group trial
- ☐ Cluster-randomized parallel-group trial
- ☐ Individually randomized cross-over (or other matched) trial

For the purposes of this assessment, the interventions being compared are defined as

Experimental: NIV Comparator: COT

Specify which outcome is being assessed for risk of bias

Re-intubation

Specify the numerical result being assessed. In case of multiple alternative analyses being presented, specify the numeric result (e.g. RR = 1.52 (95% CI 0.83 to 2.77) and/or a reference (e.g. to a table, figure or paragraph) that uniquely defines the result being assessed.

0/171 patients in the COT group vs. 2/169 patients in the HFNO group

Is the review team's aim for this result…?

- X to assess the effect of *assignment to intervention* (the 'intention-to-treat' effect)
- ☐ to assess the effect of *adhering to intervention* (the 'per-protocol' effect)

If the aim is to assess the effect of *adhering to intervention*, select the deviations from intended intervention that should be addressed (at least one must be checked):

- ☐ occurrence of non-protocol interventions
- ☐ failures in implementing the intervention that could have affected the outcome
- ☐ non-adherence to their assigned intervention by trial participants

Which of the following sources were <u>obtained</u> to help inform the risk-of-bias assessment? (tick as many as apply)

X Journal article(s) with results of the trial

☐ Trial protocol

☐ Statistical analysis plan (SAP)

X Non-commercial trial registry record (e.g. ClinicalTrials.gov record)

☐ Company-owned trial registry record (e.g. GSK Clinical Study Register record)

☐ "Grey literature" (e.g. unpublished thesis)

☐ Conference abstract(s) about the trial

☐ Regulatory document (e.g. Clinical Study Report, Drug Approval Package)

☐ Research ethics application

☐ Grant database summary (e.g. NIH RePORTER or Research Councils UK Gateway to Research)

☐ Personal communication with trialist

☐ Personal communication with the sponsor

Risk of bias assessment

Responses underlined in green are potential markers for low risk of bias, and responses in red are potential markers for a risk of bias. Where questions relate only to sign posts to other questions, no formatting is used.

Domain 1: Risk of bias arising from the randomization process

Signalling questions	Comments	Response options
**1.1 Was the allocation sequence random?**	Citation: "Randomization was by computer-generated random numbers in blocks of 12, with the sequence generated by an independent statistician, stratified by body mass index (BMI) < 35 or BMI ≥35".	Y
**1.2 Was the allocation sequence concealed until participants were enrolled and assigned to interventions?**	Citation: "Allocation concealment was maintained by using opaque, sealed, sequentially numbered envelopes prepared by a person not involved with the study".	Y
**1.3 Did baseline differences between intervention groups suggest a problem with the randomization process?**	No between-group difference is reported.	N
**Risk-of-bias judgement**		Low
Optional: What is the predicted direction of bias arising from the randomization process?		NA / Favours experimental / Favours comparator / Towards null /Away from null / Unpredictable

Domain 2: Risk of bias due to deviations from the intended interventions (*effect of assignment to intervention*)

Signalling questions	Comments	Response options
**2.1. Were participants aware of their assigned intervention during the trial?**	Citation: "A pragmatic, open-label randomized controlled trial was undertaken at a single study centre in a large metropolitan hospital"-	Y
**2.2. Were carers and people delivering the interventions aware of participants' assigned intervention during the trial?**		Y
**2.3. If Y/PY/NI to 2.1 or 2.2: Were there deviations from the intended intervention that arose because of the trial context?**	No deviations from the intended interventions are reported.	PN
**2.4 If Y/PY to 2.3: Were these deviations likely to have affected the outcome?**		NA
**2.5. If Y/PY/NI to 2.4: Were these deviations from intended intervention balanced between groups?**		NA
**2.6 Was an appropriate analysis used to estimate the effect of assignment to intervention?**	Citation: "All analyses were conducted according to a predefined statistical analysis plan using the intention-to-treat principle, with no imputation for missing values."	Y
**2.7 If N/PN/NI to 2.6: Was there potential for a substantial impact (on the result) of the failure to analyse participants in the group to which they were randomized?**		NA
**Risk-of-bias judgement**		Low
Optional: What is the predicted direction of bias due to deviations from intended interventions?		NA / Favours experimental / Favours comparator / Towards null /Away from null / Unpredictable

Domain 3: Missing outcome data

Signalling questions	Comments	Response options
**3.1 Were data for this outcome available for all, or nearly all, participants randomized?**		Y
**3.2 If N/PN/NI to 3.1: Is there evidence that the result was not biased by missing outcome data?**		NA
**3.3 If N/PN to 3.2: Could missingness in the outcome depend on its true value?**		NA
**3.4 If Y/PY/NI to 3.3: Is it likely that missingness in the outcome depended on its true value?**		NA
**Risk-of-bias judgement**		Low
Optional: What is the predicted direction of bias due to missing outcome data?		NA / Favours experimental / Favours comparator / Towards null /Away from null / Unpredictable

Domain 4: Risk of bias in measurement of the outcome

Signalling questions	Comments	Response options
**4.1 Was the method of measuring the outcome inappropriate?**		N
**4.2 Could measurement or ascertainment of the outcome have differed between intervention groups?**	Although there is not explicit mention that concealment was maintained throughout outcomes evaluation and outcome assessment was performed by investigators who were not involved in patient care, it is unlikely that the measurement or ascertainment of the outcome differed between intervention groups.	N
**4.3 If N/PN/NI to 4.1 and 4.2: Were outcome assessors aware of the intervention received by study participants?**	There is not explicit mention that concealment was maintained throughout outcomes evaluation and outcome assessment was performed by investigators who were not involved in patient care.	Y
**4.4 If Y/PY/NI to 4.3: Could assessment of the outcome have been influenced by knowledge of intervention received?**	Although there is not explicit mention that concealment was maintained throughout outcomes evaluation and outcome assessment was performed by investigators who were not involved in patient care, it is unlikely that the measurement or ascertainment of the outcome differed between intervention groups, considering that re-intubation is an outcome that does not involve any judgement.	PN
**4.5 If Y/PY/NI to 4.4: Is it likely that assessment of the outcome was influenced by knowledge of intervention received?**		NA
**Risk-of-bias judgement**		Low
Optional: What is the predicted direction of bias in measurement of the outcome?		NA / Favours experimental / Favours comparator / Towards null /Away from null / Unpredictable

Domain 5: Risk of bias in selection of the reported result

Signalling questions	Comments	Response options
**5.1 Were the data that produced this result analysed in accordance with a pre-specified analysis plan that was finalized before unblinded outcome data were available for analysis?**	Adherence to the published protocol and trial registration.	Y
**Is the numerical result being assessed likely to have been selected, on the basis of the results, from...**		
**5.2. ... multiple eligible outcome measurements (e.g. scales, definitions, time points) within the outcome domain?**		N
**5.3 ... multiple eligible analyses of the data?**		N
**Risk-of-bias judgement**		Low
Optional: What is the predicted direction of bias due to selection of the reported result?		NA / Favours experimental / Favours comparator / Towards null /Away from null / Unpredictable

Overall risk of bias

**Risk-of-bias judgement**		Low
Optional: What is the overall predicted direction of bias for this outcome?		NA / Favours experimental / Favours comparator / Towards null /Away from null / Unpredictable

This work is licensed under a Creative Commons Attribution-NonCommercial-NoDerivatives 4.0 International License.

Revised Cochrane risk-of-bias tool for randomized trials (RoB 2)
TEMPLATE FOR COMPLETION

Edited by Julian PT Higgins, Jelena Savović, Matthew J Page, Jonathan AC Sterne
on behalf of the RoB2 Development Group

Version of 22 August 2019

The development of the RoB 2 tool was supported by the MRC Network of Hubs for Trials Methodology Research (MR/L004933/2- N61), with the support of the host MRC ConDuCT-II Hub (Collaboration and innovation for Difficult and Complex randomised controlled Trials In Invasive procedures - MR/K025643/1), by MRC research grant MR/M025209/1, and by a grant from The Cochrane Collaboration.

This work is licensed under a Creative Commons Attribution-NonCommercial-NoDerivatives 4.0 International License.

Study details

**Reference**	Pennisi MA, Bello G, Congedo MT, Montini L, Nachira D, Ferretti GM, Meacci E, Gualtieri E, De Pascale G, Grieco DL, Margaritora S, Antonelli M. Early nasal high-flow versus Venturi mask oxygen therapy after lung resection: a randomized trial. Crit Care. 2019 Feb 28;23(1):68. doi: 10.1186/s13054-019-2361-5. PMID: 30819227; PMCID: PMC6396480.

Study design

X Individually-randomized parallel-group trial
☐ Cluster-randomized parallel-group trial
☐ Individually randomized cross-over (or other matched) trial

For the purposes of this assessment, the interventions being compared are defined as

Experimental: HFNO   Comparator: COT

**Specify which outcome is being assessed for risk of bias**	Re-intubation
**Specify the numerical result being assessed.** In case of multiple alternative analyses being presented, specify the numeric result (e.g. RR = 1.52 (95% CI 0.83 to 2.77) and/or a reference (e.g. to a table, figure or paragraph) that uniquely defines the result being assessed.	1/48 patients in the COT group vs. 1/47 patients in the HFNO group

Is the review team's aim for this result…?

X to assess the effect of *assignment to intervention* (the 'intention-to-treat' effect)
☐ to assess the effect of *adhering to intervention* (the 'per-protocol' effect)

If the aim is to assess the effect of *adhering to intervention*, select the deviations from intended intervention that should be addressed (at least one must be checked):

☐ occurrence of non-protocol interventions
☐ failures in implementing the intervention that could have affected the outcome
☐ non-adherence to their assigned intervention by trial participants

Which of the following sources were <u>obtained</u> to help inform the risk-of-bias assessment? (tick as many as apply)

X	Journal article(s) with results of the trial
	Trial protocol
	Statistical analysis plan (SAP)
X	Non-commercial trial registry record (e.g. ClinicalTrials.gov record)
	Company-owned trial registry record (e.g. GSK Clinical Study Register record)
	“Grey literature” (e.g. unpublished thesis)
	Conference abstract(s) about the trial
	Regulatory document (e.g. Clinical Study Report, Drug Approval Package)
	Research ethics application
	Grant database summary (e.g. NIH RePORTER or Research Councils UK Gateway to Research)
	Personal communication with trialist
	Personal communication with the sponsor

Risk of bias assessment

Responses underlined in green are potential markers for low risk of bias, and responses in red are potential markers for a risk of bias. Where questions relate only to sign posts to other questions, no formatting is used.

Domain 1: Risk of bias arising from the randomization process

Signalling questions	Comments	Response options
**1.1 Was the allocation sequence random?**	Citation: "A computer-generated random allocation list was used to allocate enrolled patients to study arms.	Y
**1.2 Was the allocation sequence concealed until participants were enrolled and assigned to interventions?**	No mention regarding any strategy to conceal the allocation sequence.	NI
**1.3 Did baseline differences between intervention groups suggest a problem with the randomization process?**	Citation: "Demographics, most relevant clinical characteristics, main comorbidities, preoperative arterial blood gases and respiratory function, and surgical procedures are reported in Table 1 and were well balanced in the two study groups".	N
**Risk-of-bias judgement**		Some concerns
Optional: What is the predicted direction of bias arising from the randomization process?		NA / Favours experimental / Favours comparator / Towards null /Away from null / Unpredictable

Domain 2: Risk of bias due to deviations from the intended interventions (*effect of assignment to intervention*)

Signalling questions	Comments	Response options
**2.1. Were participants aware of their assigned intervention during the trial?**	Citation: "it was not possible to blind staff and participants to treatment allocation".	Y
**2.2. Were carers and people delivering the interventions aware of participants' assigned intervention during the trial?**		Y
**2.3. If Y/PY/NI to 2.1 or 2.2: Were there deviations from the intended intervention that arose because of the trial context?**	Citation: "One patient from the HFNC group was not included in the "modified intention-to-treat" population because, due to intolerance to the device, the patient received the allocated treatment for less than 6 h".	Y
**2.4 If Y/PY to 2.3: Were these deviations likely to have affected the outcome?**		PN
**2.5. If Y/PY/NI to 2.4: Were these deviations from intended intervention balanced between groups?**		NA
**2.6 Was an appropriate analysis used to estimate the effect of assignment to intervention?**	Citation: "The analysis was conducted on a "modified intention -to-treat" population that included all patients who underwent the allocated treatment for at least 6 h".	Y
**2.7 If N/PN/NI to 2.6: Was there potential for a substantial impact (on the result) of the failure to analyse participants in the group to which they were randomized?**		NA
**Risk-of-bias judgement**		Some concerns
Optional: What is the predicted direction of bias due to deviations from intended interventions?		NA / Favours experimental / Favours comparator / Towards null /Away from null / Unpredictable

Domain 3: Missing outcome data

Signalling questions	Comments	Response options
**3.1 Were data for this outcome available for all, or nearly all, participants randomized?**		Y
**3.2 If N/PN/NI to 3.1: Is there evidence that the result was not biased by missing outcome data?**		NA
**3.3 If N/PN to 3.2: Could missingness in the outcome depend on its true value?**		NA
**3.4 If Y/PY/NI to 3.3: Is it likely that missingness in the outcome depended on its true value?**		NA
**Risk-of-bias judgement**		Low
Optional: What is the predicted direction of bias due to missing outcome data?		NA / Favours experimental / Favours comparator / Towards null /Away from null / Unpredictable

Domain 4: Risk of bias in measurement of the outcome

Signalling questions	Comments	Response options
**4.1 Was the method of measuring the outcome inappropriate?**		N
**4.2 Could measurement or ascertainment of the outcome have differed between intervention groups?**	Although concealment was not maintained throughout outcomes evaluation, it is unlikely that the measurement or ascertainment of the outcome differed between intervention groups.	N
**4.3 If N/PN/NI to 4.1 and 4.2: Were outcome assessors aware of the intervention received by study participants?**	Citation: "we took into account clinically objectifiable endpoints that were unlikely affected by assessors' unblinding".	Y
**4.4 If Y/PY/NI to 4.3: Could assessment of the outcome have been influenced by knowledge of intervention received?**	It is unlikely that the measurement or ascertainment of the outcome differed between intervention groups, considering that re-intubation is an outcome that does not involve any judgement.<br><br>Citation: "we took into account clinically objectifiable endpoints that were unlikely affected by assessors' unblinding".	PN
**4.5 If Y/PY/NI to 4.4: Is it likely that assessment of the outcome was influenced by knowledge of intervention received?**		NA
**Risk-of-bias judgement**		Low
Optional: What is the predicted direction of bias in measurement of the outcome?		NA / Favours experimental / Favours comparator / Towards null /Away from null / Unpredictable

Domain 5: Risk of bias in selection of the reported result

Signalling questions	Comments	Response options
**5.1 Were the data that produced this result analysed in accordance with a pre-specified analysis plan that was finalized before unblinded outcome data were available for analysis?**	Adherence to the trial registration.	Y
**Is the numerical result being assessed likely to have been selected, on the basis of the results, from...**		
**5.2. ... multiple eligible outcome measurements (e.g. scales, definitions, time points) within the outcome domain?**		N
**5.3 ... multiple eligible analyses of the data?**		N
**Risk-of-bias judgement**		Low
Optional: What is the predicted direction of bias due to selection of the reported result?		NA / Favours experimental / Favours comparator / Towards null /Away from null / Unpredictable

Overall risk of bias

**Risk-of-bias judgement**		Some concerns
Optional: What is the overall predicted direction of bias for this outcome?		NA / Favours experimental / Favours comparator / Towards null /Away from null / Unpredictable

This work is licensed under a Creative Commons Attribution-NonCommercial-NoDerivatives 4.0 International License.

Revised Cochrane risk-of-bias tool for randomized trials (RoB 2)
TEMPLATE FOR COMPLETION

Edited by Julian PT Higgins, Jelena Savović, Matthew J Page, Jonathan AC Sterne
on behalf of the RoB2 Development Group

Version of 22 August 2019

The development of the RoB 2 tool was supported by the MRC Network of Hubs for Trials Methodology Research (MR/L004933/2- N61), with the support of the host MRC ConDuCT-II Hub (Collaboration and innovation for Difficult and Complex randomised controlled Trials In Invasive procedures - MR/K025643/1), by MRC research grant MR/M025209/1, and by a grant from The Cochrane Collaboration.

This work is licensed under a Creative Commons Attribution-NonCommercial-NoDerivatives 4.0 International License.

Study details

**Reference**	PRISM trial group. Postoperative continuous positive airway pressure to prevent pneumonia, re-intubation, and death after major abdominal surgery (PRISM): a multicentre, open-label, randomised, phase 3 trial. Lancet Respir Med. 2021 Nov;9(11):1221-1230. doi: 10.1016/S2213-2600(21)00089-8. Epub 2021 Jun 18. Erratum in: Lancet Respir Med. 2021 Sep;9(9):e95. PMID: 34153272.

Study design

X Individually-randomized parallel-group trial
☐ Cluster-randomized parallel-group trial
☐ Individually randomized cross-over (or other matched) trial

For the purposes of this assessment, the interventions being compared are defined as

Experimental: CPAP   Comparator: COT

**Specify which outcome is being assessed for risk of bias**	Re-intubation
**Specify the numerical result being assessed.** In case of multiple alternative analyses being presented, specify the numeric result (e.g. RR = 1.52 (95% CI 0.83 to 2.77) and/or a reference (e.g. to a table, figure or paragraph) that uniquely defines the result being assessed.	90/2398 patients in the COT group vs. 80/2397 in the CPAP group (p = 0.45)

Is the review team's aim for this result…?

X to assess the effect of *assignment to intervention* (the 'intention-to-treat' effect)
☐ to assess the effect of *adhering to intervention* (the 'per-protocol' effect)

If the aim is to assess the effect of *adhering to intervention*, select the deviations from intended intervention that should be addressed (at least one must be checked):

☐ occurrence of non-protocol interventions
☐ failures in implementing the intervention that could have affected the outcome
☐ non-adherence to their assigned intervention by trial participants

Which of the following sources were obtained to help inform the risk-of-bias assessment? (tick as many as apply)

X Journal article(s) with results of the trial
X Trial protocol
X Statistical analysis plan (SAP)
X Non-commercial trial registry record (e.g. ClinicalTrials.gov record)
☐ Company-owned trial registry record (e.g. GSK Clinical Study Register record)
☐ "Grey literature" (e.g. unpublished thesis)
☐ Conference abstract(s) about the trial
☐ Regulatory document (e.g. Clinical Study Report, Drug Approval Package)
☐ Research ethics application
☐ Grant database summary (e.g. NIH RePORTER or Research Councils UK Gateway to Research)
☐ Personal communication with trialist
☐ Personal communication with the sponsor

Risk of bias assessment

Responses underlined in green are potential markers for low risk of bias, and responses in red are potential markers for a risk of bias. Where questions relate only to sign posts to other questions, no formatting is used.

Domain 1: Risk of bias arising from the randomization process

<table>
<tr><th>Signalling questions</th><th>Comments</th><th>Response options</th></tr>
<tr><td>1.1 Was the allocation sequence random?</td><td rowspan="2">Citation: “Patients were randomly assigned (1:1) to receive the CPAP intervention or usual care using a computer-generated dynamic procedure with the use of minimisation to balance trial group assignments according to country, planned surgical procedure category, and planned use of epidural anaesthesia.”; “The randomisation system was accessed by investigators via a secure website, which concealed the allocation sequence”.</td><td>Y</td></tr>
<tr><td>1.2 Was the allocation sequence concealed until participants were enrolled and assigned to interventions?</td><td>Y</td></tr>
<tr><td>1.3 Did baseline differences between intervention groups suggest a problem with the randomization process?</td><td>No baseline differences between intervention groups were reported.</td><td>N</td></tr>
<tr><td>Risk-of-bias judgement</td><td></td><td>Low</td></tr>
<tr><td>Optional: What is the predicted direction of bias arising from the randomization process?</td><td></td><td>NA / Favours experimental / Favours comparator / Towards null /Away from null / Unpredictable</td></tr>
</table>

Domain 2: Risk of bias due to deviations from the intended interventions (*effect of assignment to intervention*)

Signalling questions	Comments	Response options
**2.1. Were participants aware of their assigned intervention during the trial?**	Citation: "It was not possible to mask patients or clinicians delivering the intervention to the study group allocation; however, investigators collecting follow-up data were masked to group allocation. To quantify the degree of blinding, each investigator collecting primary outcome data completed a self-assessment of blinding."	Y
**2.2. Were carers and people delivering the interventions aware of participants' assigned intervention during the trial?**		Y
**2.3. If Y/PY/NI to 2.1 or 2.2: Were there deviations from the intended intervention that arose because of the trial context?**	Deviations occurred in both study arms. In the CPAP group, 6.5% of patients did not receive CPAP, CPAP was administered for less than 4 hours duration in 28.6% of patients, and was administered with significant interruptions in 1.6% of patients. In the COT group, 0.6% of patients actually received CPAP.	Y
**2.4 If Y/PY to 2.3: Were these deviations likely to have affected the outcome?**	Citations: "To test the impact of intervention compliance on clinical effectiveness, we did a post-hoc per-protocol analysis including only patients who received CPAP for 4 h. The treatment effect in this analysis was similar to our primary analysis that included all patients, indicating that the absence of clinical effectiveness was not due to poor intervention compliance. This interpretation is further supported by the findings of our process evaluation, which showed that the delivery of the trial intervention within the complex system of postoperative care was difficult in many hospitals".	PN
**2.5. If Y/PY/NI to 2.4: Were these deviations from intended intervention balanced between groups?**		NA
**2.6 Was an appropriate analysis used to estimate the effect of assignment to intervention?**	Citation: "We used an intention-to-treat approach"	Y
**2.7 If N/PN/NI to 2.6: Was there potential for a substantial impact (on the result) of the failure to analyse participants in the group to which they were randomized?**		NA
**Risk-of-bias judgement**		Some concerns

Optional: What is the predicted direction of bias due to deviations from intended interventions?		NA / Favours experimental / Favours comparator / Towards null /Away from null / Unpredictable

Domain 3: Missing outcome data

Signalling questions	Comments	Response options
**3.1 Were data for this outcome available for all, or nearly all, participants randomized?**		Y
**3.2 If N/PN/NI to 3.1: Is there evidence that the result was not biased by missing outcome data?**		NA
**3.3 If N/PN to 3.2: Could missingness in the outcome depend on its true value?**		NA
**3.4 If Y/PY/NI to 3.3: Is it likely that missingness in the outcome depended on its true value?**		NA
**Risk-of-bias judgement**		Low
Optional: What is the predicted direction of bias due to missing outcome data?		NA / Favours experimental / Favours comparator / Towards null /Away from null / Unpredictable

Domain 4: Risk of bias in measurement of the outcome

Signalling questions	Comments	Response options
**4.1 Was the method of measuring the outcome inappropriate?**		N
**4.2 Could measurement or ascertainment of the outcome have differed between intervention groups?**	Citation: "It was not possible to mask patients or clinicians delivering the intervention to the study group allocation; however, investigators collecting follow-up data were masked to group allocation. To quantify the degree of blinding, each investigator collecting primary outcome data completed a self-assessment of blinding".	PN
**4.3 If N/PN/NI to 4.1 and 4.2: Were outcome assessors aware of the intervention received by study participants?**	Citations: "It was not possible to mask patients or clinicians delivering the intervention to the study group allocation; however, investigators collecting follow-up data were masked to group allocation. To quantify the degree of blinding, each investigator collecting primary outcome data completed a self-assessment of blinding"; "It was not feasible to mask patients and clinicians involved in the delivery of CPAP to group allocation due to the nature of the intervention. However, we controlled this bias through blinded outcome assessment and we achieved good compliance with these procedures."	N
**4.4 If Y/PY/NI to 4.3: Could assessment of the outcome have been influenced by knowledge of intervention received?**		NA
**4.5 If Y/PY/NI to 4.4: Is it likely that assessment of the outcome was influenced by knowledge of intervention received?**		NA
**Risk-of-bias judgement**		Low
Optional: What is the predicted direction of bias in measurement of the outcome?		NA / Favours experimental / Favours comparator / Towards null /Away from null / Unpredictable

Domain 5: Risk of bias in selection of the reported result

Signalling questions	Comments	Response options
**5.1 Were the data that produced this result analysed in accordance with a pre-specified analysis plan that was finalized before unblinded outcome data were available for analysis?**	Citation: “The trial protocol (appendix p 62) was approved by a research ethics committee in the UK (15/LO/1595) and by the local ethics committees or institutional review boards in other participating countries, and has been published previously”; “This study is registered with the ISRCTN registry, ISRCTN56012545”.	Y
**Is the numerical result being assessed likely to have been selected, on the basis of the results, from...**		
**5.2. ... multiple eligible outcome measurements (e.g. scales, definitions, time points) within the outcome domain?**		N
**5.3 ... multiple eligible analyses of the data?**		N
**Risk-of-bias judgement**		Low
Optional: What is the predicted direction of bias due to selection of the reported result?		NA / Favours experimental / Favours comparator / Towards null /Away from null / Unpredictable

Overall risk of bias

**Risk-of-bias judgement**		Some concerns
Optional: What is the overall predicted direction of bias for this outcome?		NA / Favours experimental / Favours comparator / Towards null /Away from null / Unpredictable

This work is licensed under a Creative Commons Attribution-NonCommercial-NoDerivatives 4.0 International License.

Revised Cochrane risk-of-bias tool for randomized trials (RoB 2)
TEMPLATE FOR COMPLETION

Edited by Julian PT Higgins, Jelena Savović, Matthew J Page, Jonathan AC Sterne
on behalf of the RoB2 Development Group

Version of 22 August 2019

The development of the RoB 2 tool was supported by the MRC Network of Hubs for Trials Methodology Research (MR/L004933/2- N61), with the support of the host MRC ConDuCT-II Hub (Collaboration and innovation for Difficult and Complex randomised controlled Trials In Invasive procedures - MR/K025643/1), by MRC research grant MR/M025209/1, and by a grant from The Cochrane Collaboration.

This work is licensed under a Creative Commons Attribution-NonCommercial-NoDerivatives 4.0 International License.

Study details

Reference Mazullo Filho JB, Bonfim VJ, Aquim EE. Noninvasive mechanical ventilation in immediate postoperative cardiac surgery patients. Rev Bras Ter Intensiva. 2010 Dec;22(4):363-8. English, Portuguese. PMID: 25302513.

Study design

X Individually-randomized parallel-group trial

☐ Cluster-randomized parallel-group trial

☐ Individually randomized cross-over (or other matched) trial

For the purposes of this assessment, the interventions being compared are defined as

Experimental: NIV Comparator: COT

Specify which outcome is being assessed for risk of bias Re-intubation

Specify the numerical result being assessed. In case of multiple alternative analyses being presented, specify the numeric result (e.g. RR = 1.52 (95% CI 0.83 to 2.77) and/or a reference (e.g. to a table, figure or paragraph) that uniquely defines the result being assessed.

3/27 patients in the COT group vs. 0/14 patients in the NIV group

Is the review team's aim for this result…?

x to assess the effect of *assignment to intervention* (the 'intention-to-treat' effect)

☐ to assess the effect of *adhering to intervention* (the 'per-protocol' effect)

If the aim is to assess the effect of *adhering to intervention*, select the deviations from intended intervention that should be addressed (at least one must be checked):

☐ occurrence of non-protocol interventions
☐ failures in implementing the intervention that could have affected the outcome
☐ non-adherence to their assigned intervention by trial participants

Which of the following sources were <u>obtained</u> to help inform the risk-of-bias assessment? (tick as many as apply)

x Journal article(s) with results of the trial
☐ Trial protocol
☐ Statistical analysis plan (SAP)
☐ Non-commercial trial registry record (e.g. ClinicalTrials.gov record)
☐ Company-owned trial registry record (e.g. GSK Clinical Study Register record)
☐ "Grey literature" (e.g. unpublished thesis)
☐ Conference abstract(s) about the trial
☐ Regulatory document (e.g. Clinical Study Report, Drug Approval Package)
☐ Research ethics application
☐ Grant database summary (e.g. NIH RePORTER or Research Councils UK Gateway to Research)
☐ Personal communication with trialist
☐ Personal communication with the sponsor

Risk of bias assessment

Responses underlined in green are potential markers for low risk of bias, and responses in red are potential markers for a risk of bias. Where questions relate only to sign posts to other questions, no formatting is used.

Domain 1: Risk of bias arising from the randomization process

Signalling questions	Comments	Response options
**1.1 Was the allocation sequence random?**	Citation "After extubation, patients were randomized into two groups"	PY
**1.2 Was the allocation sequence concealed until participants were enrolled and assigned to interventions?**		NI
**1.3 Did baseline differences between intervention groups suggest a problem with the randomization process?**	Apart from a slight increased body weight in the NIV group, no differences were reported between groups.	PN
**Risk-of-bias judgement**		Some concerns
Optional: What is the predicted direction of bias arising from the randomization process?		NA / Favours experimental / Favours comparator / Towards null /Away from null / Unpredictable

Domain 2: Risk of bias due to deviations from the intended interventions (*effect of assignment to intervention*)

Signalling questions	Comments	Response options
**2.1. Were participants aware of their assigned intervention during the trial?**	The interventions involve the use of a specifical device for oxygenation in awake patients, so both patients and healthcare providers are likely aware of the interventions.	PY
**2.2. Were carers and people delivering the interventions aware of participants' assigned intervention during the trial?**		PY
**2.3. If Y/PY/NI to 2.1 or 2.2: Were there deviations from the intended intervention that arose because of the trial context?**	There were no reported deviations from the intended intervention. However, the exclusion of patients in the NIV group requiring more than 2 hours of NIV may underestimate the number of patients requiring endotracheal intubation.	PN
**2.4 If Y/PY to 2.3: Were these deviations likely to have affected the outcome?**		NA
**2.5. If Y/PY/NI to 2.4: Were these deviations from intended intervention balanced between groups?**		NA
**2.6 Was an appropriate analysis used to estimate the effect of assignment to intervention?**		NI
**2.7 If N/PN/NI to 2.6: Was there potential for a substantial impact (on the result) of the failure to analyse participants in the group to which they were randomized?**		PY
**Risk-of-bias judgement**		High
Optional: What is the predicted direction of bias due to deviations from intended interventions?		NA / Favours experimental / Favours comparator / Towards null /Away from null / Unpredictable

Domain 3: Missing outcome data

Signalling questions	Comments	Response options
**3.1 Were data for this outcome available for all, or nearly all, participants randomized?**		Y
**3.2 If N/PN/NI to 3.1: Is there evidence that the result was not biased by missing outcome data?**		NA
**3.3 If N/PN to 3.2: Could missingness in the outcome depend on its true value?**		NA
**3.4 If Y/PY/NI to 3.3: Is it likely that missingness in the outcome depended on its true value?**		NA
**Risk-of-bias judgement**		Low
Optional: What is the predicted direction of bias due to missing outcome data?		NA / Favours experimental / Favours comparator / Towards null /Away from null / Unpredictable

Domain 4: Risk of bias in measurement of the outcome

Signalling questions	Comments	Response options
**4.1 Was the method of measuring the outcome inappropriate?**		N
**4.2 Could measurement or ascertainment of the outcome have differed between intervention groups?**	Although there is not explicit mention that concealment was maintained throughout outcomes evaluation and outcome assessment was performed by investigators who were not involved in patient care, it is unlikely that the measurement or ascertainment of the outcome differed between intervention groups.	N
**4.3 If N/PN/NI to 4.1 and 4.2: Were outcome assessors aware of the intervention received by study participants?**	There is not explicit mention that concealment was maintained throughout outcomes evaluation and outcome assessment was performed by investigators who were not involved in patient care.	PY
**4.4 If Y/PY/NI to 4.3: Could assessment of the outcome have been influenced by knowledge of intervention received?**	Although there is not explicit mention that concealment was maintained throughout outcomes evaluation and outcome assessment was performed by investigators who were not involved in patient care, it is unlikely that the measurement or ascertainment of the outcome differed between intervention groups, considering that re-intubation is an outcome that does not involve any judgement.	PN
**4.5 If Y/PY/NI to 4.4: Is it likely that assessment of the outcome was influenced by knowledge of intervention received?**		NA
**Risk-of-bias judgement**		Low
Optional: What is the predicted direction of bias in measurement of the outcome?		NA / Favours experimental / Favours comparator / Towards null /Away from null / Unpredictable

Domain 5: Risk of bias in selection of the reported result

Signalling questions	Comments	Response options
**5.1 Were the data that produced this result analysed in accordance with a pre-specified analysis plan that was finalized before unblinded outcome data were available for analysis?**	No information regarding trial registration and prespecified analysis plan publication.	NI
**Is the numerical result being assessed likely to have been selected, on the basis of the results, from...**		
**5.2. ... multiple eligible outcome measurements (e.g. scales, definitions, time points) within the outcome domain?**		N
**5.3 ... multiple eligible analyses of the data?**		N
**Risk-of-bias judgement**		Some concerns
Optional: What is the predicted direction of bias due to selection of the reported result?		NA / Favours experimental / Favours comparator / Towards null /Away from null / Unpredictable

Overall risk of bias

**Risk-of-bias judgement**		High
Optional: What is the overall predicted direction of bias for this outcome?		NA / Favours experimental / Favours comparator / Towards null /Away from null / Unpredictable

This work is licensed under a Creative Commons Attribution-NonCommercial-NoDerivatives 4.0 International License.

Revised Cochrane risk-of-bias tool for randomized trials (RoB 2)
TEMPLATE FOR COMPLETION

Edited by Julian PT Higgins, Jelena Savović, Matthew J Page, Jonathan AC Sterne
on behalf of the RoB2 Development Group

Version of 22 August 2019

The development of the RoB 2 tool was supported by the MRC Network of Hubs for Trials Methodology Research (MR/L004933/2- N61), with the support of the host MRC ConDuCT-II Hub (Collaboration and innovation for Difficult and Complex randomised controlled Trials In Invasive procedures - MR/K025643/1), by MRC research grant MR/M025209/1, and by a grant from The Cochrane Collaboration.

This work is licensed under a Creative Commons Attribution-NonCommercial-NoDerivatives 4.0 International License.

Study details

**Reference**	Sahin M, El H, Akkoç I. Comparison of Mask Oxygen Therapy and High-Flow Oxygen Therapy after Cardiopulmonary Bypass in Obese Patients. Can Respir J. 2018 Jan 28;2018:1039635. doi: 10.1155/2018/1039635. PMID: 29623135; PMCID: PMC5829344.

Study design

X Individually-randomized parallel-group trial

☐ Cluster-randomized parallel-group trial

☐ Individually randomized cross-over (or other matched) trial

For the purposes of this assessment, the interventions being compared are defined as

Experimental:	HFNO	Comparator:	COT

**Specify which outcome is being assessed for risk of bias**	Re-intubation
**Specify the numerical result being assessed.** In case of multiple alternative analyses being presented, specify the numeric result (e.g. RR = 1.52 (95% CI 0.83 to 2.77) and/or a reference (e.g. to a table, figure or paragraph) that uniquely defines the result being assessed.	2/50 patients in the COT group vs. 0/50 patients in the HFNO group

Is the review team's aim for this result…?

X to assess the effect of *assignment to intervention* (the 'intention-to-treat' effect)

☐ to assess the effect of *adhering to intervention* (the 'per-protocol' effect)

If the aim is to assess the effect of *adhering to intervention*, select the deviations from intended intervention that should be addressed (at least one must be checked):

☐ occurrence of non-protocol interventions

☐ failures in implementing the intervention that could have affected the outcome

☐ non-adherence to their assigned intervention by trial participants

Which of the following sources were <u>obtained</u> to help inform the risk-of-bias assessment? (tick as many as apply)

X Journal article(s) with results of the trial

☐ Trial protocol

☐ Statistical analysis plan (SAP)

☐ Non-commercial trial registry record (e.g. ClinicalTrials.gov record)

☐ Company-owned trial registry record (e.g. GSK Clinical Study Register record)

☐ "Grey literature" (e.g. unpublished thesis)

☐ Conference abstract(s) about the trial

☐ Regulatory document (e.g. Clinical Study Report, Drug Approval Package)

☐ Research ethics application

☐ Grant database summary (e.g. NIH RePORTER or Research Councils UK Gateway to Research)

☐ Personal communication with trialist

☐ Personal communication with the sponsor

Risk of bias assessment

Responses underlined in green are potential markers for low risk of bias, and responses in red are potential markers for a risk of bias. Where questions relate only to sign posts to other questions, no formatting is used.

Domain 1: Risk of bias arising from the randomization process

Signalling questions	Comments	Response options
**1.1 Was the allocation sequence random?**	Citation: "Randomization was done by a computer-based random number-sequencing program".	Y
**1.2 Was the allocation sequence concealed until participants were enrolled and assigned to interventions?**	There is no mention regarding the concealment of the allocation sequence.	NI
**1.3 Did baseline differences between intervention groups suggest a problem with the randomization process?**	No relevant baseline differences between intervention groups were reported.	N
**Risk-of-bias judgement**		Some concerns
Optional: What is the predicted direction of bias arising from the randomization process?		NA / Favours experimental / Favours comparator / Towards null /Away from null / Unpredictable

Domain 2: Risk of bias due to deviations from the intended interventions (*effect of assignment to intervention*)

Signalling questions	Comments	Response options
**2.1. Were participants aware of their assigned intervention during the trial?**	It is likely that patients and people delivering the intervention could not be blinded to the treatment allocation.	Y
**2.2. Were carers and people delivering the interventions aware of participants' assigned intervention during the trial?**		Y
**2.3. If Y/PY/NI to 2.1 or 2.2: Were there deviations from the intended intervention that arose because of the trial context?**	No deviations from the intended intervention arose because of the trial context	N
**2.4 If Y/PY to 2.3: Were these deviations likely to have affected the outcome?**		NA
**2.5. If Y/PY/NI to 2.4: Were these deviations from intended intervention balanced between groups?**		NA
**2.6 Was an appropriate analysis used to estimate the effect of assignment to intervention?**		NI
**2.7 If N/PN/NI to 2.6: Was there potential for a substantial impact (on the result) of the failure to analyse participants in the group to which they were randomized?**		PN
**Risk-of-bias judgement**		Some concerns
Optional: What is the predicted direction of bias due to deviations from intended interventions?		NA / Favours experimental / Favours comparator / Towards null /Away from null / Unpredictable

Domain 3: Missing outcome data

Signalling questions	Comments	Response options
**3.1 Were data for this outcome available for all, or nearly all, participants randomized?**		Y
**3.2 If N/PN/NI to 3.1: Is there evidence that the result was not biased by missing outcome data?**		NA
**3.3 If N/PN to 3.2: Could missingness in the outcome depend on its true value?**		NA
**3.4 If Y/PY/NI to 3.3: Is it likely that missingness in the outcome depended on its true value?**		NA
**Risk-of-bias judgement**		Low
Optional: What is the predicted direction of bias due to missing outcome data?		NA / Favours experimental / Favours comparator / Towards null /Away from null / Unpredictable

Domain 4: Risk of bias in measurement of the outcome

Signalling questions	Comments	Response options
**4.1 Was the method of measuring the outcome inappropriate?**		N
**4.2 Could measurement or ascertainment of the outcome have differed between intervention groups?**	Although there is not explicit mention that concealment was maintained throughout outcomes evaluation and outcome assessment was performed by investigators who were not involved in patient care, re-intubation is an objectifiable outcome. Therefore, it is not likely that the ascertainment of the outcome differed between intervention group	PN
**4.3 If N/PN/NI to 4.1 and 4.2: Were outcome assessors aware of the intervention received by study participants?**	There is not explicit mention that concealment was maintained throughout outcomes evaluation and outcome assessment was performed by investigators who were not involved in patient care.	PY
**4.4 If Y/PY/NI to 4.3: Could assessment of the outcome have been influenced by knowledge of intervention received?**	Re-intubation is an outcome that does not involve any judgement and it is unlikely that outcome assessors were influenced by knowledge of intervention received by study participants.	PN
**4.5 If Y/PY/NI to 4.4: Is it likely that assessment of the outcome was influenced by knowledge of intervention received?**		NA
**Risk-of-bias judgement**		Low
Optional: What is the predicted direction of bias in measurement of the outcome?		NA / Favours experimental / Favours comparator / Towards null /Away from null / Unpredictable

Domain 5: Risk of bias in selection of the reported result

Signalling questions	Comments	Response options
**5.1 Were the data that produced this result analysed in accordance with a pre-specified analysis plan that was finalized before unblinded outcome data were available for analysis?**	No trial registration or a pre-specified analysis plan are reported.	PN
**Is the numerical result being assessed likely to have been selected, on the basis of the results, from...**		
**5.2. ... multiple eligible outcome measurements (e.g. scales, definitions, time points) within the outcome domain?**		N
**5.3 ... multiple eligible analyses of the data?**		N
**Risk-of-bias judgement**		Some concerns
Optional: What is the predicted direction of bias due to selection of the reported result?		NA / Favours experimental / Favours comparator / Towards null /Away from null / Unpredictable

Overall risk of bias

**Risk-of-bias judgement**		Some concerns
Optional: What is the overall predicted direction of bias for this outcome?		NA / Favours experimental / Favours comparator / Towards null /Away from null / Unpredictable

This work is licensed under a Creative Commons Attribution-NonCommercial-NoDerivatives 4.0 International License.

Revised Cochrane risk-of-bias tool for randomized trials (RoB 2)
TEMPLATE FOR COMPLETION

Edited by Julian PT Higgins, Jelena Savović, Matthew J Page, Jonathan AC Sterne
on behalf of the RoB2 Development Group

Version of 22 August 2019

The development of the RoB 2 tool was supported by the MRC Network of Hubs for Trials Methodology Research (MR/L004933/2- N61), with the support of the host MRC ConDuCT-II Hub (Collaboration and innovation for Difficult and Complex randomised controlled Trials In Invasive procedures - MR/K025643/1), by MRC research grant MR/M025209/1, and by a grant from The Cochrane Collaboration.

This work is licensed under a Creative Commons Attribution-NonCommercial-NoDerivatives 4.0 International License.

Study details

**Reference**	Hoda Abdellatif Zaky Soliman, Dorreya Mohammed Fikry, Ahmed Mohamed El-Attar & Mohamed Shawky El Hadidy (2022) High flow nasal cannula effect on pulmonary complications after major elective upper abdominal surgeries: A randomized control study, Egyptian Journal of Anaesthesia, 38:1, 656-664, DOI: 10.1080/11101849.2022.2143175

Study design

X Individually-randomized parallel-group trial

☐ Cluster-randomized parallel-group trial

☐ Individually randomized cross-over (or other matched) trial

For the purposes of this assessment, the interventions being compared are defined as

Experimental: HFNO Comparator: COT

Specify which outcome is being assessed for risk of bias: Re-intubation

Specify the numerical result being assessed. In case of multiple alternative analyses being presented, specify the numeric result (e.g. RR = 1.52 (95% CI 0.83 to 2.77) and/or a reference (e.g. to a table, figure or paragraph) that uniquely defines the result being assessed.

2/40 patients in the COT group vs. 0/40 patients in the HFNO group (p = 0.494).

Is the review team's aim for this result…?

X to assess the effect of *assignment to intervention* (the 'intention-to-treat' effect)

☐ to assess the effect of *adhering to intervention* (the 'per-protocol' effect)

If the aim is to assess the effect of *adhering to intervention*, select the deviations from intended intervention that should be addressed (at least one must be checked):

☐ occurrence of non-protocol interventions

☐ failures in implementing the intervention that could have affected the outcome

☐ non-adherence to their assigned intervention by trial participants

Which of the following sources were obtained to help inform the risk-of-bias assessment? (tick as many as apply)

X	Journal article(s) with results of the trial
	Trial protocol
	Statistical analysis plan (SAP)
X	Non-commercial trial registry record (e.g. ClinicalTrials.gov record)
	Company-owned trial registry record (e.g. GSK Clinical Study Register record)
	“Grey literature” (e.g. unpublished thesis)
	Conference abstract(s) about the trial
	Regulatory document (e.g. Clinical Study Report, Drug Approval Package)
	Research ethics application
	Grant database summary (e.g. NIH RePORTER or Research Councils UK Gateway to Research)
	Personal communication with trialist
	Personal communication with the sponsor

Risk of bias assessment

Responses underlined in green are potential markers for low risk of bias, and responses in red are potential markers for a risk of bias. Where questions relate only to sign posts to other questions, no formatting is used.

Domain 1: Risk of bias arising from the randomization process

Signalling questions	Comments	Response options
**1.1 Was the allocation sequence random?**	Citation: " Postoperatively, at the intensive care unit using the closed-envelope technique, patients were randomly allocated into two groups with 40 patients each…”	Y
**1.2 Was the allocation sequence concealed until participants were enrolled and assigned to interventions?**		PY
**1.3 Did baseline differences between intervention groups suggest a problem with the randomization process?**	Citation: “There were no significant differences between the two groups in the following data: age, gender, type of surgery, duration of surgery, units of packed red blood cells transfused during the surgery, hemodynamic parameters, peripheral oxygen saturation, and the need to escalate respiratory support”.	PN
**Risk-of-bias judgement**	Low	Low
Optional: What is the predicted direction of bias arising from the randomization process?		NA / Favours experimental / Favours comparator / Towards null /Away from null / Unpredictable

Domain 2: Risk of bias due to deviations from the intended interventions (*effect of assignment to intervention*)

Signalling questions	Comments	Response options
**2.1. Were participants aware of their assigned intervention during the trial?**	The interventions involve the use of a specifical device for oxygenation in awake patients, so both patients and healthcare providers are aware of the interventions.	Y
**2.2. Were carers and people delivering the interventions aware of participants' assigned intervention during the trial?**		Y
**2.3. If Y/PY/NI to 2.1 or 2.2: Were there deviations from the intended intervention that arose because of the trial context?**	Seven patients were excluded after randomization, three in the COT group and two in the HFNO group.	Y
**2.4 If Y/PY to 2.3: Were these deviations likely to have affected the outcome?**		PN
**2.5. If Y/PY/NI to 2.4: Were these deviations from intended intervention balanced between groups?**		NA
**2.6 Was an appropriate analysis used to estimate the effect of assignment to intervention?**	Patients were analyzed per protocol.	N
**2.7 If N/PN/NI to 2.6: Was there potential for a substantial impact (on the result) of the failure to analyse participants in the group to which they were randomized?**	Considering the relatively low number of patients who were excluded after randomization and the fact that this number was approximately balanced between the two groups, it is unlikely that the failure to analyze participants in the group to which they were randomized substantially impacted on the results.	PN
**Risk-of-bias judgement**		Some concerns
Optional: What is the predicted direction of bias due to deviations from intended interventions?		NA / Favours experimental / Favours comparator / Towards null /Away from null / Unpredictable

Domain 3: Missing outcome data

Signalling questions	Comments	Response options
**3.1 Were data for this outcome available for all, or nearly all, participants randomized?**	Data were available for all included patients.	Y
**3.2 If N/PN/NI to 3.1: Is there evidence that the result was not biased by missing outcome data?**		NA
**3.3 If N/PN to 3.2: Could missingness in the outcome depend on its true value?**		NA
**3.4 If Y/PY/NI to 3.3: Is it likely that missingness in the outcome depended on its true value?**		NA
**Risk-of-bias judgement**		Low
Optional: What is the predicted direction of bias due to missing outcome data?		NA / Favours experimental / Favours comparator / Towards null /Away from null / Unpredictable

Domain 4: Risk of bias in measurement of the outcome

Signalling questions	Comments	Response options
**4.1 Was the method of measuring the outcome inappropriate?**		N
**4.2 Could measurement or ascertainment of the outcome have differed between intervention groups?**	Although there is not explicit mention that concealment was maintained throughout outcomes evaluation and outcome assessment was performed by investigators who were not involved in patient care, it is unlikely that the measurement or ascertainment of the outcome differed between intervention groups.	PN
**4.3 If N/PN/NI to 4.1 and 4.2: Were outcome assessors aware of the intervention received by study participants?**	There is not explicit mention that concealment was maintained throughout outcomes evaluation and outcome assessment was performed by investigators who were not involved in patient care.	PY
**4.4 If Y/PY/NI to 4.3: Could assessment of the outcome have been influenced by knowledge of intervention received?**	Although there is not explicit mention that concealment was maintained throughout outcomes evaluation and outcome assessment was performed by investigators who were not involved in patient care, it is unlikely that the measurement or ascertainment of the outcome differed between intervention groups, considering that re-intubation is an outcome that does not involve any judgement.	PN
**4.5 If Y/PY/NI to 4.4: Is it likely that assessment of the outcome was influenced by knowledge of intervention received?**		NA
**Risk-of-bias judgement**		Low
Optional: What is the predicted direction of bias in measurement of the outcome?		NA / Favours experimental / Favours comparator / Towards null /Away from null / Unpredictable

Domain 5: Risk of bias in selection of the reported result

Signalling questions	Comments	Response options
**5.1 Were the data that produced this result analysed in accordance with a pre-specified analysis plan that was finalized before unblinded outcome data were available for analysis?**	Adherence to the protocol registered in ClinicalTrial.gov.	Y
**Is the numerical result being assessed likely to have been selected, on the basis of the results, from...**		
**5.2. ... multiple eligible outcome measurements (e.g. scales, definitions, time points) within the outcome domain?**		N
**5.3 ... multiple eligible analyses of the data?**		N
**Risk-of-bias judgement**		Low
Optional: What is the predicted direction of bias due to selection of the reported result?		NA / Favours experimental / Favours comparator / Towards null /Away from null / Unpredictable

Overall risk of bias

**Risk-of-bias judgement**		Some concerns
Optional: What is the overall predicted direction of bias for this outcome?		NA / Favours experimental / Favours comparator / Towards null /Away from null / Unpredictable

This work is licensed under a Creative Commons Attribution-NonCommercial-NoDerivatives 4.0 International License.

Revised Cochrane risk-of-bias tool for randomized trials (RoB 2)
TEMPLATE FOR COMPLETION

Edited by Julian PT Higgins, Jelena Savović, Matthew J Page, Jonathan AC Sterne
on behalf of the RoB2 Development Group

Version of 22 August 2019

The development of the RoB 2 tool was supported by the MRC Network of Hubs for Trials Methodology Research (MR/L004933/2- N61), with the support of the host MRC ConDuCT-II Hub (Collaboration and innovation for Difficult and Complex randomised controlled Trials In Invasive procedures - MR/K025643/1), by MRC research grant MR/M025209/1, and by a grant from The Cochrane Collaboration.

This work is licensed under a Creative Commons Attribution-NonCommercial-NoDerivatives 4.0 International License.

Study details

**Reference**	Squadrone V, Coha M, Cerutti E, Schellino MM, Biolino P, Occella P, Belloni G, Vilianis G, Fiore G, Cavallo F, Ranieri VM; Piedmont Intensive Care Units Network (PICUN). Continuous positive airway pressure for treatment of postoperative hypoxemia: a randomized controlled trial. JAMA. 2005 Feb 2;293(5):589-95. doi: 10.1001/jama.293.5.589. PMID: 15687314.

Study design

X Individually-randomized parallel-group trial
☐ Cluster-randomized parallel-group trial
☐ Individually randomized cross-over (or other matched) trial

For the purposes of this assessment, the interventions being compared are defined as

Experimental: CPAP   Comparator: COT

**Specify which outcome is being assessed for risk of bias**	Re-intubation
**Specify the numerical result being assessed.** In case of multiple alternative analyses being presented, specify the numeric result (e.g. RR = 1.52 (95% CI 0.83 to 2.77) and/or a reference (e.g. to a table, figure or paragraph) that uniquely defines the result being assessed.	10/104 patients in the COT group, and 1/105 patient in the CPAP group (p = 0.005).

Is the review team's aim for this result…?

X to assess the effect of *assignment to intervention* (the 'intention-to-treat' effect)
☐ to assess the effect of *adhering to intervention* (the 'per-protocol' effect)

If the aim is to assess the effect of *adhering to intervention*, select the deviations from intended intervention that should be addressed (at least one must be checked):

☐ occurrence of non-protocol interventions
☐ failures in implementing the intervention that could have affected the outcome
☐ non-adherence to their assigned intervention by trial participants

Which of the following sources were <u>obtained</u> to help inform the risk-of-bias assessment? (tick as many as apply)

X Journal article(s) with results of the trial
☐ Trial protocol
☐ Statistical analysis plan (SAP)
☐ Non-commercial trial registry record (e.g. ClinicalTrials.gov record)
☐ Company-owned trial registry record (e.g. GSK Clinical Study Register record)
☐ “Grey literature” (e.g. unpublished thesis)
☐ Conference abstract(s) about the trial
☐ Regulatory document (e.g. Clinical Study Report, Drug Approval Package)
☐ Research ethics application
☐ Grant database summary (e.g. NIH RePORTER or Research Councils UK Gateway to Research)
☐ Personal communication with trialist
☐ Personal communication with the sponsor

Risk of bias assessment

Responses underlined in green are potential markers for low risk of bias, and responses in red are potential markers for a risk of bias. Where questions relate only to sign posts to other questions, no formatting is used.

Domain 1: Risk of bias arising from the randomization process

Signalling questions	Comments	Response options
**1.1 Was the allocation sequence random?**	Citation: "Concealed randomization was conducted centrally through a dedicated Web site using a computer-generated block randomization schedule".	Y
**1.2 Was the allocation sequence concealed until participants were enrolled and assigned to interventions?**		Y
**1.3 Did baseline differences between intervention groups suggest a problem with the randomization process?**	No baseline differences between intervention groups were reported.	N
**Risk-of-bias judgement**		Low
Optional: What is the predicted direction of bias arising from the randomization process?		NA / Favours experimental / Favours comparator / Towards null /Away from null / Unpredictable

Domain 2: Risk of bias due to deviations from the intended interventions (*effect of assignment to intervention*)

Signalling questions	Comments	Response options
**2.1. Were participants aware of their assigned intervention during the trial?**	Citation: "Allocation to treatment with oxygen or oxygen plus CPAP was not blinded".	Y
**2.2. Were carers and people delivering the interventions aware of participants' assigned intervention during the trial?**		Y
**2.3. If Y/PY/NI to 2.1 or 2.2: Were there deviations from the intended intervention that arose because of the trial context?**	Eleven patients out of the 21 patients excluded from the 230 total eligible patients were excluded because of lack of bed availability in the ICU, which was not among the exclusion criteria.	Y
**2.4 If Y/PY to 2.3: Were these deviations likely to have affected the outcome?**	The exclusion of 11 patients because of lack of bed availability in the ICU occurred before randomization, the number of patients excluded is low and likely balanced between the two study groups.	PN
**2.5. If Y/PY/NI to 2.4: Were these deviations from intended intervention balanced between groups?**		NA
**2.6 Was an appropriate analysis used to estimate the effect of assignment to intervention?**	Citation: "All analyses were conducted on an intention-to-treat basis".	Y
**2.7 If N/PN/NI to 2.6: Was there potential for a substantial impact (on the result) of the failure to analyse participants in the group to which they were randomized?**		NA
**Risk-of-bias judgement**		Some concerns
Optional: What is the predicted direction of bias due to deviations from intended interventions?		NA / Favours experimental / Favours comparator / Towards null /Away from null / Unpredictable

Domain 3: Missing outcome data

Signalling questions	Comments	Response options
**3.1 Were data for this outcome available for all, or nearly all, participants randomized?**		Y
**3.2 If N/PN/NI to 3.1: Is there evidence that the result was not biased by missing outcome data?**		NA
**3.3 If N/PN to 3.2: Could missingness in the outcome depend on its true value?**		NA
**3.4 If Y/PY/NI to 3.3: Is it likely that missingness in the outcome depended on its true value?**		NA
**Risk-of-bias judgement**		Low
Optional: What is the predicted direction of bias due to missing outcome data?		NA / Favours experimental / Favours comparator / Towards null /Away from null / Unpredictable

Domain 4: Risk of bias in measurement of the outcome

Signalling questions	Comments	Response options
**4.1 Was the method of measuring the outcome inappropriate?**		N
**4.2 Could measurement or ascertainment of the outcome have differed between intervention groups?**	Citation: "Allocation to treatment with oxygen or oxygen plus CPAP was not blinded.".	PN
**4.3 If N/PN/NI to 4.1 and 4.2: Were outcome assessors aware of the intervention received by study participants?**	Citations: "Allocation to treatment with oxygen or oxygen plus CPAP was not blinded."; "To minimize potential bias in the assessment of some of the study end points, we used measures such as objective criteria for endotracheal intubation and standardization of all cointerventions that could have influenced outcome variables such as anesthesia, postoperative pain control, and respiratory physiotherapy".	N
**4.4 If Y/PY/NI to 4.3: Could assessment of the outcome have been influenced by knowledge of intervention received?**		NA
**4.5 If Y/PY/NI to 4.4: Is it likely that assessment of the outcome was influenced by knowledge of intervention received?**		NA
**Risk-of-bias judgement**		Low
Optional: What is the predicted direction of bias in measurement of the outcome?		NA / Favours experimental / Favours comparator / Towards null /Away from null / Unpredictable

Domain 5: Risk of bias in selection of the reported result

Signalling questions	Comments	Response options
**5.1 Were the data that produced this result analysed in accordance with a pre-specified analysis plan that was finalized before unblinded outcome data were available for analysis?**	There is no mention of any pre-specified analysis plan or clinical trial registration.	NI
**Is the numerical result being assessed likely to have been selected, on the basis of the results, from...**		
**5.2. ... multiple eligible outcome measurements (e.g. scales, definitions, time points) within the outcome domain?**		N
**5.3 ... multiple eligible analyses of the data?**		N
**Risk-of-bias judgement**		Some concerns
Optional: What is the predicted direction of bias due to selection of the reported result?		NA / Favours experimental / Favours comparator / Towards null /Away from null / Unpredictable

Overall risk of bias

**Risk-of-bias judgement**		Some concerns
Optional: What is the overall predicted direction of bias for this outcome?		NA / Favours experimental / Favours comparator / Towards null /Away from null / Unpredictable

This work is licensed under a Creative Commons Attribution-NonCommercial-NoDerivatives 4.0 International License.

Revised Cochrane risk-of-bias tool for randomized trials (RoB 2)
TEMPLATE FOR COMPLETION

Edited by Julian PT Higgins, Jelena Savović, Matthew J Page, Jonathan AC Sterne
on behalf of the RoB2 Development Group

Version of 22 August 2019

The development of the RoB 2 tool was supported by the MRC Network of Hubs for Trials Methodology Research (MR/L004933/2- N61), with the support of the host MRC ConDuCT-II Hub (Collaboration and innovation for Difficult and Complex randomised controlled Trials In Invasive procedures - MR/K025643/1), by MRC research grant MR/M025209/1, and by a grant from The Cochrane Collaboration.

This work is licensed under a Creative Commons Attribution-NonCommercial-NoDerivatives 4.0 International License.

Study details

**Reference**	Stéphan F, Barrucand B, Petit P, Rézaiguia-Delclaux S, Médard A, Delannoy B, Cosserant B, Flicoteaux G, Imbert A, Pilorge C, Bérard L; BiPOP Study Group. High-Flow Nasal Oxygen vs Noninvasive Positive Airway Pressure in Hypoxemic Patients After Cardiothoracic Surgery: A Randomized Clinical Trial. JAMA. 2015 Jun 16;313(23):2331-9. doi: 10.1001/jama.2015.5213. PMID: 25980660.

Study design

- X Individually-randomized parallel-group trial
- ☐ Cluster-randomized parallel-group trial
- ☐ Individually randomized cross-over (or other matched) trial

For the purposes of this assessment, the interventions being compared are defined as

Experimental:	HFNO	Comparator:	NIV

**Specify which outcome is being assessed for risk of bias**	Re-intubation
**Specify the numerical result being assessed.** In case of multiple alternative analyses being presented, specify the numeric result (e.g. RR = 1.52 (95% CI 0.83 to 2.77) and/or a reference (e.g. to a table, figure or paragraph) that uniquely defines the result being assessed.	57/416 patients with NIV and 58/414 patients with HFNO (p = 0.99)

Is the review team's aim for this result…?

- X to assess the effect of *assignment to intervention* (the 'intention-to-treat' effect)
- ☐ to assess the effect of *adhering to intervention* (the 'per-protocol' effect)

If the aim is to assess the effect of *adhering to intervention*, select the deviations from intended intervention that should be addressed (at least one must be checked):

- ☐ occurrence of non-protocol interventions
- ☐ failures in implementing the intervention that could have affected the outcome
- ☐ non-adherence to their assigned intervention by trial participants

Which of the following sources were <u>obtained</u> to help inform the risk-of-bias assessment? (tick as many as apply)

X Journal article(s) with results of the trial
X Trial protocol
☐ Statistical analysis plan (SAP)
X Non-commercial trial registry record (e.g. ClinicalTrials.gov record)
☐ Company-owned trial registry record (e.g. GSK Clinical Study Register record)
☐ "Grey literature" (e.g. unpublished thesis)
☐ Conference abstract(s) about the trial
☐ Regulatory document (e.g. Clinical Study Report, Drug Approval Package)
☐ Research ethics application
☐ Grant database summary (e.g. NIH RePORTER or Research Councils UK Gateway to Research)
☐ Personal communication with trialist
☐ Personal communication with the sponsor

Risk of bias assessment

Responses underlined in green are potential markers for low risk of bias, and responses in red are potential markers for a risk of bias. Where questions relate only to sign posts to other questions, no formatting is used.

Domain 1: Risk of bias arising from the randomization process

Signalling questions	Comments	Response options
**1.1 Was the allocation sequence random?**	Citation: "Randomization was conducted in blocks of 2 or 4, regardless of entry criteria, with opaque envelopes, with a single computer-generated (nQuery Advisor) random-number sequence for all centers. Attending physicians randomly assigned patients in a 1:1 ratio to one of the 2 groups"	Y
**1.2 Was the allocation sequence concealed until participants were enrolled and assigned to interventions?**		Y
**1.3 Did baseline differences between intervention groups suggest a problem with the randomization process?**	Citation: "Baseline characteristics were similar in the 2 groups".	N
**Risk-of-bias judgement**		Low
Optional: What is the predicted direction of bias arising from the randomization process?		NA / Favours experimental / Favours comparator / Towards null /Away from null / Unpredictable

Domain 2: Risk of bias due to deviations from the intended interventions (*effect of assignment to intervention*)

Signalling questions	Comments	Response options
**2.1. Were participants aware of their assigned intervention during the trial?**	Citation: "although we applied predefined criteria for reintubation or complications, bias cannot be completely ruled out because blinding was not feasible."	Y
**2.2. Were carers and people delivering the interventions aware of participants' assigned intervention during the trial?**		Y
**2.3. If Y/PY/NI to 2.1 or 2.2: Were there deviations from the intended intervention that arose because of the trial context?**	Citation: "Switching to the other study treatment occurred for 33 patients with BiPAP (7.9%; 95% CI, 5.6%-11.0%) and 45 with high-flow nasal oxygen therapy (10.8%; 95% CI, 8.5%-14.9%) (P = .15). Premature discontinuation was noted for 15 patients with BiPAP (3.6%; 95% CI, 2.1%-6.0%) and 6 with high-flow nasal oxygen therapy (1.4%; 95% CI, 0.6%-3.3%) (P = .04)" Switching to the other study treatment and treatment premature discontinuation were allowed by the study protocol. Switching to the other study treatment was allowed by the study protocol and accounted for treatment failure (primary outcome).	PN
**2.4 If Y/PY to 2.3: Were these deviations likely to have affected the outcome?**		NA
**2.5. If Y/PY/NI to 2.4: Were these deviations from intended intervention balanced between groups?**		NA
**2.6 Was an appropriate analysis used to estimate the effect of assignment to intervention?**	Citation: "All analyses were performed on an intention-to-treat basis".	Y
**2.7 If N/PN/NI to 2.6: Was there potential for a substantial impact (on the result) of the failure to analyse participants in the group to which they were randomized?**		NA
**Risk-of-bias judgement**		Low
Optional: What is the predicted direction of bias due to deviations from intended interventions?		NA / Favours experimental / Favours comparator / Towards null /Away from null / Unpredictable

Domain 3: Missing outcome data

Signalling questions	Comments	Response options
**3.1 Were data for this outcome available for all, or nearly all, participants randomized?**		Y
**3.2 If N/PN/NI to 3.1: Is there evidence that the result was not biased by missing outcome data?**		NA
**3.3 If N/PN to 3.2: Could missingness in the outcome depend on its true value?**		NA
**3.4 If Y/PY/NI to 3.3: Is it likely that missingness in the outcome depended on its true value?**		NA
**Risk-of-bias judgement**		Low
Optional: What is the predicted direction of bias due to missing outcome data?		NA / Favours experimental / Favours comparator / Towards null /Away from null / Unpredictable

Domain 4: Risk of bias in measurement of the outcome

Signalling questions	Comments	Response options
**4.1 Was the method of measuring the outcome inappropriate?**		N
**4.2 Could measurement or ascertainment of the outcome have differed between intervention groups?**	Although there is not explicit mention that concealment was maintained throughout outcomes evaluation and outcome assessment was performed by investigators who were not involved in patient care, re-intubation is an objectifiable outcome. Therefore, it is not likely that the ascertainment of the outcome differed between intervention group	PN
**4.3 If N/PN/NI to 4.1 and 4.2: Were outcome assessors aware of the intervention received by study participants?**	There is not explicit mention that concealment was maintained throughout outcomes evaluation and outcome assessment was performed by investigators who were not involved in patient care.	PY
**4.4 If Y/PY/NI to 4.3: Could assessment of the outcome have been influenced by knowledge of intervention received?**	Re-intubation is an outcome that does not involve any judgement and it is unlikely that outcome assessors were influenced by knowledge of intervention received by study participants.	PN
**4.5 If Y/PY/NI to 4.4: Is it likely that assessment of the outcome was influenced by knowledge of intervention received?**		NA
**Risk-of-bias judgement**		Low
Optional: What is the predicted direction of bias in measurement of the outcome?		NA / Favours experimental / Favours comparator / Towards null /Away from null / Unpredictable

Domain 5: Risk of bias in selection of the reported result

Signalling questions	Comments	Response options
**5.1 Were the data that produced this result analysed in accordance with a pre-specified analysis plan that was finalized before unblinded outcome data were available for analysis?**	Adherence to protocol registered in ClinicalTrial.gov.	Y
**Is the numerical result being assessed likely to have been selected, on the basis of the results, from...**		
**5.2. ... multiple eligible outcome measurements (e.g. scales, definitions, time points) within the outcome domain?**		N
**5.3 ... multiple eligible analyses of the data?**		N
**Risk-of-bias judgement**		Low
Optional: What is the predicted direction of bias due to selection of the reported result?		NA / Favours experimental / Favours comparator / Towards null /Away from null / Unpredictable

Overall risk of bias

**Risk-of-bias judgement**		Low
Optional: What is the overall predicted direction of bias for this outcome?		NA / Favours experimental / Favours comparator / Towards null /Away from null / Unpredictable

This work is licensed under a Creative Commons Attribution-NonCommercial-NoDerivatives 4.0 International License.

Revised Cochrane risk-of-bias tool for randomized trials (RoB 2)
TEMPLATE FOR COMPLETION

Edited by Julian PT Higgins, Jelena Savović, Matthew J Page, Jonathan AC Sterne
on behalf of the RoB2 Development Group

Version of 22 August 2019

The development of the RoB 2 tool was supported by the MRC Network of Hubs for Trials Methodology Research (MR/L004933/2- N61), with the support of the host MRC ConDuCT-II Hub (Collaboration and innovation for Difficult and Complex randomised controlled Trials In Invasive procedures - MR/K025643/1), by MRC research grant MR/M025209/1, and by a grant from The Cochrane Collaboration.

This work is licensed under a Creative Commons Attribution-NonCommercial-NoDerivatives 4.0 International License.

Study details

**Reference**	Theologou S, Ischaki E, Zakynthinos SG, Charitos C, Michopanou N, Patsatzis S, Mentzelopoulos SD. High Flow Oxygen Therapy at Two Initial Flow Settings versus Conventional Oxygen Therapy in Cardiac Surgery Patients with Postextubation Hypoxemia: A Single-Center, Unblinded, Randomized, Controlled Trial. J Clin Med. 2021 May 12;10(10):2079. doi: 10.3390/jcm10102079. PMID: 34066244; PMCID: PMC8151420.

Study design

X Individually-randomized parallel-group trial
☐ Cluster-randomized parallel-group trial
☐ Individually randomized cross-over (or other matched) trial

For the purposes of this assessment, the interventions being compared are defined as

Experimental:	HFNO at two different flow rates (60 and 40 L/min)	Comparator:	COT

**Specify which outcome is being assessed for risk of bias**	Reintubation
**Specify the numerical result being assessed.** In case of multiple alternative analyses being presented, specify the numeric result (e.g. RR = 1.52 (95% CI 0.83 to 2.77) and/or a reference (e.g. to a table, figure or paragraph) that uniquely defines the result being assessed.	2/33 patients in the HFNO 60 L/min group vs. 2/33 patients in the COT group (p > 0.99)<br>5/33 patients in the HFNO 60 L/min group vs. 2/33 patients in the COT (p = 0.43)

Is the review team's aim for this result…?

X to assess the effect of *assignment to intervention* (the 'intention-to-treat' effect)
☐ to assess the effect of *adhering to intervention* (the 'per-protocol' effect)

If the aim is to assess the effect of *adhering to intervention*, select the deviations from intended intervention that should be addressed (at least one must be checked):

☐ occurrence of non-protocol interventions
☐ failures in implementing the intervention that could have affected the outcome
☐ non-adherence to their assigned intervention by trial participants

Which of the following sources were obtained to help inform the risk-of-bias assessment? (tick as many as apply)

X Journal article(s) with results of the trial
☐ Trial protocol
X Statistical analysis plan (SAP)
X Non-commercial trial registry record (e.g. ClinicalTrials.gov record)
☐ Company-owned trial registry record (e.g. GSK Clinical Study Register record)
☐ "Grey literature" (e.g. unpublished thesis)
☐ Conference abstract(s) about the trial
☐ Regulatory document (e.g. Clinical Study Report, Drug Approval Package)
☐ Research ethics application
☐ Grant database summary (e.g. NIH RePORTER or Research Councils UK Gateway to Research)
☐ Personal communication with trialist
☐ Personal communication with the sponsor

Risk of bias assessment

Responses underlined in green are potential markers for low risk of bias, and responses in red are potential markers for a risk of bias. Where questions relate only to sign posts to other questions, no formatting is used.

Domain 1: Risk of bias arising from the randomization process

Signalling questions	Comments	Response options
**1.1 Was the allocation sequence random?**	Citation: "Following extubation, patients were randomly assigned to intervention groups 1 or 2 or to control group at a ratio of 1:1:1. Blocks of 3 numbers were consecutively drawn from a sequence of 99 unique random numbers (range, 1–99) generated using Research Randomizer version 4.0 (www.randomizer.org, accessed on 2 May 2017). Randomization was performed by the study statistician, who applied the following group allocation rule: the smallest and largest number of each block was assigned to intervention groups 1 and 2, respectively, and the remaining number to control. Upon patient enrollment, attending investigators received an SMS text message containing the patient's code number and group."	Y
**1.2 Was the allocation sequence concealed until participants were enrolled and assigned to interventions?**		PY
**1.3 Did baseline differences between intervention groups suggest a problem with the randomization process?**	Citation: "Patient baseline characteristics were similar".	N
**Risk-of-bias judgement**	Low	Low
Optional: What is the predicted direction of bias arising from the randomization process?		NA / Favours experimental / Favours comparator / Towards null /Away from null / Unpredictable

Domain 2: Risk of bias due to deviations from the intended interventions (*effect of assignment to intervention*)

Signalling questions	Comments	Response options
**2.1. Were participants aware of their assigned intervention during the trial?**	Citation: "We conducted a prospective, unblinded RCT".	Y
**2.2. Were carers and people delivering the interventions aware of participants' assigned intervention during the trial?**		Y
**2.3. If Y/PY/NI to 2.1 or 2.2: Were there deviations from the intended intervention that arose because of the trial context?**	No deviations from the intended intervention are reported.	PN
**2.4 If Y/PY to 2.3: Were these deviations likely to have affected the outcome?**		NA
**2.5. If Y/PY/NI to 2.4: Were these deviations from intended intervention balanced between groups?**		NA
**2.6 Was an appropriate analysis used to estimate the effect of assignment to intervention?**	Citation: "All analyses were performed according to the intention-to-treat principle".	Y
**2.7 If N/PN/NI to 2.6: Was there potential for a substantial impact (on the result) of the failure to analyse participants in the group to which they were randomized?**		NA
**Risk-of-bias judgement**	Low	Low
Optional: What is the predicted direction of bias due to deviations from intended interventions?		NA / Favours experimental / Favours comparator / Towards null /Away from null / Unpredictable

Domain 3: Missing outcome data

Signalling questions	Comments	Response options
**3.1 Were data for this outcome available for all, or nearly all, participants randomized?**		Y
**3.2 If N/PN/NI to 3.1: Is there evidence that the result was not biased by missing outcome data?**		NA
**3.3 If N/PN to 3.2: Could missingness in the outcome depend on its true value?**		NA
**3.4 If Y/PY/NI to 3.3: Is it likely that missingness in the outcome depended on its true value?**		NA
**Risk-of-bias judgement**	Low	Low
Optional: What is the predicted direction of bias due to missing outcome data?		NA / Favours experimental / Favours comparator / Towards null /Away from null / Unpredictable

Domain 4: Risk of bias in measurement of the outcome

Signalling questions	Comments	Response options
**4.1 Was the method of measuring the outcome inappropriate?**	The description of the methods is detailed enough to exclude the method of measurement of the outcome is inappropriate.	N
**4.2 Could measurement or ascertainment of the outcome have differed between intervention groups?**		N
**4.3 If N/PN/NI to 4.1 and 4.2: Were outcome assessors aware of the intervention received by study participants?**	The study in unblinded. Therefore, it is very likely that outcome assessors were aware of the intervention received by study participants.	PY
**4.4 If Y/PY/NI to 4.3: Could assessment of the outcome have been influenced by knowledge of intervention received?**	Although the assessment of the outcome could have been influenced by the knowledge of the intervention received by the patients, this is unlikely to be the case, considering the objective nature of the data investigated to determine the primary outcome and the overall good quality of the study methodology.	PY
**4.5 If Y/PY/NI to 4.4: Is it likely that assessment of the outcome was influenced by knowledge of intervention received?**		PN
**Risk-of-bias judgement**		Some concerns
Optional: What is the predicted direction of bias in measurement of the outcome?		NA / Favours experimental / Favours comparator / Towards null /Away from null / Unpredictable

Domain 5: Risk of bias in selection of the reported result

Signalling questions	Comments	Response options
**5.1 Were the data that produced this result analysed in accordance with a pre-specified analysis plan that was finalized before unblinded outcome data were available for analysis?**	Citation: "The study was registered 21 days prior to the enrollment of the first patient at clinicaltrials.gov (NCT03282552, registration date 14 September 2017. Principal Investigator: Stavros Theologou)." The study was conducted according to the pre-specified analysis plan detailed in the clinical trial registration.	Y
**Is the numerical result being assessed likely to have been selected, on the basis of the results, from...**		
**5.2. ... multiple eligible outcome measurements (e.g. scales, definitions, time points) within the outcome domain?**		N
**5.3 ... multiple eligible analyses of the data?**		N
**Risk-of-bias judgement**		Low / High / Some concerns
Optional: What is the predicted direction of bias due to selection of the reported result?		NA / Favours experimental / Favours comparator / Towards null /Away from null / Unpredictable

Overall risk of bias

**Risk-of-bias judgement**		Some concerns
Optional: What is the overall predicted direction of bias for this outcome?		NA / Favours experimental / Favours comparator / Towards null /Away from null / Unpredictable

This work is licensed under a Creative Commons Attribution-NonCommercial-NoDerivatives 4.0 International License.

Revised Cochrane risk-of-bias tool for randomized trials (RoB 2)
TEMPLATE FOR COMPLETION

Edited by Julian PT Higgins, Jelena Savović, Matthew J Page, Jonathan AC Sterne
on behalf of the RoB2 Development Group

Version of 22 August 2019

The development of the RoB 2 tool was supported by the MRC Network of Hubs for Trials Methodology Research (MR/L004933/2- N61), with the support of the host MRC ConDuCT-II Hub (Collaboration and innovation for Difficult and Complex randomised controlled Trials In Invasive procedures - MR/K025643/1), by MRC research grant MR/M025209/1, and by a grant from The Cochrane Collaboration.

This work is licensed under a Creative Commons Attribution-NonCommercial-NoDerivatives 4.0 International License.

Study details

**Reference**	Vourc'h M, Nicolet J, Volteau C, Caubert L, Chabbert C, Lepoivre T, Senage T, Roussel JC, Rozec B. High-Flow Therapy by Nasal Cannulae Versus High-Flow Face Mask in Severe Hypoxemia After Cardiac Surgery: A Single-Center Randomized Controlled Study-The HEART FLOW Study. J Cardiothorac Vasc Anesth. 2020 Jan;34(1):157-165. doi: 10.1053/j.jvca.2019.05.039. Epub 2019 May 29. PMID: 31230964.

Study design

X Individually-randomized parallel-group trial

☐ Cluster-randomized parallel-group trial

☐ Individually randomized cross-over (or other matched) trial

For the purposes of this assessment, the interventions being compared are defined as

Experimental:	HFNO	Comparator:	COT

**Specify which outcome is being assessed for risk of bias**	Re-intubation
**Specify the numerical result being assessed.** In case of multiple alternative analyses being presented, specify the numeric result (e.g. RR = 1.52 (95% CI 0.83 to 2.77) and/or a reference (e.g. to a table, figure or paragraph) that uniquely defines the result being assessed.	1/43 patient in the COT group vs. 3/47 patients in the HFNO group (p = 0.75)

Is the review team's aim for this result…?

× to assess the effect of *assignment to intervention* (the 'intention-to-treat' effect)

☐ to assess the effect of *adhering to intervention* (the 'per-protocol' effect)

If the aim is to assess the effect of *adhering to intervention*, select the deviations from intended intervention that should be addressed (at least one must be checked):

☐ occurrence of non-protocol interventions
☐ failures in implementing the intervention that could have affected the outcome
☐ non-adherence to their assigned intervention by trial participants

Which of the following sources were <u>obtained</u> to help inform the risk-of-bias assessment? (tick as many as apply)

✕ Journal article(s) with results of the trial
✕ Trial protocol
☐ Statistical analysis plan (SAP)
☐ Non-commercial trial registry record (e.g. ClinicalTrials.gov record)
☐ Company-owned trial registry record (e.g. GSK Clinical Study Register record)
☐ “Grey literature” (e.g. unpublished thesis)
☐ Conference abstract(s) about the trial
☐ Regulatory document (e.g. Clinical Study Report, Drug Approval Package)
☐ Research ethics application
☐ Grant database summary (e.g. NIH RePORTER or Research Councils UK Gateway to Research)
☐ Personal communication with trialist
☐ Personal communication with the sponsor

Risk of bias assessment

Responses underlined in green are potential markers for low risk of bias, and responses in red are potential markers for a risk of bias. Where questions relate only to sign posts to other questions, no formatting is used.

Domain 1: Risk of bias arising from the randomization process

Signalling questions	Comments	Response options
**1.1 Was the allocation sequence random?**	Citation: "Randomization used fixed blocks (ratio 1:1). The study statistician generated the allocation list."	Y
**1.2 Was the allocation sequence concealed until participants were enrolled and assigned to interventions?**	Citation: "Allocation was performed with opaque envelopes with a single computer-generated random-number sequence controlled by the independent research promotion unit at the University Hospital of Nantes."	Y
**1.3 Did baseline differences between intervention groups suggest a problem with the randomization process?**		N
**Risk-of-bias judgement**		Low
Optional: What is the predicted direction of bias arising from the randomization process?		NA / Favours experimental / Favours comparator / Towards null /Away from null / Unpredictable

Domain 2: Risk of bias due to deviations from the intended interventions (*effect of assignment to intervention*)

Signalling questions	Comments	Response options
**2.1. Were participants aware of their assigned intervention during the trial?**	The interventions involved the use of a specifical device for oxygenation in awake patients, so both patients and healthcare providers were likely aware of the interventions.<br><br>Citations: "Blinded devices for oxygen therapy would have strengthened the results, but that was difficult to achieve in 48 hours".	Y
**2.2. Were carers and people delivering the interventions aware of participants' assigned intervention during the trial?**		Y
**2.3. If Y/PY/NI to 2.1 or 2.2: Were there deviations from the intended intervention that arose because of the trial context?**	There is no evidence that major deviations from the intended intervention arose because of the trial context.	PN
**2.4 If Y/PY to 2.3: Were these deviations likely to have affected the outcome?**		NA
**2.5. If Y/PY/NI to 2.4: Were these deviations from intended intervention balanced between groups?**		NA
**2.6 Was an appropriate analysis used to estimate the effect of assignment to intervention?**	Citation: "An intent-to-treat analysis was conducted excluding patients with withdrawal of consent".	Y
**2.7 If N/PN/NI to 2.6: Was there potential for a substantial impact (on the result) of the failure to analyse participants in the group to which they were randomized?**		NA
**Risk-of-bias judgement**		Low
Optional: What is the predicted direction of bias due to deviations from intended interventions?		NA / Favours experimental / Favours comparator / Towards null /Away from null / Unpredictable

Domain 3: Missing outcome data

Signalling questions	Comments	Response options
**3.1 Were data for this outcome available for all, or nearly all, participants randomized?**		Y
**3.2 If N/PN/NI to 3.1: Is there evidence that the result was not biased by missing outcome data?**		NA
**3.3 If N/PN to 3.2: Could missingness in the outcome depend on its true value?**		NA
**3.4 If Y/PY/NI to 3.3: Is it likely that missingness in the outcome depended on its true value?**		NA
**Risk-of-bias judgement**		Low
Optional: What is the predicted direction of bias due to missing outcome data?		NA / Favours experimental / Favours comparator / Towards null /Away from null / Unpredictable

Domain 4: Risk of bias in measurement of the outcome

Signalling questions	Comments	Response options
**4.1 Was the method of measuring the outcome inappropriate?**		N
**4.2 Could measurement or ascertainment of the outcome have differed between intervention groups?**	Citation: "The follow-up team and the physician who interpreted the chest X-rays were blinded to the randomization group".	PN
**4.3 If N/PN/NI to 4.1 and 4.2: Were outcome assessors aware of the intervention received by study participants?**	Citation: "The follow-up team and the physician who interpreted the chest X-rays were blinded to the randomization group".	PN
**4.4 If Y/PY/NI to 4.3: Could assessment of the outcome have been influenced by knowledge of intervention received?**		NA
**4.5 If Y/PY/NI to 4.4: Is it likely that assessment of the outcome was influenced by knowledge of intervention received?**		NA
**Risk-of-bias judgement**		Low
Optional: What is the predicted direction of bias in measurement of the outcome?		NA / Favours experimental / Favours comparator / Towards null /Away from null / Unpredictable

Domain 5: Risk of bias in selection of the reported result

Signalling questions	Comments	Response options
**5.1 Were the data that produced this result analysed in accordance with a pre-specified analysis plan that was finalized before unblinded outcome data were available for analysis?**	Adherence to protocol registered in ClinicalTrial.gov	Y
**Is the numerical result being assessed likely to have been selected, on the basis of the results, from...**		
**5.2. ... multiple eligible outcome measurements (e.g. scales, definitions, time points) within the outcome domain?**		N
**5.3 ... multiple eligible analyses of the data?**		N
**Risk-of-bias judgement**		Low
Optional: What is the predicted direction of bias due to selection of the reported result?		NA / Favours experimental / Favours comparator / Towards null /Away from null / Unpredictable

Overall risk of bias

**Risk-of-bias judgement**		Low
Optional: What is the overall predicted direction of bias for this outcome?		NA / Favours experimental / Favours comparator / Towards null /Away from null / Unpredictable

This work is licensed under a Creative Commons Attribution-NonCommercial-NoDerivatives 4.0 International License.

Revised Cochrane risk-of-bias tool for randomized trials (RoB 2)
TEMPLATE FOR COMPLETION

Edited by Julian PT Higgins, Jelena Savović, Matthew J Page, Jonathan AC Sterne
on behalf of the RoB2 Development Group

Version of 22 August 2019

The development of the RoB 2 tool was supported by the MRC Network of Hubs for Trials Methodology Research (MR/L004933/2- N61), with the support of the host MRC ConDuCT-II Hub (Collaboration and innovation for Difficult and Complex randomised controlled Trials In Invasive procedures - MR/K025643/1), by MRC research grant MR/M025209/1, and by a grant from The Cochrane Collaboration.

This work is licensed under a Creative Commons Attribution-NonCommercial-NoDerivatives 4.0 International License.

Study details

Reference | Wong DT, Adly E, Ip HY, Thapar S, Maxted GR, Chung FF. A comparison between the Boussignac™ continuous positive airway pressure mask and the venturi mask in terms of improvement in the PaO2/F(I)O2 ratio in morbidly obese patients undergoing bariatric surgery: a randomized controlled trial. Can J Anaesth. 2011 Jun;58(6):532-9. doi: 10.1007/s12630-011-9497-3. Epub 2011 Apr 5. PMID: 21465320.

Study design

X Individually-randomized parallel-group trial
☐ Cluster-randomized parallel-group trial
☐ Individually randomized cross-over (or other matched) trial

For the purposes of this assessment, the interventions being compared are defined as

Experimental: CPAP | Comparator: COT

Specify which outcome is being assessed for risk of bias | Re-intubation

Specify the numerical result being assessed. In case of multiple alternative analyses being presented, specify the numeric result (e.g. RR = 1.52 (95% CI 0.83 to 2.77) and/or a reference (e.g. to a table, figure or paragraph) that uniquely defines the result being assessed. | 0/38 patients in the COT group vs. 0/43 patients in the CPAP group.

Is the review team's aim for this result…?

X to assess the effect of *assignment to intervention* (the 'intention-to-treat' effect)
☐ to assess the effect of *adhering to intervention* (the 'per-protocol' effect)

If the aim is to assess the effect of *adhering to intervention*, select the deviations from intended intervention that should be addressed (at least one must be checked):

☐ occurrence of non-protocol interventions
☐ failures in implementing the intervention that could have affected the outcome
☐ non-adherence to their assigned intervention by trial participants

Which of the following sources were <u>obtained</u> to help inform the risk-of-bias assessment? (tick as many as apply)

X Journal article(s) with results of the trial
☐ Trial protocol
☐ Statistical analysis plan (SAP)
☐ Non-commercial trial registry record (e.g. ClinicalTrials.gov record)
☐ Company-owned trial registry record (e.g. GSK Clinical Study Register record)
☐ “Grey literature” (e.g. unpublished thesis)
☐ Conference abstract(s) about the trial
☐ Regulatory document (e.g. Clinical Study Report, Drug Approval Package)
☐ Research ethics application
☐ Grant database summary (e.g. NIH RePORTER or Research Councils UK Gateway to Research)
☐ Personal communication with trialist
☐ Personal communication with the sponsor

Risk of bias assessment

Responses underlined in green are potential markers for low risk of bias, and responses in red are potential markers for a risk of bias. Where questions relate only to sign posts to other questions, no formatting is used.

Domain 1: Risk of bias arising from the randomization process

Signalling questions	Comments	Response options
**1.1 Was the allocation sequence random?**	Citation: “Patients were assigned randomly to receive either the Boussignac (Boussignac Group) or the venturi face mask (Venturi Group) immediately after tracheal extubation”.	Y
**1.2 Was the allocation sequence concealed until participants were enrolled and assigned to interventions?**	Citation: “Randomization of patients was done by the research coordinator using a list of computer-generated numbers, and the group assignments were concealed in opaque sealed envelopes”; “At the end of surgery, the sealed randomization envelope was opened, and the research coordinator allocated the patient either to the Boussignac Group or the Venturi Group”.	Y
**1.3 Did baseline differences between intervention groups suggest a problem with the randomization process?**	Citation: “The baseline demographic variables were comparable between groups”	N
**Risk-of-bias judgement**		Low
Optional: What is the predicted direction of bias arising from the randomization process?		NA / Favours experimental / Favours comparator / Towards null /Away from null / Unpredictable

Domain 2: Risk of bias due to deviations from the intended interventions (*effect of assignment to intervention*)

Signalling questions	Comments	Response options
**2.1. Were participants aware of their assigned intervention during the trial?**	It is likely that patients and caregivers could not be blinded to the treatment allocation.	PY
**2.2. Were carers and people delivering the interventions aware of participants' assigned intervention during the trial?**		PY
**2.3. If Y/PY/NI to 2.1 or 2.2: Were there deviations from the intended intervention that arose because of the trial context?**	Three patients in the CPAP group e six in the COT group did not receive the allocated intervention because of the failure to obtain intraoperative arterial blood gas analysis.	Y
**2.4 If Y/PY to 2.3: Were these deviations likely to have affected the outcome?**	It is unlikely that the deviations from intended interventions affect the outcome.	PN
**2.5. If Y/PY/NI to 2.4: Were these deviations from intended intervention balanced between groups?**		NA
**2.6 Was an appropriate analysis used to estimate the effect of assignment to intervention?**	Citation: "Patients with persistent discomfort were administered O2 through nasal prongs at 6 Lxmin-1 and analyzed according to the intention-to-treat principle. Similarly, patients in the Venturi Group who found the venturi mask intolerable received O2 via nasal prongs at 6 Lxmin-1, and their data were analyzed according to the intention- to-treat principle."	Y
**2.7 If N/PN/NI to 2.6: Was there potential for a substantial impact (on the result) of**		NA

**the failure to analyse participants in the group to which they were randomized?**		
**Risk-of-bias judgement**		Some concerns
Optional: What is the predicted direction of bias due to deviations from intended interventions?		NA / Favours experimental / Favours comparator / Towards null /Away from null / Unpredictable

Domain 3: Missing outcome data

Signalling questions	Comments	Response options
**3.1 Were data for this outcome available for all, or nearly all, participants randomized?**	Citation: "A total of 90 subjects were enrolled in the study. Nine subjects were excluded from data analysis either due to the inability to obtain arterial sampling intraoperatively, or one or two hours post extubation or due to patient refusal for arterial sampling".	Y
**3.2 If N/PN/NI to 3.1: Is there evidence that the result was not biased by missing outcome data?**		NA
**3.3 If N/PN to 3.2: Could missingness in the outcome depend on its true value?**		NA
**3.4 If Y/PY/NI to 3.3: Is it likely that missingness in the outcome depended on its true value?**		NA
**Risk-of-bias judgement**		Low
Optional: What is the predicted direction of bias due to missing outcome data?		NA / Favours experimental / Favours comparator / Towards null /Away from null / Unpredictable

Domain 4: Risk of bias in measurement of the outcome

Signalling questions	Comments	Response options
**4.1 Was the method of measuring the outcome inappropriate?**		N
**4.2 Could measurement or ascertainment of the outcome have differed between intervention groups?**	There is not explicit mention that concealment was maintained throughout outcomes evaluation and outcome assessment was performed by investigators who were not involved in patient care.	PN
**4.3 If N/PN/NI to 4.1 and 4.2: Were outcome assessors aware of the intervention received by study participants?**	There is not explicit mention that concealment was maintained throughout outcomes evaluation and outcome assessment was performed by investigators who were not involved in patient care.	PY
**4.4 If Y/PY/NI to 4.3: Could assessment of the outcome have been influenced by knowledge of intervention received?**	Although there is not explicit mention that concealment was maintained throughout outcomes evaluation and outcome assessment was performed by investigators who were not involved in patient care, it is unlikely that the measurement or ascertainment of the outcome differed between intervention groups, considering that re-intubation is an outcome that does not involve any judgement.	PN
**4.5 If Y/PY/NI to 4.4: Is it likely that assessment of the outcome was influenced by knowledge of intervention received?**		NA
**Risk-of-bias judgement**		Low
Optional: What is the predicted direction of bias in measurement of the outcome?		NA / Favours experimental / Favours comparator / Towards null /Away from null / Unpredictable

Domain 5: Risk of bias in selection of the reported result

Signalling questions	Comments	Response options
**5.1 Were the data that produced this result analysed in accordance with a pre-specified analysis plan that was finalized before unblinded outcome data were available for analysis?**	No mention of any pre-specified analysis plan	NI
**Is the numerical result being assessed likely to have been selected, on the basis of the results, from...**		
**5.2. ... multiple eligible outcome measurements (e.g. scales, definitions, time points) within the outcome domain?**		N
**5.3 ... multiple eligible analyses of the data?**		N
**Risk-of-bias judgement**		Low
Optional: What is the predicted direction of bias due to selection of the reported result?		NA / Favours experimental / Favours comparator / Towards null /Away from null / Unpredictable

Overall risk of bias

**Risk-of-bias judgement**		Some concerns
Optional: What is the overall predicted direction of bias for this outcome?		NA / Favours experimental / Favours comparator / Towards null /Away from null / Unpredictable

This work is licensed under a Creative Commons Attribution-NonCommercial-NoDerivatives 4.0 International License.

Revised Cochrane risk-of-bias tool for randomized trials (RoB 2)
TEMPLATE FOR COMPLETION

Edited by Julian PT Higgins, Jelena Savović, Matthew J Page, Jonathan AC Sterne
on behalf of the RoB2 Development Group

Version of 22 August 2019

The development of the RoB 2 tool was supported by the MRC Network of Hubs for Trials Methodology Research (MR/L004933/2- N61), with the support of the host MRC ConDuCT-II Hub (Collaboration and innovation for Difficult and Complex randomised controlled Trials In Invasive procedures - MR/K025643/1), by MRC research grant MR/M025209/1, and by a grant from The Cochrane Collaboration.

This work is licensed under a Creative Commons Attribution-NonCommercial-NoDerivatives 4.0 International License.

Study details

**Reference**	Yang Y, Liu N, Sun L, Zhou Y, Yang Y, Shang W, Li X. Noninvasive Positive-Pressure Ventilation in Treatment of Hypoxemia After Extubation Following Type-A Aortic Dissection. J Cardiothorac Vasc Anesth. 2016 Dec;30(6):1539-1544. doi: 10.1053/j.jvca.2016.03.129. Epub 2016 Mar 10. PMID: 27495963.

Study design

X Individually-randomized parallel-group trial

☐ Cluster-randomized parallel-group trial

☐ Individually randomized cross-over (or other matched) trial

For the purposes of this assessment, the interventions being compared are defined as

Experimental: NIV Comparator: COT

**Specify which outcome is being assessed for risk of bias**	Re-intubation
**Specify the numerical result being assessed.** In case of multiple alternative analyses being presented, specify the numeric result (e.g. RR = 1.52 (95% CI 0.83 to 2.77) and/or a reference (e.g. to a table, figure or paragraph) that uniquely defines the result being assessed.	9/25 patients in the COT group vs. 8/25 patients in the NIV mask group vs. 2/25 patients in the NIV Helmet group ($p < 0.05$ vs. COT and $p < 0.05$ vs. NIV mask mask)

Is the review team's aim for this result…?

x to assess the effect of *assignment to intervention* (the 'intention-to-treat' effect)

☐ to assess the effect of *adhering to intervention* (the 'per-protocol' effect)

If the aim is to assess the effect of *adhering to intervention*, select the deviations from intended intervention that should be addressed (at least one must be checked):

☐ occurrence of non-protocol interventions
☐ failures in implementing the intervention that could have affected the outcome
☐ non-adherence to their assigned intervention by trial participants

Which of the following sources were <u>obtained</u> to help inform the risk-of-bias assessment? (tick as many as apply)

x Journal article(s) with results of the trial
☐ Trial protocol
☐ Statistical analysis plan (SAP)
☐ Non-commercial trial registry record (e.g. ClinicalTrials.gov record)
☐ Company-owned trial registry record (e.g. GSK Clinical Study Register record)
☐ “Grey literature” (e.g. unpublished thesis)
☐ Conference abstract(s) about the trial
☐ Regulatory document (e.g. Clinical Study Report, Drug Approval Package)
☐ Research ethics application
☐ Grant database summary (e.g. NIH RePORTER or Research Councils UK Gateway to Research)
☐ Personal communication with trialist
☐ Personal communication with the sponsor

Risk of bias assessment

Responses underlined in green are potential markers for low risk of bias, and responses in red are potential markers for a risk of bias. Where questions relate only to sign posts to other questions, no formatting is used.

Domain 1: Risk of bias arising from the randomization process

Signalling questions	Comments	Response options
**1.1 Was the allocation sequence random?**	Citation: "Simple randomization to 1 of the 3 treatment groups was used. Patients were matched to 75 colored balls—red (n = 25), yellow (n = 25), or blue (n = 25)—that were mixed well in a container before they were drawn for group assignment. Blinded to the balls in the container, 1 ball was drawn at a time by the researcher, with patients matched with the red balls being assigned to the Venturi mask group, patients matched with the yellow balls to the mask NPPV group, and patients matched with the blue balls to the helmet NPPV group."	Y
**1.2 Was the allocation sequence concealed until participants were enrolled and assigned to interventions?**		PY
**1.3 Did baseline differences between intervention groups suggest a problem with the randomization process?**	Citation: "All 3 groups were comparable in terms of age; sex; body mass index; comorbidities; renal, lung, and heart functions; type of surgery; and causes of hypoxemia."	PN
**Risk-of-bias judgement**		Low
Optional: What is the predicted direction of bias arising from the randomization process?		NA / Favours experimental / Favours comparator / Towards null /Away from null / Unpredictable

Domain 2: Risk of bias due to deviations from the intended interventions (*effect of assignment to intervention*)

Signalling questions	Comments	Response options
**2.1. Were participants aware of their assigned intervention during the trial?**	It is likely that patients and caregivers could not be blinded to the treatment allocation.	Y
**2.2. Were carers and people delivering the interventions aware of participants' assigned intervention during the trial?**		Y
**2.3. If Y/PY/NI to 2.1 or 2.2: Were there deviations from the intended intervention that arose because of the trial context?**	There is no mention of deviations from the intended intervention.	PN
**2.4 If Y/PY to 2.3: Were these deviations likely to have affected the outcome?**		NA
**2.5. If Y/PY/NI to 2.4: Were these deviations from intended intervention balanced between groups?**		NA
**2.6 Was an appropriate analysis used to estimate the effect of assignment to intervention?**		NI
**2.7 If N/PN/NI to 2.6: Was there potential for a substantial impact (on the result) of the failure to analyse participants in the group to which they were randomized?**	All participants were apparently analyzed in the group to which they were randomized.	PN
**Risk-of-bias judgement**		Some concerns
Optional: What is the predicted direction of bias due to deviations from intended interventions?		NA / Favours experimental / Favours comparator / Towards null /Away from null / Unpredictable

Domain 3: Missing outcome data

Signalling questions	Comments	Response options
**3.1 Were data for this outcome available for all, or nearly all, participants randomized?**		PY
**3.2 If N/PN/NI to 3.1: Is there evidence that the result was not biased by missing outcome data?**		NA
**3.3 If N/PN to 3.2: Could missingness in the outcome depend on its true value?**		NA
**3.4 If Y/PY/NI to 3.3: Is it likely that missingness in the outcome depended on its true value?**		NA
**Risk-of-bias judgement**		Low
Optional: What is the predicted direction of bias due to missing outcome data?		NA / Favours experimental / Favours comparator / Towards null /Away from null / Unpredictable

Domain 4: Risk of bias in measurement of the outcome

Signalling questions	Comments	Response options
**4.1 Was the method of measuring the outcome inappropriate?**		N
**4.2 Could measurement or ascertainment of the outcome have differed between intervention groups?**	Although there is not explicit mention that concealment was maintained throughout outcomes evaluation and outcome assessment was performed by investigators who were not involved in patient care, it is unlikely that the measurement or ascertainment of the outcome differed between intervention groups.	PN
**4.3 If N/PN/NI to 4.1 and 4.2: Were outcome assessors aware of the intervention received by study participants?**	There is not explicit mention that outcome assessors were blinded to the intervention received by study participants.	Y
**4.4 If Y/PY/NI to 4.3: Could assessment of the outcome have been influenced by knowledge of intervention received?**	Although there is not explicit mention that concealment was maintained throughout outcomes evaluation and outcome assessment was performed by investigators who were not involved in patient care, it is unlikely that the measurement or ascertainment of the outcome differed between intervention groups, considering that re-intubation is an outcome that does not involve any judgement.	PN
**4.5 If Y/PY/NI to 4.4: Is it likely that assessment of the outcome was influenced by knowledge of intervention received?**		NA
**Risk-of-bias judgement**		Low
Optional: What is the predicted direction of bias in measurement of the outcome?		NA / Favours experimental / Favours comparator / Towards null /Away from null / Unpredictable

Domain 5: Risk of bias in selection of the reported result

Signalling questions	Comments	Response options
**5.1 Were the data that produced this result analysed in accordance with a pre-specified analysis plan that was finalized before unblinded outcome data were available for analysis?**	There is no mention of any trial registration or prespecified statistical analysis plan.	NI
**Is the numerical result being assessed likely to have been selected, on the basis of the results, from...**		
**5.2. ... multiple eligible outcome measurements (e.g. scales, definitions, time points) within the outcome domain?**		N
**5.3 ... multiple eligible analyses of the data?**		N
**Risk-of-bias judgement**		Some concerns
Optional: What is the predicted direction of bias due to selection of the reported result?		NA / Favours experimental / Favours comparator / Towards null /Away from null / Unpredictable

Overall risk of bias

**Risk-of-bias judgement**		Some concerns
Optional: What is the overall predicted direction of bias for this outcome?		NA / Favours experimental / Favours comparator / Towards null /Away from null / Unpredictable

This work is licensed under a Creative Commons Attribution-NonCommercial-NoDerivatives 4.0 International License.

Revised Cochrane risk-of-bias tool for randomized trials (RoB 2)
TEMPLATE FOR COMPLETION

Edited by Julian PT Higgins, Jelena Savović, Matthew J Page, Jonathan AC Sterne
on behalf of the RoB2 Development Group

Version of 22 August 2019

The development of the RoB 2 tool was supported by the MRC Network of Hubs for Trials Methodology Research (MR/L004933/2- N61), with the support of the host MRC ConDuCT-II Hub (Collaboration and innovation for Difficult and Complex randomised controlled Trials In Invasive procedures - MR/K025643/1), by MRC research grant MR/M025209/1, and by a grant from The Cochrane Collaboration.

This work is licensed under a Creative Commons Attribution-NonCommercial-NoDerivatives 4.0 International License.

Study details

**Reference**	Yu Y, Qian X, Liu C, Zhu C. Effect of High-Flow Nasal Cannula versus Conventional Oxygen Therapy for Patients with Thoracoscopic Lobectomy after Extubation. Can Respir J. 2017;2017:7894631. doi: 10.1155/2017/7894631. Epub 2017 Feb 19. PMID: 28298878; PMCID: PMC5337350.

Study design

- X Individually-randomized parallel-group trial
- ☐ Cluster-randomized parallel-group trial
- ☐ Individually randomized cross-over (or other matched) trial

For the purposes of this assessment, the interventions being compared are defined as

Experimental: HFNO Comparator: COT

Specify which outcome is being assessed for risk of bias: Re-intubation

Specify the numerical result being assessed. In case of multiple alternative analyses being presented, specify the numeric result (e.g. RR = 1.52 (95% CI 0.83 to 2.77) and/or a reference (e.g. to a table, figure or paragraph) that uniquely defines the result being assessed.

0/56 patients in the HFNO group vs. 5/54 in the COT group (p = 0.026)

Is the review team's aim for this result…?

- X to assess the effect of *assignment to intervention* (the 'intention-to-treat' effect)
- ☐ to assess the effect of *adhering to intervention* (the 'per-protocol' effect)

If the aim is to assess the effect of *adhering to intervention*, select the deviations from intended intervention that should be addressed (at least one must be checked):

- ☐ occurrence of non-protocol interventions
- ☐ failures in implementing the intervention that could have affected the outcome
- ☐ non-adherence to their assigned intervention by trial participants

Which of the following sources were <u>obtained</u> to help inform the risk-of-bias assessment? (tick as many as apply)

X	Journal article(s) with results of the trial
☐	Trial protocol
☐	Statistical analysis plan (SAP)
☐	Non-commercial trial registry record (e.g. ClinicalTrials.gov record)
☐	Company-owned trial registry record (e.g. GSK Clinical Study Register record)
☐	“Grey literature” (e.g. unpublished thesis)
☐	Conference abstract(s) about the trial
☐	Regulatory document (e.g. Clinical Study Report, Drug Approval Package)
☐	Research ethics application
☐	Grant database summary (e.g. NIH RePORTER or Research Councils UK Gateway to Research)
☐	Personal communication with trialist
☐	Personal communication with the sponsor

Risk of bias assessment

Responses underlined in green are potential markers for low risk of bias, and responses in red are potential markers for a risk of bias. Where questions relate only to sign posts to other questions, no formatting is used.

Domain 1: Risk of bias arising from the randomization process

Signalling questions	Comments	Response options
**1.1 Was the allocation sequence random?**	Citation: "Patients were classified into two groups by random figure table following extubation. A random number sequence was generated with STATA statistical software version 12.1"	Y
**1.2 Was the allocation sequence concealed until participants were enrolled and assigned to interventions?**	No mention regarding the allocation sequence concealment.	NI
**1.3 Did baseline differences between intervention groups suggest a problem with the randomization process?**	There are not apparent imbalances between groups, beyond that expected by chance. Citation: "There were no significant differences between patients in two groups in all aspects".	N
**Risk-of-bias judgement**		Some concerns
Optional: What is the predicted direction of bias arising from the randomization process?		NA / Favours experimental / Favours comparator / Towards null /Away from null / Unpredictable

Domain 2: Risk of bias due to deviations from the intended interventions (*effect of assignment to intervention*)

Signalling questions	Comments	Response options
**2.1. Were participants aware of their assigned intervention during the trial?**	The interventions involve the use of a specifical device for oxygenation in awake patients, so both patients and healthcare providers are aware of the interventions. Citation: "The study was unblinded".	Y
**2.2. Were carers and people delivering the interventions aware of participants' assigned intervention during the trial?**		Y
**2.3. If Y/PY/NI to 2.1 or 2.2: Were there deviations from the intended intervention that arose because of the trial context?**	There were no deviations from the intended intervention.	N
**2.4 If Y/PY to 2.3: Were these deviations likely to have affected the outcome?**		NA
**2.5. If Y/PY/NI to 2.4: Were these deviations from intended intervention balanced between groups?**		NA
**2.6 Was an appropriate analysis used to estimate the effect of assignment to intervention?**	Citation: "All analyses were performed on an intention-to-treat basis."	Y
**2.7 If N/PN/NI to 2.6: Was there potential for a substantial impact (on the result) of the failure to analyse participants in the group to which they were randomized?**		NA
**Risk-of-bias judgement**		Low
Optional: What is the predicted direction of bias due to deviations from intended interventions?		NA / Favours experimental / Favours comparator / Towards null /Away from null / Unpredictable

Domain 3: Missing outcome data

Signalling questions	Comments	Response options
**3.1 Were data for this outcome available for all, or nearly all, participants randomized?**	Citation: "All patients included were followed until discharge home".	Y
**3.2 If N/PN/NI to 3.1: Is there evidence that the result was not biased by missing outcome data?**		NA
**3.3 If N/PN to 3.2: Could missingness in the outcome depend on its true value?**		NA
**3.4 If Y/PY/NI to 3.3: Is it likely that missingness in the outcome depended on its true value?**		NA
**Risk-of-bias judgement**		Low
Optional: What is the predicted direction of bias due to missing outcome data?		NA / Favours experimental / Favours comparator / Towards null /Away from null / Unpredictable

Domain 4: Risk of bias in measurement of the outcome

Signalling questions	Comments	Response options
**4.1 Was the method of measuring the outcome inappropriate?**		N
**4.2 Could measurement or ascertainment of the outcome have differed between intervention groups?**	Although there is not explicit mention that concealment was maintained throughout outcomes evaluation and outcome assessment was performed by investigators who were not involved in patient care, it is unlikely that the measurement or ascertainment of the outcome differed between intervention groups.	N
**4.3 If N/PN/NI to 4.1 and 4.2: Were outcome assessors aware of the intervention received by study participants?**	Citation: "The study was unblinded".	Y
**4.4 If Y/PY/NI to 4.3: Could assessment of the outcome have been influenced by knowledge of intervention received?**	Although there is not explicit mention that concealment was maintained throughout outcomes evaluation and outcome assessment was performed by investigators who were not involved in patient care, it is unlikely that the measurement or ascertainment of the outcome differed between intervention groups, considering that re-intubation is an outcome that does not involve any judgement.	PN
**4.5 If Y/PY/NI to 4.4: Is it likely that assessment of the outcome was influenced by knowledge of intervention received?**		NA
**Risk-of-bias judgement**		Low
Optional: What is the predicted direction of bias in measurement of the outcome?		NA / Favours experimental / Favours comparator / Towards null /Away from null / Unpredictable

Domain 5: Risk of bias in selection of the reported result

Signalling questions	Comments	Response options
**5.1 Were the data that produced this result analysed in accordance with a pre-specified analysis plan that was finalized before unblinded outcome data were available for analysis?**	Citation: "One month before the start of this study, a standardized weaning protocol and statistics procedure were set by 9 investigators from these centers after 2 days' learning and discussion. All of the centers carefully followed this procedure during the study time."<br>However, the prespecified analysis plan was not published.	PY
**Is the numerical result being assessed likely to have been selected, on the basis of the results, from...**		
**5.2. ... multiple eligible outcome measurements (e.g. scales, definitions, time points) within the outcome domain?**	Reintubation is a dichotomous outcome.	N
**5.3 ... multiple eligible analyses of the data?**		N
**Risk-of-bias judgement**		Low
Optional: What is the predicted direction of bias due to selection of the reported result?		NA / Favours experimental / Favours comparator / Towards null /Away from null / Unpredictable

Overall risk of bias

**Risk-of-bias judgement**		Some concerns
Optional: What is the overall predicted direction of bias for this outcome?		NA / Favours experimental / Favours comparator / Towards null /Away from null / Unpredictable

This work is licensed under a Creative Commons Attribution-NonCommercial-NoDerivatives 4.0 International License.

Revised Cochrane risk-of-bias tool for randomized trials (RoB 2)
TEMPLATE FOR COMPLETION

Edited by Julian PT Higgins, Jelena Savović, Matthew J Page, Jonathan AC Sterne
on behalf of the RoB2 Development Group

Version of 22 August 2019

The development of the RoB 2 tool was supported by the MRC Network of Hubs for Trials Methodology Research (MR/L004933/2- N61), with the support of the host MRC ConDuCT-II Hub (Collaboration and innovation for Difficult and Complex randomised controlled Trials In Invasive procedures - MR/K025643/1), by MRC research grant MR/M025209/1, and by a grant from The Cochrane Collaboration.

This work is licensed under a Creative Commons Attribution-NonCommercial-NoDerivatives 4.0 International License.

Study details

**Reference**	Zarbock A, Mueller E, Netzer S, Gabriel A, Feindt P, Kindgen-Milles D. Prophylactic nasal continuous positive airway pressure following cardiac surgery protects from postoperative pulmonary complications: a prospective, randomized, controlled trial in 500 patients. Chest. 2009 May;135(5):1252-1259. doi: 10.1378/chest.08-1602. Epub 2008 Nov 18. PMID: 19017864.

Study design

- X Individually-randomized parallel-group trial
- ☐ Cluster-randomized parallel-group trial
- ☐ Individually randomized cross-over (or other matched) trial

For the purposes of this assessment, the interventions being compared are defined as

Experimental: CPAP Comparator: COT

**Specify which outcome is being assessed for risk of bias**	Re-intubation
**Specify the numerical result being assessed.** In case of multiple alternative analyses being presented, specify the numeric result (e.g. RR = 1.52 (95% CI 0.83 to 2.77) and/or a reference (e.g. to a table, figure or paragraph) that uniquely defines the result being assessed.	6/236 patients in the COT group vs. 3/232 patients in the CPAP group

Is the review team's aim for this result…?

- X to assess the effect of *assignment to intervention* (the 'intention-to-treat' effect)
- ☐ to assess the effect of *adhering to intervention* (the 'per-protocol' effect)

If the aim is to assess the effect of *adhering to intervention*, select the deviations from intended intervention that should be addressed (at least one must be checked):

- ☐ occurrence of non-protocol interventions
- ☐ failures in implementing the intervention that could have affected the outcome
- ☐ non-adherence to their assigned intervention by trial participants

Which of the following sources were obtained to help inform the risk-of-bias assessment? (tick as many as apply)

- X Journal article(s) with results of the trial
- ☐ Trial protocol
- ☐ Statistical analysis plan (SAP)
- ☐ Non-commercial trial registry record (e.g. ClinicalTrials.gov record)
- ☐ Company-owned trial registry record (e.g. GSK Clinical Study Register record)
- ☐ “Grey literature” (e.g. unpublished thesis)
- ☐ Conference abstract(s) about the trial
- ☐ Regulatory document (e.g. Clinical Study Report, Drug Approval Package)
- ☐ Research ethics application
- ☐ Grant database summary (e.g. NIH RePORTER or Research Councils UK Gateway to Research)
- ☐ Personal communication with trialist
- ☐ Personal communication with the sponsor

Risk of bias assessment

Responses underlined in green are potential markers for low risk of bias, and responses in red are potential markers for a risk of bias. Where questions relate only to sign posts to other questions, no formatting is used.

Domain 1: Risk of bias arising from the randomization process

Signalling questions	Comments	Response options
**1.1 Was the allocation sequence random?**	Citation: "Following arrival on the ICU, concealed randomization was conducted using a randomization list".	Y
**1.2 Was the allocation sequence concealed until participants were enrolled and assigned to interventions?**		PY
**1.3 Did baseline differences between intervention groups suggest a problem with the randomization process?**	Citation: "There were no statistical differences regarding demographic and surgical procedure data between the control and study groups".	N
**Risk-of-bias judgement**		Low
Optional: What is the predicted direction of bias arising from the randomization process?		NA / Favours experimental / Favours comparator / Towards null /Away from null / Unpredictable

Domain 2: Risk of bias due to deviations from the intended interventions (*effect of assignment to intervention*)

Signalling questions	Comments	Response options
**2.1. Were participants aware of their assigned intervention during the trial?**	Although the decision of transferring patients to a general ward was conducted by blinded physicians, the interventions involve the use of a specifical device for oxygenation in awake patients, so both patients and healthcare providers are aware of the interventions.	Y
**2.2. Were carers and people delivering the interventions aware of participants' assigned intervention during the trial?**		Y
**2.3. If Y/PY/NI to 2.1 or 2.2: Were there deviations from the intended intervention that arose because of the trial context?**	There were no deviations from the intended intervention.	N
**2.4 If Y/PY to 2.3: Were these deviations likely to have affected the outcome?**		NA
**2.5. If Y/PY/NI to 2.4: Were these deviations from intended intervention balanced between groups?**		NA
**2.6 Was an appropriate analysis used to estimate the effect of assignment to intervention?**	Citation: "All analyses were conducted on an intention-to-treat basis".	Y
**2.7 If N/PN/NI to 2.6: Was there potential for a substantial impact (on the result) of the failure to analyse participants in the group to which they were randomized?**		NA
**Risk-of-bias judgement**		Low
Optional: What is the predicted direction of bias due to deviations from intended interventions?		NA / Favours experimental / Favours comparator / Towards null /Away from null / Unpredictable

Domain 3: Missing outcome data

Signalling questions	Comments	Response options
**3.1 Were data for this outcome available for all, or nearly all, participants randomized?**		Y
**3.2 If N/PN/NI to 3.1: Is there evidence that the result was not biased by missing outcome data?**		NA
**3.3 If N/PN to 3.2: Could missingness in the outcome depend on its true value?**		NA
**3.4 If Y/PY/NI to 3.3: Is it likely that missingness in the outcome depended on its true value?**		NA
**Risk-of-bias judgement**		Low
Optional: What is the predicted direction of bias due to missing outcome data?		NA / Favours experimental / Favours comparator / Towards null /Away from null / Unpredictable

Domain 4: Risk of bias in measurement of the outcome

Signalling questions	Comments	Response options
**4.1 Was the method of measuring the outcome inappropriate?**		N
**4.2 Could measurement or ascertainment of the outcome have differed between intervention groups?**	Although there is not explicit mention that concealment was maintained throughout outcomes evaluation and outcome assessment was performed by investigators who were not involved in patient care, it is unlikely that the measurement or ascertainment of the outcome differed between intervention groups.	N
**4.3 If N/PN/NI to 4.1 and 4.2: Were outcome assessors aware of the intervention received by study participants?**	there is not explicit mention that concealment was maintained throughout outcomes evaluation and outcome assessment was performed by investigators who were not involved in patient care.	Y
**4.4 If Y/PY/NI to 4.3: Could assessment of the outcome have been influenced by knowledge of intervention received?**	Although there is not explicit mention that concealment was maintained throughout outcomes evaluation and outcome assessment was performed by investigators who were not involved in patient care, it is unlikely that the measurement or ascertainment of the outcome differed between intervention groups, considering that re-intubation is an outcome that does not involve any judgement.	PN
**4.5 If Y/PY/NI to 4.4: Is it likely that assessment of the outcome was influenced by knowledge of intervention received?**		NA
**Risk-of-bias judgement**		Low
Optional: What is the predicted direction of bias in measurement of the outcome?		NA / Favours experimental / Favours comparator / Towards null /Away from null / Unpredictable

Domain 5: Risk of bias in selection of the reported result

Signalling questions	Comments	Response options
**5.1 Were the data that produced this result analysed in accordance with a pre-specified analysis plan that was finalized before unblinded outcome data were available for analysis?**	There is no mention that the prespecified analysis plan was published.	NI
**Is the numerical result being assessed likely to have been selected, on the basis of the results, from...**		
**5.2. ... multiple eligible outcome measurements (e.g. scales, definitions, time points) within the outcome domain?**		N
**5.3 ... multiple eligible analyses of the data?**		N
**Risk-of-bias judgement**		Some concerns
Optional: What is the predicted direction of bias due to selection of the reported result?		NA / Favours experimental / Favours comparator / Towards null /Away from null / Unpredictable

Overall risk of bias

**Risk-of-bias judgement**		Some concerns
Optional: What is the overall predicted direction of bias for this outcome?		NA / Favours experimental / Favours comparator / Towards null /Away from null / Unpredictable

This work is licensed under a Creative Commons Attribution-NonCommercial-NoDerivatives 4.0 International License.

Revised Cochrane risk-of-bias tool for randomized trials (RoB 2)
TEMPLATE FOR COMPLETION

Edited by Julian PT Higgins, Jelena Savović, Matthew J Page, Jonathan AC Sterne
on behalf of the RoB2 Development Group

Version of 22 August 2019

The development of the RoB 2 tool was supported by the MRC Network of Hubs for Trials Methodology Research (MR/L004933/2- N61), with the support of the host MRC ConDuCT-II Hub (Collaboration and innovation for Difficult and Complex randomised controlled Trials In Invasive procedures - MR/K025643/1), by MRC research grant MR/M025209/1, and by a grant from The Cochrane Collaboration.

This work is licensed under a Creative Commons Attribution-NonCommercial-NoDerivatives 4.0 International License.

Study details

Reference

Zhu GF, Wang DJ, Liu S, Jia M, Jia SJ. Efficacy and safety of noninvasive positive pressure ventilation in the treatment of acute respiratory failure after cardiac surgery. Chin Med J (Engl). 2013 Dec;126(23):4463-9. PMID: 24286408.

Study design

X Individually-randomized parallel-group trial

☐ Cluster-randomized parallel-group trial

☐ Individually randomized cross-over (or other matched) trial

For the purposes of this assessment, the interventions being compared are defined as

Experimental: NIV

Comparator: COT

Specify which outcome is being assessed for risk of bias

Re-intubation

Specify the numerical result being assessed. In case of multiple alternative analyses being presented, specify the numeric result (e.g. RR = 1.52 (95% CI 0.83 to 2.77) and/or a reference (e.g. to a table, figure or paragraph) that uniquely defines the result being assessed.

9/48 patients in the NIV group vs. 38/47 patients in the COT group (p = 0.000)

Is the review team's aim for this result…?

x to assess the effect of *assignment to intervention* (the 'intention-to-treat' effect)

☐ to assess the effect of *adhering to intervention* (the 'per-protocol' effect)

If the aim is to assess the effect of *adhering to intervention*, select the deviations from intended intervention that should be addressed (at least one must be checked):

☐ occurrence of non-protocol interventions
☐ failures in implementing the intervention that could have affected the outcome
☐ non-adherence to their assigned intervention by trial participants

Which of the following sources were obtained to help inform the risk-of-bias assessment? (tick as many as apply)

x Journal article(s) with results of the trial
☐ Trial protocol
☐ Statistical analysis plan (SAP)
☐ Non-commercial trial registry record (e.g. ClinicalTrials.gov record)
☐ Company-owned trial registry record (e.g. GSK Clinical Study Register record)
☐ "Grey literature" (e.g. unpublished thesis)
☐ Conference abstract(s) about the trial
☐ Regulatory document (e.g. Clinical Study Report, Drug Approval Package)
☐ Research ethics application
☐ Grant database summary (e.g. NIH RePORTER or Research Councils UK Gateway to Research)
☐ Personal communication with trialist
☐ Personal communication with the sponsor

Risk of bias assessment

Responses underlined in green are potential markers for low risk of bias, and responses in red are potential markers for a risk of bias. Where questions relate only to sign posts to other questions, no formatting is used.

Domain 1: Risk of bias arising from the randomization process

Signalling questions	Comments	Response options
**1.1 Was the allocation sequence random?**	Citation "After considering the inclusion and exclusion criteria, patients were randomly allocated to the NPPV group or the control group thereby dictating the method utilized after extubation".	Y
**1.2 Was the allocation sequence concealed until participants were enrolled and assigned to interventions?**	No information regarding allocation sequence concealment.	NI
**1.3 Did baseline differences between intervention groups suggest a problem with the randomization process?**	Citation: "There were no significant differences between the NPPV group and the control group in patients' age, gender, concomitant diseases, smoking, type of surgery, causes of postoperative ARF, APACHE II scores".	N
**Risk-of-bias judgement**		Some concerns
Optional: What is the predicted direction of bias arising from the randomization process?		NA / Favours experimental / Favours comparator / Towards null /Away from null / Unpredictable

Domain 2: Risk of bias due to deviations from the intended interventions (*effect of assignment to intervention*)

Signalling questions	Comments	Response options
**2.1. Were participants aware of their assigned intervention during the trial?**	The interventions involve the use of a specifical device for oxygenation in awake patients, so both patients and healthcare providers are likely aware of the interventions.	PY
**2.2. Were carers and people delivering the interventions aware of participants' assigned intervention during the trial?**		PY
**2.3. If Y/PY/NI to 2.1 or 2.2: Were there deviations from the intended intervention that arose because of the trial context?**	There were no reported deviations from the intended intervention.	N
**2.4 If Y/PY to 2.3: Were these deviations likely to have affected the outcome?**		NA
**2.5. If Y/PY/NI to 2.4: Were these deviations from intended intervention balanced between groups?**		NA
**2.6 Was an appropriate analysis used to estimate the effect of assignment to intervention?**		NI
**2.7 If N/PN/NI to 2.6: Was there potential for a substantial impact (on the result) of the failure to analyse participants in the group to which they were randomized?**	Citation: "Thus, 95 patients were eligible, 48 were allocated to the NPPV group and 47 to the control group based on a predetermined randomization protocol. All of these patients were included in the analysis".	PN
**Risk-of-bias judgement**		Some concerns
Optional: What is the predicted direction of bias due to deviations from intended interventions?		NA / Favours experimental / Favours comparator / Towards null /Away from null / Unpredictable

Domain 3: Missing outcome data

Signalling questions	Comments	Response options
**3.1 Were data for this outcome available for all, or nearly all, participants randomized?**		Y
**3.2 If N/PN/NI to 3.1: Is there evidence that the result was not biased by missing outcome data?**		NA
**3.3 If N/PN to 3.2: Could missingness in the outcome depend on its true value?**		NA
**3.4 If Y/PY/NI to 3.3: Is it likely that missingness in the outcome depended on its true value?**		NA
**Risk-of-bias judgement**		Low
Optional: What is the predicted direction of bias due to missing outcome data?		NA / Favours experimental / Favours comparator / Towards null /Away from null / Unpredictable

Domain 4: Risk of bias in measurement of the outcome

Signalling questions	Comments	Response options
**4.1 Was the method of measuring the outcome inappropriate?**		N
**4.2 Could measurement or ascertainment of the outcome have differed between intervention groups?**	Although there is not explicit mention that concealment was maintained throughout outcomes evaluation and outcome assessment was performed by investigators who were not involved in patient care, it is unlikely that the measurement or ascertainment of the outcome differed between intervention groups.	N
**4.3 If N/PN/NI to 4.1 and 4.2: Were outcome assessors aware of the intervention received by study participants?**	There is not explicit mention that concealment was maintained throughout outcomes evaluation and outcome assessment was performed by investigators who were not involved in patient care	PY
**4.4 If Y/PY/NI to 4.3: Could assessment of the outcome have been influenced by knowledge of intervention received?**	Although there is not explicit mention that concealment was maintained throughout outcomes evaluation and outcome assessment was performed by investigators who were not involved in patient care, it is unlikely that the measurement or ascertainment of the outcome differed between intervention groups, considering that re-intubation is an outcome that does not involve any judgement.	PN
**4.5 If Y/PY/NI to 4.4: Is it likely that assessment of the outcome was influenced by knowledge of intervention received?**		NA
**Risk-of-bias judgement**		Low
Optional: What is the predicted direction of bias in measurement of the outcome?		NA / Favours experimental / Favours comparator / Towards null /Away from null / Unpredictable

Domain 5: Risk of bias in selection of the reported result

Signalling questions	Comments	Response options
**5.1 Were the data that produced this result analysed in accordance with a pre-specified analysis plan that was finalized before unblinded outcome data were available for analysis?**	No information regarding trial registration and prespecified analysis plan publication.	NI
**Is the numerical result being assessed likely to have been selected, on the basis of the results, from...**		
**5.2. ... multiple eligible outcome measurements (e.g. scales, definitions, time points) within the outcome domain?**		N
**5.3 ... multiple eligible analyses of the data?**		N
**Risk-of-bias judgement**		Some concerns
Optional: What is the predicted direction of bias due to selection of the reported result?		NA / Favours experimental / Favours comparator / Towards null /Away from null / Unpredictable

Overall risk of bias

**Risk-of-bias judgement**		Some concerns
Optional: What is the overall predicted direction of bias for this outcome?		NA / Favours experimental / Favours comparator / Towards null /Away from null / Unpredictable

This work is licensed under a Creative Commons Attribution-NonCommercial-NoDerivatives 4.0 International License.

Supplementary Figure 1. Risk of bias assessment summary for randomized controlled studies

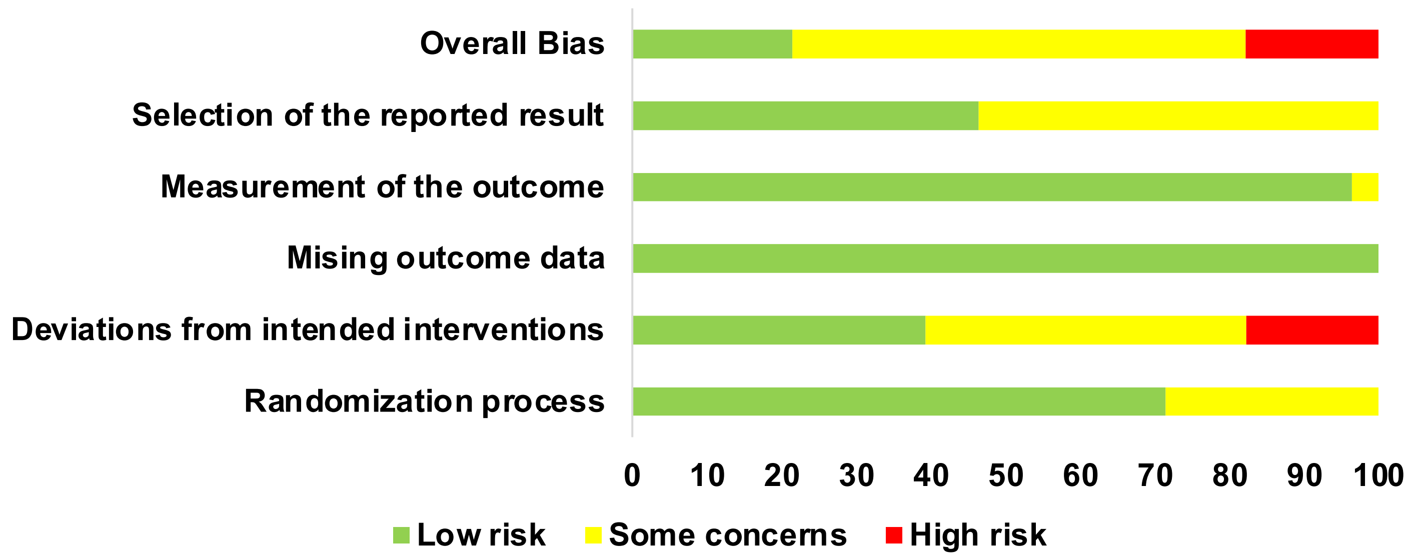

Risk of bias assessments for non-randomized controlled trials

The Risk Of Bias In Non-randomized Studies – of Interventions (ROBINS-I) assessment tool
(version for cohort-type studies)
Version 19 September 2016

This work is licensed under a Creative Commons Attribution-NonCommercial-NoDerivatives 4.0 International License.

Ishikawa 1997

ROBINS-I tool (Stage I): At protocol stage

Specify the review question

Participants	Patients undergoing extubation after surgery and subsequent non-invasive respiratory support
Experimental intervention	One modality of non-invasive respiratory support
Comparator	One or more than one modality of non-invasive respiratory support
Outcomes	Re-intubation rate

List the confounding domains relevant to all or most studies

Type of surgery, clinical setting, preoperative risk of postoperative pulmonary complications

List co-interventions that could be different between intervention groups and that could impact on outcomes

No co-interventions are expected to be different between intervention groups

ROBINS-I tool (Stage II): For each study

Specify a target randomized trial specific to the study

Design	Individually randomized
Participants	Patients undergoing extubation after surgery and subsequent non-invasive respiratory support
Experimental intervention	COT
Comparator	NIV

Is your aim for this study…?

X to assess the effect of *assignment to* intervention
☐ to assess the effect of *starting and adhering to* intervention

Specify the outcome

Specify which outcome is being assessed for risk of bias (typically from among those earmarked for the Summary of Findings table). Specify whether this is a proposed benefit or harm of intervention.

Re-intubation rate

Specify the numerical result being assessed

In case of multiple alternative analyses being presented, specify the numeric result (e.g. RR = 1.52 (95% CI 0.83 to 2.77) and/or a reference (e.g. to a table, figure or paragraph) that uniquely defines the result being assessed.

2/12 patients in the COT group, 0/8 patients in the NIV group.

Preliminary consideration of confounders

Complete a row for each important confounding domain (i) listed in the review protocol; and (ii) relevant to the setting of this particular study, or which the study authors identified as potentially important.

"Important" confounding domains are those for which, in the context of this study, adjustment is expected to lead to a clinically important change in the estimated effect of the intervention. "Validity" refers to whether the confounding variable or variables fully measure the domain, while "reliability" refers to the precision of the measurement (more measurement error means less reliability).

**(i) Confounding domains listed in the review protocol**				
Confounding domain	Measured variable(s)	Is there evidence that controlling for this variable was unnecessary?*	Is the confounding domain measured validly and reliably by this variable (or these variables)?	OPTIONAL: Is failure to adjust for this variable (alone) expected to favour the experimental intervention or the comparator?
Type of surgery	Surgery performed above or below the diaphragm	All patients belong to the same confounding domain. Therefore, adjustment is not necessary	Yes	Not applicable
Clinical setting	Patients admitted to ICU, ward or high-dependency unit	All patients belong to the same confounding domain. Therefore, adjustment is not necessary	Yes	Not applicable
Preoperative risk of postoperative pulmonary complications	High or not-high risk	All patients belong to the same confounding domain. Therefore, adjustment is not necessary	Yes	Not applicable
Baseline characteristics	Age, gender, preoperative ventilation	No	Between-group difference were not tested statistically.	No information

(ii) Additional confounding domains relevant to the setting of this particular study, or which the study authors identified as important				
Confounding domain	Measured variable(s)	Is there evidence that controlling for this variable was unnecessary?*	Is the confounding domain measured validly and reliably by this variable (or these variables)?	OPTIONAL: Is failure to adjust for this variable (alone) expected to favour the experimental intervention or the comparator?
			Yes / No / No information	Favour experimental / Favour comparator / No information
None				

* In the context of a particular study, variables can be demonstrated not to be confounders and so not included in the analysis: (a) if they are not predictive of the outcome; (b) if they are not predictive of intervention; or (c) because adjustment makes no or minimal difference to the estimated effect of the primary parameter. Note that “no statistically significant association” is not the same as “not predictive”.

Preliminary consideration of co-interventions

Complete a row for each important co-intervention (i) listed in the review protocol; and (ii) relevant to the setting of this particular study, or which the study authors identified as important.

"Important" co-interventions are those for which, in the context of this study, adjustment is expected to lead to a clinically important change in the estimated effect of the intervention.

**(i) Co-interventions listed in the review protocol**		
Co-intervention	Is there evidence that controlling for this co-intervention was unnecessary (e.g. because it was not administered)?	Is presence of this co-intervention likely to favour outcomes in the experimental intervention or the comparator
No co-interventions are expected to be different between intervention groups		

**(ii) Additional co-interventions relevant to the setting of this particular study, or which the study authors identified as important**		
Co-intervention	Is there evidence that controlling for this co-intervention was unnecessary (e.g. because it was not administered)?	Is presence of this co-intervention likely to favour outcomes in the experimental intervention or the comparator
No co-interventions are expected to be different between intervention groups		

Risk of bias assessment

Responses underlined in green are potential markers for low risk of bias, and responses in red are potential markers for a risk of bias. Where questions relate only to sign posts to other questions, no formatting is used.

Signalling questions	Description	Response options
**Bias due to confounding**		
1.1 Is there potential for confounding of the effect of intervention in this study?<br>**If N/PN to 1.1:** the study can be considered to be at low risk of bias due to confounding and no further signalling questions need be considered		PY
**If Y/PY to 1.1**: determine whether there is a need to assess time-varying confounding:		
1.2. Was the analysis based on splitting participants' follow up time according to intervention received?<br>**If N/PN**, answer questions relating to baseline confounding (1.4 to 1.6)<br>**If Y/PY**, go to question 1.3.	Participants cannot switch between interventions.	N
1.3. Were intervention discontinuations or switches likely to be related to factors that are prognostic for the outcome?<br>**If N/PN**, answer questions relating to baseline confounding (1.4 to 1.6)<br>**If Y/PY**, answer questions relating to both baseline and time-varying confounding (1.7 and 1.8)		NA

**Questions relating to baseline confounding only**		
1.4. Did the authors use an appropriate analysis method that controlled for all the important confounding domains?	No statistical test was perform to assess any between-group difference in baseline patient characteristics. No appropriate methods to control for measured confounders are used	N
1.5. **If Y/PY to 1.4**: Were confounding domains that were controlled for measured validly and reliably by the variables available in this study?		NA

1.6. Did the authors control for any post-intervention variables that could have been affected by the intervention?		N
**Questions relating to baseline and time-varying confounding**		
1.7. Did the authors use an appropriate analysis method that controlled for all the important confounding domains and for time-varying confounding?	No adjustment for time-varying confounding is used.	N
1.8. **If Y/PY to 1.7**: Were confounding domains that were controlled for measured validly and reliably by the variables available in this study?		NA
**Risk of bias judgement**		Critical
Optional: What is the predicted direction of bias due to confounding?		Unpredictable

**Bias in selection of participants into the study**		
2.1. Was selection of participants into the study (or into the analysis) based on participant characteristics observed after the start of intervention?<br>**If N/PN to 2.1:** go to 2.4	Inclusion criterion (prolonged postoperative respiratory support) is assessed after the initiation of the interventions	Y
2.2. **If Y/PY to 2.1**: Were the post-intervention variables that influenced selection likely to be associated with intervention?	The inclusion criterion (postoperative prolonged respiratory support) is likely to be associated with the interventions.	Y
2.3 **If Y/PY to 2.2**: Were the post-intervention variables that influenced selection likely to be influenced by the outcome or a cause of the outcome?	Patients were intubated after prolonged postoperative support, which was the inclusion criterion in the study.	N
2.4. Do start of follow-up and start of intervention coincide for most participants?		NI
2.5. **If Y/PY to 2.2 and 2.3, or N/PN to 2.4**: Were adjustment techniques used that are likely to correct for the presence of selection biases?		NA
**Risk of bias judgement**		Serious
Optional: What is the predicted direction of bias due to selection of participants into the study?		Unpredictable

Bias in classification of interventions		
3.1 Were intervention groups clearly defined?		Y
3.2 Was the information used to define intervention groups recorded at the start of the intervention?		Y
3.3 Could classification of intervention status have been affected by knowledge of the outcome or risk of the outcome?		N
**Risk of bias judgement**		Low
Optional: What is the predicted direction of bias due to classification of interventions?		Unpredictable

Bias due to deviations from intended interventions		
**If your aim for this study is to assess the effect of assignment to intervention, answer questions 4.1 and 4.2**		
4.1. Were there deviations from the intended intervention beyond what would be expected in usual practice?	No deviations from the intended intervention beyond what would be expected in usual practice are reported.	PN
4.2. **If Y/PY to 4.1**: Were these deviations from intended intervention unbalanced between groups *and* likely to have affected the outcome?		NA
**Risk of bias judgement**		Low
Optional: What is the predicted direction of bias due to deviations from the intended interventions?		Unpredictable

Bias due to missing data		
5.1 Were outcome data available for all, or nearly all, participants?		Y
5.2 Were participants excluded due to missing data on intervention status?		N
5.3 Were participants excluded due to missing data on other variables needed for the analysis?		N
5.4 **If PN/N to 5.1, or Y/PY to 5.2 or 5.3**: Are the proportion of participants and reasons for missing data similar across interventions?		NA

5.5 **If PN/N to 5.1, or Y/PY to 5.2 or 5.3**: Is there evidence that results were robust to the presence of missing data?		NA
**Risk of bias judgement**		Low
Optional: What is the predicted direction of bias due to missing data?		Unpredictable

**Bias in measurement of outcomes**		
6.1 Could the outcome measure have been influenced by knowledge of the intervention received?	Re-intubation is an objective variable and the outcome measurement is unlikely to be influence by knowledge of the intervention received.	PN
6.2 Were outcome assessors aware of the intervention received by study participants?	There is no explicit mention that concealment was maintained throughout outcomes evaluation and outcome assessment was performed by investigators who were not involved in patient care	PY
6.3 Were the methods of outcome assessment comparable across intervention groups?		PY
6.4 Were any systematic errors in measurement of the outcome related to intervention received?		N
**Risk of bias judgement**		Moderate
Optional: What is the predicted direction of bias due to measurement of outcomes?		Unpredictable

**Bias in selection of the reported result**		
Is the reported effect estimate likely to be selected, on the basis of the results, from...		
7.1. ... multiple outcome *measurements* within the outcome domain?	Re-intubation is an objective variable and the outcome measurement is unlikely to be influence by knowledge of the intervention received.	N
7.2 ... multiple *analyses* of the intervention-outcome relationship?	Re-intubation is an objective variable and the outcome measurement is unlikely to be influence by knowledge of the intervention received.	N
7.3 ... different *subgroups*?	Re-intubation is an objective variable and the outcome measurement is unlikely to be influence by knowledge of the intervention received.	N
**Risk of bias judgement**	Low	Low
Optional: What is the predicted direction of bias due to selection of the reported result?		Unpredictable

Overall bias		
**Risk of bias judgement**		Critical
Optional: What is the overall predicted direction of bias for this outcome?		Unpredictable

This work is licensed under a Creative Commons Attribution-NonCommercial-NoDerivatives 4.0 International License.

The Risk Of Bias In Non-randomized Studies – of Interventions (ROBINS-I) assessment tool
(version for cohort-type studies)
Version 19 September 2016

This work is licensed under a Creative Commons Attribution-NonCommercial-NoDerivatives 4.0 International License.

Kurt 2006

ROBINS-I tool (Stage I): At protocol stage

Specify the review question

Participants	Patients undergoing extubation after surgery and subsequent non-invasive respiratory support
Experimental intervention	One modality of non-invasive respiratory support
Comparator	One or more than one modality of non-invasive respiratory support
Outcomes	Re-intubation rate

List the confounding domains relevant to all or most studies

Type of surgery, clinical setting, preoperative risk of postoperative pulmonary complications

List co-interventions that could be different between intervention groups and that could impact on outcomes

No co-interventions are expected to be different between intervention groups

ROBINS-I tool (Stage II): For each study

Specify a target randomized trial specific to the study

Design	Individually randomized
Participants	Patients undergoing extubation after surgery and subsequent non-invasive respiratory support
Experimental intervention	CPAP
Comparator	NIV

Is your aim for this study…?

X to assess the effect of *assignment to* intervention
☐ to assess the effect of *starting and adhering to* intervention

Specify the outcome

Specify which outcome is being assessed for risk of bias (typically from among those earmarked for the Summary of Findings table). Specify whether this is a proposed benefit or harm of intervention.

Re-intubation rate

Specify the numerical result being assessed

In case of multiple alternative analyses being presented, specify the numeric result (e.g. RR = 1.52 (95% CI 0.83 to 2.77) and/or a reference (e.g. to a table, figure or paragraph) that uniquely defines the result being assessed.

44/173 patients in the CPAP group, 4/18 patients in the NIV group

Preliminary consideration of confounders

Complete a row for each important confounding domain (i) listed in the review protocol; and (ii) relevant to the setting of this particular study, or which the study authors identified as potentially important.

"Important" confounding domains are those for which, in the context of this study, adjustment is expected to lead to a clinically important change in the estimated effect of the intervention. "Validity" refers to whether the confounding variable or variables fully measure the domain, while "reliability" refers to the precision of the measurement (more measurement error means less reliability).

**(i) Confounding domains listed in the review protocol**				
Confounding domain	Measured variable(s)	Is there evidence that controlling for this variable was unnecessary?*	Is the confounding domain measured validly and reliably by this variable (or these variables)?	OPTIONAL: Is failure to adjust for this variable (alone) expected to favour the experimental intervention or the comparator?
Type of surgery	Surgery performed above or below the diaphragm	All patients belong to the same confounding domain. Therefore, adjustment is not necessary	Yes	Not applicable
Clinical setting	Patients admitted to ICU, ward or high-dependency unit	All patients belong to the same confounding domain. Therefore, adjustment is not necessary	Yes	Not applicable
Preoperative risk of postoperative pulmonary complications	High or not-high risk	All patients belong to the same confounding domain. Therefore, adjustment is not necessary	Yes	Not applicable
Baseline characteristics	Time since primary extubation	No	Yes	No information

(ii) Additional confounding domains relevant to the setting of this particular study, or which the study authors identified as important				
Confounding domain	Measured variable(s)	Is there evidence that controlling for this variable was unnecessary?*	Is the confounding domain measured validly and reliably by this variable (or these variables)?	OPTIONAL: Is failure to adjust for this variable (alone) expected to favour the experimental intervention or the comparator?
			Yes / No / No information	Favour experimental / Favour comparator / No information
None				

* In the context of a particular study, variables can be demonstrated not to be confounders and so not included in the analysis: (a) if they are not predictive of the outcome; (b) if they are not predictive of intervention; or (c) because adjustment makes no or minimal difference to the estimated effect of the primary parameter. Note that “no statistically significant association” is not the same as “not predictive”.

Preliminary consideration of co-interventions

Complete a row for each important co-intervention (i) listed in the review protocol; and (ii) relevant to the setting of this particular study, or which the study authors identified as important.

"Important" co-interventions are those for which, in the context of this study, adjustment is expected to lead to a clinically important change in the estimated effect of the intervention.

**(i) Co-interventions listed in the review protocol**		
Co-intervention	Is there evidence that controlling for this co-intervention was unnecessary (e.g. because it was not administered)?	Is presence of this co-intervention likely to favour outcomes in the experimental intervention or the comparator
No co-interventions are expected to be different between intervention groups		

**(ii) Additional co-interventions relevant to the setting of this particular study, or which the study authors identified as important**		
Co-intervention	Is there evidence that controlling for this co-intervention was unnecessary (e.g. because it was not administered)?	Is presence of this co-intervention likely to favour outcomes in the experimental intervention or the comparator
No co-interventions are expected to be different between intervention groups		

Risk of bias assessment

Responses underlined in green are potential markers for low risk of bias, and responses in red are potential markers for a risk of bias. Where questions relate only to sign posts to other questions, no formatting is used.

Signalling questions	Description	Response options
**Bias due to confounding**		
1.1 Is there potential for confounding of the effect of intervention in this study?<br>**If** N/PN **to 1.1:** the study can be considered to be at low risk of bias due to confounding and no further signalling questions need be considered		PY
**If** Y/PY **to 1.1**: determine whether there is a need to assess time-varying confounding:		
1.2. Was the analysis based on splitting participants' follow up time according to intervention received?<br>**If N/PN**, answer questions relating to baseline confounding (1.4 to 1.6)<br>**If Y/PY**, go to question 1.3.	Participants cannot switch between interventions.	N
1.3. Were intervention discontinuations or switches likely to be related to factors that are prognostic for the outcome?<br>**If N/PN**, answer questions relating to baseline confounding (1.4 to 1.6)<br>**If Y/PY**, answer questions relating to both baseline and time-varying confounding (1.7 and 1.8)		NA

**Questions relating to baseline confounding only**		
1.4. Did the authors use an appropriate analysis method that controlled for all the important confounding domains?	No appropriate methods to control for measured confounders are used	N
1.5. **If** Y/PY **to 1.4**: Were confounding domains that were controlled for measured validly and reliably by the variables available in this study?		NA

1.6. Did the authors control for any post-intervention variables that could have been affected by the intervention?		N
**Questions relating to baseline and time-varying confounding**		
1.7. Did the authors use an appropriate analysis method that controlled for all the important confounding domains and for time-varying confounding?	No adjustment for time-varying confounding is used.	N
1.8. **If Y/PY to 1.7**: Were confounding domains that were controlled for measured validly and reliably by the variables available in this study?		NA
**Risk of bias judgement**		Critical
Optional: What is the predicted direction of bias due to confounding?		Unpredictable

**Bias in selection of participants into the study**		
2.1. Was selection of participants into the study (or into the analysis) based on participant characteristics observed after the start of intervention?<br>**If N/PN to 2.1:** go to 2.4	The inclusion criterion (PaO2/FiO2 deterioration) was assessed before the initiation of the intervention	PN
2.2. **If Y/PY to 2.1**: Were the post-intervention variables that influenced selection likely to be associated with intervention?		NA
2.3 **If Y/PY to 2.2**: Were the post-intervention variables that influenced selection likely to be influenced by the outcome or a cause of the outcome?		NA
2.4. Do start of follow-up and start of intervention coincide for most participants?		NI
2.5. **If Y/PY to 2.2 and 2.3, or N/PN to 2.4**: Were adjustment techniques used that are likely to correct for the presence of selection biases?		NA
**Risk of bias judgement**		Moderate
Optional: What is the predicted direction of bias due to selection of participants into the study?		Unpredictable

Bias in classification of interventions		
3.1 Were intervention groups clearly defined?		Y
3.2 Was the information used to define intervention groups recorded at the start of the intervention?		Y
3.3 Could classification of intervention status have been affected by knowledge of the outcome or risk of the outcome?		N
**Risk of bias judgement**		Low
Optional: What is the predicted direction of bias due to classification of interventions?		Unpredictable

Bias due to deviations from intended interventions		
**If your aim for this study is to assess the effect of assignment to intervention, answer questions 4.1 and 4.2**		
4.1. Were there deviations from the intended intervention beyond what would be expected in usual practice?	No deviations from the intended intervention beyond what would be expected in usual practice are reported.	PN
4.2. **If Y/PY to 4.1**: Were these deviations from intended intervention unbalanced between groups *and* likely to have affected the outcome?		NA
**Risk of bias judgement**		Low
Optional: What is the predicted direction of bias due to deviations from the intended interventions?		Unpredictable

Bias due to missing data		
5.1 Were outcome data available for all, or nearly all, participants?		Y
5.2 Were participants excluded due to missing data on intervention status?		N
5.3 Were participants excluded due to missing data on other variables needed for the analysis?		N
5.4 **If PN/N to 5.1, or Y/PY to 5.2 or 5.3**: Are the proportion of participants and reasons for missing data similar across interventions?		NA

5.5 **If PN/N to 5.1, or Y/PY to 5.2 or 5.3**: Is there evidence that results were robust to the presence of missing data?		NA
**Risk of bias judgement**		Low
Optional: What is the predicted direction of bias due to missing data?		Unpredictable

**Bias in measurement of outcomes**		
6.1 Could the outcome measure have been influenced by knowledge of the intervention received?	Re-intubation is an objective variable and the outcome measurement is unlikely to be influence by knowledge of the intervention received.	PN
6.2 Were outcome assessors aware of the intervention received by study participants?	There is no explicit mention that concealment was maintained throughout outcomes evaluation and outcome assessment was performed by investigators who were not involved in patient care	PY
6.3 Were the methods of outcome assessment comparable across intervention groups?		PY
6.4 Were any systematic errors in measurement of the outcome related to intervention received?		N
**Risk of bias judgement**		Moderate
Optional: What is the predicted direction of bias due to measurement of outcomes?		Unpredictable

**Bias in selection of the reported result**		
Is the reported effect estimate likely to be selected, on the basis of the results, from...		
7.1. ... multiple outcome *measurements* within the outcome domain?	Re-intubation is an objective variable and the outcome measurement is unlikely to be influence by knowledge of the intervention received.	N
7.2 ... multiple *analyses* of the intervention-outcome relationship?	Re-intubation is an objective variable and the outcome measurement is unlikely to be influence by knowledge of the intervention received.	N
7.3 ... different *subgroups*?	Re-intubation is an objective variable and the outcome measurement is unlikely to be influence by knowledge of the intervention received.	N
**Risk of bias judgement**	Low	Low
Optional: What is the predicted direction of bias due to selection of the reported result?		Unpredictable

Overall bias		
**Risk of bias judgement**		Critical
Optional: What is the overall predicted direction of bias for this outcome?		Unpredictable

This work is licensed under a Creative Commons Attribution-NonCommercial-NoDerivatives 4.0 International License.

The Risk Of Bias In Non-randomized Studies – of Interventions (ROBINS-I) assessment tool
(version for cohort-type studies)
Version 19 September 2016

This work is licensed under a Creative Commons Attribution-NonCommercial-NoDerivatives 4.0 International License.

Melton 2019

ROBINS-I tool (Stage I): At protocol stage

Specify the review question

Participants	Patients undergoing extubation after surgery and subsequent non-invasive respiratory support
Experimental intervention	One modality of non-invasive respiratory support
Comparator	One or more than one modality of non-invasive respiratory support
Outcomes	Re-intubation rate

List the confounding domains relevant to all or most studies

Type of surgery, clinical setting, preoperative risk of postoperative pulmonary complications

List co-interventions that could be different between intervention groups and that could impact on outcomes

No co-interventions are expected to be different between intervention groups

ROBINS-I tool (Stage II): For each study

Specify a target randomized trial specific to the study

Design	Individually randomized
Participants	Patients undergoing extubation after surgery and subsequent non-invasive respiratory support
Experimental intervention	NIV
Comparator	COT

Is your aim for this study…?

X to assess the effect of *assignment to* intervention

☐ to assess the effect of *starting and adhering to* intervention

Specify the outcome

Specify which outcome is being assessed for risk of bias (typically from among those earmarked for the Summary of Findings table). Specify whether this is a proposed benefit or harm of intervention.

Re-intubation rate

Specify the numerical result being assessed

In case of multiple alternative analyses being presented, specify the numeric result (e.g. RR = 1.52 (95% CI 0.83 to 2.77) and/or a reference (e.g. to a table, figure or paragraph) that uniquely defines the result being assessed.

34/859 patients in the COT group, 35/859 patients in the NIV group

Preliminary consideration of confounders

Complete a row for each important confounding domain (i) listed in the review protocol; and (ii) relevant to the setting of this particular study, or which the study authors identified as potentially important.

"Important" confounding domains are those for which, in the context of this study, adjustment is expected to lead to a clinically important change in the estimated effect of the intervention. "Validity" refers to whether the confounding variable or variables fully measure the domain, while "reliability" refers to the precision of the measurement (more measurement error means less reliability).

(i) Confounding domains listed in the review protocol				
Confounding domain	Measured variable(s)	Is there evidence that controlling for this variable was unnecessary?*	Is the confounding domain measured validly and reliably by this variable (or these variables)?	OPTIONAL: Is failure to adjust for this variable (alone) expected to favour the experimental intervention or the comparator?
Type of surgery	Surgery performed above or below the diaphragm	All patients belong to the same confounding domain. Therefore, adjustment is not necessary	Yes	Not applicable
Clinical setting	Patients admitted to ICU, ward or high-dependency unit	All patients belong to the same confounding domain. Therefore, adjustment is not necessary	Yes	Not applicable
Preoperative risk of postoperative pulmonary complications	High or not-high risk	All patients belong to the same confounding domain. Therefore, adjustment is not necessary	Yes	Not applicable
Medical history	Number of patients with cerebrovascular disease, history of heart failure, history of percutaneous coronary intervention, history of coronary stenting, nsmoking history, using daily bronchodilators, history of pneumonia; forced expiratory volume in one second	No	Yes	No information
Intraoperative characteristics	Frequency of elective surgery, time in the operating room and on cardiopulmonary bypass, time to extubation	No	Yes	

(ii) Additional confounding domains relevant to the setting of this particular study, or which the study authors identified as important				
Confounding domain	Measured variable(s)	Is there evidence that controlling for this variable was unnecessary?*	Is the confounding domain measured validly and reliably by this variable (or these variables)?	OPTIONAL: Is failure to adjust for this variable (alone) expected to favour the experimental intervention or the comparator?
			Yes / No / No information	Favour experimental / Favour comparator / No information
None				

* In the context of a particular study, variables can be demonstrated not to be confounders and so not included in the analysis: (a) if they are not predictive of the outcome; (b) if they are not predictive of intervention; or (c) because adjustment makes no or minimal difference to the estimated effect of the primary parameter. Note that "no statistically significant association" is not the same as "not predictive".

Preliminary consideration of co-interventions

Complete a row for each important co-intervention (i) listed in the review protocol; and (ii) relevant to the setting of this particular study, or which the study authors identified as important.

"Important" co-interventions are those for which, in the context of this study, adjustment is expected to lead to a clinically important change in the estimated effect of the intervention.

**(i) Co-interventions listed in the review protocol**		
Co-intervention	Is there evidence that controlling for this co-intervention was unnecessary (e.g. because it was not administered)?	Is presence of this co-intervention likely to favour outcomes in the experimental intervention or the comparator
No co-interventions are expected to be different between intervention groups		

**(ii) Additional co-interventions relevant to the setting of this particular study, or which the study authors identified as important**		
Co-intervention	Is there evidence that controlling for this co-intervention was unnecessary (e.g. because it was not administered)?	Is presence of this co-intervention likely to favour outcomes in the experimental intervention or the comparator
No co-interventions are expected to be different between intervention groups		

Risk of bias assessment

Responses underlined in green are potential markers for low risk of bias, and responses in red are potential markers for a risk of bias. Where questions relate only to sign posts to other questions, no formatting is used.

Signalling questions	Description	Response options
**Bias due to confounding**		
1.1 Is there potential for confounding of the effect of intervention in this study?<br>**If N/PN to 1.1:** the study can be considered to be at low risk of bias due to confounding and no further signalling questions need be considered		PY
**If Y/PY to 1.1**: determine whether there is a need to assess time-varying confounding:		
1.2. Was the analysis based on splitting participants' follow up time according to intervention received?<br>**If N/PN**, answer questions relating to baseline confounding (1.4 to 1.6)<br>**If Y/PY**, go to question 1.3.	Participants cannot switch between interventions.	N
1.3. Were intervention discontinuations or switches likely to be related to factors that are prognostic for the outcome?<br>**If N/PN**, answer questions relating to baseline confounding (1.4 to 1.6)<br>**If Y/PY**, answer questions relating to both baseline and time-varying confounding (1.7 and 1.8)		NA

**Questions relating to baseline confounding only**		
1.4. Did the authors use an appropriate analysis method that controlled for all the important confounding domains?	No appropriate methods to control for measured confounders (baseline patient characteristics and intraoperative variables) are used.	N
1.5. **If Y/PY to 1.4**: Were confounding domains that were controlled for measured validly and reliably by the variables available in this study?		NA

1.6. Did the authors control for any post-intervention variables that could have been affected by the intervention?		N
**Questions relating to baseline and time-varying confounding**		
1.7. Did the authors use an appropriate analysis method that controlled for all the important confounding domains and for time-varying confounding?	No adjustment for time-varying confounding is used.	N
1.8. **If Y/PY to 1.7**: Were confounding domains that were controlled for measured validly and reliably by the variables available in this study?		NA
**Risk of bias judgement**		Critical
Optional: What is the predicted direction of bias due to confounding?		Unpredictable

**Bias in selection of participants into the study**		
2.1. Was selection of participants into the study (or into the analysis) based on participant characteristics observed after the start of intervention?	The inclusion criterion (cardiac surgery) was assessed before the initiation of the intervention	PN
**If N/PN to 2.1:** go to 2.4		
2.2. **If Y/PY to 2.1**: Were the post-intervention variables that influenced selection likely to be associated with intervention?		NA
2.3 **If Y/PY to 2.2**: Were the post-intervention variables that influenced selection likely to be influenced by the outcome or a cause of the outcome?		NA
2.4. Do start of follow-up and start of intervention coincide for most participants?	Citation: “Eight hundred and fifty-nine patients from May 2014 to April 2016 were in the protocol cohort. This protocol cohort was retrospectively compared to the control group (nasal cannula only), of 859 patients, from May 2012 to April 2014.”	N
2.5. **If Y/PY to 2.2 and 2.3, or N/PN to 2.4**: Were adjustment techniques used that are likely to correct for the presence of selection biases?		N
**Risk of bias judgement**		Serious

Optional: What is the predicted direction of bias due to selection of participants into the study?		Unpredictable

Bias in classification of interventions		
3.1 Were intervention groups clearly defined?		Y
3.2 Was the information used to define intervention groups recorded at the start of the intervention?		Y
3.3 Could classification of intervention status have been affected by knowledge of the outcome or risk of the outcome?		N
**Risk of bias judgement**		Low
Optional: What is the predicted direction of bias due to classification of interventions?		Unpredictable

Bias due to deviations from intended interventions		
**If your aim for this study is to assess the effect of assignment to intervention, answer questions 4.1 and 4.2**		
4.1. Were there deviations from the intended intervention beyond what would be expected in usual practice?	No deviations from the intended intervention beyond what would be expected in usual practice are reported.	PN
4.2. **If Y/PY to 4.1**: Were these deviations from intended intervention unbalanced between groups *and* likely to have affected the outcome?		NA
**Risk of bias judgement**		Low
Optional: What is the predicted direction of bias due to deviations from the intended interventions?		Unpredictable

Bias due to missing data		
5.1 Were outcome data available for all, or nearly all, participants?		Y
5.2 Were participants excluded due to missing data on intervention status?		N
5.3 Were participants excluded due to missing data on other variables needed for the analysis?		N

5.4 **If PN/N to 5.1, or Y/PY to 5.2 or 5.3**: Are the proportion of participants and reasons for missing data similar across interventions?		NA
5.5 **If PN/N to 5.1, or Y/PY to 5.2 or 5.3**: Is there evidence that results were robust to the presence of missing data?		NA
**Risk of bias judgement**		Low
Optional: What is the predicted direction of bias due to missing data?		Unpredictable

**Bias in measurement of outcomes**		
6.1 Could the outcome measure have been influenced by knowledge of the intervention received?	Re-intubation is an objective variable and the outcome measurement is unlikely to be influence by knowledge of the intervention received.	PN
6.2 Were outcome assessors aware of the intervention received by study participants?	There is no explicit mention that concealment was maintained throughout outcomes evaluation and outcome assessment was performed by investigators who were not involved in patient care	PY
6.3 Were the methods of outcome assessment comparable across intervention groups?		PY
6.4 Were any systematic errors in measurement of the outcome related to intervention received?		N
**Risk of bias judgement**		Moderate
Optional: What is the predicted direction of bias due to measurement of outcomes?		Unpredictable

**Bias in selection of the reported result**		
Is the reported effect estimate likely to be selected, on the basis of the results, from...<br>7.1. ... multiple outcome *measurements* within the outcome domain?	Re-intubation is an objective variable and the outcome measurement is unlikely to be influence by knowledge of the intervention received.	N
7.2 ... multiple *analyses* of the intervention-outcome relationship?	Re-intubation is an objective variable and the outcome measurement is unlikely to be influence by knowledge of the intervention received.	N
7.3 ... different *subgroups*?	Re-intubation is an objective variable and the outcome measurement is unlikely to be influence by knowledge of the intervention received.	N
**Risk of bias judgement**	Low	Low

Optional: What is the predicted direction of bias due to selection of the reported result?		Unpredictable

**Overall bias**		
**Risk of bias judgement**		Critical
Optional: What is the overall predicted direction of bias for this outcome?		Unpredictable

This work is licensed under a Creative Commons Attribution-NonCommercial-NoDerivatives 4.0 International License.

The Risk Of Bias In Non-randomized Studies – of Interventions (ROBINS-I) assessment tool
(version for cohort-type studies)
Version 19 September 2016

This work is licensed under a Creative Commons Attribution-NonCommercial-NoDerivatives 4.0 International License.

Xia 2021

ROBINS-I tool (Stage I): At protocol stage

Specify the review question

Participants	Patients undergoing extubation after surgery and subsequent non-invasive respiratory support
Experimental intervention	One modality of non-invasive respiratory support
Comparator	One or more than one modality of non-invasive respiratory support
Outcomes	Re-intubation rate

List the confounding domains relevant to all or most studies

Type of surgery, clinical setting, preoperative risk of postoperative pulmonary complications

List co-interventions that could be different between intervention groups and that could impact on outcomes

No co-interventions are expected to be different between intervention groups

ROBINS-I tool (Stage II): For each study

Specify a target randomized trial specific to the study

Design	Individually randomized
Participants	Patients undergoing extubation after surgery and subsequent non-invasive respiratory support
Experimental intervention	HFNO
Comparator	COT

Is your aim for this study…?

X to assess the effect of *assignment to* intervention

☐ to assess the effect of *starting and adhering to* intervention

Specify the outcome

Specify which outcome is being assessed for risk of bias (typically from among those earmarked for the Summary of Findings table). Specify whether this is a proposed benefit or harm of intervention.

Re-intubation rate

Specify the numerical result being assessed

In case of multiple alternative analyses being presented, specify the numeric result (e.g. RR = 1.52 (95% CI 0.83 to 2.77) and/or a reference (e.g. to a table, figure or paragraph) that uniquely defines the result being assessed.

8/99 patients in the COT group, 3/78 patients in the HFNO group

Preliminary consideration of confounders

Complete a row for each important confounding domain (i) listed in the review protocol; and (ii) relevant to the setting of this particular study, or which the study authors identified as potentially important.

"Important" confounding domains are those for which, in the context of this study, adjustment is expected to lead to a clinically important change in the estimated effect of the intervention. "Validity" refers to whether the confounding variable or variables fully measure the domain, while "reliability" refers to the precision of the measurement (more measurement error means less reliability).

**(i) Confounding domains listed in the review protocol**				
Confounding domain	Measured variable(s)	Is there evidence that controlling for this variable was unnecessary?*	Is the confounding domain measured validly and reliably by this variable (or these variables)?	OPTIONAL: Is failure to adjust for this variable (alone) expected to favour the experimental intervention or the comparator?
Type of surgery	Surgery performed above or below the diaphragm	All patients belong to the same confounding domain. Therefore, adjustment is not necessary	Yes	Not applicable
Clinical setting	Patients admitted to ICU, ward or high-dependency unit	All patients belong to the same confounding domain. Therefore, adjustment is not necessary	Yes	Not applicable
Preoperative risk of postoperative pulmonary complications	High or not-high risk	All patients belong to the same confounding domain. Therefore, adjustment is not necessary	Yes	Not applicable
Baseline characteristics	Intraoperative anesthesia, operation method and operator, age and BMI, smoking index, respiratory rate, gender, tumor location, preoperative FEV1/FVC, Hb, PaO2, operation time and thoracoscopic outcomes	Yes	Yes	No information

(ii) Additional confounding domains relevant to the setting of this particular study, or which the study authors identified as important				
Confounding domain	Measured variable(s)	Is there evidence that controlling for this variable was unnecessary?*	Is the confounding domain measured validly and reliably by this variable (or these variables)?	OPTIONAL: Is failure to adjust for this variable (alone) expected to favour the experimental intervention or the comparator?
			Yes / No / No information	Favour experimental / Favour comparator / No information
None				

* In the context of a particular study, variables can be demonstrated not to be confounders and so not included in the analysis: (a) if they are not predictive of the outcome; (b) if they are not predictive of intervention; or (c) because adjustment makes no or minimal difference to the estimated effect of the primary parameter. Note that “no statistically significant association” is not the same as “not predictive”.

Preliminary consideration of co-interventions

Complete a row for each important co-intervention (i) listed in the review protocol; and (ii) relevant to the setting of this particular study, or which the study authors identified as important.

"Important" co-interventions are those for which, in the context of this study, adjustment is expected to lead to a clinically important change in the estimated effect of the intervention.

**(i) Co-interventions listed in the review protocol**		
Co-intervention	Is there evidence that controlling for this co-intervention was unnecessary (e.g. because it was not administered)?	Is presence of this co-intervention likely to favour outcomes in the experimental intervention or the comparator
No co-interventions are expected to be different between intervention groups		

**(ii) Additional co-interventions relevant to the setting of this particular study, or which the study authors identified as important**		
Co-intervention	Is there evidence that controlling for this co-intervention was unnecessary (e.g. because it was not administered)?	Is presence of this co-intervention likely to favour outcomes in the experimental intervention or the comparator
No co-interventions are expected to be different between intervention groups		

Risk of bias assessment

Responses underlined in green are potential markers for low risk of bias, and responses in red are potential markers for a risk of bias. Where questions relate only to sign posts to other questions, no formatting is used.

Signalling questions	Description	Response options
**Bias due to confounding**		
1.1 Is there potential for confounding of the effect of intervention in this study?<br>**If N/PN to 1.1:** the study can be considered to be at low risk of bias due to confounding and no further signalling questions need be considered		PY
**If Y/PY to 1.1**: determine whether there is a need to assess time-varying confounding:		
1.2. Was the analysis based on splitting participants' follow up time according to intervention received?<br>**If N/PN**, answer questions relating to baseline confounding (1.4 to 1.6)<br>**If Y/PY**, go to question 1.3.	Participants cannot switch between interventions.	N
1.3. Were intervention discontinuations or switches likely to be related to factors that are prognostic for the outcome?<br>**If N/PN**, answer questions relating to baseline confounding (1.4 to 1.6)<br>**If Y/PY**, answer questions relating to both baseline and time-varying confounding (1.7 and 1.8)		NA

**Questions relating to baseline confounding only**		
1.4. Did the authors use an appropriate analysis method that controlled for all the important confounding domains?	No measured confounders among baseline patient characteristics and intraoperative variables are detected.	NA
1.5. **If Y/PY to 1.4**: Were confounding domains that were controlled for measured validly and reliably by the variables available in this study?		NA

1.6. Did the authors control for any post-intervention variables that could have been affected by the intervention?		N
**Questions relating to baseline and time-varying confounding**		
1.7. Did the authors use an appropriate analysis method that controlled for all the important confounding domains and for time-varying confounding?	No adjustment for time-varying confounding is used.	N
1.8. **If Y/PY to 1.7**: Were confounding domains that were controlled for measured validly and reliably by the variables available in this study?		NA
**Risk of bias judgement**		Serious
Optional: What is the predicted direction of bias due to confounding?		Unpredictable

**Bias in selection of participants into the study**		
2.1. Was selection of participants into the study (or into the analysis) based on participant characteristics observed after the start of intervention?<br>**If N/PN to 2.1:** go to 2.4	The inclusion criterion (hypoxemia after extubation) was assessed before the initiation of the intervention	PN
2.2. **If Y/PY to 2.1**: Were the post-intervention variables that influenced selection likely to be associated with intervention?		NA
2.3 **If Y/PY to 2.2**: Were the post-intervention variables that influenced selection likely to be influenced by the outcome or a cause of the outcome?		NA
2.4. Do start of follow-up and start of intervention coincide for most participants?		NI
2.5. **If Y/PY to 2.2 and 2.3, or N/PN to 2.4**: Were adjustment techniques used that are likely to correct for the presence of selection biases?		NA
**Risk of bias judgement**		Moderate
Optional: What is the predicted direction of bias due to selection of participants into the study?		Unpredictable

Bias in classification of interventions		
3.1 Were intervention groups clearly defined?		Y
3.2 Was the information used to define intervention groups recorded at the start of the intervention?		Y
3.3 Could classification of intervention status have been affected by knowledge of the outcome or risk of the outcome?		N
**Risk of bias judgement**		Low
Optional: What is the predicted direction of bias due to classification of interventions?		Unpredictable

Bias due to deviations from intended interventions		
**If your aim for this study is to assess the effect of assignment to intervention, answer questions 4.1 and 4.2**		
4.1. Were there deviations from the intended intervention beyond what would be expected in usual practice?	No deviations from the intended intervention beyond what would be expected in usual practice are reported.	PN
4.2. **If Y/PY to 4.1**: Were these deviations from intended intervention unbalanced between groups *and* likely to have affected the outcome?		NA
**Risk of bias judgement**		Low
Optional: What is the predicted direction of bias due to deviations from the intended interventions?		Unpredictable

Bias due to missing data		
5.1 Were outcome data available for all, or nearly all, participants?		Y
5.2 Were participants excluded due to missing data on intervention status?		N
5.3 Were participants excluded due to missing data on other variables needed for the analysis?		N
5.4 **If PN/N to 5.1, or Y/PY to 5.2 or 5.3**: Are the proportion of participants and reasons for missing data similar across interventions?		NA

5.5 **If PN/N to 5.1, or Y/PY to 5.2 or 5.3**: Is there evidence that results were robust to the presence of missing data?		NA
**Risk of bias judgement**		Low
Optional: What is the predicted direction of bias due to missing data?		Unpredictable

**Bias in measurement of outcomes**		
6.1 Could the outcome measure have been influenced by knowledge of the intervention received?	Re-intubation is an objective variable and the outcome measurement is unlikely to be influence by knowledge of the intervention received.	PN
6.2 Were outcome assessors aware of the intervention received by study participants?	There is no explicit mention that concealment was maintained throughout outcomes evaluation and outcome assessment was performed by investigators who were not involved in patient care	PY
6.3 Were the methods of outcome assessment comparable across intervention groups?		PY
6.4 Were any systematic errors in measurement of the outcome related to intervention received?		N
**Risk of bias judgement**		Moderate
Optional: What is the predicted direction of bias due to measurement of outcomes?		Unpredictable

**Bias in selection of the reported result**		
Is the reported effect estimate likely to be selected, on the basis of the results, from...<br>7.1. ... multiple outcome *measurements* within the outcome domain?	Re-intubation is an objective variable and the outcome measurement is unlikely to be influence by knowledge of the intervention received.	N
7.2 ... multiple *analyses* of the intervention-outcome relationship?	Re-intubation is an objective variable and the outcome measurement is unlikely to be influence by knowledge of the intervention received.	N
7.3 ... different *subgroups*?	Re-intubation is an objective variable and the outcome measurement is unlikely to be influence by knowledge of the intervention received.	N
**Risk of bias judgement**	Low	Low
Optional: What is the predicted direction of bias due to selection of the reported result?		Unpredictable

**Overall bias**		
**Risk of bias judgement**		Serious
Optional: What is the overall predicted direction of bias for this outcome?		Unpredictable

This work is licensed under a Creative Commons Attribution-NonCommercial-NoDerivatives 4.0 International License.

The Risk Of Bias In Non-randomized Studies – of Interventions (ROBINS-I) assessment tool
(version for cohort-type studies)
Version 19 September 2016

This work is licensed under a Creative Commons Attribution-NonCommercial-NoDerivatives 4.0 International License.

Yan 2021

ROBINS-I tool (Stage I): At protocol stage

Specify the review question

Participants	Patients undergoing extubation after surgery and subsequent non-invasive respiratory support
Experimental intervention	One modality of non-invasive respiratory support
Comparator	One or more than one modality of non-invasive respiratory support
Outcomes	Re-intubation rate

List the confounding domains relevant to all or most studies

Type of surgery, clinical setting, preoperative risk of postoperative pulmonary complications

List co-interventions that could be different between intervention groups and that could impact on outcomes

No co-interventions are expected to be different between intervention groups

ROBINS-I tool (Stage II): For each study

Specify a target randomized trial specific to the study

Design	Individually randomized
Participants	Patients undergoing extubation after surgery and subsequent non-invasive respiratory support
Experimental intervention	NIV
Comparator	COT

Is your aim for this study…?

X to assess the effect of *assignment to* intervention
☐ to assess the effect of *starting and adhering to* intervention

Specify the outcome

Specify which outcome is being assessed for risk of bias (typically from among those earmarked for the Summary of Findings table). Specify whether this is a proposed benefit or harm of intervention.

Re-intubation rate

Specify the numerical result being assessed

In case of multiple alternative analyses being presented, specify the numeric result (e.g. RR = 1.52 (95% CI 0.83 to 2.77) and/or a reference (e.g. to a table, figure or paragraph) that uniquely defines the result being assessed.

8/46 patients in the COT group, 1/41 patients in the HFNO group

Preliminary consideration of confounders

Complete a row for each important confounding domain (i) listed in the review protocol; and (ii) relevant to the setting of this particular study, or which the study authors identified as potentially important.

"Important" confounding domains are those for which, in the context of this study, adjustment is expected to lead to a clinically important change in the estimated effect of the intervention. "Validity" refers to whether the confounding variable or variables fully measure the domain, while "reliability" refers to the precision of the measurement (more measurement error means less reliability).

**(i) Confounding domains listed in the review protocol**				
Confounding domain	Measured variable(s)	Is there evidence that controlling for this variable was unnecessary?*	Is the confounding domain measured validly and reliably by this variable (or these variables)?	OPTIONAL: Is failure to adjust for this variable (alone) expected to favour the experimental intervention or the comparator?
Type of surgery	Surgery performed above or below the diaphragm	All patients belong to the same confounding domain. Therefore, adjustment is not necessary	Yes	Not applicable
Clinical setting	Patients admitted to ICU, ward or high-dependency unit	All patients belong to the same confounding domain. Therefore, adjustment is not necessary	Yes	Not applicable
Preoperative risk of postoperative pulmonary complications	High or not-high risk	All patients belong to the same confounding domain. Therefore, adjustment is not necessary	Yes	Not applicable
Baseline and intraoperative characteristics		Yes	Yes	No information

(ii) Additional confounding domains relevant to the setting of this particular study, or which the study authors identified as important				
Confounding domain	Measured variable(s)	Is there evidence that controlling for this variable was unnecessary?*	Is the confounding domain measured validly and reliably by this variable (or these variables)?	OPTIONAL: Is failure to adjust for this variable (alone) expected to favour the experimental intervention or the comparator?
			Yes / No / No information	Favour experimental / Favour comparator / No information
None				

* In the context of a particular study, variables can be demonstrated not to be confounders and so not included in the analysis: (a) if they are not predictive of the outcome; (b) if they are not predictive of intervention; or (c) because adjustment makes no or minimal difference to the estimated effect of the primary parameter. Note that "no statistically significant association" is not the same as "not predictive".

Preliminary consideration of co-interventions

Complete a row for each important co-intervention (i) listed in the review protocol; and (ii) relevant to the setting of this particular study, or which the study authors identified as important.

"Important" co-interventions are those for which, in the context of this study, adjustment is expected to lead to a clinically important change in the estimated effect of the intervention.

**(i) Co-interventions listed in the review protocol**		
Co-intervention	Is there evidence that controlling for this co-intervention was unnecessary (e.g. because it was not administered)?	Is presence of this co-intervention likely to favour outcomes in the experimental intervention or the comparator
No co-interventions are expected to be different between intervention groups		

**(ii) Additional co-interventions relevant to the setting of this particular study, or which the study authors identified as important**		
Co-intervention	Is there evidence that controlling for this co-intervention was unnecessary (e.g. because it was not administered)?	Is presence of this co-intervention likely to favour outcomes in the experimental intervention or the comparator
No co-interventions are expected to be different between intervention groups		

Risk of bias assessment

Responses underlined in green are potential markers for low risk of bias, and responses in red are potential markers for a risk of bias. Where questions relate only to sign posts to other questions, no formatting is used.

Signalling questions	Description	Response options
**Bias due to confounding**		
1.1 Is there potential for confounding of the effect of intervention in this study?<br>**If N/PN to 1.1:** the study can be considered to be at low risk of bias due to confounding and no further signalling questions need be considered		PY
**If Y/PY to 1.1**: determine whether there is a need to assess time-varying confounding:		
1.2. Was the analysis based on splitting participants' follow up time according to intervention received?<br>**If N/PN**, answer questions relating to baseline confounding (1.4 to 1.6)<br>**If Y/PY**, go to question 1.3.	Participants cannot switch between interventions.	N
1.3. Were intervention discontinuations or switches likely to be related to factors that are prognostic for the outcome?<br>**If N/PN**, answer questions relating to baseline confounding (1.4 to 1.6)<br>**If Y/PY**, answer questions relating to both baseline and time-varying confounding (1.7 and 1.8)		NA

**Questions relating to baseline confounding only**		
1.4. Did the authors use an appropriate analysis method that controlled for all the important confounding domains?	No measured confounders (baseline patient characteristics and intraoperative variables) are observed.	NA
1.5. **If Y/PY to 1.4**: Were confounding domains that were controlled for measured validly and reliably by the variables available in this study?		NA

1.6. Did the authors control for any post-intervention variables that could have been affected by the intervention?		N
**Questions relating to baseline and time-varying confounding**		
1.7. Did the authors use an appropriate analysis method that controlled for all the important confounding domains and for time-varying confounding?	No adjustment for time-varying confounding is used.	N
1.8. **If Y/PY to 1.7**: Were confounding domains that were controlled for measured validly and reliably by the variables available in this study?		NA
**Risk of bias judgement**		Serious
Optional: What is the predicted direction of bias due to confounding?		Unpredictable

**Bias in selection of participants into the study**		
2.1. Was selection of participants into the study (or into the analysis) based on participant characteristics observed after the start of intervention?	The inclusion criteria are assessed before the initiation of the intervention.	PN
**If N/PN to 2.1:** go to 2.4		
2.2. **If Y/PY to 2.1**: Were the post-intervention variables that influenced selection likely to be associated with intervention?		NA
2.3 **If Y/PY to 2.2**: Were the post-intervention variables that influenced selection likely to be influenced by the outcome or a cause of the outcome?		NA
2.4. Do start of follow-up and start of intervention coincide for most participants?	Citation: "In these hospitals, COT was used in all patients before July 2017, and HNFC was used thereafter".	N
2.5. **If Y/PY to 2.2 and 2.3, or N/PN to 2.4**: Were adjustment techniques used that are likely to correct for the presence of selection biases?		N
**Risk of bias judgement**		Serious
Optional: What is the predicted direction of bias due to selection of participants into the study?		Unpredictable

Bias in classification of interventions		
3.1 Were intervention groups clearly defined?		Y
3.2 Was the information used to define intervention groups recorded at the start of the intervention?		Y
3.3 Could classification of intervention status have been affected by knowledge of the outcome or risk of the outcome?		N
**Risk of bias judgement**		Low
Optional: What is the predicted direction of bias due to classification of interventions?		Unpredictable

Bias due to deviations from intended interventions		
**If your aim for this study is to assess the effect of assignment to intervention, answer questions 4.1 and 4.2**		
4.1. Were there deviations from the intended intervention beyond what would be expected in usual practice?	No deviations from the intended intervention beyond what would be expected in usual practice are reported.	PN
4.2. **If Y/PY to 4.1**: Were these deviations from intended intervention unbalanced between groups *and* likely to have affected the outcome?		NA
**Risk of bias judgement**		Low
Optional: What is the predicted direction of bias due to deviations from the intended interventions?		Unpredictable

Bias due to missing data		
5.1 Were outcome data available for all, or nearly all, participants?		Y
5.2 Were participants excluded due to missing data on intervention status?		N
5.3 Were participants excluded due to missing data on other variables needed for the analysis?		N
5.4 **If PN/N to 5.1, or Y/PY to 5.2 or 5.3**: Are the proportion of participants and reasons for missing data similar across interventions?		NA

5.5 **If PN/N to 5.1, or Y/PY to 5.2 or 5.3**: Is there evidence that results were robust to the presence of missing data?		NA
**Risk of bias judgement**		Low
Optional: What is the predicted direction of bias due to missing data?		Unpredictable

**Bias in measurement of outcomes**		
6.1 Could the outcome measure have been influenced by knowledge of the intervention received?	Re-intubation is an objective variable and the outcome measurement is unlikely to be influence by knowledge of the intervention received.	PN
6.2 Were outcome assessors aware of the intervention received by study participants?	There is no explicit mention that concealment was maintained throughout outcomes evaluation and outcome assessment was performed by investigators who were not involved in patient care	PY
6.3 Were the methods of outcome assessment comparable across intervention groups?		PY
6.4 Were any systematic errors in measurement of the outcome related to intervention received?		N
**Risk of bias judgement**		Moderate
Optional: What is the predicted direction of bias due to measurement of outcomes?		Unpredictable

**Bias in selection of the reported result**		
Is the reported effect estimate likely to be selected, on the basis of the results, from...<br>7.1. ... multiple outcome *measurements* within the outcome domain?	Re-intubation is an objective variable and the outcome measurement is unlikely to be influence by knowledge of the intervention received.	N
7.2 ... multiple *analyses* of the intervention-outcome relationship?	Re-intubation is an objective variable and the outcome measurement is unlikely to be influence by knowledge of the intervention received.	N
7.3 ... different *subgroups*?	Re-intubation is an objective variable and the outcome measurement is unlikely to be influence by knowledge of the intervention received.	N
**Risk of bias judgement**	Low	Low
Optional: What is the predicted direction of bias due to selection of the reported result?		Unpredictable

**Overall bias**		
**Risk of bias judgement**		Serious
Optional: What is the overall predicted direction of bias for this outcome?		Unpredictable

This work is licensed under a Creative Commons Attribution-NonCommercial-NoDerivatives 4.0 International License.

Supplementary Digital Content 6. Funnel plots for primary and secondary outcomes

P-value of the Egger's test is reported, when appropriate. Abbreviations: COT, conventional oxygen therapy; HFNO, high-flow nasal oxygen; CPAP, continuous positive airway pressure; NIV, non-invasive ventilation.

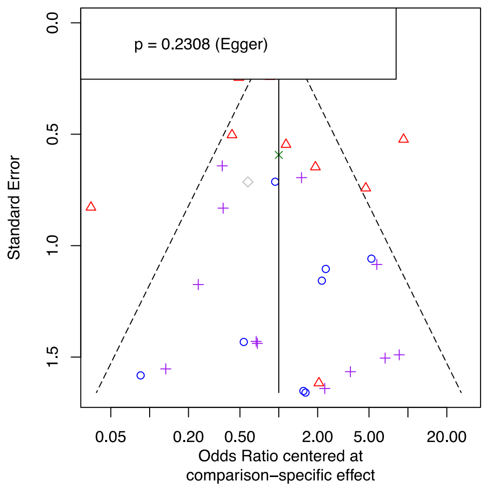

RE-INTUBATION

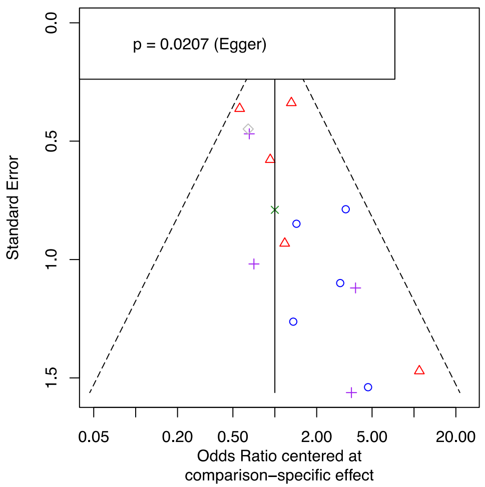

NOSOCOMIAL PNEUMONIA

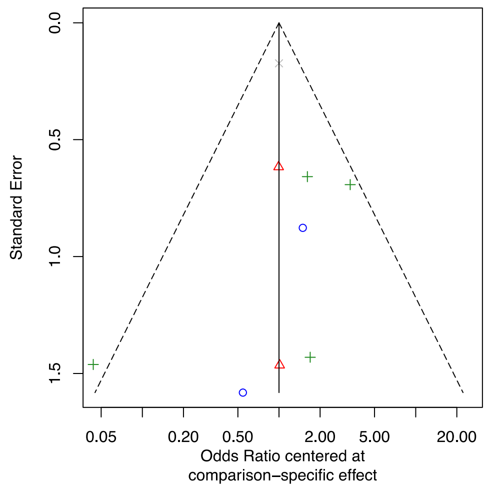

DISCOMFORT

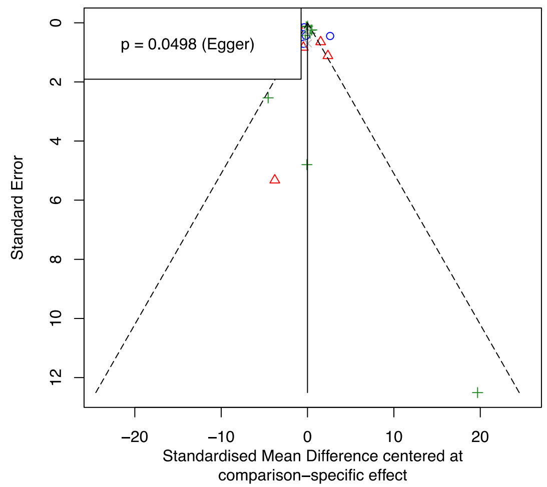

ICU LENGTH OF STAY

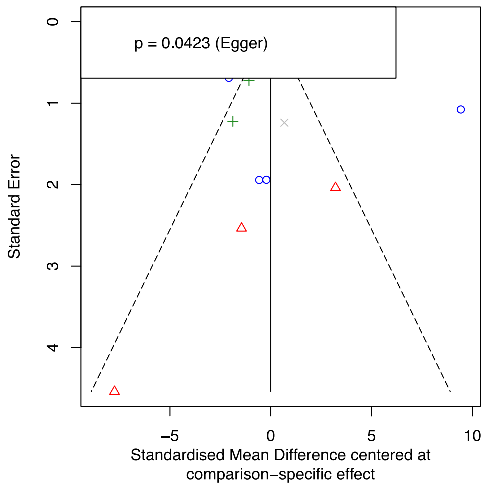

HOSPITAL LENGTH OF STAY

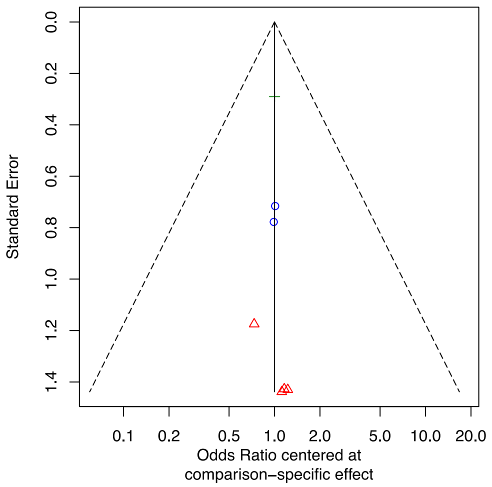

ICU MORTALITY

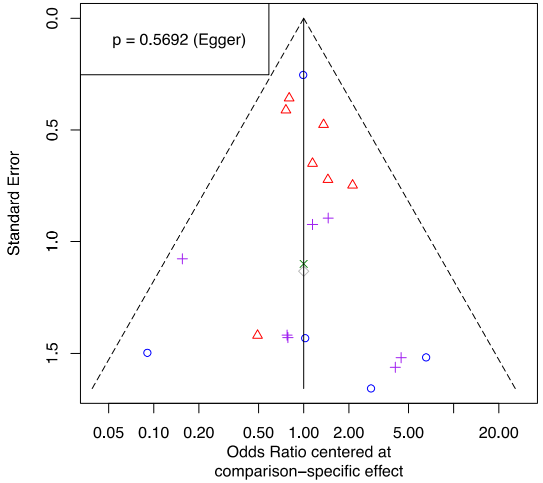

HOSPITAL MORTALITY

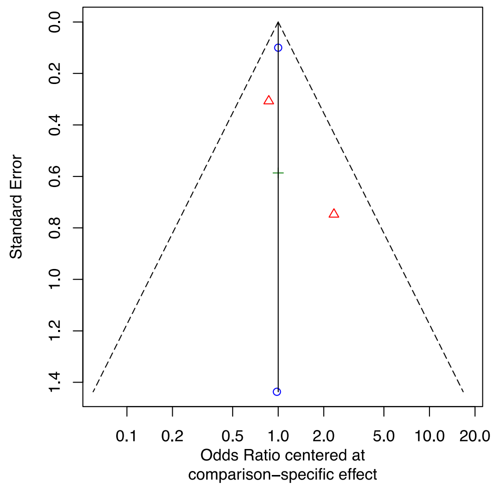

LONG-TERM MORTALITY

Supplementary Digital Content 7. Meta-regression surface under the cumulative ranking curve scatterplots

Scatterplots of the surface under the cumulative ranking curve (SUCRA) values under the network meta-analysis (y-axis) against the SUCRA values under the network meta-regression (x-axis) for a specified level or value of the investigated covariate are depicted. The names of the interventions appear above each point in the plot. Three colored rectangles are drawn in the scatterplot: a red rectangle for SUCRA values up to 50%, a yellow rectangular for SUCRA values between 50% and 80%, and a green rectangle for SUCRA values over 80%. Interventions falling at the green area are considered as the highest ranked interventions, whilst interventions falling at the red area are considered as the lowest ranked interventions. Abbreviations: NRS, non-invasive respiratory support; COT, conventional oxygen therapy; HFNO, high-flow nasal oxygen; CPAP, continuous positive airway pressure; NIV, non-invasive ventilation; ICU, intensive care unit.

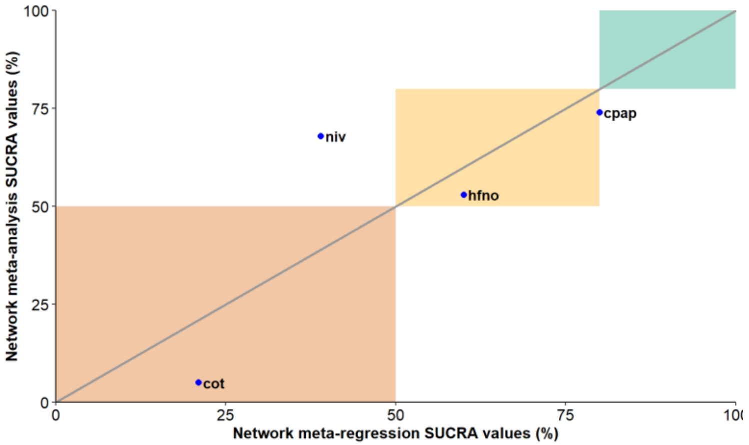

PROPHYLACTIC NRS

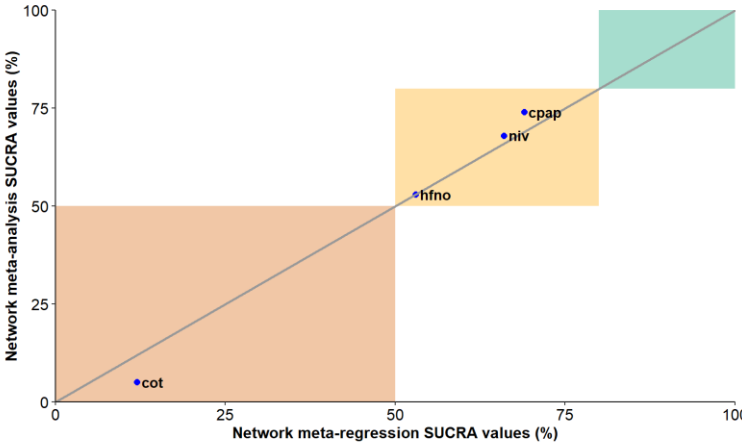

THERAPEUTIC NRS

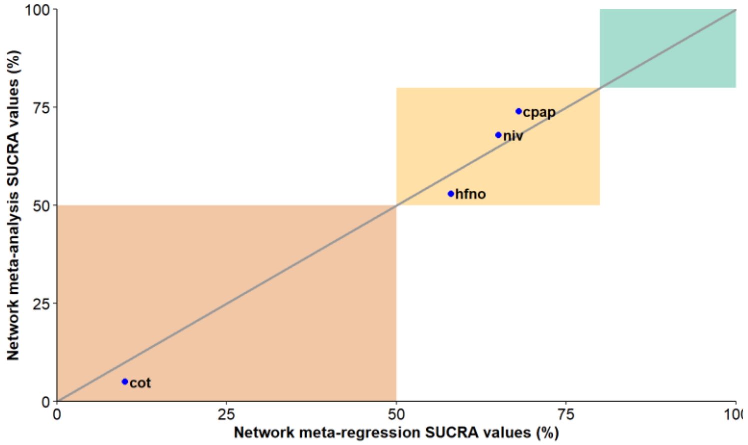

SUPRA-DIAPHRAGMATIC SURGERY

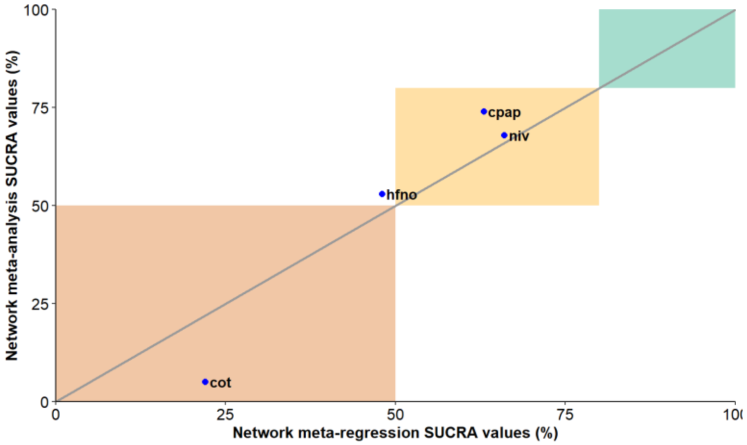

INFRA-DIAPHRAGMATIC SURGERY

.

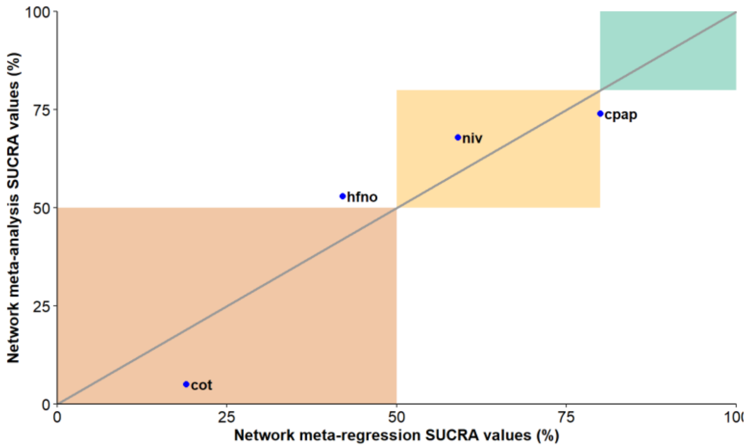

LOW RISK

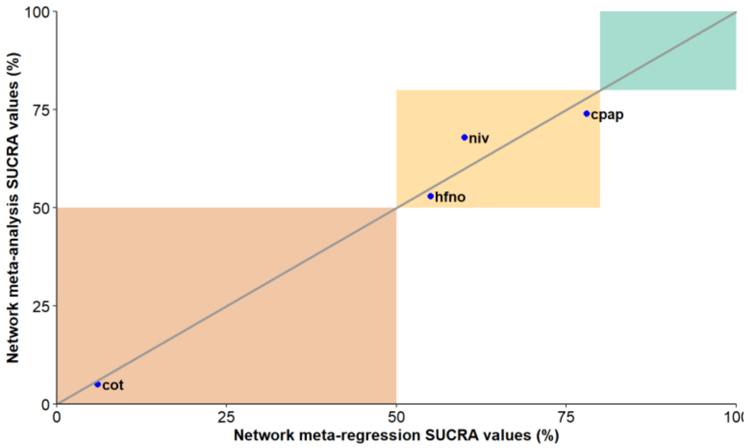

HIGH NRS

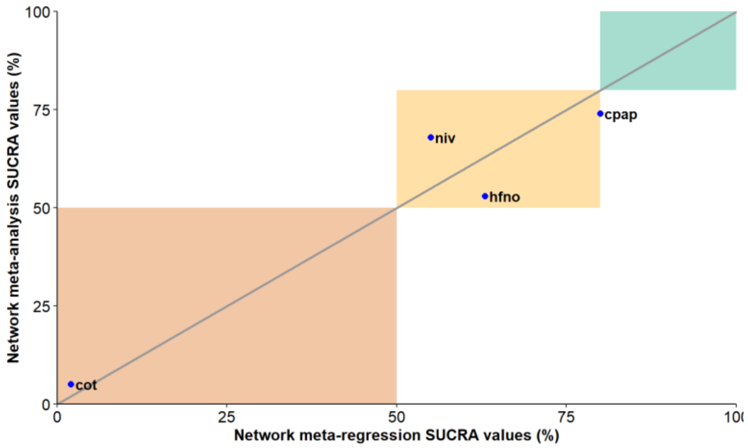

ICU

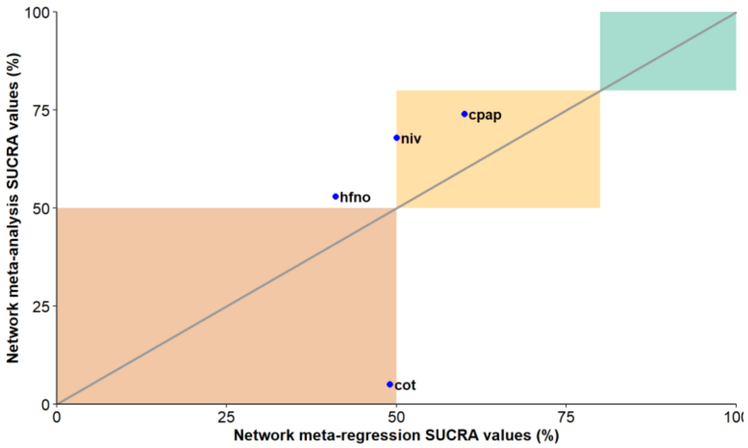

OUT OF THE ICU

Supplementary Digital Content 8. Direct and indirect evidence of the impact of the interventions

**Supplementary Table 5. Direct and indirect evidence of the impact of the interventions on primary and secondary outcomes in the overall population**				
**Comparison**	**Prop**	**Direct (SMD or OR, 95% CI)**	**Indirect (SMD or OR, 95% CI)**	**p-value**[a]
Re-intubation				
HFNO vs. COT	0.76	0.68 [0.31; 1.50]	0.70 [0.17; 2.83]	0.975
CPAP vs. COT	0.85	0.47 [0.20; 1.09]	0.60 [0.08; 4.47]	0.829
NIV vs. COT	0.78	0.51 [0.27; 0.96]	0.45 [0.13; 1.52]	0.871
CPAP vs. HFNO	0	-	0.71 [0.26; 1.98]	-
HFNO vs. NIV	0.39	1.41 [0.40; 4.96]	1.38 [0.51; 3.75]	0.975
CPAP vs. NIV	0.22	1.19 [0.17; 8.15]	0.94 [0.34; 2.63]	0.829
Nosocomial pneumonia				
HFNO vs. COT	0.48	0.68 [0.28; 1.69]	0.61 [0.25; 1.45]	0.853
CPAP vs. COT	0.90	0.59 [0.32; 1.09]	0.50 [0.08; 3.03]	0.867
NIV vs. COT	0.72	0.52 [0.29; 0.95]	0.61 [0.23; 1.59]	0.791
CPAP vs. HFNO	0	-	0.90 [0.39; 2.08]	-
HFNO vs. NIV	0.72	1.14 [0.58; 2.22]	1.28 [0.44; 3.72]	0.853
CPAP vs. NIV	0.18	0.93 [0.17; 5.20]	1.09 [0.49; 2.45]	0.867
Discomfort				
HFNO vs. COT	0.83	0.83 [0.13; 5.48]	15.63 [0.25; 976.62]	0.206
CPAP vs. COT	1.00	3.02 [0.19; 49.12]	-	-

NIV vs. COT	0.66	16.74 [1.18; 236.82]	0.89 [0.02; 35.80]	0.206
CPAP vs. HFNO	0	-	2.19 [0.08; 57.84]	-
HFNO vs. NIV	0.51	0.93 [0.04; 22.33]	0.05 [0.00; 1.28]	0.206
CPAP vs. NIV	0	-	0.49 [0.01; 6.56]	-
ICU length of stay				
HFNO vs. COT	0.87	-0.06 [-0.60; 0.49]	-1.16 [-2.59; 0.26]	0.155
CPAP vs. COT	1.00	-1.39 [-2.04; -0.73]		-
NIV vs. COT	0.67	-1.14 [-2.01; -0.27]	-0.03 [-1.29; 1.22]	0.155
CPAP vs. HFNO	0	-	-1.19 [-2.02; -0.36]	-
HFNO vs. NIV	0.45	-0.02 [-1.15; 1.10]	1.09 [0.06; 2.11]	0.155
CPAP vs. NIV	0	-	-0.61 [-1.58; 0.36]	-
Hospital length of stay				
HFNO vs. COT	0.93	-0.88 [-2.04; 0.28]	-3.57 [-7.80; 0.65]	0.228
CPAP vs. COT	1.00	-2.58 [-3.99; -1.17]	-	-
NIV vs. COT	0.37	-3.45 [-6.92; 0.02]	-0.76 [-3.43; 1.91]	0.228
CPAP vs. HFNO	0	-	-1.51 [-3.31; 0.29]	-
HFNO vs. NIV	0.70	-0.12 [-2.53; 2.28]	2.57 [-1.09; 6.23]	0.228
CPAP vs. NIV	0	-	-0.82 [-3.36; 1.72]	-
ICU mortality				
HFNO vs. COT	0.44	1.12 [0.30; 4.20]	0.31 [0.10; 1.02]	0.160
CPAP vs. COT	-	-	-	-
NIV vs. COT	0.66	0.25 [0.09; 0.71]	0.90 [0.21; 3.81]	0.160

CPAP vs. HFNO	-	-	-	-
HFNO vs. NIV	0.90	1.24 [0.70; 2.19]	4.43 [0.83; 23.74]	0.160
CPAP vs. NIV	-	-	-	-
Hospital mortality				
HFNO vs. COT	0.87	0.78 [0.33; 1.86]	2.14 [0.23; 20.39]	0.414
CPAP vs. COT	0.96	0.90 [0.56; 1.44]	0.35 [0.04; 3.14]	0.411
NIV vs. COT	0.94	0.50 [0.34; 0.57]	0.53 [0.10; 2.65]	0.962
CPAP vs. HFNO	0	-	0.97 [0.38; 2.45]	-
HFNO vs. NIV	0.15	4.13 [0.45; 38.02]	1.51 [0.58; 3.91]	0.414
CPAP vs. NIV	0.07	0.72 [0.08; 6.18]	1.83 [0.99; 3.38]	0.411
Long-term mortality				
HFNO vs. COT	0	-	0.65 [0.18; 2.33]	-
CPAP vs. COT	1.00	0.92 [0.76; 1.12]	-	-
NIV vs. COT	1.00	0.56 [0.32; 0.97]	-	-
CPAP vs. HFNO	0	-	1.42 [0.39; 5.16]	-
HFNO vs. NIV	1.00	1.17 [0.37; 3.68]	-	-
CPAP vs. NIV	0	-	1.65 [0.92; 2.98]	-

Abbreviations: Prop, direct evidence proportion; SMD, standardized mean difference; OR, odds ratio; CI, confidence interval; HFNO, high-low nasal oxygen; COT, conventional oxygen therapy; CPAP, continuous positive airway pressure; NIV, non-invasive ventilation; ICU, intensive care unit.

[a]p-value is the significance level for network incoherence.

Supplementary Figure 2. Direct and indirect evidence of the impact of the interventions on primary and secondary outcomes in the overall population.

Abbreviations: OR, odds ratio; CI, confidence interval; CPAP, continuous positive airway pressure; COT, conventional oxygen therapy; HFNO, high-flow nasal oxygen; NIV, non-invasive ventilation; ICU, intensive care unit; SMD, standardized mean difference.

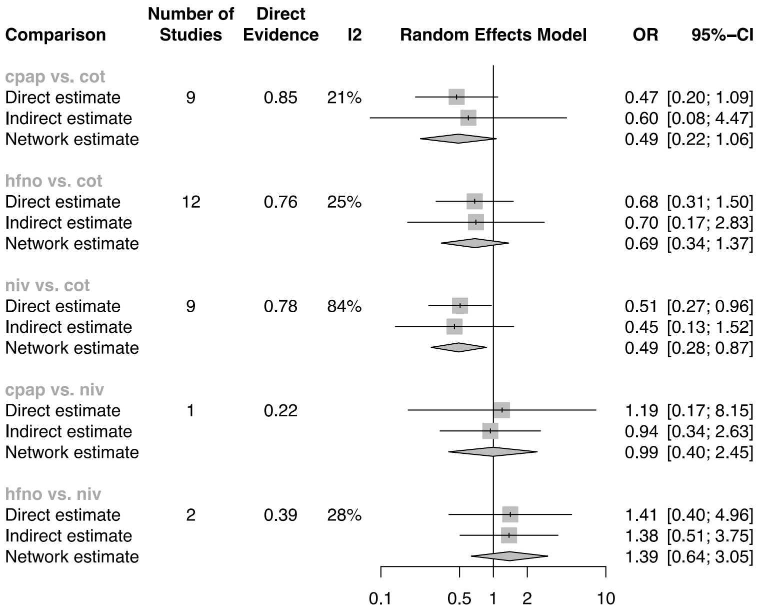

RE-INTUBATION

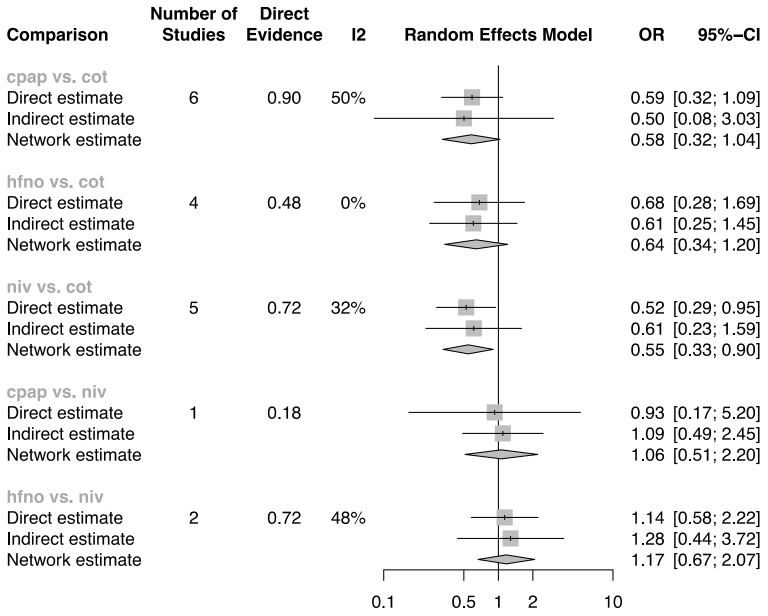

NOSOCOMIAL PNEUMONIA

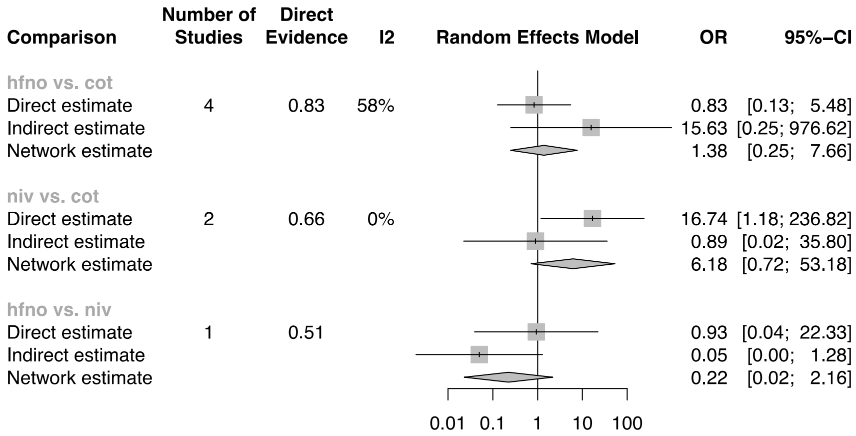

DISCOMFORT

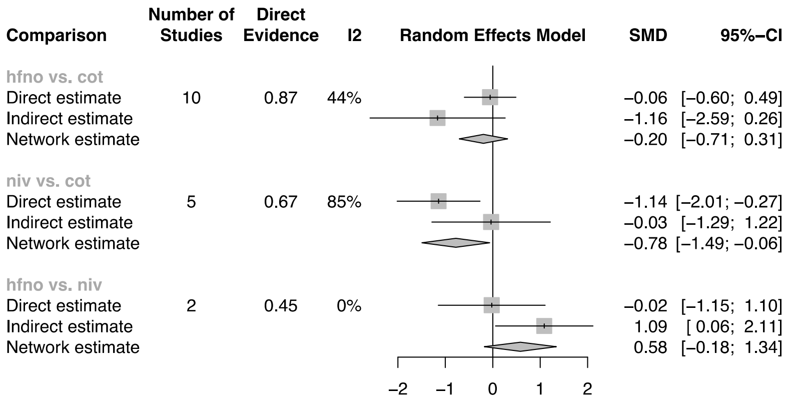

ICU LENGTH OF STAY

Comparison	Number of Studies	Direct Evidence	I2	SMD	95%−CI
**hfno vs. cot**					
Direct estimate	7	0.93	92%	−0.88	[−2.04; 0.28]
Indirect estimate				−3.57	[−7.80; 0.65]
Network estimate				−1.07	[−2.19; 0.05]
**niv vs. cot**					
Direct estimate	3	0.37	65%	−3.45	[−6.92; 0.02]
Indirect estimate				−0.76	[−3.43; 1.91]
Network estimate				−1.76	[−3.87; 0.36]
**hfno vs. niv**					
Direct estimate	2	0.70	0%	−0.12	[−2.53; 2.28]
Indirect estimate				2.57	[−1.09; 6.23]
Network estimate				0.69	[−1.32; 2.70]

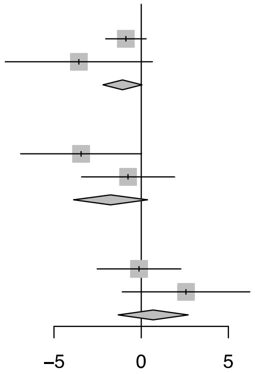

HOSPITAL LENGTH OF STAY

Comparison	Number of Studies	Direct Evidence	I2	OR	95%−CI
**hfno vs. cot**					
Direct estimate	4	0.44	0	1.12	[0.30; 4.20]
Indirect estimate				0.31	[0.10; 1.02]
Network estimate				0.55	[0.23; 1.32]
**niv vs. cot**					
Direct estimate	2	0.66	0	0.25	[0.09; 0.71]
Indirect estimate				0.90	[0.21; 3.81]
Network estimate				0.39	[0.17; 0.90]
**hfno vs. niv**					
Direct estimate	1	0.90		1.24	[0.70; 2.19]
Indirect estimate				4.43	[0.83; 23.74]
Network estimate				1.41	[0.82; 2.42]

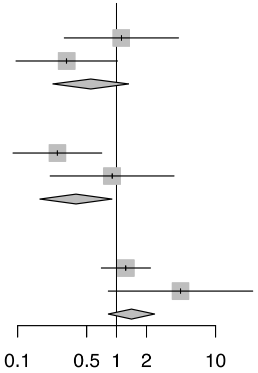

ICU MORTALITY

**Supplementary Table 6. Direct and indirect evidence of the impact of the interventions on re-intubation in sensitivity analyses**				
**Comparison**	**Prop**	**Direct (SMD or OR, 95% CI)**	**Indirect (SMD or OR, 95% CI)**	**p-value**[a]
Excluding studies investigating a sequential combination of NRS modalities				
HFNO vs. COT	0.84	0.68 [0.30; 1.51]	0.71 [0.17; 2.98]	0.960
CPAP vs. COT	0.79	0.51 [0.19; 1.38]	0.60 [0.08; 4.67]	0.890
NIV vs. COT	0.77	0.51 [0.26; 0.98]	0.46 [0.13; 1.61]	0.898
CPAP vs. HFNO	0	-	0.77 [0.25; 2.35]	-
HFNO vs. NIV	0.29	1.42 [0.39; 5.17]	1.36 [0.49; 3.78]	0.890
CPAP vs. NIV	0.30	1.19 [0.17; 8.51]	1.02 [0.32; 3.27]	0.960
Excluding studies requiring the application of the continuity correction				
HFNO vs. COT	0.75	0.66 [0.29; 1.51]	0.70 [0.17; 2.93]	0.949
CPAP vs. COT	0.84	0.45 [0.18; 1.09]	0.60 [0.08; 4.63]	0.800
NIV vs. COT	0.78	0.51 [0.26; 0.97]	0.43 [0.12; 1.51]	0.830
CPAP vs. HFNO	0	-	0.70 [0.24; 2.03]	-
HFNO vs. NIV	0.39	1.42 [0.39; 5.14]	1.35 [0.48; 3.80]	0.949
CPAP vs. NIV	0.23	1.19 [0.17; 8.47]	0.89 [0.31; 2.62]	0.800
Excluding studies at high risk of bias				

HFNO vs. COT	0.81	0.90 [0.36; 2.27]	0.23 [0.03; 1.51]	0.199
CPAP vs. COT	1.00	0.48 [0.20; 1.17]	-	-
NIV vs. COT	0.80	0.22 [0.09; 0.57]	0.88 [0.13; 5.79]	0.199
CPAP vs. HFNO	0	-	0.69 [0.21; 2.33]	-
HFNO vs. NIV	0.39	1.03 [0.20; 5.30]	4.08 [1.09; 15.24]	0.199
CPAP vs. NIV	0	-	1.65 [0.48; 5.60]	-
HFNO 40 L/min in Theologou et al. and NIV with mask in Yang et al.				
HFNO vs. COT	0.76	0.71 [0.32; 1.58]	0.75 [0.18; 3.08]	0.949
CPAP vs. COT	0.85	0.47 [0.20; 1.10]	0.64 [0.08; 4.87]	0.783
NIV vs. COT	0.78	0.54 [0.28; 1.04]	0.46 [0.14; 1.57]	0.820
CPAP vs. HFNO	0	-	0.68 [0.24; 1.92]	-
HFNO vs. NIV	0.39	1.42 [0.40; 5.05]	1.34 [0.49; 3.70]	0.949
CPAP vs. NIV	0.22	1.19 [0.17; 8.30]	0.88 [0.31; 2.48]	0.783
HFNO 40 L/min in Theologou et al. NIV with helmet in Yang et al.				
HFNO vs. COT	0.76	0.71 [0.31; 1.59]	0.65 [0.15; 2.80]	0.922

CPAP vs. COT	0.85	0.47 [0.20; 1.11]	0.56 [0.07; 4.48]	0.875
NIV vs. COT	0.77	0.46 [0.23; 0.91]	0.46 [0.13; 1.61]	0.987
CPAP vs. HFNO	0	-	0.69 [0.24; 1.98]	-
HFNO vs. NIV	0.39	1.42 [0.38; 5.28]	1.55 [0.55; 4.37]	0.922
CPAP vs. NIV	0.22	1.19 [0.16; 8.70]	1.00 [0.34; 2.89]	0.875
HFNO 60 L/min in Theologou et al. NIV with mask in Yang et al.				
HFNO vs. COT	0.75	0.62 [0.28; 1.39]	0.75 [0.19; 3.00]	0.827
CPAP vs. COT	0.85	0.47 [0.20; 1.09]	0.63 [0.08; 4.64]	0.797
NIV vs. COT	0.78	0.54 [0.29; 1.03]	0.43 [0.13; 1.43]	0.727
CPAP vs. HFNO	0	-	0.75 [0.27; 2.10]	-
HFNO vs. NIV	0.39	1.41 [0.40; 4.92]	1.18 [0.43; 3.24]	0.827
CPAP vs. NIV	0.22	1.19 [0.18; 8.08]	0.90 [0.32; 2.50]	0.797
HFNO 60 L/min in Theologou et al. and NIV with helmet in Yang et al				
HFNO vs. COT	0.76	0.62 [0.28; 1.40]	0.65 [0.15; 2.73]	0.959
CPAP vs. COT	0.85	0.47 [0.20; 1.10]	0.55 [0.07; 4.27]	0.889

NIV vs. COT	0.77	0.46 [0.24; 0.91]	0.42 [0.12; 1.46]	0.895
CPAP vs. HFNO	0	-	0.76 [0.27; 2.17]	-
HFNO vs. NIV	0.39	1.42 [0.39; 5.14]	1.36 [0.48; 3.82]	0.959
CPAP vs. NIV	0.22	1.19 [0.17; 8.46]	1.02 [0.36; 2.92]	0.889
Consideration of those studies comparing either CPAP or NIV to COT in the same group				
HFNO vs. COT	0.73	0.69 [0.32; 1.48]	0.69 [0.20; 2.43]	0.998
CPAP/NIV vs. COT	0.89	0.50 [0.31; 0.80]	0.50 [0.12; 1.99]	0.998
CPAP/NIV vs. HFNO	0.38	0.72 [0.23; 2.30]	0.72 [0.29; 1.77]	0.998

Abbreviations: Prop, direct evidence proportion; SMD, standardized mean difference; OR, odds ratio; CI, confidence interval; NRS, non-invasive respiratory support; HFNO, high-flow nasal oxygen; COT, conventional oxygen therapy; CPAP, continuous positive airway pressure; NIV, non-invasive ventilation.

[a]p-value is the significance level for network incoherence.

**Supplementary Table 7. Direct and indirect evidence of the impact of the interventions on re-intubation in patient subgroups**				
**Comparison**	**Prop**	**Direct (SMD or OR, 95% CI)**	**Indirect (SMD or OR, 95% CI)**	**p-value[a]**
Prophylactic intervention				
HFNO vs. COT	0.86	0.65 [0.28; 1.54]	4.55 [0.53; 39.19]	0.100
CPAP vs. COT	1.00	0.53 [0.22; 1.27]	-	-
NIV vs. COT	0.83	1.86 [0.71; 4.83]	0.27 [0.03; 2.20]	0.100
CPAP vs. HFNO	0	-	0.62 [0.19; 2.02]	-
HFNO vs. NIV	0.31	2.45 [0.36; 16.88]	0.35 [0.10; 1.27]	0.100
CPAP vs. NIV	0	-	0.39 [0.11; 1.36]	-
Therapeutic intervention				
HFNO vs. COT	1.00	0.91 [0.15; 5.65]	-	-
CPAP vs. COT	0.61	0.39 [0.06; 2.59]	0.27 [0.02; 2.95]	0.814
NIV vs. COT	0.9	0.22 [0.09; 0.59]	0.32 [0.02; 5.94]	0.814
CPAP vs. HFNO	0	-	0.37 [0.03; 3.87]	-
HFNO vs. NIV	0	-	3.93 [0.51; 30.24]	-
CPAP vs. NIV	0.48	1.19 [0.13; 10.79]	1.72 [0.20; 14.55]	0.814
High risk patients				
HFNO vs. COT	0.59	0.50 [0.13; 1.94]	0.51 [0.10; 2.63]	0.978
CPAP vs. COT	1.00	0.54 [0.05; 5.78]	-	-
NIV vs. COT	0.73	0.36 [0.12; 1.10]	0.35 [0.06; 2.20]	0.978
CPAP vs. HFNO	0	-	1.08 [0.08; 14.41]	-

HFNO vs. NIV	0.68	1.40 [0.42; 4.72]	1.36 [0.23; 7.89]	0.978
CPAP vs. NIV	0	-	1.50 [0.12; 19.22]	-
Low risk patients				
HFNO vs. COT	1.00	0.81 [0.29; 2.23]	-	-
CPAP vs. COT	0.85	0.45 [0.17; 1.19]	0.72 [0.07; 7.02]	0.713
NIV vs. COT	0.88	0.60 [0.25; 1.43]	0.38 [0.04; 3.85]	0.713
CPAP vs. HFNO	0	-	0.60 [0.15; 2.32]	-
HFNO vs. NIV	0	-	1.43 [0.39; 5.25]	-
CPAP vs. NIV	0.28	1.19 [0.14; 9.86]	0.75 [0.20; 2.77]	0.713
Supra-diaphragmatic surgery				
HFNO vs. COT	0.83	0.60 [0.23; 1.60]	0.59 [0.07; 5.11]	0.989
CPAP vs. COT	0.59	0.43 [0.06; 3.29]	0.71 [0.06; 7.92]	0.759
NIV vs. COT	0.80	0.60 [0.25; 1.45]	0.50 [0.08; 2.97]	0.855
CPAP vs. HFNO	0	-	0.89 [0.15; 5.16]	-
HFNO vs. NIV	0.30	1.03 [0.14; 7.47]	1.04 [0.29; 3.81]	0.989
CPAP vs. NIV	0.48	1.19 [0.12; 11.51]	0.73 [0.08; 6.47]	0.759
Infra-diaphragmatic surgery				
HFNO vs. COT	0.67	1.17 [0.27; 5.11]	1.45 [0.17; 12.17]	0.868
CPAP vs. COT	1.00	0.59 [0.25; 1.35]	-	-
NIV vs. COT	0.79	0.59 [0.18; 1.93]	0.48 [0.05; 4.76]	0.868
CPAP vs. HFNO	0	-	0.47 [0.11; 2.04]	-
HFNO vs. NIV	0.53	2.45 [0.42; 14.37]	1.97 [0.30; 13.04]	0.868

CPAP vs. NIV	0	-	1.04 [0.27; 3.96]	-
In ICU				
HFNO vs. COT	0.77	0.54 [0.19; 1.53]	0.49 [0.07; 3.19]	0.919
CPAP vs. COT	0.69	0.24 [0.06; 1.05]	0.60 [0.07; 5.31]	0.494
NIV vs. COT	0.84	0.50 [0.25; 1.01]	0.36 [0.07; 1.78]	0.710
CPAP vs. HFNO	0	-	0.61 [0.14; 2.72]	-
HFNO vs. NIV	0.33	1.03 [0.18; 5.95]	1.15 [0.33; 3.94]	0.919
CPAP vs. NIV	0.38	1.19 [0.15; 9.46]	0.48 [0.10; 2.38]	0.494
Out of ICU				
CPAP vs. COT	1.00	0.46 [0.08; 2.72]	-	-

Abbreviations: Prop, direct evidence proportion; SMD, standardized mean difference; OR, odds ratio; CI, confidence interval; HFNO, high-flow nasal oxygen; COT, conventional oxygen therapy; CPAP, continuous positive airway pressure; NIV, non-invasive ventilation; ICU, intensive care unit.

[a]p-value is the significance level for network incoherence.

**Supplementary Table 8. Direct and indirect evidence of the impact of the interventions on re-intubation in patient subgroups when considering those studies comparing either CPAP or NIV to COT in the same group**				
**Comparison**	**Prop**	**Direct (SMD or OR, 95% CI)**	**Indirect (SMD or OR, 95% CI)**	**p-value**[a]
Prophylactic intervention				
HFNO vs. COT	0.84	0.69 [0.31; 1.53]	2.34 [0.38; 14.40]	0.226
CPAP/NIV vs. COT	0.92	0.95 [0.55; 1.65]	0.28 [0.04; 1.89]	0.226
CPAP/NIV vs. HFNO	0.24	0.41 [0.07; 2.30]	1.39 [0.52; 3.69]	0.226
Therapeutic intervention				
HFNO vs. COT	1.00	0.91 [0.15; 5.74]	-	-
CPAP/NIV vs. COT	1.00	0.25 [0.11; 0.60]	-	-
CPAP/NIV vs. HFNO	0	-	0.27 [0.04; 2.08]	-
High risk patients				
HFNO vs. COT	0.56	0.52 [0.14; 1.94]	0.54 [0.12; 2.41]	0.958
CPAP/NIV vs. COT	0.77	0.39 [0.15; 1.03]	0.37 [0.07; 2.14]	0.958
CPAP/NIV vs. HFNO	0.67	0.72 [0.23; 2.25]	0.76 [0.15; 3.91]	0.958

Low risk patients				
HFNO vs. COT	1.00	0.80 [0.31; 2.09]	-	-
CPAP/NIV vs. COT	1.00	0.53 [0.30; 0.96]	-	-
CPAP/NIV vs. HFNO	0	-	0.66 [0.22; 2.04]	-
Supra-diaphragmatic surgery				
HFNO vs. COT	0.83	0.60 [0.23; 1.60]	0.58 [0.07; 5.00]	0.981
CPAP/NIV vs. COT	0.88	0.57 [0.25; 1.28]	0.59 [0.06; 5.38]	0.981
CPAP/NIV vs. HFNO	0.29	0.97 [0.13; 7.13]	0.95 [0.27; 3.38]	0.981
Infra-diaphragmatic surgery				
HFNO vs. COT	0.61	1.27 [0.35; 4.60]	1.60 [0.32; 7.99]	0.829
CPAP/NIV vs. COT	0.95	0.65 [0.40; 1.05]	0.52 [0.07; 3.84]	0.829
CPAP/NIV vs. HFNO	0.44	0.41 [0.09; 1.89]	.51 [0.13; 2.01]	0.829
In ICU				
HFNO vs. COT	0.77	0.54 [0.19; 1.53]	0.45 [0.07; 2.98]	0.869
CPAP/NIV vs. COT	0.91	0.44 [0.23; 0.83]	0.53 [0.07; 4.14]	0.869

CPAP/NIV vs. HFNO	0.32	0.97 [0.16; 5.76]	0.81 [0.24; 2.75]	0.869
Out of ICU				
CPAP/NIV vs. COT	1.00	0.46 [0.08; 2.72]	-	-

Abbreviations: Prop, direct evidence proportion; SMD, standardized mean difference; OR, odds ratio; CI, confidence interval; HFNO, high-flow nasal oxygen; COT, conventional oxygen therapy; CPAP, continuous positive airway pressure; NIV, non-invasive ventilation; ICU, intensive care unit.

[a]p-value is the significance level for network incoherence.

Supplementary Digital Content 9. Grades of Recommendation, Assessment, Development and Evaluation (GRADE) assessment

Outcome			Direct evidence				Direct rating		Indirect evidence		Indirect rating	Network				Certainty	
			ROB	Inconsistency	Indirectness	Publication			Lowest rating	Intransitivity		Proportion	Rating of most contributing evidence	Incoherence	Imprecision	Partial	Final
Re-intubation	HFNO vs. COT	0.69 [0.34; 1.37]	1[ab]	0	0	0	1	Moderate	Low	0	Low	0.76	Direct	0	2[c]	Moderate	Very low
	CPAP vs. COT	0.49 [0.22; 1.06]	1[d]	0	0		1	Moderate	Low	0	Low	0.85	Direct	0	2[e]	Moderate	Very low
	NIV vs. COT	0.49 [0.28; 0.87]	2[f]	0	0		2	Low	Moderate	0	Moderate	0.78	Direct	0	0	Low	Low
	CPAP vs. HFNO	0.71 [0.26; 1.98]	-	-	-		-	-	Moderate	0	Moderate	0	Indirect	0	2[c]	Moderate	Very low
	HFNO vs. NIV	1.39 [0.64; 3.05]	1[g]	0	0		1	Moderate	Low	0	Low	0.39	Indirect	0	2[h]	Low	Very low
	CPAP vs. NIV	0.99 [0.40; 2.45]	2[i]	0	0		2	Low	Low	0	Low	0.22	Indirect	0	2[j]	Low	Very low
Nosocomial pneumonia	HFNO vs. COT	0.64 [0.34; 1.20]	1[k]	0	0	1[l]	2	Low	Low	0	Low	0.48	Indirect	0	2[c]	Low	Very low
	CPAP vs. COT	0.58 [0.32; 1.04]	1[m]	0	0		2	Low	Very low	0	Very low	0.90	Direct	0	2[e]	Low	Very low
	NIV vs. COT	0.55 [0.33; 0.90]	1[n]	0	0		2	Low	Very low	0	Very low	0.72	Direct	0	0	Low	Low

	CPAP vs. HFNO	0.90 [0.39; 2.08]	-	-	-		-	-	Low	0	Low	0	Indirect	0	2[j]	Low	Very low
	HFNO vs. NIV	1.17 [0.67; 2.07]	1[g]				2	Low	Low	0	Low	0.72	Direct	0	2[h]	Low	Very low
	CPAP vs. NIV	1.06 [0.51; 2.20]	2[i]	0	0		3	Very low	Low	0	Low	0.18	Indirect	0	2[j]	Low	Very low
Discomfort	HFNO vs. COT	1.38 [0.25; 7.66]	1[o]	0	0	1[p]	2	Low	Very low	0	Very low	0.83	Direct	0	2[h]	Low	Very low
	CPAP vs. COT	3.02 [0.19; 49.12]	1[q]	0	0		2	Low	Low	0	Low	1.00	Direct	-	2[h]	Low	Very low
	NIV vs. COT	6.18 [0.72; 53.18]	2[r]	0	0		3	Very low	Low	0	Low	0.66	Direct	0	2[h]	Very low	Very low
	CPAP vs. HFNO	2.19 [0.08; 57.84]	-	-	-		-	-	Low	0	Low	0	Indirect	-	2[h]	Low	Very low
	HFNO vs. NIV	0.22 [0.02; 2.16]	0	0	0		1	Moderate	Very low	0	Very low	0.51	Direct	0	2[c]	Moderate	Very low
	CPAP vs. NIV	0.49 [0.01; 16.56]	-	-	-		-	-	Very low	0	Very low	0	Indirect	-	2[e]	Very low	Very low
ICU length of stay	HFNO vs. COT	-0.20 [-0.71; 0.31]	1[s]	1[t]	0	1[u]	3	Very low	Very low	0	Very low	0.87	Direct	0	2[e]	Very low	Very low
	CPAP vs. COT	-1.39 [-2.04; -0.73]	1[v]	0	0		2	Low	Low	0	Low	1.00	Direct	-	2[e]	Low	Very low

	NIV vs. COT	-0.78 [-1.49; -0.06]	0	0	0		1	Moderate	Very low	0	Very low	0.67	Direct	0	2[e]	Moderate	Very low
	CPAP vs. HFNO	-1.19 [-2.02; -0.36]	-	-	-		-	-	Very low	0	Very low	0	Indirect	-	2[e]	Very low	Very low
	HFNO vs. NIV	0.58 [-0.18; 1.34]	1[g]	0	0		2	Low	Very low	0	Very low	0.45	Indirect	0	2[h]	Very low	Very low
	CPAP vs. NIV	-0.61 [-1.58; 0.36]	-	-	-		-	-	Low	-	Low	0	Indirect	-	2[e]	Low	Very low
Hospital length of stay	HFNO vs. COT	-1.07 [-2.19; 0.05]	1[w]	0	0	1[x]	2	Low	Very low	0	Very low	0.9	Direct	0	2[e]	Low	Very low
	CPAP vs. COT	-2.58 [-3.99; -1.17]	1[k]	0	0		2	Low	Low	0	Low	1.00	Direct	-	2[e]	Low	Very low
	NIV vs. COT	-1.76 [-3.87; 0.36]	2[y]	0	0		3	Very low	Low	0	Low	0.37	Indirect	0	2[e]	Low	Very low
	CPAP vs. HFNO	-1.51 [-3.31; 0.29]	-	-	-		-	-	Low	0	Low	0	Indirect	-	2[e]	Low	Very low
	HFNO vs. NIV	0.69 [-1.32; 2.70]	1[g]	0	0		2	Low	Very low	0	Very low	0.70	Direct	0	2[h]	Low	Very low
	CPAP vs. NIV	-0.82 [-3.36; 1.72]	-	-	-		-	-	Very low	0	Very low	0	Indirect	-	2[e]	Very low	Very low
Icu	HFNO vs. COT	0.55 [0.23; 1.32]	1[z]	0	0	0	1	Moderate	Moderate	0	Moderate	0.44	Indirect	0	2[c]	Moderate	Very low

	CPAP vs. COT	-	-	-	-	-	-	-	-	0	-	-	-	-	-	-	-
	NIV vs. COT	0.39 [0.17; 0.90]	0	1[^]	0	0	1	Moderate	Moderate	0	Moderate	0.66	Direct	0	0	Moderate	Moderate
	CPAP vs. HFNO	-	-	-	-	-	-	-	-	0	-	-	-	-	-	-	-
	HFNO vs. NIV	1.41 [0.82; 2.42]	0	0	0	0	0	High	Moderate	0	Moderate	0.90	Direct	0	2[h]	High	Low
	CPAP vs. NIV	-	-	-	-	-	-	-	-	0	-	-	-	-	-	-	-
Hospital mortality	HFNO vs. COT	0.89 [0.40; 2.01]	2[^^]	0	0	0	2	Low	Low	0	Low	0.87	Direct	0	2[c]	Low	Very low
	CPAP vs. COT	0.86 [0.54; 1.37]	2[^^^]	0	0	0	2	Low	Low	0	Low	0.96	Direct	0	2[c]	Low	Very low
	NIV vs. COT	0.51 [0.34; 0.74]	2[*]	0	0	0	2	Low	Low	0	Low	0.94	Direct	0	0	Low	Low
	CPAP vs. HFNO	0.97 [0.38; 2.45]	-	-	-	-	-	-	Low	0	Low	0	Indirect	0	2[c]	Low	Very low
	HFNO vs. NIV	1.75 [0.72; 4.20]	2[**]	0	0	0	2	Low	Low	0	Low	0.15	Indirect	0	2[h]	Low	Very low
	CPAP vs. NIV	1.69 [0.93; 3.07]	2[***]	0	0	0	2	Low	Low	0	Low	0.07	Indirect	0	2[h]	Low	Very low
Long-	HFNO vs. COT	0.65 [0.18; 2.33]	-	-	-	-	-	-	Low	0	Low	0	Indirect	-	2[c]	Low	Very low

	CPAP vs. COT	0.92 [0.76; 1.12]	1[#]	0	0	0	1	Moderate	Moderate	0	Moderate	1.00	Direct	-	2[e]	Moderate	Very low
	NIV vs. COT	0.56 [0.32; 0.97]	1[g]	0	0	0	1	Moderate	Moderate	0	Moderate	1.00	Direct	-	0	Moderate	Moderate
	CPAP vs. HFNO	1.42 [0.39; 5.16]	-	-	-	-	-	-	Low	0	Low	0	Indirect	-	2[h]	Low	Very low
	HFNO vs. NIV	1.17 [0.37; 3.68]	2[**]	0	0	0	2	Low	Low	0	Low	1.00	Direct	-	2[h]	Low	Very low
	CPAP vs. NIV	1.65 [0.92; 2.98]	-	-	-	-	-	-	Moderate	0	Moderate	0	Indirect	-	2[h]	Moderate	Very low

[a]Numbers represent the degree of downrating of certainty of evidence.
[b]Lowered one level for risk of bias in RCTs (some concerns in 6 out of 10 studies) and non-RCTs (serious risk in 2 out of 2 studies)
[c]The point estimate reflects an important benefit and the boundary of the CI least favorable to the intervention includes the possibility of important harm
[d]Lowered one level for risk of bias in RCTs (some concerns in 7 out of 10 studies and high risk in 1)
[e]The point estimate reflects an important benefit and the boundary of the CI least favorable to the intervention includes the possibility of harm
[f]Lowered two levels for risk of bias in RCTs (some concerns in 3 out of 7 studies and high risk in 3 out of 7 studies) and non-RCTs (critical risk in 2 out of 2 studies)
[g]Lowered one level for risk of bias in RCTs (high risk in 1 out of 2 studies)
[h]The point estimate reflects an important harm and the boundary of the CI most favorable to the intervention includes the possibility of important benefit
[i]Lowered two levels for high risk of bias in 1 non-RCT
[j]The point estimate is consistent with a trivial effect and the CI includes the possibility of both important benefit and important harm
[k]Lowered one level for risk of bias in RCTs (some concerns in 5 out of 6 studies)
[l]The presence of publication bias was strongly suspected from the visual inspection of the funnel plot and confirmed by the Egger's test (p = 0.0207)
[m]Lowered one level for risk of bias in RCTs (some concerns in 4 out of 6 studies and high risk in 1 out of 6 studies)
[n]Lowered one levels for risk of bias in RCTs (some concerns in 3 out of 4 studies) and non-RCT (critical risk in 1 out of 1 study)
[o]Lowered one level for risk of bias in RCTs (some concerns in 2 out of 3 studies) and non-RCT (serious risk in 1 out of 1 study)
[p]The presence of publication bias was strongly suspected from the visual inspection of the funnel plot
[q]Lowered one level for risk of bias in RCTs (some concerns in 1 out of 1 study)
[r]Lowered two levels for risk of bias in RCTs (some concerns in 1 out of 1 study and high risk in 1 out of 1 study)
[s]Lowered one level for risk of bias in RCTs (some concerns in 4 out of 8 studies) and non-RCT (serious risk in 2 out of 2 studies)
[t]Relevant differences in estimates of effect across studies
[u]The presence of publication bias was strongly suspected from the visual inspection of the funnel plot and confirmed by the Egger's test (p = 0.0498)
[v]Lowered one level for risk of bias in RCTs (some concerns in 4 out of 5 studies)
[w]Lowered one level for risk of bias in RCTs (some concerns in 4 out of 6 studies) and non-RCT (serious risk in 1 out of 1 study)
[x]The presence of publication bias was strongly suspected from the visual inspection of the funnel plot and confirmed by the Egger's test (p = 0.0423)
[y]Lowered two levels for risk of bias in RCTs (some concerns in 1 out of 2 studies) and non-RCT (critical risk in 1 out of 1 study)
[z]Lowered one level for risk of bias in RCTs (some concerns in 3 out of 4 studies)
[^]Relevant differences in estimates of effect across studies
[^^]Lowered two levels for risk of bias in RCTs (some concerns in 3 out of 5 studies) and non-RCT (serious risk in 2 out of 2 studies)
[^^^]Lowered two levels for risk of bias in RCTs (some concerns in 3 out of 5 studies and high risk in 1 out of 5 studies)
[*]Lowered two levels for risk of bias in RCTs (some concerns in 3 out of 6 studies and high risk in 2 out of 6 studies) and non-RCT (critical risk in 1 out of 1 study)
[**]Lowered two levels for risk of bias in RCTs (high risk in 1 out of 1 study)
[***]Lowered two levels for risk of bias in non-RCTs (critical risk in 1 out of 1 study)
[#]Lowered one level for risk of bias in RCTs (some concerns in 2 out of 2 studies)
Abbreviations: ROB, risk of bias; COT, conventional oxygen therapy; HFNO, high-flow nasal oxygen; NIV, non-invasive ventilation; CPAP, continuous positive airway pressure; ICU, intensive care unit.

Supplementary Digital Content 10. Forest plots of pairwise comparisons of the effect of non-invasive respiratory support on primary outcome.

Abbreviations: COT, conventional oxygen therapy; OR, Odds ratio; M-H, Mantel-Haenszel; CI, confidence interval; HFNO, high-flow nasal oxygen; CPAP, continuous positive airway pressure; NIV, non-invasive ventilation.

	COT		HFNO			Odds Ratio
Study or Subgroup	Events	Total	Events	Total	Weight	M-H, Random, 95% CI
Burra 2021	1	30	1	30	5.8%	1.00 [0.06, 16.76]
Corley 2015	1	74	0	81	4.6%	3.33 [0.13, 82.93]
Futier 2016	4	112	7	108	17.2%	0.53 [0.15, 1.88]
Parke 2013	0	171	2	169	5.1%	0.20 [0.01, 4.10]
Pennisi 2019	1	48	1	47	5.8%	0.98 [0.06, 16.12]
Sahin 2018	4	50	0	50	5.4%	9.77 [0.51, 186.52]
Soliman 2022	2	40	0	40	5.0%	5.26 [0.24, 113.11]
Theologlu 2021	2	33	7	66	12.9%	0.54 [0.11, 2.78]
Vourc'h 2020	1	43	3	47	8.0%	0.35 [0.03, 3.49]
Xia 2021	8	99	3	78	15.9%	2.20 [0.56, 8.58]
Yan 2021	8	46	1	41	9.0%	8.42 [1.01, 70.56]
Yu 2017	5	54	0	56	5.4%	12.56 [0.68, 232.82]
**Total (95% CI)**		**800**		**813**	**100.0%**	**1.43 [0.68, 3.02]**
Total events	37		25			

Heterogeneity: $Tau^2 = 0.43$; $Chi^2 = 14.90$, df = 11 (P = 0.19); $I^2 = 26\%$

Test for overall effect: Z = 0.95 (P = 0.34)

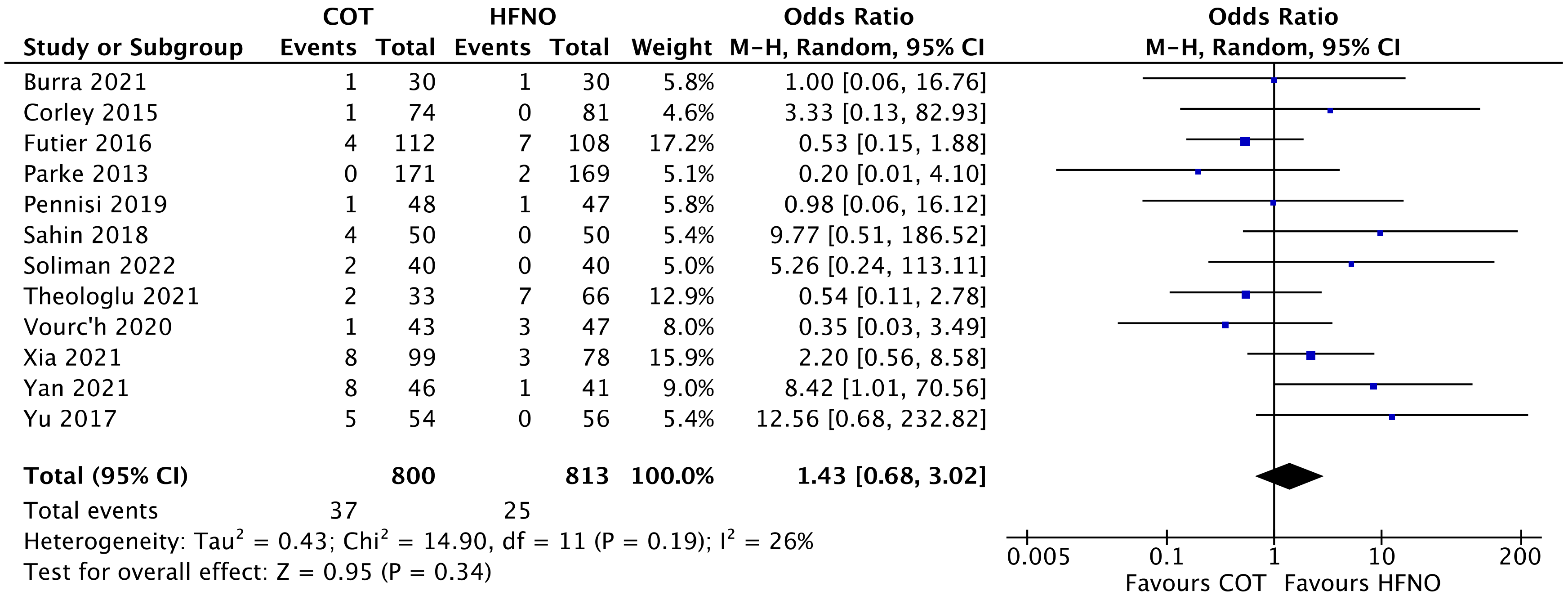

	COT		CPAP			Odds Ratio
Study or Subgroup	Events	Total	Events	Total	Weight	M-H, Random, 95% CI
Bohner 2002	5	105	1	99	7.4%	4.90 [0.56, 42.70]
Gupta 2008	0	20	2	20	3.9%	0.18 [0.01, 4.01]
Hewidy 2016	1	22	0	24	3.5%	3.42 [0.13, 88.40]
Kindgen-Milles 2005	4	25	1	25	6.8%	4.57 [0.47, 44.17]
Olper 2016	1	31	0	33	3.6%	3.30 [0.13, 83.97]
PRISM 2021	90	2397	80	2396	47.3%	1.13 [0.83, 1.53]
Squadrone 2005	10	104	1	105	7.9%	11.06 [1.39, 88.07]
Wong 2011	1	38	1	43	4.7%	1.14 [0.07, 18.79]
Zarbock 2009	6	236	3	232	14.8%	1.99 [0.49, 8.06]
**Total (95% CI)**		**2978**		**2977**	**100.0%**	**1.82 [0.96, 3.43]**
Total events	118		89			

Heterogeneity: $Tau^2 = 0.20$; $Chi^2 = 10.20$, df = 8 (P = 0.25); $I^2 = 22\%$

Test for overall effect: Z = 1.85 (P = 0.06)

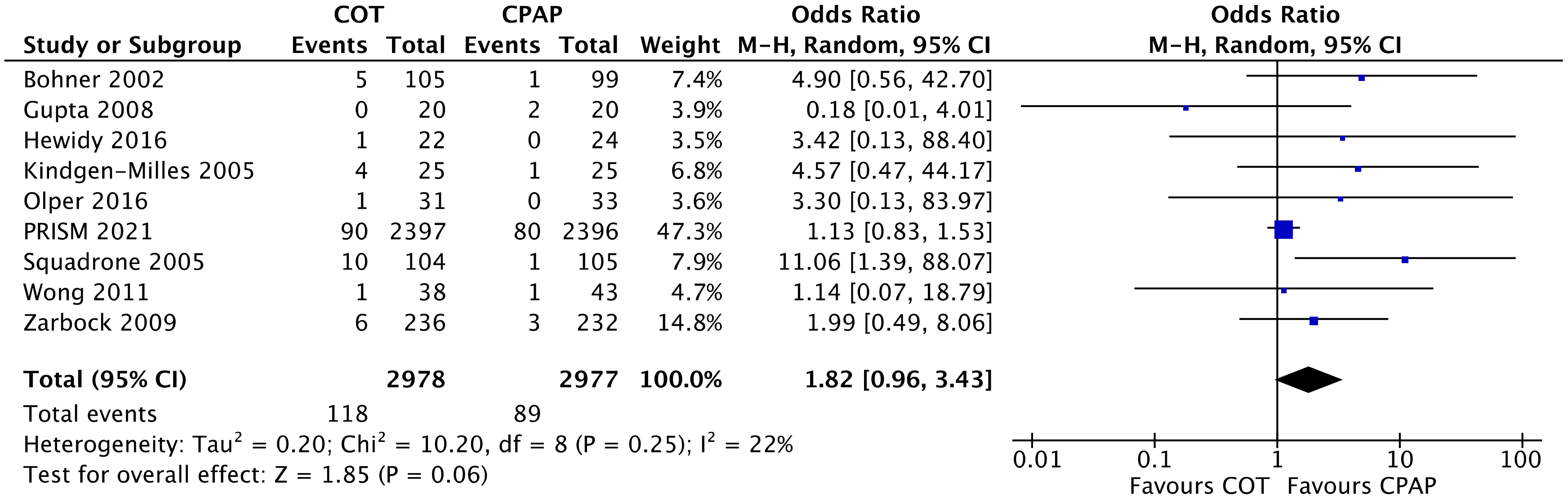

Study or Subgroup	COT Events	COT Total	NIV Events	NIV Total	Weight	Odds Ratio M-H, Random, 95% CI
Abrard 2021	8	128	9	125	12.3%	0.86 [0.32, 2.30]
Antonelli 2000	14	20	4	20	10.1%	9.33 [2.18, 39.96]
Auriant 2001	12	24	5	24	11.0%	3.80 [1.07, 13.52]
Ishikawa 1997	2	12	0	8	4.5%	4.05 [0.17, 96.19]
Jaber 2016	66	145	49	148	14.4%	1.69 [1.05, 2.71]
Melton 2019	34	859	35	859	14.4%	0.97 [0.60, 1.57]
RM Filho 2010	3	27	9	14	9.3%	0.07 [0.01, 0.35]
Yang 2016	9	25	10	50	11.9%	2.25 [0.77, 6.57]
Zhu 2013	38	47	9	48	12.1%	18.30 [6.56, 51.06]
**Total (95% CI)**		**1287**		**1296**	**100.0%**	**1.99 [0.90, 4.41]**
Total events	186		130			

Heterogeneity: $Tau^2 = 1.09$; $Chi^2 = 49.85$, df = 8 ($P < 0.00001$); $I^2 = 84\%$

Test for overall effect: Z = 1.69 (P = 0.09)

Odds Ratio M-H, Random, 95% CI

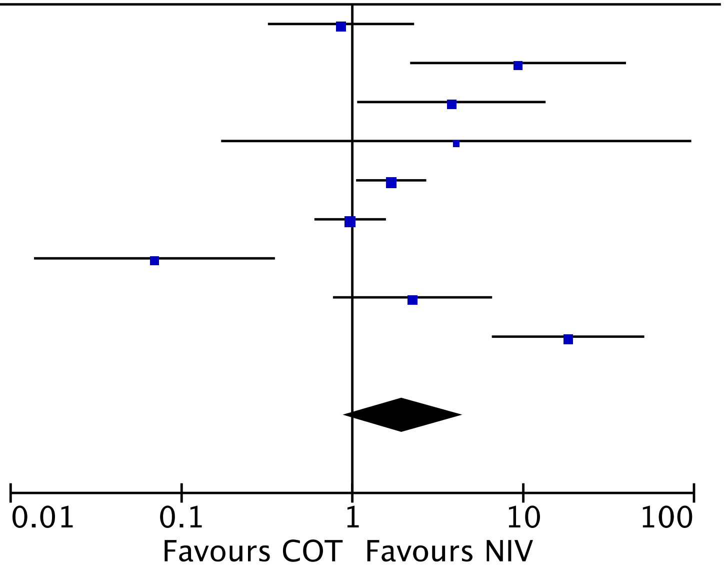

Study or Subgroup	HFNO Events	HFNO Total	NIV Events	NIV Total	Weight	Odds Ratio M-H, Random, 95% CI
Lockstone 2022	7	65	3	64	19.2%	2.45 [0.61, 9.95]
Stephan 2015	58	414	57	416	80.8%	1.03 [0.69, 1.52]
**Total (95% CI)**		**479**		**480**	**100.0%**	**1.21 [0.62, 2.38]**
Total events	65		60			

Heterogeneity: $Tau^2 = 0.11$; $Chi^2 = 1.38$, df = 1 ($P = 0.24$); $I^2 = 28\%$

Test for overall effect: Z = 0.56 (P = 0.57)

Odds Ratio M-H, Random, 95% CI

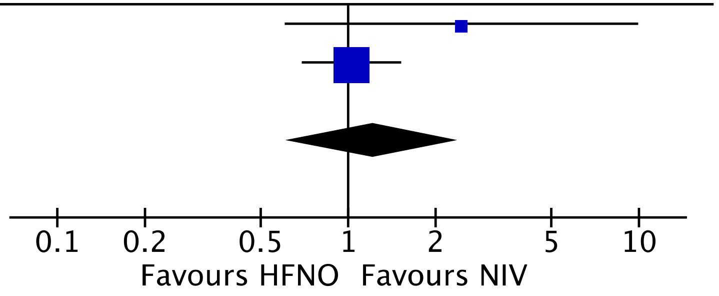

Supplementary Digital Content 11. Network diagrams for primary and secondary outcomes

The nodes represent different treatments being compared. The node size is proportional to the number of patients assigned to each group, and line thickness is proportional to the number of trials directly comparing interventions, which is reported inside the line. Next to each intervention label, the number of patients (n) is reported. Abbreviations: COT, conventional oxygen therapy; HFNO, high-flow nasal oxygen; CPAP, continuous positive airway pressure; NIV, noninvasive ventilation.

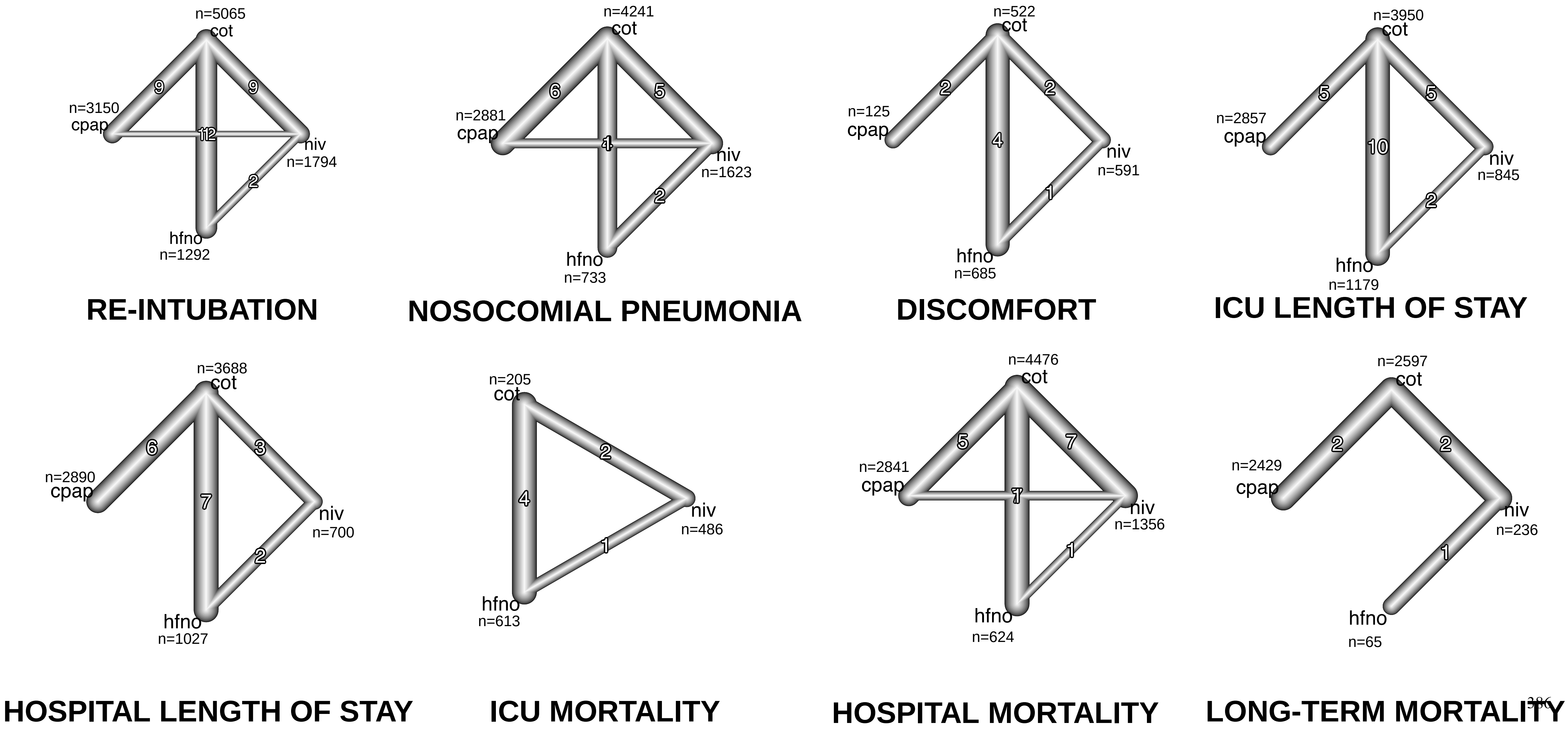

Supplementary Digital Content 12. Network estimates evaluating the impact of the interventions

Supplementary Table 9. Network estimates evaluating the impact of the interventions on re-intubation in sensitivity analyses on re-intubation in sensitivity analyses					
**Comparison**	**SMD or OR (95% CI)**	**p-value**	**I^2 (95% CI)**	**Tau^2**	**K**
Exclusion of studies investigating a sequential combination of NRS modalities					
HFNO vs. COT	0.69 [0.34; 1.38]	0.291	62.3% [43.8%; 74.8%]	0.6525	12
CPAP vs. COT	0.52 [0.21; 1.29]	0.159			7
NIV vs. COT	0.50 [0.28; 0.89]	0.018			9
CPAP vs. HFNO	0.77 [0.25; 2.35]	0.640			0
HFNO vs. NIV	1.39 [0.62; 3.08]	0.425			2
CPAP vs. NIV	1.06 [0.39; 2.89]	0.909			1
Exclusion of studies requiring the application of the continuity correction					
HFNO vs. COT	0.67 [0.33; 1.37]	0.275	63.1% [45.1%; 75.3%]	0.6473	11
CPAP vs. COT	0.47 [0.21; 1.06]	0.067			8
NIV vs. COT	0.49 [0.27; 0.87]	0.016			9

CPAP vs. HFNO	0.70 [0.24; 2.03]	0.506			0
HFNO vs. NIV	1.37 [0.61; 3.08]	0.440			2
CPAP vs. NIV	0.96 [0.37; 2.45]	0.926			1
Exclusion of studies at high, serious or critical risk of bias					
HFNO vs. COT	0.69 [0.30; 1.59]	0.387	55.9% [28.1%; 73.0%]	0.6619	10
CPAP vs. COT	0.48 [0.20; 1.17]	0.106			8
NIV vs. COT	0.29 [0.13; 0.68]	0.004			4
CPAP vs. HFNO	0.69 [0.21; 2.33]	0.554			0
HFNO vs. NIV	2.38 [0.85; 6.64]	0.100			1
CPAP vs. NIV	1.65 [0.48; 5.60]	0.424			0
HFNO 40 L/min in Theologou et al. and NIV with mask in Yang et al.					
HFNO vs. COT	0.72 [0.36; 1.44]	0.352	61% [42.4%; 73.6%]	0.6271	12
CPAP vs. COT	0.49 [0.22; 1.08]	0.076			9
NIV vs. COT	0.52 [0.29; 0.93]	0.028			9

CPAP vs. HFNO	0.68 [0.24; 1.92]	0.472			0
HFNO vs. NIV	1.37 [0.62; 3.03]	0.434			2
CPAP vs. NIV	0.94 [0.38; 2.35]	0.893			1
HFNO 40 L/min in Theologou et al. NIV with helmet in Yang et al.					
HFNO vs. COT	0.69 [0.34; 1.41]	0.309	62.2% [44.4%; 74.4%]	0.6752	12
CPAP vs. COT	0.48 [0.22; 1.07]	0.071			9
NIV vs. COT	0.46 [0.25; 0.84]	0.011			9
CPAP vs. HFNO	0.69 [0.24; 1.98]	0.493			0
HFNO vs. NIV	1.50 [0.67; 3.38]	0.329			2
CPAP vs. NIV	1.04 [0.41; 2.65]	0.939			1
HFNO 60 L/min in Theologou et al. NIV with mask in Yang et al.					
HFNO vs. COT	0.65 [0.33; 1.31]	0.228	59.8% [40.4%; 72.9%]	0.6004	12
CPAP vs. COT	0.49 [0.23; 1.07]	0.073			9
NIV vs. COT	0.51 [0.29; 0.91]	0.022			9

CPAP vs. HFNO	0.75 [0.27; 2.10]	0.591			0
HFNO vs. NIV	1.27 [0.58; 2.78]	0.554			2
CPAP vs. NIV	0.96 [0.39; 2.36]	0.923			1
HFNO 60 L/min in Theologou et al. and NIV with helmet in Yang et al.					
HFNO vs. COT	0.63 [0.31; 1.27]	0.197	61.1% [42.5%; 73.7%]	0.6469	12
CPAP vs. COT	0.48 [0.22; 1.06]	0.068			9
NIV vs. COT	0.45 [0.25; 0.82]	0.009			9
CPAP vs. HFNO	0.76 [0.27; 2.17]	0.613			0
HFNO vs. NIV	1.38 [0.62; 3.10]	0.429			2
CPAP vs. NIV	1.06 [0.42; 2.67]	0.908			1
Consideration of those studies comparing either CPAP or NIV to COT in the same group					
HFNO vs. COT	0.69 [0.36; 1.33]	0.267	61.3% [42.8%; 73.8%]	0.4971	12
CPAP/NIV vs. COT	0.50 [0.32; 0.78]	0.003			18
CPAP/NIV vs. HFNO	0.72 [0.35; 1.47]	0.366			2

Abbreviations: SMD, standardized mean difference; OR, odds ratio; CI, confidence interval; I^2, within-design heterogeneity; Tau^2, between-design inconsistency; K, number of studies providing direct evidence for each outcome; NRS, non-invasive respiratory support; HFNO, high-flow nasal oxygen; COT, conventional oxygen therapy; CPAP, continuous positive airway pressure; NIV, non-invasive ventilation.

**Supplementary Table 10. Network estimates evaluating the impact of the interventions on re-intubation in patient subgroups**					
**Comparison**	**SMD or OR (95% CI)**	**p-value**	**I^2 (95% CI)**	**Tau^2**	**K**
Prophylactic intervention					
HFNO vs. COT	0.85 [0.38; 1.90]	0.697	41.2% [0.0%; 66.4%]	0.4583	10
CPAP vs. COT	0.53 [0.22; 1.27]	0.152			6
NIV vs. COT	1.33 [0.56; 3.19]	0.516			3
CPAP vs. HFNO	0.62 [0.19; 2.02]	0.425			0
HFNO vs. NIV	0.64 [0.22; 1.86]	0.413			1
CPAP vs. NIV	0.39 [0.11; 1.36]	0.141			0
Therapeutic intervention					
HFNO vs. COT	0.91 [0.15; 5.65]	0.921	70.2% [40.7%; 85.0%]	0.9102	2
CPAP vs. COT	0.33 [0.08; 1.49]	0.151			3
NIV vs. COT	0.23 [0.09; 0.58]	0.002			5
CPAP vs. HFNO	0.37 [0.03; 3.87]	0.405			0
HFNO vs. NIV	3.93 [0.51; 30.24]	0.189			0
CPAP vs. NIV	1.44 [0.31; 6.68]	0.639			1
High risk patients					
HFNO vs. COT	0.50 [0.18; 1.43]	0.198	51.3% [0.0%; 77.2%]	0.5580	4
CPAP vs. COT	0.54 [0.05; 5.78]	0.612			2
NIV vs. COT	0.36 [0.14; 0.93]	0.034			3
CPAP vs. HFNO	1.08 [0.08; 14.41]	0.953			0

HFNO vs. NIV	1.39 [0.51; 3.77]	0.518			2
CPAP vs. NIV	1.50 [0.12; 19.22]	0.754			0
Low risk patients					
HFNO vs. COT	0.81 [0.29; 2.23]	0.682	67.7% [48.6%; 79.7%]	0.8091	8
CPAP vs. COT	0.48 [0.20; 1.18]	0.111			7
NIV vs. COT	0.57 [0.25; 1.28]	0.170			6
CPAP vs. HFNO	0.60 [0.15; 2.32]	0.455			0
HFNO vs. NIV	1.43 [0.39; 5.25]	0.591			0
CPAP vs. NIV	0.85 [0.28; 2.59]	0.777			1
Supra-diaphragmatic surgery					
HFNO vs. COT	0.60 [0.25; 1.46]	0.259	67.8% [48.2%; 80.0%]	0.9848	10
CPAP vs. COT	0.53 [0.11; 2.51]	0.426			2
NIV vs. COT	0.58 [0.26; 1.27]	0.173			7
CPAP vs. HFNO	0.89 [0.15; 5.16]	0.897			0
HFNO vs. NIV	1.04 [0.35; 3.07]	0.946			1
CPAP vs. NIV	0.92 [0.19; 4.45]	0.922			1
Infra-diaphragmatic surgery					
HFNO vs. COT	1.25 [0.37; 4.22]	0.714	28.9% [0.0%; 68.2%]	0.3034	2
CPAP vs. COT	0.59 [0.25; 1.35]	0.209			6
NIV vs. COT	0.57 [0.20; 1.62]	0.288			1
CPAP vs. HFNO	0.47 [0.11; 2.04]	0.311			0
HFNO vs. NIV	2.22 [0.61; 8.06]	0.227			1

CPAP vs. NIV	1.04 [0.27; 3.96]	0.960			0
In ICU					
HFNO vs. COT	0.53 [0.21; 1.31]	0.169	68.8% [50.6%; 80.3%]	0.7640	8
CPAP vs. COT	0.32 [0.10; 1.09]	0.068			3
NIV vs. COT	0.48 [0.25; 0.91]	0.024			9
CPAP vs. HFNO	0.61 [0.14; 2.72]	0.517			0
HFNO vs. NIV	1.11 [0.40; 3.04]	0.846			1
CPAP vs. NIV	0.67 [0.19; 2.40]	0.543			1
Out of ICU					
CPAP vs. COT	0.46 [0.08; 2.72]	0.392	0% [0.0%; 89.6%]	0	3

Abbreviations: SMD, standardized mean difference; OR, odds ratio; CI, confidence interval; I^2, within-design heterogeneity; Tau^2, between-design inconsistency; K, number of studies providing direct evidence for each outcome; HFNO, high-flow nasal oxygen; COT, conventional oxygen therapy; CPAP, continuous positive airway pressure; NIV, non-invasive ventilation; ICU, intensive care unit.

Supplementary Figure 3. Forest plots of the effect of NRS on re-intubation in patient subgroups.

Abbreviations: COT, conventional oxygen therapy; NRS, non-invasive respiratory support; OR, odds ratio; CI, confidence interval; CPAP, continuous positive airway pressure; HFNO, high-flow nasal oxygen; NIV, non-invasive ventilation; ICU, intensive care unit.

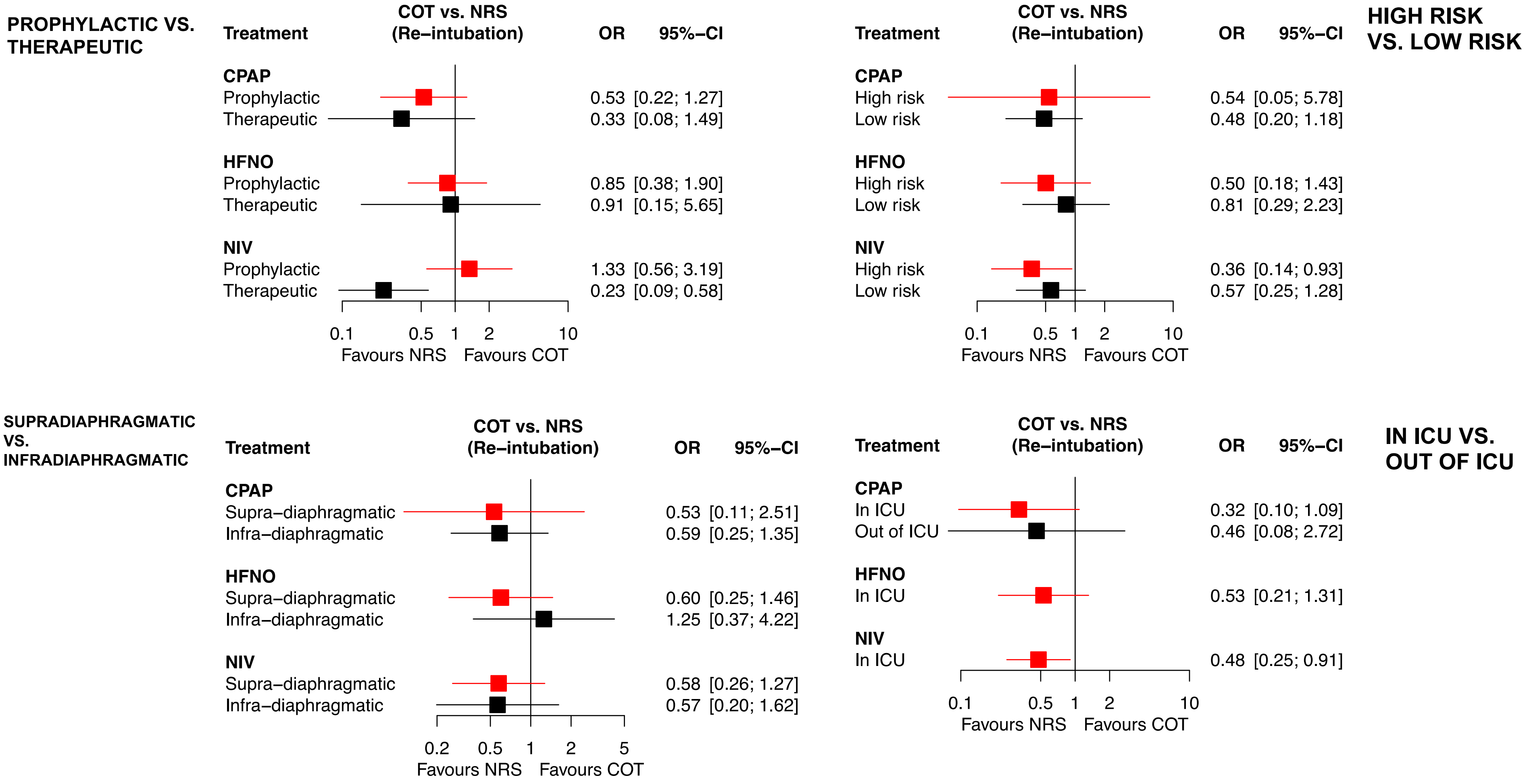

**Supplementary Table 11. Network estimates evaluating the impact of the interventions on re-intubation in patient subgroups when considering those studies comparing either CPAP or NIV to COT in the same group**					
**Comparison**	**SMD or OR (95% CI)**	**p-value**	**I^2 (95% CI)**	**Tau^2**	**K**
Prophylactic intervention					
HFNO vs. COT	0.84 [0.40; 1.75]	0.640	41.4% [0.0%; 66.0%]	0.2708	10
CPAP/NIV vs. COT	0.87 [0.51; 1.47]	0.601			9
CPAP/NIV vs. HFNO	1.03 [0.44; 2.42]	0.937			1
Therapeutic intervention					
HFNO vs. COT	0.91 [0.15; 5.74]	0.924	70.1% [40.6%; 85.0%]	0.9325	2
CPAP/NIV vs. COT	0.25 [0.11; 0.60]	0.002			8
CPAP/NIV vs. HFNO	0.27 [0.04; 2.08]	0.211			0
High risk patients					
HFNO vs. COT	0.53 [0.20; 1.42]	0.207	45.2% [0.0%; 73.7%]	0.4722	4
CPAP/NIV vs. COT	0.39 [0.17; 0.90]	0.027			5
CPAP/NIV vs. HFNO	0.74 [0.29; 1.87]	0.519			2

Low risk patients					
HFNO vs. COT	0.80 [0.31; 2.09	0.652	68.2% [49.4%; 80.0%]	0.5934	8
CPAP/NIV vs. COT	0.53 [0.30; 0.96]	0.035			13
CPAP/NIV vs. HFNO	0.66 [0.22; 2.04]	0.475			0
Supra-diaphragmatic surgery					
HFNO vs. COT	0.60 [0.24; 1.46]	0.257	67.8% [48.1%; 80.0%]	0.9904	10
CPAP/NIV vs. COT	0.57 [0.27; 1.22]	0.148			9
CPAP/NIV vs. HFNO	0.95 [0.33; 2.79]	0.933			1
Infra-diaphragmatic surgery					
HFNO vs. COT	1.39 [0.51; 3.80]	0.519	29.4% [0.0%; 67.3%]	0.1042	2
CPAP/NIV vs. COT	0.64 [0.40; 1.03]	0.064			7
CPAP/NIV vs. HFNO	0.46 [0.17; 1.29]	0.139			1
In ICU					
HFNO vs. COT	0.52 [0.21; 1.29]	0.158	69.2% [51.2%; 80.5%]	0.7820	8
CPAP/NIV vs. COT	0.45 [0.24; 0.82]	0.009			12

CPAP/NIV vs. HFNO	0.86 [0.32; 2.35]	0.771			1
Out of ICU					
CPAP/NIV vs. COT	0.46 [0.08; 2.72]	0.392	0% [0.0%; 89.6%]	0	3

Abbreviations: SMD, standardized mean difference; OR, odds ratio; CI, confidence interval; I^2, within-design heterogeneity; Tau^2, between-design inconsistency; K, number of studies providing direct evidence for each outcome; HFNO, high-flow nasal oxygen; COT, conventional oxygen therapy; CPAP, continuous positive airway pressure; NIV, non-invasive ventilation; ICU, intensive care unit.

Supplementary Figure 4. Forest plots of the effect of NRS on re-intubation in patient subgroups when considering those studies comparing either CPAP or NIV to COT in the same group.

Abbreviations: COT, conventional oxygen therapy; NRS, non-invasive respiratory support; OR, odds ratio; CI, confidence interval; CPAP, continuous positive airway pressure; HFNO, high-flow nasal oxygen; NIV, non-invasive ventilation; ICU, intensive care unit.

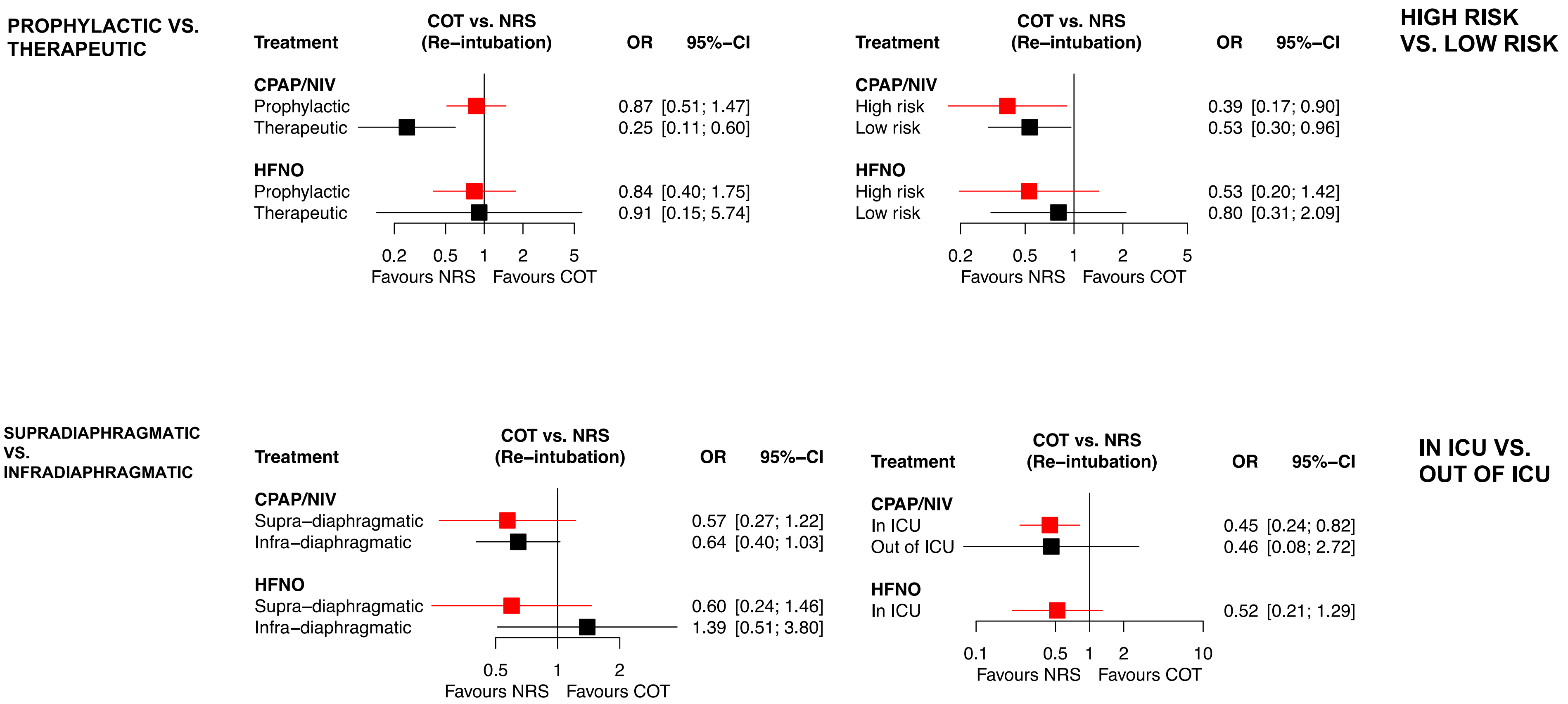

Supplementary Digital Content 13. P-values of the interventions

**Supplementary Table 12. P-values of the interventions for primary and secondary outcomes in the overall patient population**	
**Intervention**	**P-value**
Re-intubation	
COT	0.062
HFNO	0.440
CPAP	0.738
NIV	0.760
Nosocomial pneumonia	
COT	0.042
HFNO	0.538
CPAP	0.666
NIV	0.755
Discomfort	
COT	0.792
HFNO	0.646
CPAP	0.398
NIV	0.164
ICU length of stay	
COT	0.080
HFNO	0.282
CPAP	0.963
NIV	0.676
Hospital length of stay	
COT	0.028
HFNO	0.424
CPAP	0.895
NIV	0.654
ICU mortality	
COT	0.052

HFNO	0.507
CPAP	-
NIV	0.941
Hospital mortality	
COT	0.220
HFNO	0.394
CPAP	0.433
NIV	0.944
Long-term mortality	
COT	0.158
HFNO	0.615
CPAP	0.382
NIV	0.846
Abbreviations: COT, conventional oxygen therapy; HFNO, high-low nasal oxygen; CPAP, continuous positive airway pressure; NIV, non-invasive ventilation; ICU, intensive care unit.	

Supplementary Table 13. P-values of the interventions for re-intubation in sensitivity analyses

Intervention	P-value
Excluding studies investigating a sequential combination of NRS modalities	
COT	0.078
HFNO	0.462
CPAP	0.685
NIV	0.775
Excluding studies requiring the application of the continuity correction	
COT	0.060
HFNO	0.445
CPAP	0.750

NIV	0.745
Excluding studies at high risk of bias	
COT	0.083
HFNO	0.378
CPAP	0.627
NIV	0.912
HFNO 40 L/min in Theologou et al. and NIV with mask in Yang et al.	
COT	0.076
HFNO	0.426
CPAP	0.760
NIV	0.739
HFNO 40 L/min in Theologou et al. NIV with helmet in Yang et al.	
COT	0.065
HFNO	0.419
CPAP	0.729
NIV	0.787
HFNO 60 L/min in Theologou et al. NIV with mask in Yang et al.	
COT	0.054
HFNO	0.486
CPAP	0.736
NIV	0.725
HFNO 60 L/min in Theologou et al. and NIV with helmet in Yang et al.	
COT	0.046
HFNO	0.474
CPAP	0.705
NIV	0.776
Consideration of those studies comparing either CPAP or NIV to COT in the same group	
COT	0.067

HFNO	0.525
CPAP/NIV	0.908
Abbreviations: NRS, non-invasive respiratory support; COT, conventional oxygen therapy; HFNO, high-low nasal oxygen; CPAP, continuous positive airway pressure; NIV, non-invasive ventilation.	

**Supplementary Table 14. P-values of the interventions for re-intubation in patient subgroups**	
**Intervention**	**P-value**
Prophylactic intervention	
COT	0.389
HFNO	0.553
CPAP	0.880
NIV	0.178
Therapeutic intervention	
COT	0.179
HFNO	0.279
CPAP	0.680
NIV	0.862
High risk patients	
COT	0.141
HFNO	0.561
CPAP	0.516
NIV	0.782
Low risk patients	
COT	0.161
HFNO	0.394
CPAP	0.776
NIV	0.669
Supra-diaphragmatic surgery	

COT	0.143
HFNO	0.597
CPAP	0.626
NIV	0.634
Infra-diaphragmatic surgery	
COT	0.297
HFNO	0.209
CPAP	0.740
NIV	0.754
In ICU	
COT	0.043
HFNO	0.532
CPAP	0.812
NIV	0.612
Out of ICU	
COT	0.196
CPAP	0.804
Abbreviations: COT, conventional oxygen therapy; HFNO, high-low nasal oxygen; CPAP, continuous positive airway pressure; NIV, non-invasive ventilation; ICU, intensive care unit.	

Supplementary Table 15. P-values of the interventions for re-intubation in patient subgroups when considering those studies comparing either CPAP or NIV to COT in the same group

Intervention	P-value
Prophylactic intervention	
COT	0.310
HFNO	0.606
CPAP/NIV	0.584
Therapeutic intervention	

COT	0.232
HFNO	0.322
CPAP/NIV	0.947
High risk patients	
COT	0.059
HFNO	0.578
CPAP/NIV	0.863
Low risk patients	
COT	0.172
HFNO	0.456
CPAP/NIV	0.873
Supra-diaphragmatic surgery	
COT	0.101
HFNO	0.669
CPAP/NIV	0.730
Infra-diaphragmatic surgery	
COT	0.386
HFNO	0.165
CPAP/NIV	0.949
In ICU	
COT	0.042
HFNO	0.653
CPAP/NIV	0.805
Out of ICU	
COT	0.196
CPAP/NIV	0.804
Abbreviations: COT, conventional oxygen therapy; HFNO, high-low nasal oxygen; CPAP, continuous positive airway pressure; NIV, non-invasive ventilation; ICU, intensive care unit.	
